# Supplementary material for: hTERT-Driven Immortalization of RDEB Fibroblast and Keratinocyte Cell Lines Followed by Cre-Mediated Transgene Elimination
Source: Int J Mol Sci. 2021 Apr 7;22(8):3809. doi: 10.3390/ijms22083809 (PMC8067634; doi:10.3390/ijms22083809)
Supplement: Supplementary file 1 [file ijms-22-03809-s001.zip › sup/ijms-1131048 sup.docx]

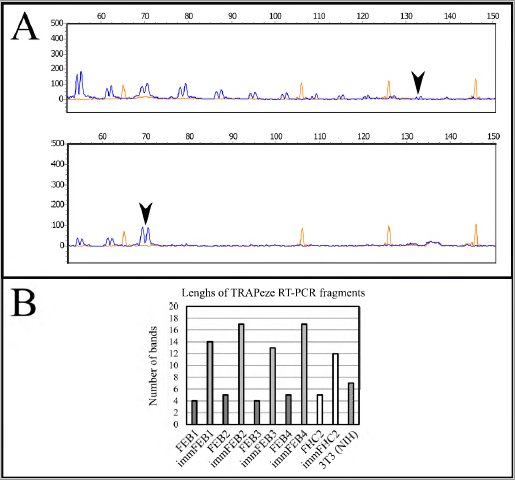


**Figure S1.** Capillary electrophoresis of TRAPEZE probes. The distribution of amplified fragments for one of the pairs of FEB lines immFEB line(A, upper) and FEB line (A, bottom). B. Quantification of the distribution found on capillary electrophoresis of TRAPEZE probes.


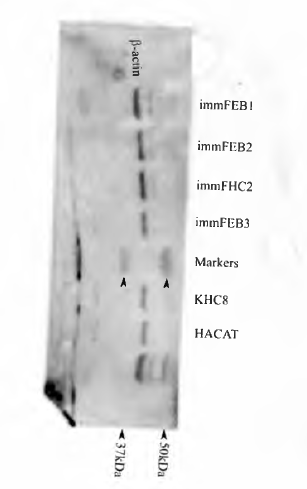


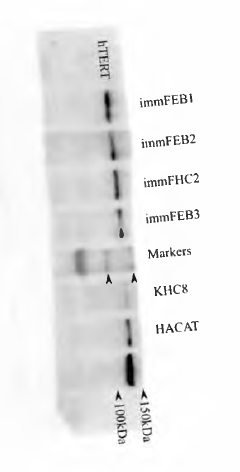


**Figure S2.** The uncut and unadjusted Western blot analysis of hTERT expression. SDS-PAGE (10%), anti-hTERT monoclonal antibody (upper panel), anti-b-actin antibody polyclonal antibody (lower panel), ECL detection.


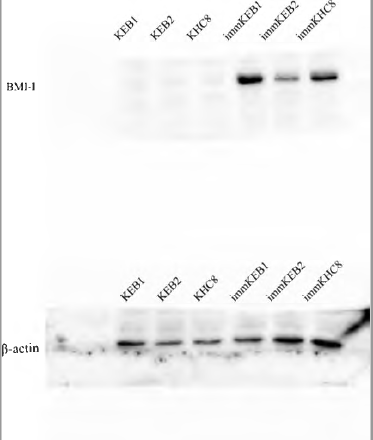


**Figure S3.** The uncut and unadjusted Western blot analysis of BMI-I expression. 10% SDS-PAGE, anti-BMI-I monoclonal antibody (upper panel), anti-lamin B1 antibody monoclonal antibody (lower panel), ECL detection.


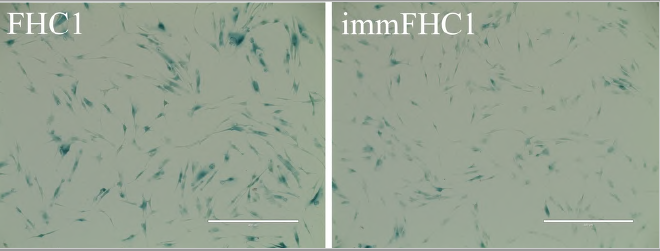


**Figure S4.** Examples of senescence associated-β-galactosidase (SA-β-gal) staining in fibroblasts. Brightfield light microscope EVOS FL AUTO was used. For analyzing staining intensity images were subjected to color deconvolution using FiJi plugin. The XGAL staining and separation of signal described in 4.17.


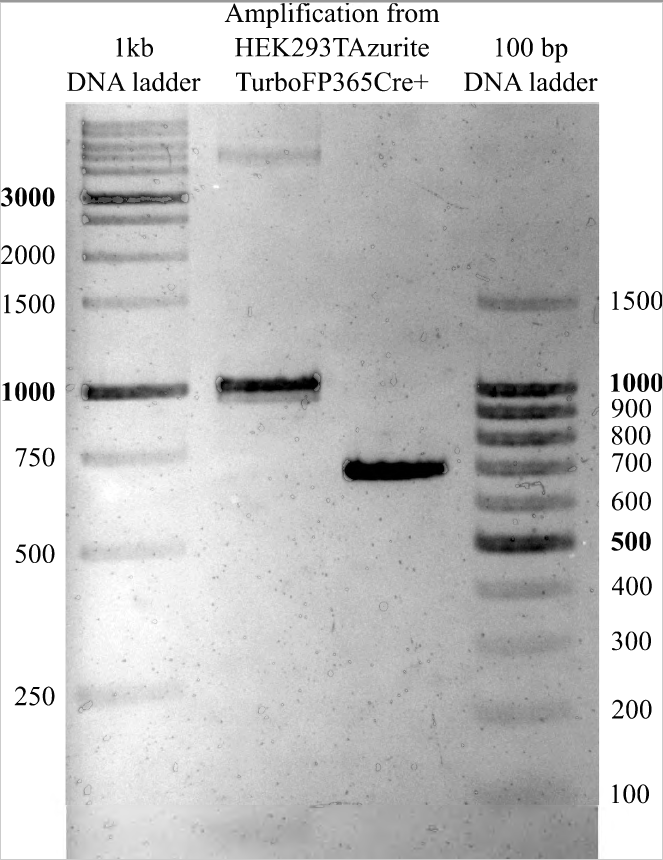


**Figure S5.** PCR amplification of DNA from HEK293T Azurite cells after Cre-mediated recombination. Line 1- DNA ladder. Line 2 - PCR with pGKdir5, Turborev end primers; line 3 - PCR with nested primers pGKdir 4, TurboFP635rev2. The first amplification with pGKdir5 TurboFP635rev end primers produced the band with the expected length of about 1 kb. The product shown in line 3 was obtained from the first product with nested specific primers Turbo rev2 and pGKdir4, revealing the band of 700 base pairs, expected to result from cutting out the target construct. Line4 -DNA ladder.


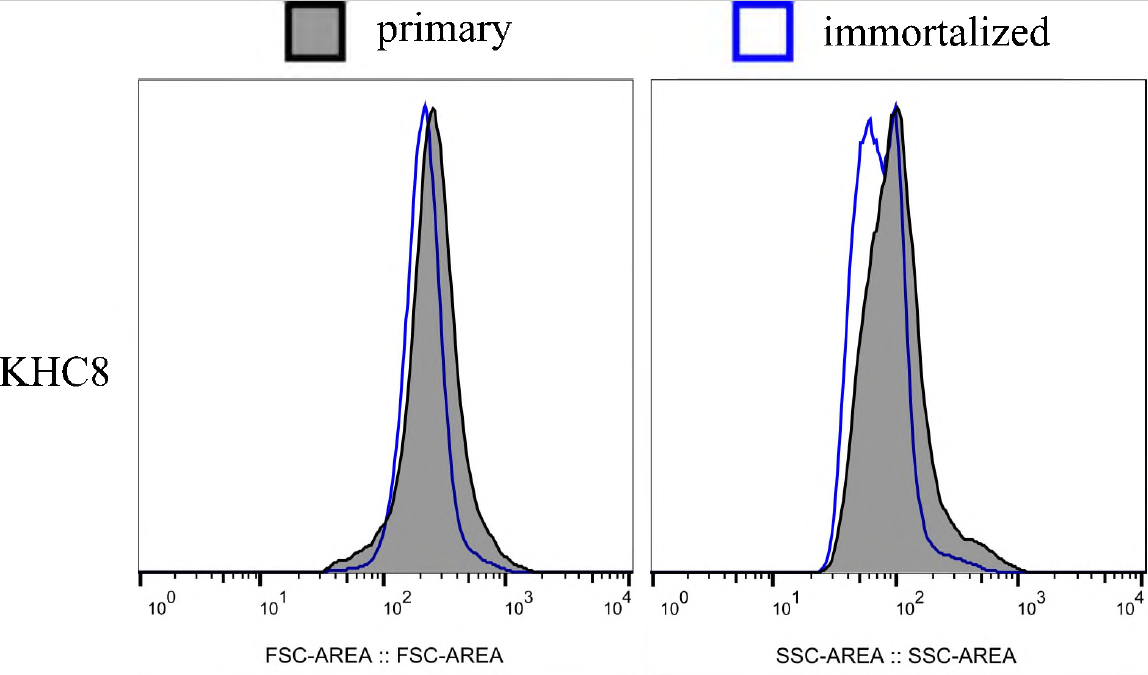


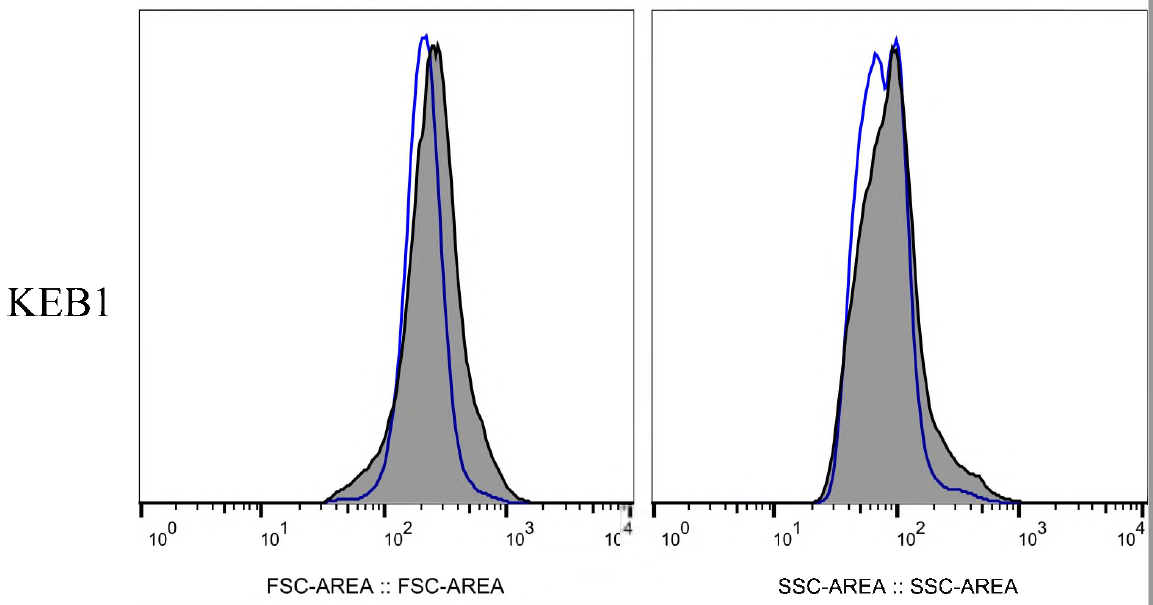


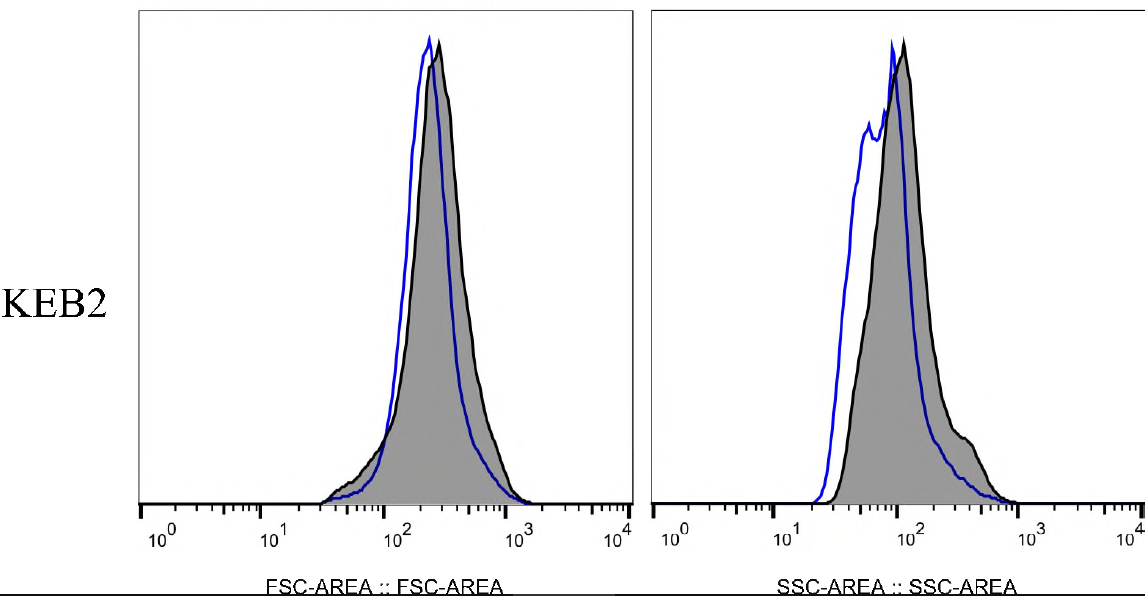


**Figure S6.** Comparison of primary and immortalized keratinocytes cell lines by FSC and SSC flow cytometry parameters by the FlowJo Chi Squared comparison.


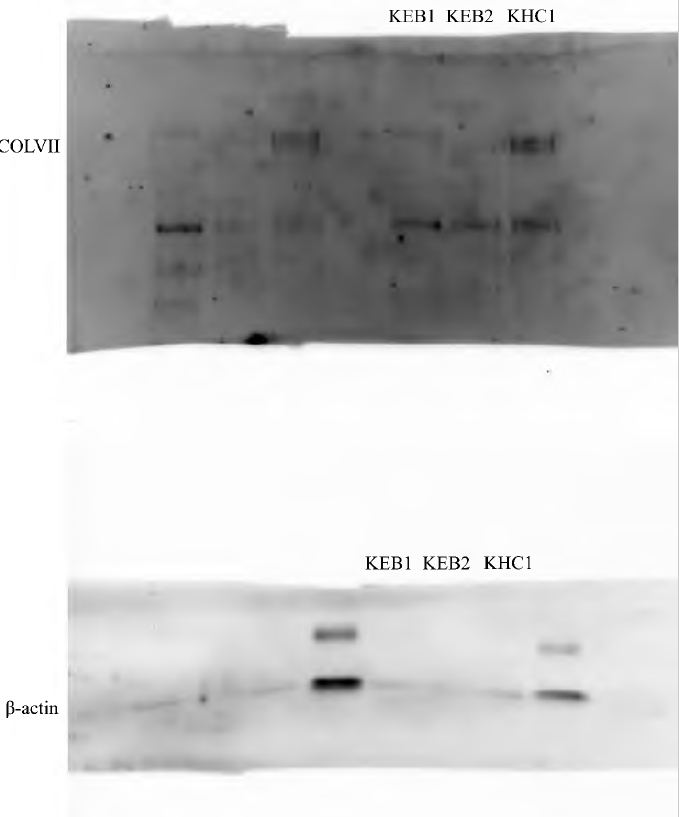


**Figure S7.** The uncut and unadjusted version of western blot analysis of total cell lysates of immortalized keratinocytes; anti-type VII collagen polyclonal antibody (upper panel), anti-b-actin (lower panel).


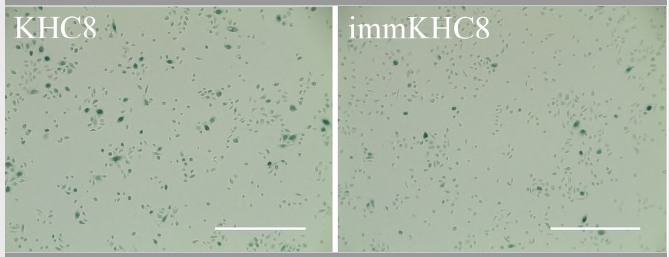


**Figure S8**. Examples of senescence associated-β-galactosidase (SA-β-gal) staining in keratinocytes. Brightfield light microscope EVOS FL AUTO was used.


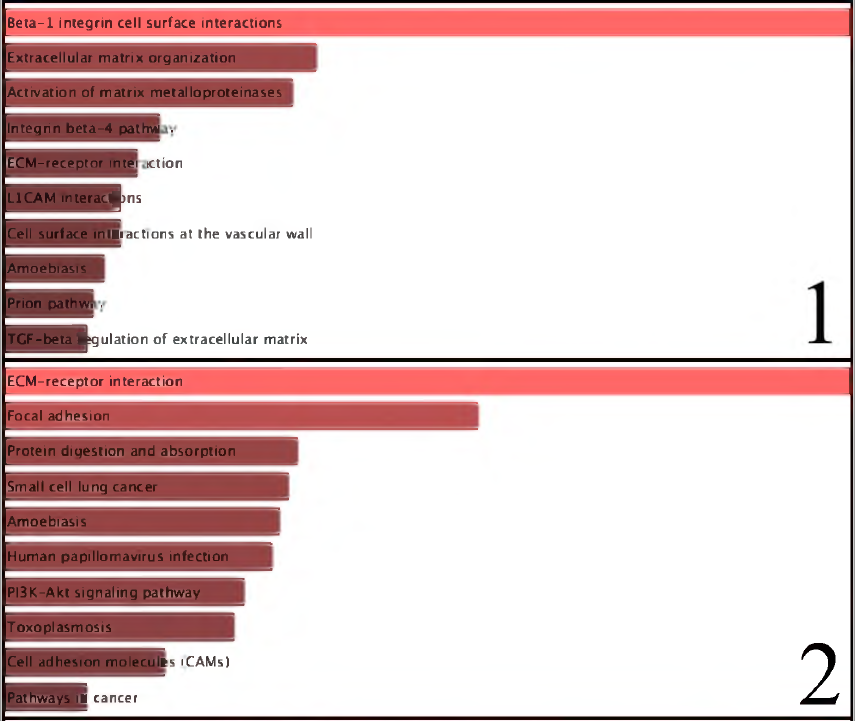


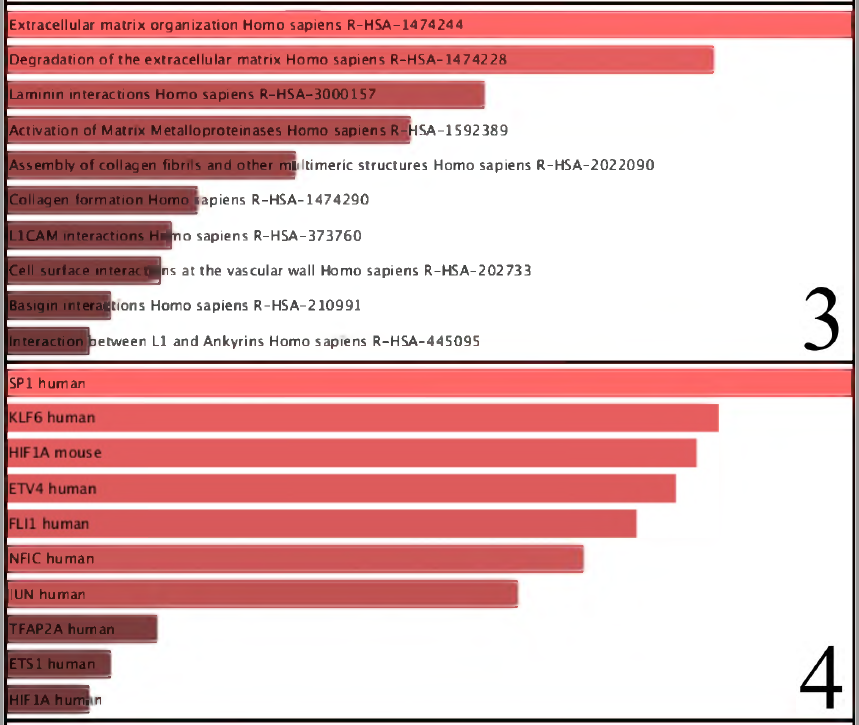


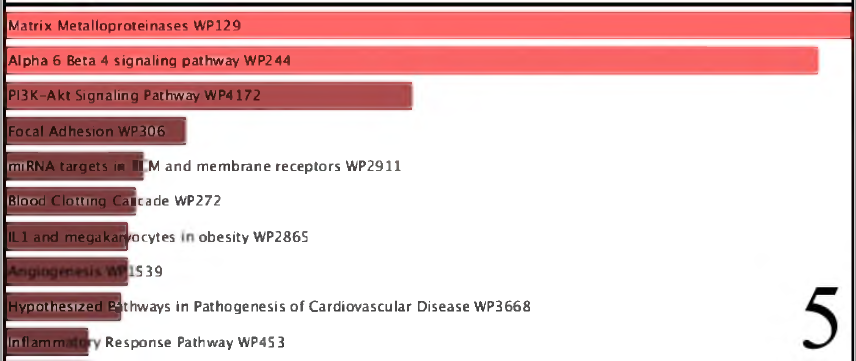


**Figure S9.** Results of EnrichR analysis of DEP found in secretome data, common DEP for immKEB1 and immKEB2 versus immKHC. 1 BioPlanet_2019_bar_graph ; 2 KEGG_2019_Human_bar_graph; 3 Reactome_2016_bar_graph; 4 TRRUST_Transcription_Factors_2019_bar_graph; 5 WikiPathways_2019_Human_bar_graph.


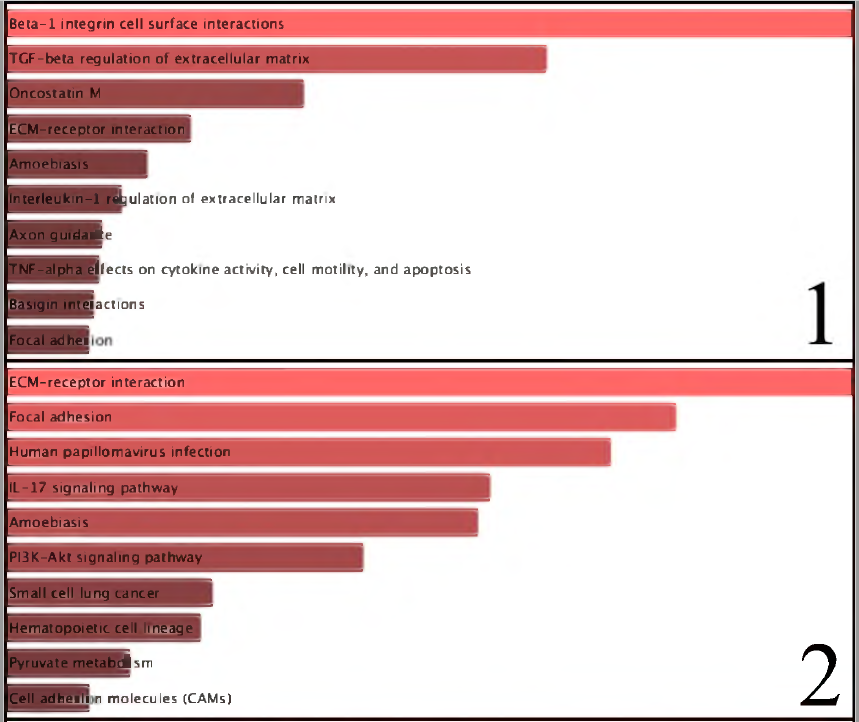


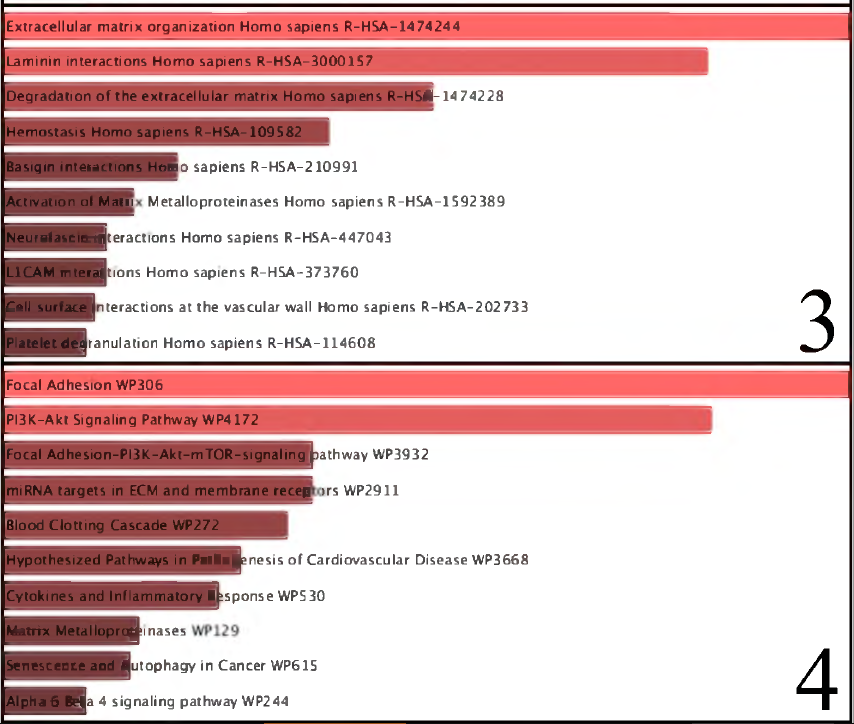


**Figure S10**. Results of EnrichR analysis of DEP found in secretome data, DEP in immKEB1 or immKEB2 versus immKHC. 1 BioPlanet_2019_bar_graph ; 2 KEGG_2019_Human_bar_graph; 3 Reactome_2016_bar_graph; 4 TRRUST_Transcription_Factors_2019_bar_graph; 5 WikiPathways_2019_Human_bar_graph.

**Table S1.** Profile of STR analysis of immortalized cell lines.


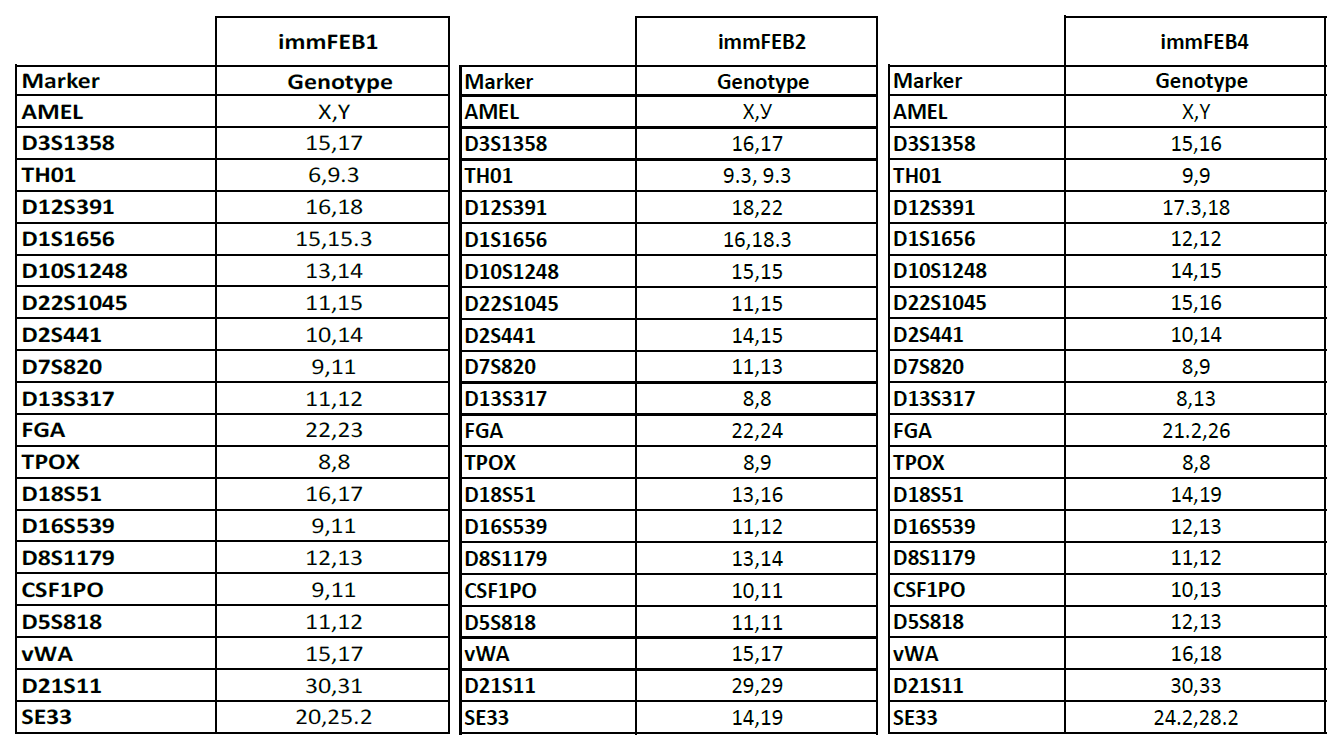


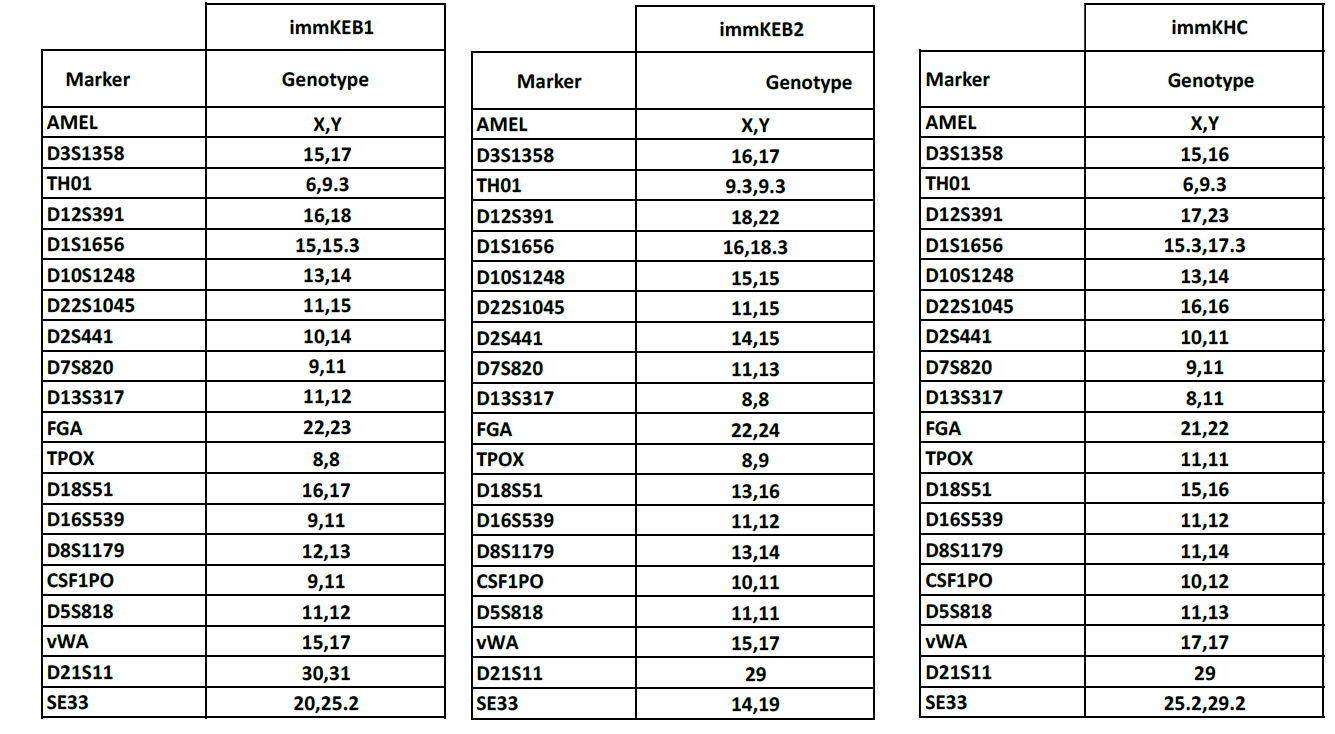


**Table S2.** MaxQuant Data Analysis of Secretomes. Stat 1-2. The comparison of immKEB1 and immKEB2 DEP. Stat 1-3. The comparison of immKEB1 and immKHC. Stat 2-3. The comparison of immKEB2 and immKHC.

(please find it at sup.)

**Table S3**. STRING protein interaction network. Page1 described the network of the list of DEPs found in immKEB1 or in immKEB2 versus immKHC (KEB1&KEB2_KHC); page2 described the network of the list of DEPs found common in immKEB1and in immKEB2 versus immKHC.

(please find it at sup.)
