# Supplementary material for: hTERT-Driven Immortalization of RDEB Fibroblast and Keratinocyte Cell Lines Followed by Cre-Mediated Transgene Elimination
Source: Int J Mol Sci. 2021 Apr 7;22(8):3809. doi: 10.3390/ijms22083809 (PMC8067634; doi:10.3390/ijms22083809)
Supplement: Supplementary file 1 [file ijms-22-03809-s001.zip › sup/Table S2.pdf]

LFQ intens 1	LFQ intens 1	LFQ intens 1	LFQ intens 2	LFQ intens 2	LFQ intens 2	LFQ intens 2	LFQ intens 3	LFQ intens 3
29,78377	31,4543	31,45292	30,42302	31,36656	30,96987	31,01065	30,13394	29,97937
31,83876	32,27556	33,19985	30,84416	30,55536	31,14108	31,12127	28,38928	27,05992
31,03118	32,02697	32,30549	28,31095	28,08035	29,06669	28,91336	20,79574	24,09746
24,49698	28,37501	30,17415	21,70664	24,67246	22,18999	22,98011	22,38854	22,66983
25,30247	23,26814	30,43599	25,17741	22,8621	24,30911	24,60071	22,0442	20,70717
33,83921	33,80713	34,3397	31,56883	31,73376	32,68712	32,62625	29,26591	27,52992
24,74222	24,60337	30,64418	25,10153	25,00449	21,73413	23,56802	21,70214	22,83614
28,93188	30,30051	31,04999	27,05144	26,39643	28,41143	27,3339	23,9612	24,71225
22,99402	22,92194	23,06814	24,61234	24,17456	23,74875	29,0791	27,32199	28,00646
21,99236	23,77998	23,67256	20,3393	20,89156	21,14923	20,90991	20,98905	21,36959
30,68529	30,28703	30,85964	30,81879	30,90928	30,77941	30,85823	30,37399	30,38577
22,19592	22,21376	21,9892	22,62654	23,66371	22,6054	22,51538	23,07883	23,2337
33,43204	33,12132	33,52426	34,14444	34,1734	33,66034	33,77686	34,1496	34,19638
24,80228	24,53925	22,37404	22,42995	25,92134	25,04222	24,20963	24,18624	20,9857
33,44233	33,07312	33,15658	32,61617	32,74256	33,55408	33,48424	32,51371	32,86356
23,0782	24,21573	24,30058	23,73355	23,35205	23,34027	24,0616	23,02624	23,14763
22,09261	23,93129	25,10321	24,61857	23,12668	22,78339	22,55286	22,82993	22,60608
33,00556	32,62901	32,61581	32,94073	32,86133	32,7856	33,02602	33,07253	33,08569
25,96016	26,23316	28,2069	28,04411	27,45591	27,88495	27,38714	26,86667	26,94492
28,59754	29,47365	29,71357	28,63596	28,62502	29,0331	28,81403	28,41393	28,11629
24,54144	24,05358	24,24405	26,21647	26,88285	23,95601	23,44199	25,95476	26,61077
31,73759	30,27275	29,1043	30,81215	30,58262	30,93818	30,58414	32,10942	31,34757
26,97235	27,53875	23,70641	22,08734	23,46811	22,2817	22,63029	27,70695	27,51427
22,93884	22,8041	29,19943	28,65775	22,67563	28,79383	29,29743	22,48849	23,56157
27,09295	26,39926	26,58606	28,29824	28,05274	26,49989	26,80193	28,56384	28,69026
28,13104	28,25503	28,74641	28,37977	28,43305	28,94693	28,85878	27,75085	27,60159
28,65492	30,31646	29,05628	27,62944	27,36328	25,98713	27,53282	24,71282	25,02129
24,86585	25,66858	26,89342	24,55467	24,4985	24,65529	21,50152	24,3995	26,34206
28,38961	29,04604	27,39198	27,05734	28,2615	27,86071	27,11128	28,68201	29,20016
29,42252	28,33654	28,53126	29,20744	28,26616	28,82299	28,99418	29,84019	30,15798
32,96418	32,67141	32,896	33,34889	33,30157	32,60907	33,00887	33,36307	33,28444
28,45391	23,5664	23,91694	23,14386	27,57788	21,32798	23,06441	27,5285	28,11277
26,35839	28,33151	26,54073	25,614	26,20596	27,04458	26,9249	21,48138	26,02842
29,36146	28,99612	29,57028	29,23779	28,91288	28,17327	28,51661	29,26282	28,85243
30,43559	30,01983	30,76928	30,10749	29,93579	30,29734	30,09572	29,60846	29,72095
22,9875	22,37928	25,87683	23,99438	24,1868	26,35328	25,0115	21,9154	24,04902
25,27824	25,41796	23,60397	25,33149	25,43039	26,54541	26,21545	25,56404	25,33248
22,87401	22,50927	23,7104	24,00533	25,3654	23,46208	26,69856	27,58857	27,57975
24,83543	25,70187	25,88129	28,09989	27,96157	27,57521	27,98994	28,46045	28,40068
31,3198	32,1689	30,87943	30,74903	30,78659	30,80301	30,76256	31,63901	31,60342
30,49037	30,49486	30,10011	30,22458	30,02314	29,36208	29,68611	29,85046	29,70337
29,46919	29,0311	30,18648	29,65763	29,75707	28,64516	28,96063	28,84972	29,1438
32,04986	32,27134	32,02815	32,79498	32,75521	31,31802	31,96716	31,79052	32,39749
22,55595	24,25068	24,56715	23,0456	23,46548	22,989	23,09163	22,47135	22,52411
25,95821	26,75468	26,51845	26,03626	26,50314	25,78497	25,75491	23,51093	25,66473
27,68646	22,77694	24,11508	27,24998	26,84374	26,66519	26,03526	27,90133	26,9982
29,76029	28,75646	28,52832	29,17926	28,34744	27,79631	27,73098	27,82171	27,83864
30,69594	30,59067	30,49295	30,91663	30,91748	30,831	30,68361	31,33527	31,499

23,29637	25,09152	25,01103	23,23882	23,89069	25,0916	25,45848	23,61908	24,25263
22,49253	22,52481	25,14794	23,05078	22,8054	21,69381	22,78836	22,47301	22,89599
39,82963	40,3972	40,3675	39,97216	40,09383	39,7945	39,99011	39,55767	39,61191
28,40186	29,72688	29,72761	28,52862	29,12443	28,77982	28,26808	27,7348	28,20983
34,12325	32,30138	33,52869	33,55225	33,47255	32,45425	32,37485	31,58352	31,52687
30,13883	29,47183	28,8308	29,95934	29,9157	29,73702	29,175	29,41877	29,20156
22,62466	25,91079	25,23092	22,80388	24,75694	24,96521	25,17489	22,48554	23,17973
25,66971	25,48395	23,57503	26,97126	26,78694	26,20247	26,27021	26,45172	26,68565
26,47663	27,08861	28,3572	27,35481	27,29658	25,45707	25,58337	26,46857	26,36766
27,38303	28,39734	32,13302	27,33253	26,12108	25,9608	27,51307	23,24587	21,69751
25,05519	28,31765	30,25214	28,23571	26,87373	28,07923	27,23425	27,06764	29,22977
28,35342	27,33415	26,39274	27,92875	27,98555	26,77706	26,85115	27,75793	28,13335
34,81926	34,74259	34,96942	35,25993	35,31477	34,69872	34,8409	35,07112	34,91665
26,38739	29,42734	29,03265	28,95795	27,89891	30,17985	28,8887	28,28105	27,13134
25,75524	24,58153	26,69803	29,68005	28,98682	23,46787	24,53582	27,83545	27,79228
31,47581	29,98983	30,45582	31,25259	31,19366	29,44123	28,88757	32,26411	32,34884
23,90181	23,45889	22,8481	23,49253	23,7263	23,40093	23,49521	23,95961	24,07227
23,96913	23,94351	25,28884	24,42714	23,9736	24,42822	25,31517	24,33688	24,59429
26,46247	26,39802	26,11337	27,12495	26,4502	26,37867	25,8764	26,75824	27,07745
27,74175	26,71686	26,68284	27,62485	28,0773	27,45834	28,0267	27,94447	27,14248
26,32468	28,36611	28,62708	26,59861	26,49697	27,7252	27,80107	26,69432	26,16611
32,49028	32,54522	32,02716	32,55034	32,41956	33,49118	32,99525	31,95491	31,90081
30,75422	30,03604	31,20005	30,95088	30,94831	30,04218	29,87449	29,93227	29,90297
24,87891	25,97367	25,76981	25,20105	24,93405	25,19959	24,85771	23,93373	24,59821
29,45912	29,6348	29,13973	29,22299	29,29959	29,25986	29,29769	28,45709	28,59715
23,09327	22,72077	24,42926	29,01081	29,04226	29,36077	29,08563	28,76284	29,60442
29,35548	22,51285	27,11108	30,4184	29,97528	22,12187	27,80931	31,11166	30,12854
30,29351	30,37098	30,32034	29,59586	29,41542	29,22338	29,18959	28,92872	28,58043
30,80209	30,56074	30,1162	30,4565	30,12842	30,64134	30,5539	30,16949	30,31008
28,33769	28,78279	28,03837	26,8832	27,00131	27,44979	27,41224	27,20602	27,0276
30,47373	29,53393	29,67622	30,88358	30,62104	30,64581	30,31149	29,83079	30,10974
31,64736	31,49781	31,01824	31,2683	31,09339	30,62052	31,00618	31,18943	30,85712
26,02485	22,60765	26,55919	26,79699	23,39697	26,79476	25,26446	22,918	27,45403
30,69917	30,19084	29,30687	30,18459	30,73287	31,3022	31,05705	30,66619	30,62043
29,70017	31,17516	30,40669	29,74439	29,73148	30,72525	31,16249	29,19221	29,52684
32,1478	31,99044	31,91071	32,20597	32,28532	32,09276	32,07431	32,46709	32,60202
36,66924	36,67477	37,06567	36,23622	36,39741	36,92138	36,83975	36,54697	36,51467
28,83641	27,71673	28,26006	29,13582	28,71823	28,22366	28,1248	27,88332	27,59399
31,38469	31,11873	31,19578	31,53723	31,60116	31,29576	31,11471	32,09704	32,07728
29,73119	27,79749	28,15938	27,87701	28,09628	28,40255	28,18778	28,98829	29,14239
26,75468	27,26141	28,06712	27,51209	27,91102	27,01723	27,34706	27,24164	26,99949
24,06817	22,51959	22,60184	26,4321	24,59241	23,57057	24,31043	23,02665	24,03884
28,95074	29,18386	29,2963	28,62383	28,84107	29,17331	29,1775	28,1155	28,13951
23,45989	24,27916	24,54799	24,41886	21,94694	24,67745	24,417	24,42873	24,85529
25,5526	23,09792	24,06357	27,97301	27,77335	26,13055	25,72093	27,91296	27,27293
29,78544	27,05631	26,84074	30,28824	29,64406	28,51537	28,73463	30,31959	30,06958
31,08772	30,64736	31,39719	31,11787	31,49815	31,23026	31,08715	31,30883	31,2161
22,48696	23,48598	22,96295	25,59269	25,5305	25,10185	25,35279	24,66536	25,05383
26,77568	23,67122	27,37426	26,84182	26,88344	26,01937	25,8469	26,18799	26,702
23,47735	23,90008	23,88922	23,2737	23,33624	23,76486	23,2089	23,51364	24,24561

22,53613	23,74615	23,54647	25,32358	24,49582	24,10509	23,78548	23,13526	24,43243
28,64038	28,47406	29,14611	29,47485	28,93306	28,28498	28,2889	27,83286	28,09053
29,46527	28,86555	29,29649	28,86938	29,17762	28,87132	28,94419	29,59793	29,31106
27,06938	28,89143	28,26526	27,44261	27,41973	27,19639	27,51796	26,59974	26,78207
31,45405	31,32876	30,25327	31,0185	31,25175	30,93227	31,12368	31,43251	31,40009
32,5143	31,86172	32,04274	32,38851	32,54153	32,39657	32,43649	32,79676	32,60309
30,11496	29,17999	28,50233	30,25777	29,8096	29,40576	29,48535	30,18707	29,51655
27,49579	26,13406	25,21182	26,31403	26,73376	27,57087	27,8052	28,65363	28,81427
31,02373	31,7677	31,75561	31,30721	31,30948	31,69499	31,41825	30,72972	31,02281
27,14092	26,77228	27,16959	27,53349	27,53705	27,61034	27,64972	27,46521	27,64113
28,46443	27,67445	27,16615	27,87496	22,43601	28,38059	27,90029	29,15318	29,11966
26,15194	26,13663	26,40391	26,1744	25,58695	25,60925	25,44594	24,83359	24,6608
28,86977	28,23768	28,17874	27,6006	27,57167	27,58914	27,88367	28,00774	27,87121
27,03601	26,83581	26,9506	22,51562	27,15808	26,49095	26,48205	27,01041	26,90633
24,22059	25,35528	25,29018	24,60156	24,26556	24,5428	24,76157	24,10556	22,01563
28,37248	27,89389	29,32772	28,45128	27,78457	27,89192	27,72858	28,12623	27,99205
26,14332	23,01189	24,33834	27,70088	23,52517	23,64966	22,34579	27,47839	28,50415
27,54399	25,67411	26,14766	27,58628	27,5059	27,32174	27,3808	28,95366	29,51783
22,40098	23,81693	23,63272	26,63145	26,90392	27,20909	22,62445	26,7656	26,85222
28,62429	26,96345	27,71476	28,46848	28,2867	27,39526	27,73841	28,50469	28,57838
28,73053	24,52453	28,43924	28,86414	28,93092	28,77878	22,56012	23,16895	28,88824
22,22519	24,05714	23,08479	20,84115	20,80986	20,17731	20,20192	21,1201	21,69238
21,90548	23,68897	23,85056	25,06817	23,37174	25,15623	24,58164	22,24401	21,58573
27,4773	26,69458	28,40807	28,08233	27,94078	26,12778	26,64045	27,4259	27,39395
25,24107	23,69519	23,87995	25,01504	24,50355	23,90812	24,18526	26,31443	26,06079
30,49486	31,13663	32,0949	30,66195	29,75248	32,0186	31,615	30,53557	30,70471
27,68759	27,50385	28,24354	28,02189	26,97913	26,97978	27,68746	26,87467	26,90323
27,59634	27,47057	27,37923	28,11753	27,47908	28,82642	28,21933	28,18834	28,32693
22,38697	23,94143	25,0925	25,21749	22,11606	22,6314	25,75771	24,90724	22,14161
23,78216	26,74496	26,26912	26,66655	26,46883	26,35175	26,35805	26,64733	26,30196
33,49441	32,92128	32,59539	32,3817	32,26994	32,90683	32,90919	33,27711	33,37036
23,67721	22,78137	23,41876	26,10536	26,18569	25,36903	25,84886	23,16877	21,86169
25,58569	23,1495	22,57251	24,56248	27,52709	26,79613	26,39562	25,28355	24,47191
27,75513	28,21183	27,7604	26,90529	26,82905	27,53646	26,94905	26,56261	26,16398
29,76917	28,95427	28,57236	29,82902	30,14722	29,61994	30,10762	31,08759	31,39622
27,97104	24,13355	23,53183	25,66793	26,47122	22,09738	26,35919	29,65397	28,37335
24,58526	25,44518	25,80934	25,38788	25,90371	25,1848	25,36262	25,01636	25,70197
26,35753	25,14492	26,15178	27,13575	27,41579	25,93001	25,80102	26,41308	26,95005
29,00715	28,8387	29,76729	29,54305	29,31948	28,25399	29,28131	28,55421	28,74156
29,21792	32,58394	30,50654	28,12623	30,26919	29,19049	29,2372	26,63409	22,71205
29,22757	23,924	26,58721	27,54399	21,78727	27,06548	26,48171	29,63819	30,59495
29,96939	29,87926	28,24984	28,92269	29,53094	29,31214	29,26177	30,53853	30,19531
27,03149	27,33142	27,70081	27,11038	27,41716	26,63048	27,27622	26,92785	27,31829
30,34467	30,25372	30,40761	30,49343	30,13198	29,22607	29,44213	29,93312	29,39983
26,32978	25,5773	25,67737	27,30042	27,01595	27,28569	26,81115	26,51752	27,29413
23,30494	25,02753	24,20769	21,66731	21,06474	24,80292	24,66129	23,87211	23,80055
22,7308	23,63123	24,27583	26,40868	26,53424	27,85412	27,98462	27,96505	27,55311
23,01668	23,20372	24,27764	26,49803	26,36123	21,74912	26,1163	23,94667	22,99179
23,00669	23,45689	22,91417	22,25534	24,40763	23,71639	23,2798	23,6228	24,35692
23,84414	24,56426	24,14361	26,90047	27,01797	23,04612	26,99194	23,29207	21,7728

27,69737	26,80772	26,85555	28,44892	27,86455	28,01143	27,57427	28,09119	28,61376
32,21095	32,23803	32,37464	32,07061	31,97004	32,08949	31,83217	31,84176	31,17146
26,73104	25,19689	24,38988	22,31128	25,25555	24,68581	23,04007	23,24094	22,1104
23,23996	24,51734	23,0489	24,38555	22,96906	23,09225	23,3615	21,63014	21,64979
33,1712	32,76987	31,82775	32,78755	32,74049	32,65612	32,74585	33,03581	32,32381
33,56207	33,87646	33,62843	33,68301	34,18844	34,17913	34,08417	33,73721	32,55218
23,21048	24,95903	25,34147	24,46065	26,02003	22,13707	22,23981	25,55888	25,82129
28,62048	28,59463	28,16322	28,52099	28,36035	28,34617	28,57015	27,47212	27,3232
27,19563	26,77944	26,91718	26,77291	27,0135	27,20844	26,25686	26,69485	26,17672
29,05012	28,27177	27,92156	28,84554	27,93123	28,2327	28,08618	28,54197	28,47119
28,27728	24,215	24,57643	27,95427	26,79699	22,89161	26,77744	28,58499	28,03601
30,5313	28,74301	29,35727	30,95387	30,81261	29,55379	28,98414	30,88664	30,98956
22,72538	22,34425	21,81765	23,43149	21,9234	21,65298	21,76786	22,38853	21,80982
25,93488	26,56552	26,27538	27,25953	26,7604	27,72702	27,55186	27,5742	27,85792
30,49895	30,54563	31,03636	31,73884	31,96558	31,36124	31,4488	31,45983	31,59072
29,78499	29,14092	28,8289	29,47299	29,28615	29,43408	29,58163	29,46194	29,64397
23,25643	23,04572	22,66345	23,46148	22,90094	23,43582	23,54321	24,35057	24,77258
29,98251	29,46474	29,52045	29,93818	30,00638	29,49331	29,8263	30,06111	29,98861
30,53399	31,64255	31,45988	32,47361	31,34022	21,94433	30,41901	30,48693	23,17258
29,03701	27,4182	27,61744	30,29428	30,01478	29,87616	30,71498	26,92717	27,37103
29,41945	28,1755	27,99572	28,05388	27,88745	28,8979	28,90722	28,08811	28,4265
29,86488	29,6667	29,86606	30,12139	29,96637	29,59092	29,0759	29,22869	29,25798
26,29667	25,03846	24,52492	25,97248	25,33504	25,60096	26,30412	24,09491	24,71654
27,38492	28,44091	27,86685	27,42926	27,12416	28,70606	28,12297	27,77769	27,13065
29,24704	28,01919	28,46486	27,12475	27,50127	27,40162	27,56761	28,76853	29,01962
26,1668	27,14763	26,99043	26,60442	26,65335	27,12839	27,13193	25,5854	25,88229
22,12114	23,35111	23,77726	22,72528	23,21424	22,32888	23,98546	24,90405	23,08309
21,43329	21,26316	23,21829	21,77338	21,94389	23,43033	22,17335	21,88558	23,09263
23,27266	23,05515	24,49419	28,20527	23,44089	22,41971	23,52586	28,58692	28,11098
25,57245	23,58268	25,70585	23,47914	23,83335	25,23169	24,42541	23,34204	24,55397
27,6803	22,83008	27,2553	26,95638	27,37625	28,4215	27,46886	28,86166	27,3435
26,15642	26,49176	26,30198	26,3562	26,16638	26,56796	26,51907	26,16265	26,14602
27,24336	28,7107	23,02098	27,99916	28,12204	28,44762	28,63786	28,07086	28,57532
22,48635	23,09693	24,33101	23,50749	23,4279	23,46438	23,98442	22,792	22,30644
26,7406	27,39648	27,47885	26,64636	26,88367	27,06569	27,16873	27,61147	27,93061
27,87473	26,84828	26,64403	24,01014	22,0047	23,69696	22,98111	23,15847	24,53848
31,02538	28,0608	26,95283	29,20121	28,55677	20,3209	23,47313	29,68667	28,82845
27,63319	25,07255	25,58253	28,68067	28,78544	27,80101	27,63409	28,96416	29,14397
23,18387	21,93488	23,89936	22,62006	22,4368	23,5451	22,93451	22,33549	22,08537
26,06747	26,14028	26,19261	25,5405	24,27242	24,804	22,52962	23,97202	24,54699
20,66865	22,29856	22,86931	21,98116	22,22385	21,91947	22,37928	22,00295	21,68611
22,87826	27,17693	26,35365	27,31077	22,16631	26,01827	23,9734	28,36415	26,89446
22,46193	24,14566	23,93981	22,21463	22,41504	22,0996	22,17139	22,28238	22,73144
29,34995	30,30791	29,86489	29,35098	29,56202	29,8237	30,01265	29,09605	29,22467
25,43825	25,71874	26,0371	25,00924	25,57306	25,1314	25,22902	23,40778	24,62851
33,84633	33,18176	32,9191	34,94026	34,86277	34,7928	34,7456	34,83479	34,94576
30,32571	30,38061	29,69471	28,77445	28,88037	28,9153	29,22858	29,26152	29,23364
26,60484	26,35755	27,0202	27,37708	27,16029	27,31363	27,36853	25,76352	26,29381
28,24173	28,04682	28,11981	28,26481	28,57275	28,65615	28,37683	27,85293	27,32852
28,94824	27,49694	26,82177	27,39534	26,58663	28,36507	28,20186	27,2893	27,09305

31,21714	31,15509	31,74727	29,25818	28,87643	29,77217	30,3184	29,67677	28,89209
23,78097	24,24307	25,10033	24,19753	23,75817	24,66096	24,51147	24,51244	24,22118
25,80181	27,30374	27,68117	28,20662	28,06332	27,5666	27,60625	26,79959	27,09073
30,52673	30,50503	30,39479	30,92741	30,84236	31,31484	31,244	31,09213	31,13589
30,80186	30,69835	30,70347	31,23186	31,23181	30,83996	30,74166	31,29082	31,40202
20,94663	25,10793	29,54775	29,44782	23,03416	29,40538	29,19777	29,74551	28,54878
28,80999	28,71509	28,60918	28,03239	28,31825	28,30138	28,43583	29,45138	29,59557
32,08724	31,52272	31,39018	31,12805	31,05349	30,89829	31,05226	32,28653	32,35935
21,37512	22,7043	21,79578	23,34055	23,29488	23,16682	23,1216	21,44602	21,38864
21,08503	22,76161	23,87801	23,80747	22,82011	21,57761	22,86011	27,84356	27,88681
26,98512	23,88829	25,92445	25,79927	25,93492	27,40965	27,26428	27,23726	27,24245
28,7162	28,86865	28,6635	28,6732	28,22605	28,45517	28,25948	28,63775	28,65458
29,0598	29,72306	29,90124	28,15528	28,18253	29,42274	29,04757	27,12495	27,30496
22,41892	21,9556	26,00719	26,80587	27,00089	26,6245	26,67681	26,63506	26,22015
29,99105	30,73731	30,59566	28,90779	28,90976	30,09043	30,21048	29,82221	29,62558
26,36351	24,09426	23,43975	21,1175	25,6198	24,95832	25,10386	25,21593	25,46588
31,72082	30,41649	30,87298	32,31355	32,22556	30,76975	31,00691	32,54494	32,64534
32,07333	31,20705	31,79579	32,15551	32,04954	31,58289	31,57808	31,72428	31,43226
30,47383	30,22205	30,35374	30,26953	29,9057	30,05892	30,18966	29,8763	29,84151
27,64952	27,24627	27,75015	26,81982	27,20051	27,89735	27,65895	26,94213	27,03276
28,17693	22,669	27,6284	28,16111	28,41679	28,05352	27,52716	28,37902	28,13536
32,43964	31,66072	31,17665	34,3535	34,38985	33,92344	33,84763	34,53907	34,4746
27,63437	24,60421	23,3362	27,51187	27,37152	22,66718	24,74349	27,94436	27,13663
28,76202	29,10482	27,51923	29,5136	29,89116	30,42722	29,85855	29,03499	29,16816
31,91812	31,49729	31,76746	31,74021	32,05985	32,19759	32,46127	32,38785	32,43547
28,07572	28,64599	30,51625	25,26263	24,3812	26,74098	27,54458	24,61745	25,62589
27,91536	26,85626	27,82019	27,62401	27,78625	27,58771	27,64746	26,86207	27,31173
27,46544	27,27044	28,29281	28,24812	28,54374	27,47576	27,75251	27,67721	27,64856
23,619	22,80758	24,78621	24,2452	21,4702	22,93478	22,62632	27,23781	26,85875
27,90621	23,60109	25,8839	28,06358	28,09984	22,3937	22,48816	28,32225	27,60406
31,3891	32,20644	31,21784	31,12712	31,16075	32,05462	31,98217	31,1827	31,06676
35,727	35,08001	34,21993	35,31125	35,36708	34,7692	34,89767	35,99507	36,21522
27,78075	25,62995	26,9862	28,32822	27,9165	26,24567	26,04947	28,63682	27,9645
26,8405	27,13956	27,20359	26,94044	27,01073	26,72546	26,91319	27,10179	26,94949
33,14163	33,0862	33,01676	32,73808	32,75444	32,81086	32,49651	32,88815	32,58511
28,97509	28,84161	27,99226	28,70902	29,11279	29,50673	29,20676	29,74953	29,41581
26,53551	26,10412	25,55915	26,66384	25,96404	26,32924	26,47822	26,15151	25,37518
24,86117	24,58233	24,77077	24,16615	21,33634	22,8082	23,02755	24,55356	23,5323
27,41345	27,08192	26,77027	26,86479	27,41401	25,27459	25,02644	26,20646	25,87446
27,04302	26,42065	26,76307	27,92224	27,7395	26,83509	26,89018	27,84667	27,64746
32,24073	32,4552	32,24505	30,98346	31,16423	32,62991	32,65057	31,34409	31,77999
32,34393	32,45435	32,4553	32,94348	32,95486	32,43572	32,60366	32,71596	32,97266
30,05672	29,71406	30,27453	30,81138	30,90792	29,45875	29,50932	30,14819	30,21037
25,77042	26,13144	26,3175	26,07843	26,08483	26,78818	26,09887	24,87235	25,29915
28,89357	28,32582	28,6976	29,45996	29,7921	29,70604	29,07184	30,20455	30,12842
32,37023	33,27378	33,19432	32,67712	32,71778	33,38317	33,51136	32,40586	32,37746
27,82457	22,22908	26,8411	28,13467	27,67101	27,37815	26,85958	27,07449	26,65567
22,98498	23,10019	24,09997	22,65718	23,60543	21,79398	23,99501	22,06209	23,02199
22,69794	22,9823	25,89069	26,70807	25,99596	21,42328	25,08148	26,87091	26,11504
22,44216	22,29515	21,8745	26,8918	27,93343	22,3351	23,1682	27,71803	27,88821

28,86847	25,22255	23,93872	28,67949	28,82271	28,34875	28,94827	29,42582	30,35174
27,409	29,02929	28,78812	26,86962	27,06589	28,87678	28,89235	26,98	26,92343
21,14364	22,45959	21,6066	23,11883	23,52409	21,33205	22,62649	23,46276	23,12591
28,06902	26,75175	22,9078	28,69942	28,62457	26,73854	27,21234	28,70547	28,86124
28,45591	27,97115	25,94675	30,13931	30,33543	29,29721	29,07702	30,74671	30,57362
28,08396	28,29772	27,10669	27,45772	27,76989	21,23318	27,06969	28,09018	28,31385
32,25367	31,42842	31,05543	32,6382	32,8189	30,53751	30,67152	33,03574	32,60869
21,83059	22,35942	23,95503	22,99011	22,52471	22,67241	22,55497	23,16306	23,2048
24,49363	25,39369	25,63425	25,80092	25,88462	26,39831	26,72988	23,14286	25,68035
28,25142	28,18698	28,13892	27,08172	26,78731	28,6101	28,57236	27,50385	27,46599
29,18348	29,40099	28,37952	29,30898	29,31954	29,29999	29,52232	30,40304	30,03486
22,35912	23,95871	21,64258	23,57819	22,99253	21,29182	21,36832	24,78731	24,35057
28,59399	28,64935	28,3438	28,34164	28,56107	28,7376	28,03249	28,91088	28,1472
23,26826	27,42005	26,16066	26,02789	26,17493	27,22894	27,662	25,26964	22,28789
27,09154	27,17693	23,85863	22,72165	27,13438	26,72078	26,47979	21,88856	25,99466
26,93348	25,91684	25,40669	24,97456	26,01767	26,10015	26,38017	26,146	25,63702
29,70532	30,88729	30,16854	28,60038	28,53267	30,07915	30,23421	28,67074	28,63737
29,74069	27,99857	28,44221	29,78396	29,52427	29,45252	29,43796	30,38915	30,22768
23,89384	24,98298	24,33845	23,1695	22,83973	24,19528	24,15267	22,20413	22,43987
27,79371	27,19094	27,70372	28,74622	29,1228	28,4729	28,49102	29,57782	29,8098
28,50881	27,51089	27,90828	28,44557	28,73841	28,99021	28,80557	28,76819	28,57578
27,09778	28,37061	27,34087	27,58606	28,19315	29,02324	28,3803	27,94921	28,35094
26,33748	23,30331	25,61288	27,87596	27,00689	25,76532	25,80087	26,28348	26,59548
23,76749	27,1333	26,10571	22,37297	24,27405	25,9091	26,6363	24,42316	24,67955
28,4394	29,1112	29,40734	28,72994	28,32444	28,37886	27,92914	26,89504	27,72286
27,38278	28,32813	27,62819	28,76294	27,96752	27,93703	27,80538	27,67324	28,35288
29,42906	29,21403	29,45862	29,81929	30,12657	29,93551	29,57002	29,90985	30,32152
29,70731	30,41084	27,82766	27,80655	27,55391	26,67101	28,73386	28,55757	28,7679
25,23356	26,33668	24,68886	24,4084	24,56209	26,16092	25,99401	23,71963	24,19764
28,70514	29,27173	29,10652	29,75592	29,60495	27,91085	29,09439	23,45103	29,32959
28,92337	29,32068	29,82935	28,1907	29,30967	28,75875	28,64375	28,84852	28,08639
28,39334	29,27344	27,98462	25,62171	25,95491	26,39537	27,39174	27,75786	27,45301
28,35368	27,61267	26,77128	27,12001	23,49497	27,9723	28,18461	28,13506	27,65928
26,15244	28,02205	27,40251	22,93626	21,8841	27,4373	28,38414	22,43506	21,28574
28,20592	29,57266	28,76135	28,06327	28,50116	28,72306	29,16674	27,72468	28,35069
37,42421	38,73302	38,14632	37,56347	37,76457	39,12461	39,19818	38,10963	38,10559
24,86815	25,55318	24,44979	26,60399	26,9215	27,41828	26,91969	25,86976	26,79687
30,00437	30,95768	31,37932	29,33731	29,04497	31,8546	31,86924	29,74962	29,76882
33,4021	33,25462	32,80859	33,53125	33,70419	33,37244	33,57678	33,78107	33,82231
22,39099	22,82568	23,34163	29,5508	30,38618	27,79123	22,99408	31,32254	31,28312
23,98237	23,15892	23,08046	28,98454	28,68949	22,54555	22,31535	29,65041	29,91613
25,87103	22,70381	27,51443	28,61765	29,86532	24,29221	26,28829	29,95421	28,29167

6MJE0_HU tr|A0A1D6ZNI8|A0A1D6ZNI8_HUMAN MHC class I antigen (Fragment) OS=Homo sapiens

22,87294	22,8583	23,72993	27,1518	23,52378	22,30649	21,8716	28,1249	28,01036
23,24184	25,25133	23,87793	22,94744	22,44208	24,57404	24,97657	23,29908	22,72538
24,52641	24,32745	22,21216	29,76836	29,95754	30,88103	31,05601	30,82533	29,67459
30,33947	28,09894	28,59385	30,34794	29,98726	29,70336	30,16591	29,10734	29,01404
28,38681	28,48585	28,70879	29,32706	30,13516	28,76816	28,2122	29,11647	28,88899
28,80649	28,7424	28,63395	28,24127	28,88405	28,61041	28,39844	28,49209	28,58223
33,24076	33,12498	33,48015	33,36071	33,09248	32,49522	32,63644	32,6707	32,82423

30,90821	30,49895	28,22628	29,54652	29,25636	29,20595	29,30136	30,01185	30,42352
27,32672	27,07816	27,07908	23,25214	21,07985	26,08558	22,13194	20,47863	22,74585
30,37223	31,0658	30,05453	30,20233	30,64341	30,85162	22,18038	30,42542	30,56047
32,99998	30,75756	31,38283	34,14186	33,95593	33,23891	32,67272	33,22777	33,01876
23,78987	22,73767	24,36073	22,48009	28,41708	23,01492	22,59114	29,18362	29,21988
27,43913	28,14739	24,51552	28,59602	28,23759	22,3943	27,99841	22,1329	27,55054
27,87079	28,04323	27,51668	27,61996	27,93326	27,3379	27,7082	29,23938	29,22041

LFQ intens	LFQ intens	Potential c	Student's T	Student's T	Peptides	Razor + un	Unique pe	Sequence c
3	3							
30,2014	30,51841	+			11	11	11	75,3
27,47568	32,87364	+			28	28	6	64,6
22,47785	30,51427	+	+	1_2	29	17	3	46,1
22,98594	25,6666	+			24	13	13	55
24,41796	24,93535	+			20	17	16	43,5
27,7845	34,07933	+			34	34	8	45,3
25,49537	22,65014	+			16	15	15	41,6
20,61423	30,23261	+			25	13	12	57,6
27,20592	22,63946				2	2	2	28,6
21,7265	20,35057				2	2	2	18,8
30,30171	30,06201				18	18	3	26,5
23,09436	24,24235				3	3	3	5,1
34,17042	33,84652				70	70	70	51,8
24,45632	25,07292				4	4	1	18,2
32,66465	32,65151				21	21	6	63,5
22,90845	23,64265				2	2	2	5,9
21,30471	22,27878				2	2	2	12,3
33,04968	32,51022				105	105	105	39,2
26,48228	27,71646				11	11	1	22,7
28,66848	29,82502				30	22	22	42,2
26,17122	25,20165				5	5	5	6,2
31,53765	30,2683				5	5	5	39,7
27,15528	27,30669				8	8	6	30,8
21,97597	22,60404				3	3	3	11,3
28,50199	28,15764				11	11	11	20,3
27,83786	29,16813				27	27	26	14,9
21,99211	27,43643				10	10	10	29,8
22,73737	24,74796				6	6	6	7,4
28,69604	29,52217				5	5	5	31,7
29,86137	27,6372				6	6	6	25,6
33,09179	33,19459				37	37	1	43,9
28,13501	22,22175				5	5	5	20,9
23,22935	26,05658				4	4	4	18,4
28,74945	29,10052				8	8	8	43
29,64069	30,2934				15	15	14	51,5
22,70791	24,7093				4	4	4	10,8
25,40788	25,35387				3	3	3	12,3
28,20741	25,89396				8	8	8	14,8
27,99145	27,2674		+	1_2	26	26	24	21,6
31,44618	31,4631				8	8	8	83,2
29,78757	29,64566				7	7	7	50,7
28,67182	28,42146				11	11	11	15,4
32,01475	31,72012				2	2	2	29,8
22,40927	24,00084				2	2	2	30,8
22,03864	25,182				4	4	4	11
28,0004	27,1547				3	3	3	17
27,53148	28,49377				16	16	16	24,1
31,20432	31,15551				15	15	7	46,7

23,92379	23,30522				110	2	0	59,9
22,62042	23,47148				1	1	1	6,3
39,64786	40,11942				83	83	1	86,4
27,70807	29,21914				9	9	9	29,7
31,4446	31,74186				16	16	16	42
29,4308	29,30269				12	12	10	34,2
23,66297	23,16938				3	3	3	58,2
26,87853	25,42037				7	7	7	29,2
26,64623	27,34528				6	6	6	28,4
27,07296	27,94229	+			25	19	14	37,6
29,44583	25,93578				9	9	9	37,3
27,75168	23,33238				3	3	3	20,6
34,82941	34,81855				27	27	25	47,5
28,28476	28,96832				4	4	4	39,8
28,11083	27,75443				11	11	11	39
32,45405	31,01618				10	10	10	39,6
24,05117	25,27188				6	6	6	15,6
24,60162	24,63229				3	3	3	37,4
26,54038	26,44435				7	7	7	10,9
27,71581	28,31799	+			10	10	10	14,5
26,22245	28,21919				4	4	4	44,7
31,98512	33,03262				4	4	4	45,9
30,21188	29,05245				18	18	18	51,6
24,57097	25,78114				7	7	7	15,4
28,89888	29,60247				6	6	6	27,9
29,71363	21,02804		+	1_2	6	6	3	21,1
30,15231	27,78332				12	12	12	40,7
28,83533	29,82773		+	1_2	11	10	10	39,3
30,02472	31,08278				17	17	17	39,8
27,06497	27,54687				9	9	9	30,1
29,83017	30,49486				22	22	22	65,2
30,98352	31,59513				18	18	18	43,9
22,2937	25,29489				5	4	4	16,9
30,88074	30,92825				9	9	9	82,1
29,10702	29,76517				12	12	12	42,5
32,33111	31,71759				51	51	51	36,5
36,31932	36,28361				72	72	1	67,9
28,00443	28,85103				14	14	6	24,7
32,10793	32,37233				22	22	22	54,9
29,07694	28,57261				12	12	12	10
26,96939	28,13036				10	10	10	7,7
23,22929	22,0688				8	7	6	16,5
27,87085	28,74871				9	9	9	36,2
25,1225	24,67659				3	3	3	25,9
27,05258	26,74098				5	5	5	45,8
30,39489	27,65663				6	6	6	60
31,25496	30,96279				13	13	13	49,5
24,57306	24,00873		+	1_2	5	5	5	15
26,72481	27,25836				5	5	5	61
24,13085	22,86091				2	2	2	9,1

23,92615	23,37663			9	6	4	16,8
28,17312	28,36102			7	7	7	57,7
29,11195	29,22637			28	28	28	20,2
26,89631	29,84933			3	3	3	32,4
31,51267	32,05937			26	26	26	45
32,76289	32,05148			57	57	57	48,3
29,70504	29,26924			15	15	12	28,5
28,65591	27,46544			8	8	8	11,1
30,71367	32,10659			31	31	23	45,9
27,3973	27,80489			8	8	8	17,3
28,72972	22,85296			2	2	2	10,4
25,12684	26,26031			9	9	9	15,2
28,37927	29,38264			15	15	15	23,4
27,25638	27,37219			4	4	4	25,6
22,38684	22,3639			4	4	3	10,7
27,63235	27,59278			4	4	4	36,9
28,08208	23,7703			1	1	1	8,2
28,57841	27,46669			15	15	15	18
27,10059	25,79726			7	7	7	16
28,42266	27,61982			14	13	4	23,6
22,04314	28,46303			4	4	4	15,5
21,42224	19,70838			2	2	2	26,9
26,05842	25,92295			3	3	3	6,1
28,12357	28,34177			7	7	7	24,2
25,89375	25,32711			6	6	6	35,2
30,21547	31,61986			5	5	5	6,6
26,86962	27,13985			14	14	14	17,7
27,85822	27,64877			6	6	6	51,3
25,46955	25,74981			8	8	8	9,4
26,56551	26,61526			5	5	5	26
33,19407	32,88736			18	18	18	65,3
24,02359	22,4964	+	1_2	2	2	2	21,4
26,76648	26,63948			4	4	4	20
26,27837	27,31769			25	24	24	15,3
30,8017	30,96237			8	8	8	53,4
28,90058	26,61583			5	5	5	17,7
25,07916	26,10211			3	3	3	29,8
26,33712	26,60964			7	7	7	13,5
28,69315	30,06625			5	5	2	31,8
27,77335	31,74053			5	5	3	70,8
30,15906	27,11862			5	5	5	23,6
30,52318	30,37389			13	13	4	30,9
28,05698	27,26785			15	15	15	19,5
29,78983	31,10905			7	7	7	33,2
27,1057	25,89901			7	7	7	38,2
23,78566	25,39061			58	1	0	42,8
27,3946	27,05765	+	1_2	13	13	13	5,9
21,65471	23,13136			5	5	5	21,4
23,61605	24,59007			4	4	4	26,8
23,14555	22,7834			2	2	2	2,7

28,86994	27,17207			11	11	11	19,1
31,98077	32,86765			15	15	15	56,2
22,87529	22,73595			4	4	4	14,1
21,57464	22,0971			2	2	2	12
32,67082	32,5158			21	21	21	46,5
34,00018	33,87317			7	7	7	46,9
25,26338	22,54016			4	4	4	18,5
27,91239	27,94665			12	12	12	25,8
26,32445	27,66452			4	4	4	24,1
28,50994	28,98076			11	11	9	25,8
28,68656	28,03889			5	5	5	6,9
30,83771	30,31084			5	5	5	32,7
21,06007	22,18177			2	2	2	17
27,67283	26,34415			10	4	4	43,4
31,81867	31,29975			16	16	13	59,3
29,81967	29,82792			5	5	5	41,9
24,82182	23,49875			5	5	5	4,1
30,07202	30,27487			16	16	16	39,6
22,25546	22,98968			1	1	1	4,9
27,52447	26,34316			21	18	18	24,7
27,55772	25,58446			23	23	23	8,7
29,45405	30,24762			32	32	32	21,5
24,94864	24,56889			3	3	1	9,6
27,70299	28,13775			10	10	10	52,9
28,78176	29,24681			7	7	7	6,7
25,67694	26,49496			5	5	5	20,7
23,256	22,54659			5	5	5	8,3
23,00335	21,34792			1	1	1	9,6
28,78924	27,59662			3	3	3	25,7
24,47914	25,04635			7	7	7	4,8
27,32002	22,81877			7	7	7	20,5
26,27046	26,74638			4	4	4	24,1
28,38718	28,44249			8	8	8	15,9
22,26167	24,22538			3	3	3	36,3
27,73596	27,54768			5	5	5	21
23,00949	22,52667	+	1_2	3	3	3	9,7
29,14936	26,91319			2	2	2	12,4
28,95333	26,63533			10	10	10	29
22,11832	21,33434			5	5	5	13,2
23,93957	25,17268			4	4	4	25,8
22,14252	20,9939			3	3	3	3
26,73195	26,98663			3	3	3	4,5
22,43952	22,41341			2	2	2	21,8
29,07181	30,04101			8	8	8	52,6
24,93472	26,07812			4	4	4	27,7
34,96456	35,41516			5	5	5	43,7
29,41357	29,77698			8	8	8	60,3
26,50303	27,1378			11	8	7	46,6
28,1601	27,92818			15	12	12	54,9
27,64678	27,53942			5	5	5	70,5

29,54481	31,90203			5	5	5	38,8
24,96899	21,43893			7	7	7	53,9
27,2516	28,17016			3	3	3	41,7
31,04785	30,74214			14	11	9	55,5
31,69387	30,24853			17	17	1	61,6
29,0922	28,78609			2	2	2	50
29,28701	29,04679			7	7	7	17,1
32,14659	31,15158			24	24	24	30,1
21,26774	22,32003			4	4	4	31,3
27,65055	23,1283			7	5	5	18,3
26,42686	26,03687			5	5	5	5,7
28,51273	28,56779			4	4	4	21,7
27,57795	29,18182			6	6	1	46,6
27,09225	28,22246			3	3	3	5,5
29,84894	30,19507			26	26	26	14,1
25,31316	25,29635			9	9	9	15,5
32,44586	31,76228			24	24	22	52,5
31,15672	31,89638			20	18	18	35,5
29,70914	30,1343			7	7	6	39,2
26,46579	27,45513			4	4	4	7,2
27,99394	28,1456			10	10	10	11,9
34,5739	34,16121	+	1_2	22	22	22	49,7
27,55223	26,41745			8	8	8	14,8
28,96058	28,41216			11	11	11	35,6
32,60213	32,57786			16	16	16	58,4
22,94758	26,84122			13	13	13	15,3
26,62324	25,73244			7	7	7	28,2
27,94559	28,32633			10	10	10	18,4
27,50945	21,8165			3	3	3	12,7
27,4753	21,95672			6	6	6	11,3
31,08087	31,5073			44	44	44	56,1
36,06305	35,62631			61	61	61	61,7
28,53118	27,55538			10	10	10	17,7
26,15883	27,18782			9	9	9	22,3
32,42542	33,33165			8	8	8	34,6
30,00945	29,20506			15	15	14	11,9
25,21656	26,79588			5	5	5	20,4
21,35252	22,27447			15	15	13	3,9
26,00071	26,82784			3	3	3	13,3
27,75983	26,20253			11	11	11	21,4
31,54209	30,68813			22	22	22	41,3
32,87197	32,17173			14	14	14	56,4
30,22159	30,5139			21	21	21	47,2
25,88159	25,53074			7	7	7	20,1
29,96472	30,12312			8	8	8	29,9
32,42114	32,80205			60	60	60	45,6
26,91547	26,26469			9	9	9	13,7
21,97225	24,02431			4	4	1	23,3
25,49308	26,83062			3	3	3	8,4
27,33151	21,88122			4	4	4	10,7

30,30726	29,43418			28	28	28	17,4
26,47011	27,54532			16	16	16	17,9
22,41431	22,69217			5	5	5	11,5
28,52671	28,23622			10	10	10	21,5
30,34742	29,24407			14	14	14	15,9
28,11709	26,58892			13	13	13	20,8
32,61489	30,55344			19	19	19	42,2
22,95408	22,2762			5	5	5	13,7
24,72858	26,61933			5	5	5	25,6
27,61253	28,66075			20	20	20	18,2
29,72728	30,19507			9	9	9	29,4
24,67632	24,27049			7	7	7	7,9
28,45254	29,0643			17	17	17	14,8
25,69238	26,59235			2	2	2	12,5
26,92411	23,56164			4	4	4	26,5
26,54432	25,27501			5	5	5	16,8
28,464	30,06381			46	46	46	21,2
30,00223	29,88217			16	16	16	28,4
22,24566	23,55415			3	3	3	34,1
28,83843	28,87059			7	7	7	37,1
28,83699	28,58405			11	11	11	36
28,34401	22,16493			4	4	4	74,2
26,7057	26,33868			8	8	8	10,1
22,79165	25,97412			9	9	9	5,5
27,25422	27,97208			18	18	18	27,5
28,57124	28,5634			9	9	9	9,7
30,01492	30,15846			10	10	1	43,1
27,71967	29,03572			4	4	4	15,2
23,18006	22,52028			4	4	4	17,4
24,1877	29,62633			10	10	10	7,1
29,00566	21,58528			5	5	5	25,6
26,76497	27,97405			7	7	7	39,7
27,5837	27,0662			10	10	10	16,5
22,62	27,64911			8	8	8	11,6
28,01845	28,46404			6	6	6	17,9
38,09501	37,49905			111	111	0	62,5
26,64072	25,56842	+	1_2	6	6	6	34,8
29,53185	31,83665			59	59	1	43,7
33,70913	33,52402			106	106	49	36,7
30,96747	22,67339			3	3	1	20,3
29,75681	27,0701			13	8	0	39,6
29,24266	26,092			14	14	0	56

OX=9606 GN=HLA-A PE=3 SV=1;tr|A0A0S4XRM0|A0A0S4XRM0_HUMAN MHC class I antigen OS=H

28,1209	25,79025			2	1	0	12,2
23,26055	23,22372			110	2	0	67
29,61569	29,87948	+	1_2	18	18	17	36,4
29,35323	29,58923			5	5	1	4,8
28,84999	29,16181			6	6	1	50
28,45846	28,02797			7	7	0	19,1
32,39971	32,94805			82	3	2	86,1

30,16651	29,49446	20	20	20	36,6
22,94437	23,02712	8	1	1	82,7
30,73651	31,21477	11	1	1	57,5
33,11114	32,84277	24	24	24	64,3
30,15279	22,7509	13	2	0	41,5
28,35001	28,34202	3	3	3	27
29,24386	28,63159	12	12	12	41

Unique + r	Unique sec	Mol. weigh	Q-value	Score	Intensity	MS/MS co	iBAQ 1-1	iBAQ 1-3
75,3	75,3	24,409	0	97,144	1,29E+10	56	72499000	1,6E+08
64,6	21,4	51,621	0	323,31	2,44E+10	173	1,75E+08	1,43E+08
31,7	6,7	58,536	0	132,71	9,64E+09	86	53050000	60895000
34,5	34,5	51,267	0	187,1	1,04E+09	26	589190	5860500
39,6	37,7	58,826	0	285,48	1,13E+09	36	7393700	413300
45,3	9,3	62,378	0	323,31	6,27E+10	186	4,88E+08	2,73E+08
40,4	40,4	62,129	0	323,31	1,21E+09	33	57599	1043400
37,7	35,4	48,105	0	292,46	3,9E+09	76	18414000	28835000
28,6	28,6	11,29	0	91,897	8,87E+08	19	0	100850
18,8	18,8	26,793	0	4,8618	17190000	16	64148	44043
26,5	4,4	82,577	0	195,5	1,26E+10	179	54165000	12210000
5,1	5,1	94,411	0	5,6156	61725000	10	111380	169530
51,8	51,8	214,84	0	323,31	1,21E+11	873	1,62E+08	41900000
18,2	5,7	32,316	0	18,095	3,54E+08	21	224290	69058
63,5	12,3	42,026	0	299,05	6,5E+10	215	4,69E+08	1,99E+08
5,9	5,9	57,896	0	5,1365	40824000	8	31181	10329
12,3	12,3	19,793	0	2,4636	52130000	8	452110	0
39,2	39,2	399,73	0	323,31	6,03E+10	783	50947000	19382000
22,7	1,5	58,397	0	50,188	1,25E+09	50	1198200	736720
33,2	33,2	104,85	0	182,3	3,99E+09	98	9785600	10522000
6,2	6,2	116,43	0	15,039	3,87E+08	18	871770	230690
39,7	39,7	13,89	0	101,98	1,68E+10	52	3,75E+08	1,23E+08
30,8	28,4	37,371	0	10,842	9,27E+08	27	8190000	9313400
11,3	11,3	47,984	0	2,6777	2,77E+09	7	23685000	17189000
20,3	20,3	74,823	0	15,573	1,83E+09	39	3527200	1954600
14,9	14,5	275,66	0	134,65	2,73E+09	111	2464100	2379800
29,8	29,8	41,174	0	13,75	1,99E+09	32	16969000	32253000
7,4	7,4	129,83	0	7,7814	3,1E+08	11	101600	915650
31,7	31,7	32,212	0	108,45	2,79E+09	30	31201000	18398000
25,6	25,6	28,671	0	92,323	4,49E+09	38	33758000	15949000
43,9	0,9	108,64	0	311,9	6,94E+10	333	2,32E+08	91592000
20,9	20,9	43,059	0	25,883	1,15E+09	14	25800000	0
18,4	18,4	30,497	0	31,254	6,3E+08	30	964530	11473000
43	43	24,96	0	187,45	4,25E+09	58	64130000	10672000
51,5	47,9	36,688	0	145,06	8,98E+09	81	89583000	18618000
10,8	10,8	41,326	0	4,3518	1,84E+08	10	126200	949770
12,3	12,3	41,428	0	9,2444	3,64E+08	10	1428400	864140
14,8	14,8	62,965	0	23,956	6,21E+08	21	0	0
21,6	20,5	187,15	0	220,13	1,57E+09	99	227840	107200
83,2	83,2	16,057	0	60,299	2,01E+10	126	3,41E+08	2,58E+08
50,7	50,7	15,799	0	135,39	9,29E+09	132	2,94E+08	1,11E+08
15,4	15,4	104,75	0	92,771	4,66E+09	75	14286000	13547000
29,8	29,8	10,16	0	89,507	5,61E+10	478	2,06E+09	40180000
30,8	30,8	11,765	0	10,41	66256000	18	1266400	2177600
11	11	53,224	0	6,1669	4,54E+08	15	5082400	3095100
17	17	25,331	0	251,01	1,1E+09	30	26111000	1344800
24,1	24,1	74,139	0	55,115	3,22E+09	79	35741000	5348700
46,7	15,5	36,154	0	269,99	1,65E+10	146	97461000	12038000

1,4	0	272,32	0	52,354	1,58E+08	36	81907	203620
6,3	6,3	38,797	0	15,898	31980000	11	288510	0
86,4	2,7	77,063	0	323,31	8,7E+12	4262	2,62E+10	2,22E+10
29,7	29,7	51,676	0	36,438	3,51E+09	51	15293000	6793500
42	42	53,981	0	323,31	6,47E+10	186	1,07E+09	1,67E+08
34,2	30,9	45,26	0	52,492	5,88E+09	63	32043000	18202000
58,2	58,2	11,139	0	13,186	1,18E+08	12	655760	8601400
29,2	29,2	38,714	0	13,819	6,39E+08	30	4519500	649710
28,4	28,4	30,768	0	62,195	1,06E+09	39	11794000	6004700
32,6	24,5	66,11	0	323,31	4,02E+09	57	10021000	6480000
37,3	37,3	27,87	0	38,935	2,62E+09	45	63316	23093000
20,6	20,6	18,552	0	10,841	1,58E+09	19	62550000	14557000
47,5	46,3	84,818	0	323,31	2,7E+11	562	9,95E+08	4,66E+08
39,8	39,8	10,834	0	13,423	3,12E+09	30	32816000	61618000
39	39	46,611	0	119,95	1,95E+09	76	3136400	373360
39,6	39,6	45,059	0	127,92	2,24E+10	127	2,23E+08	20886000
15,6	15,6	49,757	0	14,252	1,03E+08	14	0	319500
37,4	37,4	11,665	0	5,8479	1,88E+08	16	4794900	1871800
10,9	10,9	88,414	0	13,238	7,89E+08	24	4905700	1217500
14,5	14,5	85,696	0	32,006	1,57E+09	42	6595000	2566100
44,7	44,7	13,21	0	24,415	1,31E+09	48	10455000	24562000
45,9	45,9	9,681	0	50,598	4,66E+10	33	1,25E+09	8,32E+08
51,6	51,6	47,168	0	201,88	1,16E+10	125	1,5E+08	13146000
15,4	15,4	67,285	0	236,24	2,66E+08	39	889080	2386100
27,9	27,9	29,464	0	87,992	4,77E+09	67	94962000	39584000
21,1	12,1	28,955	0	12,282	3,05E+09	20	2358100	0
40,7	40,7	37,157	0	219,27	6,22E+09	74	12932000	69736
35,8	35,8	37,294	0	196,48	6,44E+09	90	1,13E+08	60047000
39,8	39,8	57,116	0	185,88	1,13E+10	159	65829000	24810000
30,1	30,1	44,552	0	30,727	1,61E+09	49	25298000	13145000
65,2	65,2	38,604	0	316,74	1,02E+10	128	92081000	17935000
43,9	43,9	58,14	0	218,1	1,81E+10	133	84055000	39637000
16,9	16,9	25,5	0	7,43	6,56E+08	24	16929000	2931000
82,1	82,1	15,054	0	86,434	1,37E+10	72	3,27E+08	61809000
42,5	42,5	37,821	0	195,99	8,29E+09	87	32655000	78236000
36,5	36,5	198,04	0	323,31	3,76E+10	518	70880000	25573000
67,9	2,2	129,38	0	323,31	7,85E+11	1688	1,87E+09	1,15E+09
24,7	13,1	83,263	0	65,326	2,62E+09	73	9861500	1149200
54,9	54,9	47,472	0	221,05	2,57E+10	201	56633000	29258000
10	10	167,55	0	175,05	3,33E+09	86	10641000	1712800
7,7	7,7	157,71	0	17,316	1,41E+09	56	1046700	500800
15	13,1	53,683	0	6,8444	1,57E+08	12	1028000	0
36,2	36,2	35,936	0	24,174	3,46E+09	64	22675000	15380000
25,9	25,9	19,48	0	14,533	1,6E+08	25	286380	1611900
45,8	45,8	11,301	0	50,679	9,18E+08	36	4136500	37048
60	60	14,716	0	91,859	5,69E+09	40	35649000	3951300
49,5	49,5	34,632	0	110,35	2,06E+10	148	2,45E+08	1,22E+08
15	15	57,519	0	7,4178	1,92E+08	16	0	0
61	61	147,33	0	9,749	7,98E+08	21	1586300	116090
9,1	9,1	30,992	0	2,8125	71957000	14	1216100	501650

12,6	7,6	70,108	0	24,943	1,03E+08	17	95421	130300
57,7	57,7	16,837	0	49,202	2,96E+09	42	13041000	35847000
20,2	20,2	211,68	0	110,05	4,69E+09	150	12781000	5521300
32,4	32,4	11,737	0	9,2184	2,13E+09	33	78400000	39687000
45	45	72,332	0	232,6	1,97E+10	192	81451000	57684000
48,3	48,3	174,01	0	323,31	4,34E+10	395	81565000	25649000
28,5	24,3	70,897	0	95,29	6,82E+09	84	58448000	3691400
11,1	11,1	108,34	0	106,17	1,71E+09	49	3170500	255080
45,9	36,7	103,06	0	246,79	2,14E+10	294	60622000	45274000
17,3	17,3	83,822	0	53,973	1,36E+09	74	7130600	2397500
10,4	10,4	25,673	0	46,705	2,25E+09	21	19435000	8106900
15,2	15,2	95,337	0	17,914	4,32E+08	31	1411300	452630
23,4	23,4	72,932	0	26,028	2,34E+09	43	7107400	7619200
25,6	25,6	18,108	0	3,2074	9,67E+08	8	31137000	2594300
10,7	8,6	70,289	0	49,458	1,44E+08	16	321640	435020
36,9	36,9	14,529	0	11,593	2,24E+09	36	48669000	23314000
8,2	8,2	15,523	0	7,339	8,77E+08	12	7983200	0
18	18	122,02	0	73,637	2,05E+09	74	2874900	72081
16	16	57,936	0	6,1243	6,01E+08	19	733900	0
22,1	7,7	92,339	0	47,732	2,33E+09	52	16121000	2518100
15,5	15,5	39,579	0	23,514	2,55E+09	7	33801000	165960
26,9	26,9	16,729	0	13,145	12704000	11	100530	0
6,1	6,1	65,568	0	4,645	1,59E+08	11	27737	28526
24,2	24,2	27,895	0	127,02	1,66E+09	84	17222000	5135500
35,2	35,2	20,195	0	36,023	2,77E+08	23	1245600	1639600
6,6	6,6	81,537	0	11,376	1,74E+10	43	72686000	63612000
17,7	17,7	106,78	0	51,324	1,47E+09	59	6993100	634840
51,3	51,3	20,838	0	120,62	2,01E+09	73	40160000	12528000
9,4	9,4	116,74	0	15,067	1,84E+08	21	7116,7	45561
26	26	28,804	0	45,273	6E+08	26	0	6306000
65,3	65,3	34,803	0	323,31	6,47E+10	239	6,73E+08	2,1E+08
21,4	21,4	12,269	0	4,7231	1,89E+08	9	0	0
20	20	23,787	0	40,708	6,22E+08	10	4803700	388220
15	15	277,5	0	47,411	1,13E+09	69	869610	274400
53,4	53,4	14,374	0	29,647	1E+10	38	95637000	32647000
17,7	17,7	41,654	0	53,085	1,64E+09	15	12191000	0
29,8	29,8	15,906	0	38,944	3,47E+08	25	3365700	2167200
13,5	13,5	86,551	0	43,375	7,7E+08	41	4116200	399030
31,8	6,4	30,137	0	19,122	4,56E+09	37	53133000	44254000
70,8	46,1	9,8875	0	57,927	9,4E+09	36	1,37E+08	5,28E+08
23,6	23,6	27,934	0	72,268	3,57E+09	35	77047000	360870
30,9	8,7	70,154	0	67,45	7,24E+09	83	33291000	12880000
19,5	19,5	119,09	0	76,539	1,35E+09	63	3356300	677960
33,2	33,2	22,742	0	40,946	9,11E+09	63	1,15E+08	48321000
38,2	38,2	22,728	0	84,366	8,11E+08	41	1048000	659540
1	0	240,85	0	8,211	1,33E+08	13	97468	160780
5,9	5,9	337,08	0	41,513	1,03E+09	34	237820	160850
21,4	21,4	27,647	0	6,5667	4,4E+08	13	182900	13376000
26,8	26,8	21,364	0	4,6214	75850000	12	285670	217270
2,7	2,7	81,996	0	4,2977	5,09E+08	7	1133600	804460

19,1	19,1	67,819	0	20,715	1,83E+09	28	673190	378960
56,2	56,2	46,918	0	154,86	3,41E+10	164	2,46E+08	1,94E+08
14,1	14,1	45,421	0	6,8391	2,13E+08	8	9112400	44635
12	12	26,425	0	6,001	47823000	6	1015500	284770
46,5	46,5	63,544	0	263,9	5,14E+10	181	3,42E+08	1,85E+08
46,9	46,9	11,117	0	49,284	1,11E+11	70	3,71E+09	2,29E+09
18,5	18,5	54,305	0	10,937	2,61E+08	8	1329200	457450
25,8	25,8	67,877	0	41,025	2,24E+09	49	10391000	13131000
24,1	24,1	21,057	0	7,7377	9,21E+08	21	15632000	7729400
25,8	21	54,102	0	37,765	2,89E+09	64	18071000	5014800
6,9	6,9	98,336	0	57,528	1,7E+09	18	2439600	936830
32,7	32,7	16,545	0	83,634	1,09E+10	41	1,97E+08	29201000
17	17	16,849	0	3,6318	22655000	11	1840300	106640
25,4	25,4	27,85	0	49,181	1,03E+09	25	3581700	677050
59,3	49,2	27,774	0	215,13	2,23E+10	164	1,68E+08	80143000
41,9	41,9	11,74	0	13,605	5,67E+09	49	1,68E+08	58536000
4,1	4,1	138,91	0	11,174	99376000	11	709760	0
39,6	39,6	61,68	0	48,744	7,68E+09	132	35864000	11411000
4,9	4,9	53,119	0,007813	1,5315	1,76E+10	10	87305000	1,11E+08
21,6	21,6	129,99	0	141,34	4,38E+09	75	12295000	1243100
8,7	8,7	314,77	0	58,895	2,49E+09	80	3768900	1025300
21,5	21,5	226,53	0	277,72	6,5E+09	134	8367300	11191000
9,6	4,5	42,1	0	23,912	3,13E+08	26	2549200	869580
52,9	52,9	21,086	0	46,253	1,65E+09	55	15973000	17529000
6,7	6,7	135,51	0	79,196	2,89E+09	58	9983300	823220
20,7	20,7	36,426	0	23,244	7,43E+08	26	11018000	8268400
8,3	8,3	107,54	0	9,187	50587000	16	75691	163590
9,6	9,6	29,295	0	13,229	36130000	13	194420	82660
25,7	25,7	19,365	0	17,92	1,25E+09	12	0	0
4,8	4,8	273,42	0	11,931	2,09E+08	14	370200	158520
20,5	20,5	46,943	0	62,298	1,45E+09	20	8382800	14420
24,1	24,1	23,115	0	7,4712	6,13E+08	21	2225700	934220
15,9	15,9	67,404	0	49,821	2,18E+09	33	1943300	9855400
36,3	36,3	21,517	0	13,334	80023000	20	383350	290690
21	21	36,923	0	31,222	1,28E+09	33	9219400	3546100
9,7	9,7	51,853	0	5,2514	3,99E+08	5	9752800	3496400
12,4	12,4	17,444	0	51,051	4,71E+09	38	3,59E+08	28579000
29	29	54,739	0	63,493	2,43E+09	78	5752400	150420
13,2	13,2	71,065	0	7,7924	36665000	13	55056	100110
25,8	25,8	20,7	0	11,647	2,9E+08	17	7189200	2780300
3	3	195,98	0	5,6753	24215000	19	14529	2561,9
4,5	4,5	73,786	0	5,2505	1,18E+09	11	8541600	2047500
21,8	21,8	17,963	0	22,148	32376000	13	358710	233820
52,6	52,6	22,871	0	233,96	6,11E+09	70	47126000	43673000
27,7	27,7	16,645	0	6,3143	3,04E+08	10	933470	744580
43,7	43,7	13,714	0	140,96	2,01E+11	100	2,73E+09	7,9E+08
60,3	60,3	16,584	0	157,16	5,94E+09	75	1,54E+08	91582000
40,5	36,8	28,302	0	93,449	9,49E+08	45	4728000	1208100
49	49	29,174	0	81,485	2,29E+09	60	23407000	7546000
70,5	70,5	5,0526	0	70,109	1,73E+09	26	2,51E+08	25921000

38,8	38,8	11,314	0	44,884	1,23E+10	45	5,87E+08	2,47E+08
53,9	53,9	18,012	0	26,594	1,78E+08	42	989130	664610
41,7	41,7	11,951	0	14,532	1,37E+09	28	6076500	18143000
49,4	45,3	27,745	0	106,42	1,49E+10	87	74083000	51614000
61,6	5,1	37,348	0	122,74	1,67E+10	148	95256000	42202000
50	50	5,0256	0	14,666	4,96E+09	9	3,79E+08	62006000
17,1	17,1	55,394	0	28,686	3,58E+09	38	14707000	10814000
30,1	30,1	133,8	0	279,35	2,58E+10	168	73337000	29399000
31,3	31,3	22,588	0	13,402	44157000	17	369740	405650
16	16	40,368	0	40,17	7,29E+08	18	1903000	277100
5,7	5,7	126,51	0	24,245	7,95E+08	20	2787600	188930
21,7	21,7	38,006	0	39,377	3,04E+09	49	23799000	7785700
46,6	13,2	19,202	0	84,558	3,47E+09	35	37246000	61871000
5,5	5,5	70,169	0	9,1631	8,52E+08	16	1516000	725840
14,1	14,1	295,22	0	323,31	7,67E+09	103	6780300	10068000
15,5	15,5	79,398	0	10,877	3,16E+08	11	2991600	274270
52,5	50,8	53,879	0	323,31	3,09E+10	218	1,29E+08	17027000
34,2	34,2	81,979	0	323,31	2,81E+10	245	1,37E+08	43015000
39,2	35,7	22,11	0	48,825	8,22E+09	59	46930000	57910000
7,2	7,2	96,792	0	74,085	1,2E+09	34	3371300	3494100
11,9	11,9	101,8	0	46,993	2,02E+09	27	4384600	2244400
49,7	49,7	65,33	0	323,31	1,23E+11	243	2E+08	59886000
14,8	14,8	60,793	0	53,174	9,6E+08	35	5597000	25649
35,6	35,6	46,231	0	107,1	4,97E+09	88	27149000	12938000
58,4	58,4	34,985	0	175,99	3,66E+10	166	1,12E+08	53009000
15,3	15,3	111,87	0	40,253	1,86E+09	33	9239900	5783600
28,2	28,2	43,078	0	21,772	1,42E+09	34	22682000	318850
18,4	18,4	84,364	0	71,859	1,91E+09	53	989460	1780200
12,7	12,7	36,728	0	2,525	3,23E+08	7	0	0
11,3	11,3	69,323	0	14,499	1,31E+09	16	10780000	1747700
56,1	56,1	129,57	0	323,31	2,13E+10	348	41793000	53764000
61,7	61,7	130,97	0	323,31	3,73E+11	747	9,16E+08	3,04E+08
17,7	17,7	97,539	0	105,58	1,73E+09	58	3312800	1348600
22,3	22,3	59,589	0	24,957	9,8E+08	49	6123200	2927000
34,6	34,6	26,264	0	210,73	6,09E+10	166	1,24E+09	3,29E+08
11,9	11,5	189,24	0	122,13	4,78E+09	82	6085800	1337000
20,4	20,4	47,837	0	23,371	5,8E+08	25	6615500	2035400
3,9	3,3	512,6	0	16,414	2,28E+08	24	145970	34076
13,3	13,3	26,697	0	4,324	8,07E+08	15	12477000	4058600
21,4	21,4	83,073	0	78,704	1,26E+09	62	440140	182870
41,3	41,3	74,68	0	323,31	3,07E+10	260	2,4E+08	1,12E+08
56,4	56,4	29,13	0	309,45	4,96E+10	175	3,44E+08	2,37E+08
47,2	47,2	60,673	0	221,31	9,75E+09	204	33085000	11616000
20,1	20,1	52,276	0	16,912	4,77E+08	33	3097400	2109200
29,9	29,9	40,876	0	39,226	6,46E+09	63	26180000	6088600
45,6	45,6	190,46	0	323,31	5,7E+10	534	63105000	70025000
13,7	13,7	110,11	0	57,344	1,24E+09	31	4179400	1404600
23,3	9,3	30,492	0	9,3771	37428000	6	520210	371950
8,4	8,4	37,106	0	3,1167	4,69E+08	15	1428400	0
10,7	10,7	43,235	0	7,9497	7,18E+08	19	0	0

17,4	17,4	250,53	0	117,47	4,52E+09	135	9374900	102170
17,9	17,9	159,69	0	141,77	1,87E+09	82	3631400	4628600
11,5	11,5	57,929	0	5,5525	49544000	11	61890	0
21,5	21,5	54,498	0	43,512	2,24E+09	50	6513900	610540
15,9	15,9	130,93	0	106,13	6,76E+09	78	13404000	2328500
20,8	20,8	100,16	0	110,82	1,7E+09	35	13206000	3207300
42,2	42,2	74,285	0	323,31	3,73E+10	210	2,28E+08	45913000
13,7	13,7	71,819	0	21,691	49831000	18	57178	43985
25,6	25,6	24,68	0	25,144	4E+08	41	2347200	5327500
18,2	18,2	165,27	0	139,2	2,04E+09	84	6033500	3353800
29,4	29,4	51,286	0	275,36	6,29E+09	60	36163000	13834000
7,9	7,9	144,37	0	15,203	91054000	25	40472	2135,8
14,8	14,8	134,55	0	37,994	3,02E+09	58	6407300	1396500
12,5	12,5	16,003	0	10,168	6,84E+08	16	13945000	14251000
26,5	26,5	19,891	0	1,9773	6,06E+08	7	14385000	10464000
16,8	16,8	47,071	0	40,784	6,02E+08	30	15576000	1127500
21,2	21,2	324,57	0	114,5	6,01E+09	170	7573900	8479800
28,4	28,4	85,206	0	183,26	6,39E+09	115	11127000	2828300
34,1	34,1	13,941	0	7,3546	98880000	17	457540	529600
37,1	37,1	23,031	0	11,535	3,44E+09	24	4439100	2042900
36	36	48,807	0	65,254	3,1E+09	74	7718800	1785000
74,2	74,2	10,438	0	14,13	2,16E+09	22	72547000	26657000
10,1	10,1	81,975	0	48,042	7,82E+08	23	2081800	55795
5,5	5,5	281,52	0	15,522	3,09E+08	20	131140	794250
27,5	27,5	83,432	0	92,302	2,46E+09	69	8607800	13751000
9,7	9,7	98,555	0	24,87	2,07E+09	43	859650	4680400
43,1	2,6	38,39	0	226,3	7,48E+09	127	29603000	7991300
15,2	15,2	33,868	0	70,212	3,58E+09	24	81727000	33833000
17,4	17,4	35,634	0	13,115	2,33E+08	21	4258900	6014000
7,1	7,1	142,34	0	72,862	4,15E+09	33	11716000	2358300
25,6	25,6	26,671	0	139,34	3,39E+09	29	21554000	25555000
39,7	39,7	24,341	0	52,488	1,61E+09	32	28141000	33562000
16,5	16,5	81,196	0	89,655	1,64E+09	33	20461000	2345900
11,6	11,6	94,75	0	31,508	7,01E+08	23	688780	5539500
17,9	17,9	32,227	0	5,4462	2,75E+09	22	10291000	41498000
62,5	0	259,21	0	323,31	2,36E+12	2794	2,17E+09	2,96E+09
34,8	34,8	24,093	0	11,912	6,03E+08	32	722330	1656600
43,7	0,5	240,91	0	323,31	1,33E+10	363	12119000	10214000
36,7	16,3	464,01	0	323,31	9,26E+10	944	64082000	28890000
20,3	11,6	14,813	0	41,996	6,83E+09	16	22583000	0
26,5	0	40,853	0	43,39	2,7E+09	34	0	0
56	0	31,468	0	94,489	2,75E+09	51	10619000	40222
omo sapiens OX=9606 GN=HLA-A PE=3 SV=1;tr|A0A0U5PUM0|A0A0U5PUM0_HUMAN MHC class I a								
6,1	0	21,216	0	6,2742	8,31E+08	8	3560700	839600
1,8	0	239,62	0	3,6111	1,24E+08	16	129590	219870
36,4	35	76,613	0	102,9	6,63E+09	108	572730	319880
4,8	1,2	99,978	0	22,836	6,46E+09	38	29561000	133310
50	9,6	17,471	0	26,583	4,16E+09	55	57192000	20338000
19,1	0	61,94	0	17,079	2,9E+09	37	21728000	24443000
4,7	1,9	74,831	0	323,31	6,66E+10	267	2,63E+08	1,09E+08

36,6	36,6	71,553	0	77,483	8E+09	128	71519000	22670000
20	20	7,99	0	16,266	6,66E+08	6	29338000	18754000
7,5	7,5	14,691	0	25,049	1,19E+10	30	2,77E+08	2,32E+08
64,3	64,3	46,924	0	323,31	7,22E+10	229	2,83E+08	20022000
7,7	0	38,865	0	26,998	1,99E+09	17	0	0
27	27	12,759	0	5,4929	1,72E+09	8	9018300	39553000
41	41	38,069	0	64,295	2,72E+09	54	13368000	2663600

iBAQ 1-5	iBAQ 2-1	iBAQ 2-2	iBAQ 2-3	iBAQ 2-4	iBAQ 3-1	iBAQ 3-2	iBAQ 3-3	iBAQ 3-4
1,82E+08	1,05E+08	1,32E+08	91384000	1,47E+08	60927000	48299000	59657000	1,09E+08
2,19E+08	40525000	34509000	46834000	41358000	6615700	3411300	7267300	1,24E+08
97959000	7555900	4620300	20505000	16035000	28207	21636	24610	71577000
27575000	210850	461720	8027,8	43448	0	0	0	1083900
31740000	916690	25936	172870	519910	258390	33832	1652600	318810
4,03E+08	65898000	50950000	1,09E+08	1,12E+08	11830000	4731800	3983700	5,01E+08
35007000	4229200	177750	23580	3145600	31924	4447,6	2518700	164010
49636000	4218700	3013200	5472900	3077900	103020	989190	26555	20837000
114900	111040	83312	21802000	25206000	29277000	29182000	20714000	88344
78521	64080	139190	143620	124760	89228	164760	177490	56133
29242000	39609000	32490000	17230000	26972000	24950000	20929000	14283000	8368400
0	230370	126850	164700	135830	159120	179900	151870	334010
52278000	1,62E+08	1,32E+08	81273000	82041000	2,05E+08	1,59E+08	1,5E+08	1,14E+08
15244000	0	7053000	710930	3327300	584480	3818100	535120	639020
2E+08	2,18E+08	1,91E+08	2,59E+08	2,69E+08	1,77E+08	1,89E+08	1,61E+08	1,68E+08
23838	646900	239000	8401,8	9439,1	223790	174350	122860	21909
402960	2153600	957970	499750	324580	135480	397720	275690	192390
13930000	36281000	28205000	20357000	22626000	51405000	40424000	34476000	16936000
7473700	6273400	6123300	4653000	5297700	7930100	3129100	5662800	5957500
13357000	10353000	4761500	3139500	1882700	4061700	2044300	3612200	8934000
0	1967000	1524400	0	4279,3	1223100	902590	1676000	13886
27551000	2,16E+08	1,35E+08	1,74E+08	1,39E+08	3,79E+08	3,46E+08	3,5E+08	1,36E+08
2033800	3624300	2735800	2599600	2297300	400020	8990700	4583100	6714600
39423000	14621000	14565000	7251600	14615000	0	9435800	0	12914000
639750	6368500	5430700	1430400	2615000	11815000	9264400	10239000	2180000
2381400	3688500	1138000	2197900	677430	1350200	391630	741190	1984800
18283000	4233900	650300	226370	3595400	58313	0	0	10344000
996690	600200	469310	588320	22679	631890	675290	767890	314280
10513000	204400	19545000	13218000	7775800	34127000	30843000	47751000	39640000
12031000	15576000	17121000	28386000	28861000	72395000	66779000	53269000	926910
1,05E+08	2,35E+08	2,02E+08	74989000	1,19E+08	2,89E+08	1,86E+08	1,94E+08	2,01E+08
287240	791970	1207000	0	37384	9170000	7950400	5776700	1162200
2702800	3771500	4837300	6209300	4407500	76335	521850	105240	4324600
29487000	44635000	35712000	14131000	16104000	63096000	31838000	35204000	41604000
50316000	49505000	39921000	33384000	39247000	45506000	45180000	35977000	51711000
1059000	832450	469850	1629300	1511600	147640	128010	32707	1121900
528490	1602400	1269100	1637300	1446200	1718100	1045700	1251000	1190500
0	9343,1	141610	9715,8	686790	5932600	4764000	7840100	24739
403000	2500100	1334000	1378500	1308900	3850300	1884600	2576400	658530
1,55E+08	1,92E+08	2,04E+08	99544000	77229000	4,11E+08	2,89E+08	2,88E+08	2,03E+08
1,11E+08	1,08E+08	1,19E+08	1,06E+08	82367000	81481000	99246000	1,37E+08	79296000
14854000	8103400	6415800	4846500	8272300	4474100	4081400	2620200	9955200
34340000	3,36E+09	4,73E+09	1,05E+08	3,91E+08	2,37E+09	3,45E+09	2,13E+09	54176000
3699900	167900	187620	730820	669660	783590	644580	639950	2283300
1324400	2133900	3518100	1255100	137430	0	2258900	0	97747
2116500	16270000	5176900	6898000	6057200	18663000	10432000	25804000	3824200
3519900	7383300	3734000	3580000	3516900	3812000	4163400	3820100	3919300
38363000	96996000	89981000	48083000	39776000	1,26E+08	98471000	67365000	36460000

215050	82966	118100	209690	252820	97615	131470	104580	68452
0	384920	256860	0	0	219380	246270	203040	0
1,64E+10	2,1E+10	2,18E+10	1,47E+10	1,69E+10	1,81E+10	1,47E+10	1,73E+10	2,28E+10
11786000	2062800	20092000	21291000	937280	15160000	13408000	15271000	30671000
1,59E+08	3,83E+08	5,26E+08	1,52E+08	1,34E+08	1,12E+08	1,32E+08	40595000	65369000
13949000	36794000	31254000	25819000	20322000	22993000	19090000	21822000	13156000
2872900	919430	412820	2399700	3166900	107420	157110	117220	291650
498610	5557800	3948700	1168700	1162100	4346800	3264600	5956900	859320
15815000	13400000	5847300	1085600	1082200	10154000	8089200	11397000	4013100
93590000	5586300	1520900	1372100	2987600	199960	0	2127200	5784700
54993000	18533000	9151300	10682000	10186000	15632000	14026000	43835000	1177000
3650900	35490000	20408000	8419600	8554800	14660000	15677000	12225000	1850700
6,06E+08	1,01E+09	8,81E+08	4,96E+08	5,35E+08	9,67E+08	8,24E+08	7,77E+08	6,33E+08
36601000	12336000	12853000	1,1E+08	43006000	17690000	8951600	24023000	29304000
4899900	37985000	38978000	131790	148490	4619400	5197000	12166000	6786300
17827000	72562000	87642000	6827700	4905300	2,53E+08	1,85E+08	1,89E+08	56359000
72864	310110	507650	342760	145580	816210	661340	722970	1255800
4854700	4967600	1954400	1952600	3393500	5390100	525350	3756900	4089900
1397300	4730900	2081000	989720	460010	1671600	1966200	2068000	1717600
1931500	3552300	1911800	3709800	4191900	5640800	2131000	5751900	8249900
22127000	6350800	7001900	14533000	15337000	22374000	11025000	6624900	22859000
4,36E+08	9,68E+08	7,57E+08	1,3E+09	9,24E+08	7,15E+08	4,39E+08	5,81E+08	1,12E+09
71416000	72631000	54177000	24272000	10427000	54317000	27292000	42971000	5229700
1024000	995490	393310	1554500	256150	141220	213180	318400	994590
29321000	47318000	30098000	36169000	21226000	23021000	9606500	28188000	37924000
0	20937000	13857000	32459000	26807000	26709000	25082000	22270000	9153000
5355200	56101000	32330000	890400	2406500	85524000	33565000	50027000	3390700
47984000	33719000	11325000	13995000	28474000	15008000	19226000	12486000	23964000
14943000	36456000	27635000	24211000	18630000	26005000	13553000	15377000	56741000
9474200	4466100	4969200	4706900	4571000	4657000	4688800	3378700	5604800
13821000	68859000	48922000	29689000	32317000	35765000	31455000	26640000	44134000
34779000	64872000	41657000	19132000	85064000	49624000	42816000	45368000	59032000
9935300	14082000	4720400	11029000	7304500	7058600	7131500	5887300	6701100
47917000	1,37E+08	84217000	1,63E+08	1,09E+08	1,79E+08	1,55E+08	1,34E+08	1,26E+08
41287000	34238000	22387000	56231000	97100000	37127000	26358000	3075000	7745900
26884000	51580000	46058000	26581000	32880000	80598000	61567000	61938000	23160000
1,09E+09	8,96E+08	1,03E+09	1,09E+09	1,17E+09	1,39E+09	8,77E+08	1,22E+09	8,8E+08
3996500	10678000	7514000	7969000	7600600	8394300	6119500	4601800	11384000
30019000	65485000	89792000	52195000	55961000	1,92E+08	1,2E+08	1,97E+08	1E+08
2335900	1838500	1734200	4253600	4157300	3882100	3745000	2287600	2609700
1110300	6232000	874710	530190	583580	1026300	6464700	478740	1867200
0	2524300	485520	134320	215520	44103	153150	32416	0
17152000	23998000	13087000	14632000	14478000	7597800	4544800	5377200	18418000
2166900	2997500	333290	1959700	1475600	1963800	2219100	2752000	2278200
643540	15559000	11083000	4065100	2373300	13966000	67739000	6657100	4849400
5049200	48052000	69258000	42977000	22012000	1,67E+08	79332000	1,52E+08	6511000
99425000	1,95E+08	2,17E+08	1,1E+08	55882000	1,5E+08	84413000	2,42E+08	65920000
0	1918000	1511600	552490	640380	1071300	1375000	792110	150430
903880	1206500	353830	152650	428070	988020	1181700	299400	2060300
224780	984650	100190	668190	437400	996490	2071600	1760900	32627

0	539650	165920	312850	164500	28977	82198	1649000	56252
49852000	55434000	37191000	16565000	16506000	30389000	31779000	19848000	22718000
1886200	3050900	3429400	2659200	2091000	4235900	3263200	4408800	3995900
30113000	46096000	44319000	2552800	34726000	21436000	687200	1394500	5528800
15875000	59735000	43419000	42222000	43375000	76475000	68665000	65412000	1,03E+08
24177000	48974000	51668000	42281000	45663000	74211000	75251000	77066000	39901000
3131100	14883000	17426000	16066000	11594000	36215000	7593400	19921000	17679000
398550	1362900	584540	2554100	1024400	5081000	9786300	6024300	2069500
47336000	43580000	30528000	31958000	20996000	25448000	27765000	10688000	67320000
4628100	4498000	7374400	3586200	5312300	5616300	5550400	5534600	7339800
4885900	23180000	4513400	24004000	17549000	64303000	44772000	33758000	5754600
2615000	991100	408750	359690	243830	345670	175420	790890	1808100
3975000	8148100	3356300	1891400	3577800	3188300	7263400	6039700	9536400
5614500	4036800	13823000	13295000	1016900	31728000	25435000	4436300	4996400
509920	1673100	350350	253220	301970	27477	10296	0	0
47341000	59775000	20268000	45713000	16222000	54921000	56264000	44206000	31483000
0	26785000	8278600	0	0	20994000	35778000	25478000	0
564620	2742100	951150	1184800	1042700	14862000	8506800	7509700	2337300
64855	1795100	4334600	229810	107300	3928000	4247600	2324300	432860
2354200	6287000	8251300	2771200	2881600	4306200	4876000	4217800	3785900
15350000	23824000	22523000	15958000	0	0	26174000	22580000	21862000
0	235270	204010	117010	93420	221170	313830	216280	86530
15951	699830	50694	543750	221810	57689	31837	837520	2973200
19608000	24480000	21652000	2876400	3734300	31635000	9583700	15300000	33276000
232680	886330	483090	178550	1638200	11482000	1409300	9706600	1869100
64845000	55280000	29663000	58580000	69350000	54209000	68709000	40992000	68165000
5683200	6872300	1801300	458650	1399800	1234300	1101400	2277800	2742400
19174000	32335000	18562000	25684000	21937000	20368000	22750000	20584000	17590000
245000	440740	95521	10158	68303	26037	11112	362620	1138400
5628800	2703100	2577600	5474900	3243100	9301700	2998400	5052100	2902200
1,97E+08	2,47E+08	1,82E+08	2,31E+08	2,42E+08	5,8E+08	4,18E+08	3,61E+08	2,5E+08
1063800	9016000	7426100	3955500	7096900	2074800	517490	0	362010
434000	12303000	23079000	3843400	4492300	11940000	431740	3274900	4103200
1250000	726740	334760	1807200	721410	794420	429520	106020	1253900
29674000	80375000	1,16E+08	42079000	81811000	1,9E+08	2,94E+08	3,28E+08	1,43E+08
13047	422500	51700	4837,5	34500	36906000	20182000	26490000	201000
3614400	7568800	5390300	2761500	3545000	4585800	9450600	5826200	9483400
2465300	6076100	3567000	955480	778580	1794700	2394200	1778600	1349800
4611800	25697000	7099600	1403200	30219000	26981000	11932000	3735300	31170000
1,33E+08	44361000	89486000	56402000	46069000	37914000	5106200	25516000	2,4E+08
2865700	6671000	973290	1618900	6551600	44378000	92984000	83365000	7422900
4528100	11167000	17090000	19611000	10555000	45536000	30795000	35279000	28766000
1863300	2148400	11368000	463350	294690	859240	207440	627230	552630
54653000	97647000	59853000	45166000	48324000	72823000	66026000	51302000	1E+08
711780	14984000	2980100	8875500	9888600	7429900	5712700	6512200	3582000
131200	26045	20893	155490	137210	92771	97682	80599	283420
114580	511800	515550	841040	596960	1513900	1613200	382330	370260
4186700	8284600	3395700	430240	2567300	288600	176800	0	955330
134240	84157	384880	267430	167620	78571	40390	1410100	3825100
0	2944800	2909900	600990	2468800	984840	2354600	0	1706800

850390	2653000	4397100	12391000	5459600	13679000	8532700	5094100	1362200
89312000	1,75E+08	1,39E+08	1,23E+08	1,2E+08	1,17E+08	97577000	1,15E+08	2,06E+08
17609	54107	1699900	897600	19699	6072,3	0	0	0
865890	939080	67069	33608	92605	237110	204290	169660	437960
1,04E+08	2,04E+08	2,28E+08	2,67E+08	1,77E+08	2,61E+08	1,31E+08	2,83E+08	1,52E+08
1,64E+09	3,16E+09	1,83E+09	3,26E+09	1,94E+09	3,29E+09	1,38E+09	2,63E+09	2,7E+09
585070	1175000	925850	603810	67625	3331200	2385300	2152100	54472
7034500	12217000	2674500	8963800	9819600	5250600	2834400	8120600	8996500
7730500	9172200	5510200	7147000	5013000	6152000	5342100	7172500	15451000
5143900	12384000	5910400	6025400	5157600	13138000	9538500	8454600	14261000
4478500	3657700	3046000	457790	1572500	6894300	14098000	7553800	3517800
50739000	2,22E+08	1,68E+08	52830000	38762000	2,59E+08	2,28E+08	1,97E+08	1,12E+08
549670	239930	730550	380080	375020	165840	173360	187520	914720
6440000	2250300	8533100	17587000	12336000	2962000	14845000	2730300	1560900
1,01E+08	1,54E+08	1,76E+08	68020000	1,05E+08	1,41E+08	1,21E+08	1,93E+08	85416000
41910000	99454000	1,08E+08	90809000	1,01E+08	1,14E+08	1,28E+08	1,21E+08	1,04E+08
0	0	0	0	0	919880	91628	84405	69351
11069000	21667000	30476000	19059000	16835000	37319000	25814000	31706000	33210000
1,34E+08	2,39E+08	1,61E+08	71774000	45707000	84781000	39352000	80158000	46462000
1423900	19339000	18337000	16780000	23172000	911920	1128200	1135700	1642200
1112100	1382700	682400	2302300	2577100	1542200	3030300	876070	123680
2997300	6878200	5493100	7455200	8357000	2349300	2498300	3578300	3349100
419210	1406200	748630	5639800	955750	214840	430000	552740	456350
1609100	2429400	1953900	32063000	31407000	14608000	9713000	5981700	4378200
1948900	1929500	1741400	879280	1289500	8670700	8390800	7596200	6608100
3462600	4445400	3489800	3876100	5970400	2786800	1432100	116750	4681100
143140	149220	273720	18487	93938	9996,6	109620	10666	28236
90297	261310	239340	336910	229650	240560	306520	317480	109540
105440	34258000	9122300	0	0	31512000	25213000	40969000	14786000
101120	136900	106230	69655	69513	172580	36870	260470	190810
181910	1416100	3324200	3688600	3941100	18496000	4072000	10159000	0
1706300	1617500	1001600	1201400	122270	1171500	1065600	9231700	30839000
15978	10850000	7294600	3097500	4566800	8477300	3311600	11317000	12047000
2188600	1023200	865370	948010	1120500	546660	680510	624150	1331900
10790000	2615500	12031000	409580	9383900	19149000	1070800	9065700	7795300
3898700	426320	0	239380	520440	662200	0	0	0
25231000	1,21E+08	83370000	16603000	17858000	1,4E+08	64703000	77697000	8362400
829560	31504000	14057000	3685700	2937800	18960000	17700000	18595000	1688900
39489	117180	160360	180700	115960	96832	63601	75511	42765
4583300	1771200	708920	423580	135310	1041400	2859000	1657300	5877900
0	33527	51927	17977	26782	29206	19278	49526	15066
2108400	3685500	1731300	1011400	2108400	6948300	2919600	3027700	3947300
171190	846360	958140	437690	461990	755570	834130	690680	726870
29990000	34102000	26451000	39245000	36520000	32588000	23544000	39796000	54007000
1439800	3694900	3112200	343170	380430	126050	195770	2566000	14129000
8,16E+08	4,44E+09	3,85E+09	3E+09	3,04E+09	3,67E+09	3,43E+09	3,97E+09	3,71E+09
51006000	46953000	35582000	27638000	29204000	29268000	35476000	45700000	47358000
1738300	20937000	1656400	12164000	1293400	471300	2559900	9277100	3266000
8671100	15272000	14231000	21665000	8360200	5621200	6073100	15873000	8153000
17469000	78262000	16449000	1,11E+08	94139000	61383000	57606000	89905000	60342000

2,74E+08	80414000	63561000	1,24E+08	1,64E+08	1,21E+08	2390300	40798000	3,53E+08
4368600	2286800	493440	2026700	1070600	1865300	1526300	2359100	182630
20475000	40651000	82397000	45655000	48278000	2749100	33811000	54151000	1,06E+08
64358000	1,26E+08	1,05E+08	1,03E+08	1E+08	1,47E+08	1,21E+08	1,1E+08	59192000
53292000	1,23E+08	76969000	68361000	56861000	95065000	1,11E+08	1,3E+08	27577000
2,66E+08	3,27E+08	3,29E+08	2,01E+08	1,52E+08	3,37E+08	1,21E+08	1,86E+08	1,22E+08
11216000	10729000	6854500	7890300	8859200	22486000	31864000	9694800	7987600
24071000	36136000	29285000	19162000	37122000	82755000	84533000	73413000	26313000
343140	951700	842810	444170	405350	250270	232330	198010	463180
0	6970200	1768200	1349200	785500	13768000	12798000	3233600	0
331050	1003900	1501600	1061100	1817800	3108300	983480	2412300	1370700
12470000	16906000	9237500	11047000	10283000	28313000	19470000	11638000	9270000
38834000	24155000	14584000	58162000	27051000	14253000	23726000	14500000	32792000
1262900	4497700	3815300	1429400	2603200	4132900	953940	4290500	3173600
10039000	1208500	963310	2047900	1612300	4468700	3812100	3891800	3642700
229220	55399	509900	186320	214500	1593000	365360	535810	386630
32548000	1,44E+08	1,37E+08	29788000	30909000	1,87E+08	1,77E+08	1,51E+08	71265000
73951000	1,26E+08	93276000	58259000	49557000	77373000	52941000	56885000	58555000
41721000	22917000	55426000	50588000	51872000	66047000	52326000	54662000	47693000
6041600	1539200	1123000	4216800	2592600	3228500	1910400	957120	2338700
3359300	8412800	5438900	6098600	4135700	4105700	5691200	4894700	2899900
37044000	6,17E+08	6,56E+08	3,42E+08	3,78E+08	8,27E+08	5,07E+08	6,61E+08	4,61E+08
12228	3302700	2706200	154290	101840	9393100	3185000	6156400	348900
4480700	35552000	46108000	43277000	22996000	22419000	18940000	21083000	6627200
89459000	2,27E+08	1,72E+08	80458000	1,9E+08	2,73E+08	77437000	2,05E+08	1,84E+08
10602000	682660	107950	3928300	1997000	157980	575110	13801	2641500
1647400	15260000	10844000	815300	6598700	553060	6074900	7715300	6578000
1436400	9821700	3074100	3137100	4096500	9289600	5015100	7267100	706370
0	0	12259	0	0	9337300	8358200	208090	46366
483740	7300200	6309000	0	0	8564500	4318000	4088100	0
22271000	22491000	15471000	50178000	46881000	24443000	19879000	19544000	22028000
1,39E+08	5,7E+08	5,05E+08	2,63E+08	2,72E+08	9,2E+08	8,35E+08	7,71E+08	5,14E+08
1596000	10125000	7702300	1641200	446320	5853600	5587800	3250500	1408100
2978200	3826700	3431600	1643600	1541200	2518900	1878200	447200	6480900
4,76E+08	8,1E+08	6,21E+08	2,57E+08	3,1E+08	8,77E+08	5,27E+08	5,37E+08	7,81E+08
1267800	2882900	6371400	4784800	4056400	15235000	4018500	21550000	2641200
1868100	4454000	2506500	2349600	3137800	3103200	1558800	1506700	5009900
90036	15394	329530	6993,4	4909,4	135270	54222	0	0
5629400	10354000	6730300	3115900	3156800	4972800	3287100	3246900	5035400
2008500	9008000	2518100	1584600	1880700	7710800	6280400	6192800	2710700
79332000	38383000	35204000	1,24E+08	93233000	64160000	79940000	1,1E+08	12496000
2,23E+08	3,06E+08	4,34E+08	2,56E+08	2,26E+08	3,54E+08	5,09E+08	3,99E+08	2,56E+08
17027000	50769000	43176000	10246000	12801000	34611000	22162000	38012000	41163000
1002300	2291100	298270	4330600	1230900	1098200	31634	2128700	45655
8828800	18841000	39162000	31162000	18598000	70020000	52721000	78125000	53814000
64480000	61455000	47967000	81579000	79961000	39820000	31420000	45717000	47305000
796200	11279000	1882200	2097100	2292300	1681000	1691800	1994000	2372100
627300	0	259280	0	106910	199590	134440	0	119610
606330	1428800	705030	385330	366140	6432200	5349700	7451000	1874600
1145700	3091000	7271200	23391	24282	5657100	14586000	4065100	33088

11247	2089000	4025700	2553100	389920	8701000	8097200	8049000	10453000
4508100	1681400	1779200	4359700	4883800	125270	1068700	781010	1739900
169680	356090	276600	30010	112440	333970	228640	180750	155470
666160	11817000	12244000	473290	507440	17952000	11642000	17567000	9736500
594070	19758000	19612000	11722000	4948700	14865000	27437000	21406000	10862000
2757500	3006000	1305800	131070	1043500	10562000	6756600	9589000	15967
46266000	1,76E+08	1,62E+08	38799000	44761000	1,91E+08	1,43E+08	77791000	12925000
139740	117150	68191	63067	54501	139180	112320	239270	431040
1388700	4070700	1956000	2171900	4670600	129090	1783300	217560	6742400
2400400	3246700	880040	2253100	2151800	2390600	1228800	1277400	2739700
8268700	40633000	37509000	16472000	19337000	50362000	39359000	30740000	21783000
21210	126400	78488	13311	4421,6	397330	213590	275180	296080
2275500	2467700	1610100	1462700	1821500	11595000	6207100	10618000	3689400
7526900	10733000	8014900	14101000	17800000	4814100	2890200	6051100	13808000
513650	931210	897700	6536200	7923900	400690	4095300	496770	3853400
1255100	1123100	1425600	1134700	658420	4209400	2148600	2700100	341170
4103600	1020700	491180	6317600	5408000	1910900	1219800	1699900	3490100
4598400	18907000	12056000	11895000	10116000	32147000	25670000	21787000	12655000
380930	394450	233370	333960	298120	154210	2507900	5769400	1300500
8304200	29925000	25528000	23943000	6387100	53006000	50748000	40329000	20034000
6558500	22155000	28806000	5749200	8895100	22967000	18273000	24302000	7720700
34223000	53591000	84912000	49173000	51800000	709250	56249000	73459000	36044000
407710	11692000	2004100	601500	533500	1029100	894900	1298100	1116900
180960	5026,6	71172	447070	489760	97372	93446	3427,8	325230
5932800	8989800	4447200	8801900	5473300	4462300	4106700	282780	1566800
2005400	3735400	3343100	4371100	2649200	1479700	2865200	8327400	6255300
18015000	42766000	51836000	25624000	15732000	49831000	47011000	44505000	41083000
29774000	19025000	14395000	11715000	20917000	29962000	29046000	32674000	22030000
2969700	211360	210550	320570	293730	95702	57033	50182	59157
3991300	19748000	8706300	3972100	3031300	555610	12290000	872070	8137400
25922000	16702000	78997000	40511000	42579000	848060	2288400	83522000	255490
7578700	2440000	4405000	5696500	4542000	13366000	7409900	7991700	8534100
1814000	3075400	199930	6636400	4079000	5406000	3141300	2235200	242170
1581800	0	122590	458910	9392300	0	0	0	2258000
9785800	13476000	17330000	15479000	20954000	4618500	13932000	12040000	12251000
1,03E+09	1,52E+09	1,4E+09	3,32E+09	3,3E+09	2,41E+09	1,93E+09	2,37E+09	1,15E+09
2198700	9511400	9987700	12240000	5983300	440490	4866300	4471200	2736900
6838400	2331300	2585400	22674000	23935000	7904300	7630300	4619600	25856000
19128000	57285000	51440000	34573000	34508000	67250000	62687000	56468000	32542000
17176000	2,09E+08	3,59E+08	33559000	14240	4,98E+08	8,76E+08	2,59E+08	1914200
0	22733000	25546000	16728	19658	31133000	41316000	29162000	229830
63748	22608000	38373000	133460	1171200	44600000	35582000	18577000	375850
ntigen OS=Hom								
831260	10998000	2704600	1052500	0	23233000	16291000	17599000	5953100
87889	73687	65350	251030	281800	45721	51231	38103	60169
32887	11261000	14385000	31679000	46276000	30816000	12062000	21324000	10422000
171330	17590000	16631000	12359000	19762000	13544000	7727300	6778300	7545500
15246000	58785000	96481000	42391000	17423000	99906000	79547000	82599000	24076000
1871000	9836600	6318200	5023200	4078400	3430000	9821800	17080000	91666
2,21E+08	2,64E+08	1,87E+08	77783000	96763000	1,29E+08	1,09E+08	70512000	1,38E+08

887020	19300000	10887000	4722400	8407000	19072000	28634000	32527000	10037000
17654000	34429000	9474100	6613300	4293100	27189	5624200	2548800	4417600
1,43E+08	2,12E+08	2,47E+08	2,1E+08	1,1E+08	2,03E+08	2,09E+08	2,3E+08	3,12E+08
46364000	6,46E+08	4,47E+08	2,12E+08	1,39E+08	3,49E+08	3,66E+08	3,06E+08	1,93E+08
0	1844100	7713300	0	670990	29065000	22905000	45292000	3167000
14813000	68825000	27235000	5365200	26930000	7431400	23949000	42821000	20424000
3127200	10215000	6946400	1788900	3031000	21819000	14093000	21828000	9978000

Student's T	Student's T	Student's T	Student's T	Majority pr	Fasta headers
0,933574	0,957416	-0,04553	-0,07348	CON__P00761	
0,009543	0,105333	1,522588	3,217542	CON__P02	;sp|P02533|K1C14_HUMAN Keratin, type I c
0,000678	0,0104	3,195041	6,05605	tr|B4DRU6	tr|B4DRU6|B4DRU6_HUMAN cDNA FLJ5465
0,030138	0,144	4,794743	2,822366	sp|P08779	sp|P08779|K1C16_HUMAN Keratin, type I cy
0,314473	0,464058	2,098201	1,061241	sp|P13645	sp|P13645|K1C10_HUMAN Keratin, type I cy
0,004429	0,093143	1,841355	3,877429	CON__P13	;sp|P13647|K2C5_HUMAN Keratin, type II cy
0,200843	0,342776	2,811212	1,399306	CON__P35	;sp|P35527|K1C9_HUMAN Keratin, type I cy
0,011495	0,103048	2,795826	3,416882	sp|Q04695	sp|Q04695|K1C17_HUMAN Keratin, type I c
0,161013	0,309	-2,40899	-1,53933	tr|A0M8Q	tr|A0M8Q9|A0M8Q9_HUMAN C1 segment
0,006829	0,090824	2,325798	3,721142	tr|A0A1U9	tr|A0A1U9X8Y4|A0A1U9X8Y4_HUMAN Chlo
0,173662	0,503744	-0,23077	-0,93989	sp|O00391	sp|O00391|QSOX1_HUMAN Sulfhydryl oxida
0,078788	0,285622	-0,71979	-1,6868	tr|E9PFW2	tr|E9PFW2|E9PFW2_HUMAN Beta-mannosi
0,025391	0,211709	-0,57956	-2,04062	sp|O00468	sp|O00468-6|AGRIN_HUMAN Isoform 6 of A
0,668577	0,798773	-0,4956	-0,41625	sp|O00560	sp|O00560-2|SDCB1_HUMAN Isoform 2 of S
0,69827	0,839165	0,12475	0,309004	tr|Q6FI18|	tr|Q6FI18|Q6FI18_HUMAN CYR61 protein C
0,558562	0,750911	0,242966	0,497979	tr|A0AV88	tr|A0AV88|A0AV88_HUMAN Disintegrin and
0,652324	0,787713	0,438661	0,431635	tr|Q53HL1	tr|Q53HL1|Q53HL1_HUMAN Myosin regula
0,268442	0,642141	-0,15329	-0,68689	sp|O15230	sp|O15230|LAMA5_HUMAN Laminin subun
0,210576	0,407439	-0,89296	-1,23682	sp|O43278	sp|O43278|SPIT1_HUMAN Kunitz-type prot
0,17361	0,409025	0,484562	1,194869	tr|A0A0S2	tr|A0A0S2Z3G9|A0A0S2Z3G9_HUMAN Actin
0,437598	0,606736	-0,84464	-0,76664	sp|O60245	sp|O60245-2|PCDH7_HUMAN Isoform B of
0,603494	0,761019	-0,35772	-0,47965	sp|O60814	sp|O60814|H2B1K_HUMAN Histone H2B typ
0,022633	0,136308	3,455642	2,972709	tr|B2R717	tr|B2R717|B2R717_HUMAN cDNA, FLJ9323
0,395929	0,533635	-2,37537	-0,89324	tr|B4DY05	tr|B4DY05|B4DY05_HUMAN cDNA FLJ5830
0,252272	0,440925	-0,72044	-1,09683	sp|O75326	sp|O75326|SEM7A_HUMAN Semaphorin-7A
0,286866	0,563195	-0,27714	-0,83334	sp|O75369	sp|O75369-2|FLNB_HUMAN Isoform 2 of Fil
0,015839	0,12336	2,214388	3,083337	sp|O75635	sp|O75635-2|SPB7_HUMAN Isoform 2 of Se
0,109853	0,271059	2,006785	1,770272	tr|B4DZ36	tr|B4DZ36|B4DZ36_HUMAN cDNA FLJ58441
0,24327	0,428812	0,703169	1,113125	tr|B3KNF2	tr|B3KNF2|B3KNF2_HUMAN cDNA FLJ14484
0,87823	0,916112	-0,05925	-0,12674	tr|I1W660	tr|I1W660|I1W660_HUMAN Dickkopf-like p
0,345758	0,628612	-0,22324	-0,70973	sp|O94985	sp|O94985-2|CSTN1_HUMAN Isoform 2 of C
0,48912	0,630242	1,533889	0,711588	tr|B3KQQ9	tr|B3KQQ9|B3KQQ9_HUMAN cDNA PSEC00
0,382896	0,563835	0,629514	0,830092	tr|Q5JPJ9|	tr|Q5JPJ9|Q5JPJ9_HUMAN Uncharacterized
0,109494	0,322826	0,59915	1,467828	sp|O95633	sp|O95633-2|FSTL3_HUMAN Isoform 2 of Fo
0,197558	0,491528	0,299145	0,992576	tr|V9HWB	tr|V9HWB9|V9HWB9_HUMAN L-lactate deh
0,349426	0,501792	-1,13862	-0,94615	tr|B3KUZ8	tr|B3KUZ8|B3KUZ8_HUMAN Aspartate amin
0,122994	0,2999	-1,11396	-1,58909	sp|P00558	sp|P00558-2|PGK1_HUMAN Isoform 2 of Ph
0,097141	0,252862	-1,85161	-1,83576	tr|B8ZX62	tr|B8ZX62|B8ZX62_HUMAN Plasminogen ac
0,000478	0,008667	-2,43379	-6,05135	tr|V9HWA	tr|V9HWA9|V9HWA9_HUMAN C3-beta-c OS
0,084467	0,297632	0,680743	1,632746	tr|Q5H9A7	tr|Q5H9A7|Q5H9A7_HUMAN Metalloprotei
0,084009	0,305929	0,537806	1,537097	tr|A0A0K0	tr|A0A0K0K1J1|A0A0K0K1J1_HUMAN Epidid
0,502913	0,704776	0,307134	0,58426	tr|H0YMD	tr|H0YMD1|H0YMD1_HUMAN Low-density
0,835554	0,899898	-0,09239	-0,17682	tr|F6MZK5	tr|F6MZK5|F6MZK5_HUMAN Insulin (Fragm
0,28521	0,479636	0,643329	1,008721	sp|P01834	sp|P01834|IGKC_HUMAN Immunoglobulin k
0,228147	0,488257	0,39063	1,015886	tr|Q8NCL6	tr|Q8NCL6|Q8NCL6_HUMAN cDNA FLJ9017
0,204807	0,355412	-1,83905	-1,35035	tr|A9LSU1	tr|A9LSU1|A9LSU1_HUMAN Type IV collage
0,198649	0,411044	0,751528	1,237309	sp|P02545	sp|P02545|LMNA_HUMAN Prelamin-A/C OS
0,03037	0,355379	-0,24399	-1,344	tr|A0A0S2	tr|A0A0S2Z3D5|A0A0S2Z3D5_HUMAN Apol

0,955059	0,975041	0,046409	0,052531	sp|P02751	sp|P02751|FINC_HUMAN Fibronectin OS=H
0,371276	0,544845	0,803837	0,874955	tr|D6RBJ7	tr|D6RBJ7|D6RBJ7_HUMAN Gc-globulin OS=
0,226825	0,546765	0,235458	0,869067	sp|P02787	sp|P02787|TRFE_HUMAN Serotransferrin O
0,213387	0,419429	0,610215	1,155433	sp|P0279C	sp|P02790|HEMO_HUMAN Hemopexin OS=
0,571477	0,738819	0,354302	0,516886	tr|Q5TZP0	tr|Q5TZP0|Q5TZP0_HUMAN Matrix metallo
0,595101	0,78011	-0,21628	-0,44935	sp|P04075	sp|P04075-2|ALDOA_HUMAN Isoform 2 of F
0,883284	0,906728	0,163558	0,141137	tr|Q76LA1	tr|Q76LA1|Q76LA1_HUMAN CSTB protein C
0,041217	0,149702	-1,64815	-2,34301	sp|P04083	sp|P04083|ANXA1_HUMAN Annexin A1 OS=
0,303804	0,485277	0,884523	1,014263	tr|O75942	tr|O75942|O75942_HUMAN Major prion pr
0,104435	0,254438	2,572591	1,839423	tr|H6VRG3	tr|H6VRG3|H6VRG3_HUMAN Cytokeratin-1
0,8472	0,902572	0,269262	0,188695	tr|E7EUT5	tr|E7EUT5|E7EUT5_HUMAN Glyceraldehyde
0,968467	0,977172	-0,02552	-0,03573	tr|A0A0U1	tr|A0A0U1RRH7|A0A0U1RRH7_HUMAN His
0,372969	0,669596	-0,18482	-0,63959	sp|P05067	sp|P05067-8|A4_HUMAN Isoform APP751 c
0,503912	0,667453	-0,69889	-0,65254	sp|P05109	sp|P05109|S10A8_HUMAN Protein S100-A8
0,627711	0,751163	-0,98937	-0,49062	tr|B2R7Y0	tr|B2R7Y0|B2R7Y0_HUMAN cDNA, FLJ9365
0,602661	0,752056	0,446723	0,49385	tr|A0A024	tr|A0A024QYT5|A0A024QYT5_HUMAN Serp
0,659023	0,820494	-0,12581	-0,34147	tr|A0A348	tr|A0A348GSH7|A0A348GSH7_HUMAN Serp
0,796615	0,881389	-0,13554	-0,22642	tr|A0A024	tr|A0A024RCA7|A0A024RCA7_HUMAN Ribo
0,691878	0,831435	-0,13294	-0,3192	sp|P05556	sp|P05556|ITB1_HUMAN Integrin beta-1 OS
0,079614	0,283562	-0,74964	-1,69774	sp|P06396	sp|P06396|GELS_HUMAN Gelsolin OS=Hom
0,440928	0,622382	0,617159	0,736772	tr|B2R4M6	tr|B2R4M6|B2R4M6_HUMAN Protein S100
0,170101	0,404983	-0,50986	-1,21887	tr|R4GN98	tr|R4GN98|R4GN98_HUMAN Protein S100 (
0,656652	0,804991	0,209469	0,385357	sp|P06733	sp|P06733|ENOA_HUMAN Alpha-enolase O
0,161909	0,410365	0,492697	1,230491	tr|A0A2U3	tr|A0A2U3TZU2|A0A2U3TZU2_HUMAN Glu
0,303423	0,671361	0,141182	0,632743	tr|A0A0S2	tr|A0A0S2Z4G7|A0A0S2Z4G7_HUMAN Nucl
5,07E-05	0	-5,71043	-10,4934	sp|P06753	sp|P06753-3|TPM3_HUMAN Isoform 3 of Tr
0,674537	0,786054	-1,25475	-0,43029	tr|B4DMR3	tr|B4DMR3|B4DMR3_HUMAN cDNA FLJ518
0,00035	0,041455	0,972214	4,564527	tr|A0A5F9	tr|A0A5F9ZHM4|A0A5F9ZHM4_HUMAN L-la
0,831577	0,901879	0,04797	0,152725	sp|P07237	sp|P07237|PDIA1_HUMAN Protein disulfide
0,004726	0,1032	1,199645	3,447239	sp|P07339	sp|P07339|CATD_HUMAN Cathepsin D OS=
0,050505	0,229143	-0,72085	-1,89037	sp|P07355	sp|P07355|ANXA2_HUMAN Annexin A2 OS=
0,145509	0,411567	0,390706	1,195672	tr|Q53FJ5	tr|Q53FJ5|Q53FJ5_HUMAN Prosaposin (Var
0,737447	0,825976	-0,4994	-0,33098	tr|Q5T8F0	tr|Q5T8F0|Q5T8F0_HUMAN Cathepsin L1 O
0,150722	0,3436	-0,75355	-1,38417	sp|P07737	sp|P07737|PROF1_HUMAN Profilin-1 OS=H
0,882289	0,91369	0,086437	0,132003	sp|P07858	sp|P07858|CATB_HUMAN Cathepsin B OS=H
0,133184	0,577216	-0,14828	-0,81132	sp|P07942	sp|P07942|LAMB1_HUMAN Laminin subuni
0,406776	0,672677	0,204539	0,627582	sp|P07996	sp|P07996|TSP1_HUMAN Thrombospondin-
0,502755	0,706995	-0,27956	-0,57368	tr|A0A024	tr|A0A024RD80|A0A024RD80_HUMAN Hea
0,346045	0,673056	-0,15415	-0,62097	tr|Q53H39	tr|Q53H39|Q53H39_HUMAN Activin beta-A
0,448404	0,644761	0,42178	0,68804	tr|A0A024	tr|A0A024RDW8|A0A024RDW8_HUMAN Cc
0,833778	0,898415	-0,08578	-0,17577	sp|P08581	sp|P08581-2|MET_HUMAN Isoform 2 of He
0,102537	0,268179	-1,66318	-1,78167	tr|Q53HU8	tr|Q53HU8|Q53HU8_HUMAN Vimentin (Fra
0,343487	0,643894	0,189701	0,674215	tr|V9HWE0	tr|V9HWE0|V9HWE0_HUMAN Annexin OS=
0,786715	0,874656	0,230617	0,254045	tr|A8MX94	tr|A8MX94|A8MX94_HUMAN GST class-pi C
0,031715	0,136111	-2,66143	-2,65939	sp|P09341	sp|P09341|GROA_HUMAN Growth-regulate
0,19233	0,35501	-1,40141	-1,36015	tr|A0A384	tr|A0A384MR27|A0A384MR27_HUMAN Ga
0,414889	0,686814	-0,18927	-0,60473	sp|P09486	sp|P09486|SPRC_HUMAN SPARC OS=Homo
0,000313	0,017333	-2,41583	-6,45563	tr|F8WCZ6	tr|F8WCZ6|F8WCZ6_HUMAN Complement (
0,670227	0,796916	-0,45749	-0,41133	tr|Q59EM9	tr|Q59EM9|Q59EM9_HUMAN Ubiquitin C v
0,115249	0,412324	0,359629	1,244965	sp|Q01105	sp|Q01105-3|SET_HUMAN Isoform 3 of Pro

0,070607	0,229097	-1,15124	-1,91039	tr|A0A0G2	tr|A0A0G2JIW1|A0A0G2JIW1_HUMAN Heat
0,983855	0,984053	0,008065	0,01683	tr|B4DJ51	tr|B4DJ51|B4DJ51_HUMAN Calmodulin OS=
0,217039	0,536785	0,243475	0,893724	sp|P10586	sp|P10586-2|PTPRF_HUMAN Isoform 2 of R
0,194305	0,407483	0,681185	1,228222	sp|P10599	sp|P10599|THIO_HUMAN Thioredoxin OS=H
0,841041	0,905025	-0,06952	-0,16201	sp|P11021	sp|P11021|BIP_HUMAN Endoplasmic reticu
0,132943	0,425606	-0,30119	-1,12394	tr|A0A024	tr|A0A024R972|A0A024R972_HUMAN Lami
0,343681	0,558699	-0,47386	-0,85652	sp|P11142	sp|P11142|HSP7C_HUMAN Heat shock cogr
0,286813	0,47742	-0,82541	-1,04144	tr|A0A087	tr|A0A087X0S5|A0A087X0S5_HUMAN Colla
0,734988	0,874954	0,083198	0,250279	tr|A0A024	tr|A0A024R694|A0A024R694_HUMAN Alph
0,0043	0,136216	-0,55505	-2,61539	tr|B3GN61	tr|B3GN61|B3GN61_HUMAN Truncated E-ca
0,538222	0,673	1,120377	0,62361	tr|A6XNE3	tr|A6XNE3|A6XNE3_HUMAN Interferon gam
0,049084	0,277314	0,526691	1,73434	tr|A0A384	tr|A0A384N6H1|A0A384N6H1_HUMAN Epi
0,013357	0,129524	0,767458	2,517029	tr|A0A090	tr|A0A090N8Y2|A0A090N8Y2_HUMAN Prot
0,355136	0,502761	1,279134	0,943441	tr|J3KPG2	tr|J3KPG2|J3KPG2_HUMAN Translationally-
0,268136	0,496863	0,412476	0,956629	tr|V9HWJ7	tr|V9HWJ7|V9HWJ7_HUMAN Epididymis se
0,218828	0,429631	0,567276	1,126491	sp|P13987	sp|P13987-2|CD59_HUMAN Isoform 2 of CD
0,907682	0,929958	0,192476	0,114691	tr|A0A0S2	tr|A0A0S2Z4A0|A0A0S2Z4A0_HUMAN Prote
0,090684	0,276958	-0,99342	-1,728	sp|P14543	sp|P14543-2|NID1_HUMAN Isoform 2 of Ni
0,112339	0,264667	-2,55868	-1,78952	sp|P14618	sp|P14618|KPYM_HUMAN Pyruvate kinase
0,697407	0,822314	-0,20471	-0,34297	tr|Q5CAQ5	tr|Q5CAQ5|Q5CAQ5_HUMAN Epididymis se
0,981886	0,98104	-0,05206	-0,02282	tr|B3KSS4	tr|B3KSS4|B3KSS4_HUMAN cDNA FLJ36858
0,003189	0,057846	2,614814	4,407696	tr|B4DMM	tr|B4DMM8|B4DMM8_HUMAN cDNA FLJ57
0,107486	0,278611	-1,39611	-1,7176	tr|B7Z5V2	tr|B7Z5V2|B7Z5V2_HUMAN cDNA FLJ54141
0,660591	0,798333	0,328818	0,408408	sp|P15514	sp|P15514|AREG_HUMAN Amphiregulin OS
0,802072	0,883143	-0,13092	-0,21993	tr|A0A024	tr|A0A024R8V7|A0A024R8V7_HUMAN Met
0,760604	0,862381	0,230121	0,28234	sp|P16070	sp|P16070|CD44_HUMAN CD44 antigen OS
0,324512	0,556707	0,394598	0,855493	sp|P16144	sp|P16144-5|ITB4_HUMAN Isoform Beta-4E
0,095347	0,299171	-0,67854	-1,57579	sp|P17900	sp|P17900|SAP3_HUMAN Ganglioside GM2
0,925756	0,942325	-0,1237	-0,09079	tr|A0A384	tr|A0A384NYN5|A0A384NYN5_HUMAN Me
0,315495	0,486231	-0,86255	-0,98748	tr|Q6FHU2	tr|Q6FHU2|Q6FHU2_HUMAN Phosphoglyce
0,250501	0,491627	0,386781	0,972621	sp|P19883	sp|P19883-2|FST_HUMAN Isoform 2 of Folli
0,000415	0,013	-2,58479	-6,2821	sp|P19957	sp|P19957|ELAF_HUMAN Elafin OS=Homo s
0,0636	0,180157	-2,55109	-2,17228	sp|P20827	sp|P20827|EFNA1_HUMAN Ephrin-A1 OS=H
0,013832	0,134359	0,854158	2,587314	tr|Q60FE6	tr|Q60FE6|Q60FE6_HUMAN Filamin A OS=H
0,053928	0,227082	-0,82735	-1,92488	tr|E9PPJ5|	tr|E9PPJ5|E9PPJ5_HUMAN Midkine (Fragme
0,971561	0,974067	0,063209	0,035372	sp|P21810	sp|P21810|PGS1_HUMAN Biglycan OS=Hom
0,633583	0,801489	-0,17983	-0,39562	tr|B4DPP0	tr|B4DPP0|B4DPP0_HUMAN cDNA FLJ5103
0,289775	0,482761	-0,6859	-1,00941	sp|P22223	sp|P22223-2|CADH3_HUMAN Isoform 2 of C
0,810868	0,892687	0,104921	0,203377	sp|P22392	sp|P22392-2|NDKB_HUMAN Isoform 3 of N
0,167898	0,322323	1,563692	1,46128	sp|P22528	sp|P22528|SPR1B_HUMAN Cornifin-B OS=H
0,689135	0,800489	0,859977	0,404149	sp|P22692	sp|P22692|IBP4_HUMAN Insulin-like growth
0,832552	0,900812	0,10928	0,185026	tr|B4DUV1	tr|B4DUV1|B4DUV1_HUMAN Fibulin-1 OS=H
0,386453	0,636984	0,246016	0,684543	sp|P23229	sp|P23229-4|ITA6_HUMAN Isoform Alpha-6
0,204926	0,429719	0,511928	1,134141	tr|V9HWC	tr|V9HWC6|V9HWC6_HUMAN Peptidyl-pro
0,003626	0,089333	-1,24182	-3,63853	tr|E9PK25	tr|E9PK25|E9PK25_HUMAN Cofilin, non-mu
0,400676	0,5485	1,130993	0,849186	sp|P24821	sp|P24821|TENA_HUMAN Tenascin OS=Hor
0,002042	0,030222	-3,64947	-5,0539	sp|P25391	sp|P25391|LAMA1_HUMAN Laminin subuni
0,282234	0,428855	-1,68183	-1,12415	sp|P25788	sp|P25788-2|PSA3_HUMAN Isoform 2 of Pr
0,622851	0,783073	-0,28887	-0,44334	sp|P25789	sp|P25789-2|PSA4_HUMAN Isoform 2 of Pr
0,18482	0,337208	-1,80512	-1,41668	tr|Q59F03	tr|Q59F03|Q59F03_HUMAN Integrin alpha

0,046087	0,209241	-0,85458	-2,01576	tr|V9HWC	tr|V9HWC0|V9HWC0_HUMAN Epididymis lu
0,017676	0,303373	0,283962	1,563503	tr|Q53G71	tr|Q53G71|Q53G71_HUMAN Calreticulin va
0,166023	0,323473	1,616096	1,472923	sp|P27930	sp|P27930|IL1R2_HUMAN Interleukin-1 rec
0,793044	0,880821	0,149978	0,233641	tr|Q5U0A0	tr|Q5U0A0|Q5U0A0_HUMAN Proteasome s
0,687187	0,825967	-0,1429	-0,32872	sp|P28799	sp|P28799|GRN_HUMAN Progranulin OS=H
0,08696	0,374885	-0,3447	-1,31456	sp|P29034	sp|P29034|S10A2_HUMAN Protein S100-A2
0,551527	0,69988	0,789271	0,590308	sp|P29317	sp|P29317-2|EPHA2_HUMAN Isoform 2 of E
0,946023	0,972811	0,01003	0,041629	tr|V9HWD	tr|V9HWD9|V9HWD9_HUMAN Epididymis l
0,591719	0,796478	0,151156	0,415269	tr|D9IAI1|	tr|D9IAI1|D9IAI1_HUMAN Epididymis secret
0,716748	0,844608	0,140572	0,301625	tr|B3KQT9	tr|B3KQT9|B3KQT9_HUMAN Protein disulfid
0,816676	0,880758	-0,41551	-0,23077	sp|P30530	sp|P30530|UFO_HUMAN Tyrosine-protein k
0,492338	0,666618	-0,53224	-0,64998	sp|P31431	sp|P31431-2|SDC4_HUMAN Isoform 2 of Sy
0,857703	0,905108	0,101829	0,159242	tr|B4DP51	tr|B4DP51|B4DP51_HUMAN cDNA FLJ5453
0,014909	0,138061	-1,06611	-2,71332	sp|P31946	sp|P31946-2|1433B_HUMAN Isoform Short
0,007776	0,136296	-0,93496	-2,94152	sp|P31947	sp|P31947|1433S_HUMAN 14-3-3 protein s
0,470792	0,722	-0,19211	-0,55466	tr|V9HWH	tr|V9HWH9|V9HWH9_HUMAN Protein S10
1	1	0	0	sp|P32004	sp|P32004-3|L1CAM_HUMAN Isoform 3 of L
0,442921	0,722505	-0,16014	-0,54787	tr|A0A384	tr|A0A384NPH9|A0A384NPH9_HUMAN Gly
0,482416	0,620313	2,167848	0,732752	sp|P35408	sp|P35408|PE2R4_HUMAN Prostaglandin E
0,005885	0,098133	-2,20083	-3,79903	sp|P35442	sp|P35442|TSP2_HUMAN Thrombospondin
0,856913	0,903742	0,093612	0,157822	sp|P35556	sp|P35556|FBN2_HUMAN Fibrillin-2 OS=Ho
0,709973	0,854614	0,110567	0,29037	tr|A0A024	tr|A0A024R1N1|A0A024R1N1_HUMAN Myo
0,354923	0,550371	-0,51647	-0,85112	tr|A0A024	tr|A0A024R2B6|A0A024R2B6_HUMAN Serp
0,919925	0,942497	0,051949	0,087834	tr|X6RJP6|	tr|X6RJP6|X6RJP6_HUMAN Transgelin-2 (Fra
0,014549	0,13625	1,178216	2,7943	tr|D3DSM	tr|D3DSM4|D3DSM4_HUMAN Collagen, typ
0,731864	0,861365	-0,11124	-0,27335	tr|V9HWF	tr|V9HWF2|V9HWF2_HUMAN Malate dehy
0,974745	0,977177	0,019704	0,028466	tr|Q6P1N4	tr|Q6P1N4|Q6P1N4_HUMAN IQGAP1 prote
0,623695	0,777413	-0,35866	-0,45599	tr|B1AK87	tr|B1AK87|B1AK87_HUMAN F-actin-capping
0,637214	0,766028	-0,7906	-0,47172	tr|Q8NE89	tr|Q8NE89|Q8NE89_HUMAN Similar to tissu
0,375726	0,553745	0,711263	0,855081	sp|P49327	sp|P49327|FAS_HUMAN Fatty acid synthase
0,279671	0,43076	-1,63385	-1,12826	tr|Q6FH59	tr|Q6FH59|Q6FH59_HUMAN VEGFC protein
0,55256	0,817384	-0,08568	-0,36514	tr|Q5SX91	tr|Q5SX91|Q5SX91_HUMAN Rab GDP disso
0,227501	0,390566	-1,97666	-1,28571	sp|P50895	sp|P50895|BCAM_HUMAN Basal cell adhesi
0,570042	0,747736	-0,29128	-0,50267	tr|J3KTF8|	tr|J3KTF8|J3KTF8_HUMAN Rho GDP-dissoci
0,317173	0,600233	0,264197	0,782037	sp|P52799	sp|P52799|EFNB2_HUMAN Ephrin-B2 OS=H
0,001365	0,016571	3,949118	5,519495	sp|P53634	sp|P53634|CATC_HUMAN Dipeptidyl peptid
0,278198	0,414903	3,291668	1,172834	sp|P55001	sp|P55001-3|MFAP2_HUMAN Isoform B of
0,035082	0,130537	-2,12921	-2,52744	sp|P55058	sp|P55058|PLTP_HUMAN Phospholipid tran
0,836206	0,89775	0,12192	0,184778	tr|Q96IF9|	tr|Q96IF9|Q96IF9_HUMAN VCP protein (Fra
0,059095	0,183692	1,846821	2,150504	sp|P55145	sp|P55145|MANF_HUMAN Mesencephalic a
0,762766	0,862422	-0,18044	-0,27093	sp|P55268	sp|P55268|LAMB2_HUMAN Laminin subuni
0,74256	0,822623	0,602427	0,328302	sp|P55290	sp|P55290-5|CAD13_HUMAN Isoform 5 of C
0,035286	0,152957	1,290633	2,343152	tr|E9PJK1|	tr|E9PJK1|E9PJK1_HUMAN Tetraspanin OS=
0,617184	0,798241	0,153579	0,395403	tr|B4DUI5	tr|B4DUI5|B4DUI5_HUMAN Triosephosphat
0,059108	0,293766	0,495687	1,632305	tr|G3V3I1|	tr|G3V3I1|G3V3I1_HUMAN Proteasome sub
0,001349	0,053	-1,51963	-4,51928	sp|P61769	sp|P61769|B2MG_HUMAN Beta-2-microglo
0,002833	0,0965	1,184003	3,73061	tr|Q53HV6	tr|Q53HV6|Q53HV6_HUMAN Epididymal se
0,013467	0,149867	-0,64402	-2,36499	sp|P61981	sp|P61981|1433G_HUMAN 14-3-3 protein g
0,035507	0,30386	-0,33152	-1,53471	sp|P62258	sp|P62258|1433E_HUMAN 14-3-3 protein e
0,874802	0,906539	0,118422	0,145446	tr|A2VCK8	tr|A2VCK8|A2VCK8_HUMAN Thymosin beta

0,006334	0,088421	1,816873	3,613861	tr|Q0VAS5	tr|Q0VAS5|Q0VAS5_HUMAN Histone H4 OS
0,825982	0,903659	0,092758	0,185367	sp|P62937	sp|P62937|PPIA_HUMAN Peptidyl-prolyl cis
0,131211	0,315448	-0,93179	-1,51085	sp|P62942	sp|P62942|FKB1A_HUMAN Peptidyl-prolyl c
0,007826	0,127721	-0,60664	-2,5112	tr|D0PNI1	tr|D0PNI1|D0PNI1_HUMAN Epididymis lum
0,134387	0,444324	-0,27676	-1,08501	tr|B4DVQ(	tr|B4DVQ0|B4DVQ0_HUMAN cDNA FLJ582
0,39993	0,5349	-2,57051	-0,88792	sp|P63313	sp|P63313|TYB10_HUMAN Thymosin beta-
0,011124	0,203037	0,439456	2,073324	sp|P78324	sp|P78324-2|SHPS1_HUMAN Isoform 2 of T
0,019763	0,17792	0,633691	2,202518	sp|P78504	sp|P78504|JAG1_HUMAN Protein jagged-1
0,012474	0,133286	-1,27256	-2,93335	sp|P80188	sp|P80188|NGAL_HUMAN Neutrophil gelati
1	1	0	0	tr|E9PKG6	tr|E9PKG6|E9PKG6_HUMAN Nucleobindin-
0,321796	0,489931	-1,00274	-0,99055	tr|Q86V58	tr|Q86V58|Q86V58_HUMAN Fibulin 2 OS=H
0,047535	0,3164	0,345974	1,488456	sp|P98172	sp|P98172|EFNB1_HUMAN Ephrin-B1 OS=H
0,103458	0,299026	0,859334	1,61478	tr|Q5T0R7	tr|Q5T0R7|Q5T0R7_HUMAN Adenylyl cycla
0,02738	0,139355	-3,31645	-2,82067	tr|Q7RTW	tr|Q7RTW3|Q7RTW3_HUMAN Neuregulin 1
0,107903	0,296608	0,911724	1,610173	sp|Q0238	sp|Q02388|CO7A1_HUMAN Collagen alpha-
0,774981	0,858972	0,432635	0,282087	tr|B4DGN	tr|B4DGN8|B4DGN8_HUMAN Procollagen-l
0,361427	0,55041	-0,57551	-0,85493	sp|Q0281	sp|Q02818|NUCB1_HUMAN Nucleobindin-1
0,615748	0,797219	-0,14945	-0,39382	tr|A0A024	tr|A0A024R3N3|A0A024R3N3_HUMAN Am
0,08162	0,420819	0,24392	1,149786	tr|A0A384	tr|A0A384NPQ2|A0A384NPQ2_HUMAN Epi
0,641192	0,812936	0,154488	0,375223	tr|A6NCT7	tr|A6NCT7|A6NCT7_HUMAN Collagen alpha
0,260171	0,405724	-1,88154	-1,18918	sp|Q0834	sp|Q08345-5|DDR1_HUMAN Isoform 4 of E
0,001073	0,0275	-2,3696	-5,26204	tr|A0A0S2	tr|A0A0S2Z3Y1|A0A0S2Z3Y1_HUMAN Gale
0,834722	0,893228	-0,38193	-0,20784	tr|G3V1S6	tr|G3V1S6|G3V1S6_HUMAN Polypeptide N-
0,024799	0,134632	-1,46061	-2,60508	tr|Q59G97	tr|Q59G97|Q59G97_HUMAN EGF-containin
0,117987	0,399514	-0,38711	-1,26808	sp|Q1284	sp|Q12841|FSTL1_HUMAN Follistatin-relate
0,031373	0,134	3,096969	2,705127	sp|Q1286(	sp|Q12860-2|CNTN1_HUMAN Isoform 2 of
0,668486	0,820852	-0,13075	-0,33729	tr|C9J4H5	tr|C9J4H5|C9J4H5_HUMAN Semaphorin-3F
0,434108	0,645766	-0,3288	-0,67543	tr|Q59FV9	tr|Q59FV9|Q59FV9_HUMAN PTK7 protein t
1	1	0	0	tr|H3BR90	tr|H3BR90|H3BR90_HUMAN Mesothelin (Fr
0,816446	0,884183	0,535747	0,233971	tr|F8WC5	tr|F8WC54|F8WC54_HUMAN Disintegrin an
0,955067	0,973687	0,023295	0,04722	tr|A0A0S2	tr|A0A0S2Z3R6|A0A0S2Z3R6_HUMAN Lami
0,856248	0,904643	-0,07732	-0,15298	sp|Q1375	sp|Q13753|LAMC2_HUMAN Laminin subun
0,712755	0,824921	-0,33599	-0,34926	tr|A0A024	tr|A0A024R2W4|A0A024R2W4_HUMAN Al
0,224337	0,617841	0,163759	0,750706	tr|B4DZY7	tr|B4DZY7|B4DZY7_HUMAN cDNA FLJ57022
0,007358	0,209684	0,381558	2,032676	sp|Q1451	sp|Q14512|FGFP1_HUMAN Fibroblast grow
0,161096	0,409926	-0,53084	-1,25525	tr|G3V511	tr|G3V511|G3V511_HUMAN Latent-transfo
0,36592	0,621559	-0,29258	-0,74186	sp|Q1508	sp|Q15084-3|PDIA6_HUMAN Isoform 3 of P
0,04014	0,141091	1,903532	2,405584	sp|Q1514	sp|Q15149-7|PLEC_HUMAN Isoform 7 of Pl
0,24236	0,419264	0,943591	1,16218	tr|Q6SYC2	tr|Q6SYC2|Q6SYC2_HUMAN Poliovirus rece
0,159783	0,387695	-0,60451	-1,29642	tr|Q59EZ1	tr|Q59EZ1|Q59EZ1_HUMAN Protein tyrosin
0,436432	0,63158	0,456621	0,713376	tr|A0A0S2	tr|A0A0S2Z4Q2|A0A0S2Z4Q2_HUMAN Tran
0,097117	0,4115	-0,31657	-1,23983	sp|Q1627(	sp|Q16270|IBP7_HUMAN Insulin-like growt
0,761996	0,861473	-0,15674	-0,26566	tr|A0A140	tr|A0A140VJI7|A0A140VJI7_HUMAN Testic
0,47785	0,718481	-0,18946	-0,54584	tr|B3KTA3	tr|B3KTA3|B3KTA3_HUMAN Fascin OS=Hon
0,014374	0,131	-0,86849	-2,58212	sp|Q1676	sp|Q16769|QPCT_HUMAN Glutaminyl-pept
0,735671	0,86	-0,12624	-0,27829	tr|A0A0A6	tr|A0A0A6YYF2|A0A0A6YYF2_HUMAN HCG
0,259281	0,406426	-1,87927	-1,19147	sp|Q24JP5	sp|Q24JP5|T132A_HUMAN Transmembrane
0,585059	0,749555	0,382145	0,505997	tr|B4DTF2	tr|B4DTF2|B4DTF2_HUMAN Annexin OS=Hc
0,587325	0,721952	-0,94522	-0,5461	sp|Q6H3X	sp|Q6H3X3|ULBP5_HUMAN UL-16 binding
0,137795	0,290773	-2,8782	-1,6632	tr|A0A024	tr|A0A024R091|A0A024R091_HUMAN von

0,083092	0,212	-2,68989	-2,00022	sp|Q6UVK	sp|Q6UVK1|CSPG4_HUMAN Chondroitin su
0,563424	0,726029	0,482642	0,548123	sp|Q6YHK:	sp|Q6YHK3-4|CD109_HUMAN Isoform 4 of
0,218305	0,408444	-0,91375	-1,21963	tr|B3KRK1	tr|B3KRK1|B3KRK1_HUMAN cDNA FLJ34442
0,237249	0,406532	-1,90919	-1,25404	sp|Q86X29	sp|Q86X29-6|LSR_HUMAN Isoform 6 of Lipo
0,028499	0,136	-2,25431	-2,68475	sp|Q8IUX7	sp|Q8IUX7|AEBP1_HUMAN Adipocyte enha
0,344816	0,488411	1,946838	0,989777	sp|Q8IWU	sp|Q8IWU5-2|SULF2_HUMAN Isoform 2 of
0,915774	0,938014	-0,08736	-0,09865	sp|Q8NBP	sp|Q8NBP7|PCSK9_HUMAN Proprotein con
0,959267	0,972854	0,029464	0,045409	tr|B7Z5C1	tr|B7Z5C1|B7Z5C1_HUMAN cDNA FLJ56126
0,045871	0,196226	-1,02958	-2,10242	sp|Q9252(	sp|Q92520|FAM3C_HUMAN Protein FAM3C
0,485787	0,672479	0,429569	0,640145	sp|Q9262(	sp|Q92626|PXDN_HUMAN Peroxidasin hom
0,220128	0,488086	-0,37471	-1,01972	sp|Q9274:	sp|Q92743|HTRA1_HUMAN Serine protease
0,71405	0,818722	0,345754	0,348777	sp|Q9282:	sp|Q92823-5|NRCAM_HUMAN Isoform 5 of
0,596573	0,811068	0,110844	0,374176	sp|Q9289(	sp|Q92896|GSLG1_HUMAN Golgi apparatus
0,353387	0,500535	-1,15712	-0,93959	tr|C9JMK5	tr|C9JMK5|C9JMK5_HUMAN Phosphoinosit
0,861664	0,897522	0,27822	0,172088	tr|V9HWC	tr|V9HWC2|V9HWC2_HUMAN Maillard deg
0,69475	0,821483	0,21753	0,349127	tr|B7Z4S8	tr|B7Z4S8|B7Z4S8_HUMAN Asparaginyl end
0,207207	0,415418	0,892112	1,245994	sp|Q9971!	sp|Q99715-4|COCA1_HUMAN Isoform 4 of
0,125488	0,314382	-0,82252	-1,50222	sp|Q9998!	sp|Q99985|SEM3C_HUMAN Semaphorin-3C
0,1539	0,341737	0,815796	1,392734	tr|A0A140	tr|A0A140VJY7|A0A140VJY7_HUMAN Thior
0,004867	0,101818	-1,14545	-3,38445	sp|Q9BRK!	sp|Q9BRK5-4|CAB45_HUMAN Isoform 4 of
0,039148	0,207929	-0,76895	-2,03895	sp|Q9GZN	sp|Q9GZM7-3|TINAL_HUMAN Isoform 3 of
0,207547	0,414588	-0,6926	-1,19693	sp|Q9H29!	sp|Q9H299|SH3L3_HUMAN SH3 domain-bir
0,177966	0,337305	-1,5277	-1,42097	sp|Q9NPR	sp|Q9NPR2-2|SEM4B_HUMAN Isoform 2 of
0,560329	0,704277	0,87073	0,581784	tr|F8VNQ3	tr|F8VNQ3|F8VNQ3_HUMAN Teneurin-2 OS
0,090493	0,301086	0,645386	1,580757	tr|Q4ZG85	tr|Q4ZG85|Q4ZG85_HUMAN Uncharacterize
0,377943	0,62032	-0,33852	-0,75216	sp|Q9P2B:	sp|Q9P2B2|FPRP_HUMAN Prostaglandin F2
0,022448	0,218133	-0,49561	-1,96646	sp|Q9UBP	sp|Q9UBP4|DKK3_HUMAN Dickkopf-relatec
0,103687	0,26713	1,623938	1,770139	tr|Q5U00(	tr|Q5U000|Q5U000_HUMAN Cathepsin X O
0,846947	0,902388	0,138339	0,177219	tr|B7Z5J4|	tr|B7Z5J4|B7Z5J4_HUMAN Carboxypeptidas
0,906241	0,938118	-0,06373	-0,10371	sp|Q9UMI	sp|Q9UMD9-2|COHA1_HUMAN Isoform 2 o
0,130391	0,343216	0,632086	1,405929	sp|Q9UNN	sp|Q9UNN8|EPCR_HUMAN Endothelial prot
0,010525	0,099652	2,209536	3,372905	tr|A0A2I2I	tr|A0A2I2MP48|A0A2I2MP48_HUMAN Kalli
0,539322	0,67804	0,886234	0,612962	tr|W6I206	tr|W6I206|W6I206_HUMAN Lysyl oxidase h
0,348354	0,484322	2,031884	0,98431	tr|B2RAF9	tr|B2RAF9|B2RAF9_HUMAN Suppressor of t
0,610333	0,778577	0,233089	0,440525	tr|D3DVF0	tr|D3DVF0|D3DVF0_HUMAN Junctional adh
0,627879	0,780018	-0,31152	-0,44265	tr|A0A024	tr|A0A024R462|A0A024R462_HUMAN Fibro
0,001844	0,0324	-2,00883	-4,62096	tr|W8GRN	tr|W8GRM7|W8GRM7_HUMAN Kallikrein 1
0,805142	0,885824	0,25393	0,235951	tr|A0A024	tr|A0A024R884|A0A024R884_HUMAN Tena
0,069573	0,324	-0,39106	-1,44914	tr|A0A024	tr|A0A024RAB6|A0A024RAB6_HUMAN Hep
0,057841	0,148167	-4,8278	-2,33289	tr|D7RVA3	tr|D7RVA3|D7RVA3_HUMAN Granulocyte c
0,358636	0,491894	-2,22648	-0,96661	tr|A0A1V1	tr|A0A1V1IFQ2|A0A1V1IFQ2_HUMAN MHC
0,358259	0,493105	-1,90278	-0,96031	tr|A0A1D6ZNI8|A0A1D6ZNI8_HUMAN;tr|A0A0S4XRM(	
0,713169	0,815412	-0,5597	-0,36387	tr|A0A288	tr|A0A288Q9P9|A0A288Q9P9_HUMAN MH
0,677388	0,803791	0,388665	0,396347	tr|A0A384	tr|A0A384MR03|A0A384MR03_HUMAN Fib
0,00025	0	-6,72706	-8,12234	tr|F5H2D0	tr|F5H2D0|F5H2D0_HUMAN Complement s
0,138829	0,315136	-1,04036	-1,50491	tr|A8K6T3	tr|A8K6T3|A8K6T3_HUMAN cDNA FLJ78674
0,288974	0,486	-0,58349	-0,98552	tr|D3DS13	tr|D3DS13|D3DS13_HUMAN Ribonuclease /
0,305282	0,6312	0,194068	0,718828	tr|B3KNN9	tr|B3KNN9|B3KNN9_HUMAN Activated leuk
0,187686	0,439911	0,385751	1,093116	tr|B4E1B2	tr|B4E1B2|B4E1B2_HUMAN Beta-1 metal-b

0,469581	0,640409	0,550268	0,684735	tr|B7Z507	tr|B7Z507|B7Z507_HUMAN cDNA FLJ51036
0,025389	0,130483	4,023942	2,921018	tr|C9JB55|	tr|C9JB55|C9JB55_HUMAN Serotransferrin
0,453781	0,598682	2,028084	0,780596	tr|C9JVG0	tr|C9JVG0|C9JVG0_HUMAN Serotransferrin
0,048117	0,161469	-1,7889	-2,2718	tr|E7ET40	tr|E7ET40|E7ET40_HUMAN Urokinase-type
0,787338	0,861852	-0,49638	-0,2692	tr|V5NQE0	tr|V5NQE0|V5NQE0_HUMAN MHC class I ar
0,959386	0,972751	-0,1059	-0,05095	tr|H0YBI8|	tr|H0YBI8|H0YBI8_HUMAN Vascular endoth
0,448264	0,717455	0,160405	0,543675	tr|Q5M8T4	tr|Q5M8T4|Q5M8T4_HUMAN Connective ti

:ytoskeletal 14 OS=Homo sapiens OX=9606 GN=KRT14 PE=1 SV=4;tr|A0A024R1X6|A0A024R1X6_HU
;7, highly similar to Keratin, type II cytoskeletal 6A OS=Homo sapiens OX=9606 PE=2 SV=1;sp|P02538

ytoskeletal 5 OS=Homo sapiens OX=9606 GN=KRT5 PE=1 SV=3;tr|B4E1T1|B4E1T1_HUMAN cDNA FL
toskeletal 9 OS=Homo sapiens OX=9606 GN=KRT9 PE=1 SV=3;tr|K7EQQ3|K7EQQ3_HUMAN Keratin,
ytoskeletal 17 OS=Homo sapiens OX=9606 GN=KRT17 PE=1 SV=2;;tr|F5GWP8|F5GWP8_HUMAN Ke
protein (Fragment) OS=Homo sapiens OX=9606 GN=C1 segment PE=4 SV=1;tr|Q5CZ94|Q5CZ94_HU
oride intracellular channel protein OS=Homo sapiens OX=9606 PE=3 SV=1;tr|Q5SRT3|Q5SRT3_HUM
ase 1 OS=Homo sapiens OX=9606 GN=QSOX1 PE=1 SV=3;tr|A0A140VKE5|A0A140VKE5_HUMAN Sul
dase OS=Homo sapiens OX=9606 GN=MANBA PE=1 SV=1;tr|B4DT18|B4DT18_HUMAN Beta-mannos
Agrin OS=Homo sapiens OX=9606 GN=AGRN;sp|O00468-7|AGRIN_HUMAN Isoform 7 of Agrin OS=Ho
Syntenin-1 OS=Homo sapiens OX=9606 GN=SDCBP;tr|G5EA09|G5EA09_HUMAN Syndecan binding p
)S=Homo sapiens OX=9606 GN=CYR61 PE=1 SV=1;sp|O00622|CCN1_HUMAN CCN family member 1
d metalloproteinase domain-containing protein 10 OS=Homo sapiens OX=9606 GN=ADAM10 PE=2 S
tory light chain MRCL3 variant (Fragment) OS=Homo sapiens OX=9606 PE=2 SV=1;sp|P19105|ML12

ease inhibitor 1 OS=Homo sapiens OX=9606 GN=SPINT1 PE=1 SV=2;tr|H3BVD9|H3BVD9_HUMAN Ku
in alpha 4 isoform 1 (Fragment) OS=Homo sapiens OX=9606 GN=ACTN4 PE=2 SV=1;sp|O43707|ACT
Protocadherin-7 OS=Homo sapiens OX=9606 GN=PCDH7;sp|O60245|PCDH7_HUMAN Protocadherin
pe 1-K OS=Homo sapiens OX=9606 GN=H2BC12 PE=1 SV=3;tr|A0A024RCJ9|A0A024RCJ9_HUMAN Hi
5, highly similar to Homo sapiens cathepsin L2 (CTSL2), mRNA OS=Homo sapiens OX=9606 PE=2 SV=1
3, highly similar to WD repeat protein 1 OS=Homo sapiens OX=9606 PE=2 SV=1;sp|O75083-3|WDR1_
A OS=Homo sapiens OX=9606 GN=SEMA7A PE=1 SV=1;sp|O75326-2|SEM7A_HUMAN Isoform 2 of Se
lamin-B OS=Homo sapiens OX=9606 GN=FLNB;sp|O75369-9|FLNB_HUMAN Isoform 9 of Filamin-B O
rpin B7 OS=Homo sapiens OX=9606 GN=SERPINB7;sp|O75635|SPB7_HUMAN Serpin B7 OS=Homo s
l, highly similar to Attractin OS=Homo sapiens OX=9606 PE=2 SV=1;sp|O75882-3|ATRN_HUMAN Iso
4 fis, clone MAMMA1002485, highly similar to Stanniocalcin-2 OS=Homo sapiens OX=9606 PE=2 SV=1
rotein 1 OS=Homo sapiens OX=9606 GN=DKK1 PE=2 SV=1;sp|O94907|DKK1_HUMAN Dickkopf-relat

48 fis, clone NT2RP2000028, highly similar to Serine protease 23 OS=Homo sapiens OX=9606 PE=2 S
protein DKFZp686D0114 OS=Homo sapiens OX=9606 GN=DKFZp686D0114 PE=4 SV=1;tr|B2RBR3|B
ollistatin-related protein 3 OS=Homo sapiens OX=9606 GN=FSTL3;tr|A0A024R1Y8|A0A024R1Y8_HU
ydrogenase OS=Homo sapiens OX=9606 GN=HEL-S-133P PE=2 SV=1;sp|P00338|LDHA_HUMAN L-la
otransferase OS=Homo sapiens OX=9606 PE=2 SV=1;sp|P00505-2|AATM_HUMAN Isoform 2 of Asp
osphoglycerate kinase 1 OS=Homo sapiens OX=9606 GN=PGK1;tr|V9HWF4|V9HWF4_HUMAN Phos
tivator OS=Homo sapiens OX=9606 GN=PLAT PE=2 SV=1;sp|P00750|TPA_HUMAN Tissue-type plasm
S=Homo sapiens OX=9606 GN=HEL-S-62p PE=2 SV=1;sp|P01024|CO3_HUMAN Complement C3 OS=
inase inhibitor 1 OS=Homo sapiens OX=9606 GN=TIMP1 PE=1 SV=1;tr|Q6FGX5|Q6FGX5_HUMAN Me
dymis secretory protein Li 2 OS=Homo sapiens OX=9606 GN=HEL-S-2 PE=2 SV=1;sp|P01034|CYTC_H
lipoprotein receptor OS=Homo sapiens OX=9606 GN=LDLR PE=1 SV=1;tr|Q9UH51|Q9UH51_HUMAN
ent) OS=Homo sapiens OX=9606 GN=INS PE=2 SV=1;tr|A6XGL2|A6XGL2_HUMAN Insulin OS=Homo
appa constant OS=Homo sapiens OX=9606 GN=IGKC PE=1 SV=2;tr|A0A5H1ZRQ3|A0A5H1ZRQ3_HU
0 fis, clone MAMMA1000370, highly similar to Ig alpha-1 chain C region OS=Homo sapiens OX=9606
n alpha 1 (Fragment) OS=Homo sapiens OX=9606 PE=2 SV=1;tr|A0A3B3ISV3|A0A3B3ISV3_HUMAN
S=Homo sapiens OX=9606 GN=LMNA PE=1 SV=1;tr|A0A384MQX1|A0A384MQX1_HUMAN Epididymi
ipoprotein E isoform 1 (Fragment) OS=Homo sapiens OX=9606 GN=APOE PE=2 SV=1;sp|P02649|APO

omo sapiens OX=9606 GN=FN1 PE=1 SV=5;sp|P02751-7|FINC_HUMAN Isoform 7 of Fibronectin OS=
=Homo sapiens OX=9606 GN=GC PE=1 SV=1;sp|P02774-2|VTDB_HUMAN Isoform 2 of Vitamin D-bin
S=Homo sapiens OX=9606 GN=TF PE=1 SV=3;tr|Q06AH7|Q06AH7_HUMAN Beta-1 metal-binding glo

proteinase 1 (Interstitial collagenase) OS=Homo sapiens OX=9606 PE=2 SV=1;tr|Q53G96|Q53G96_H
Fructose-bisphosphate aldolase A OS=Homo sapiens OX=9606 GN=ALDOA;tr|V9HWN7|V9HWN7_HU
OS=Homo sapiens OX=9606 GN=CSTB PE=2 SV=1;sp|P04080|CYTB_HUMAN Cystatin-B OS=Homo sap
=Homo sapiens OX=9606 GN=ANXA1 PE=1 SV=2;tr|Q5TZZ9|Q5TZZ9_HUMAN Annexin OS=Homo sap
otein OS=Homo sapiens OX=9606 GN=PRNP PE=3 SV=2;tr|Q86XR1|Q86XR1_HUMAN Major prion pr
OS=Homo sapiens OX=9606 GN=KRT1 PE=3 SV=1;tr|H6VRG0|H6VRG0_HUMAN Cytokeratin-1 OS=H
e-3-phosphate dehydrogenase OS=Homo sapiens OX=9606 GN=GAPDH PE=1 SV=1;sp|P04406-2|G3P
tone H2A OS=Homo sapiens OX=9606 PE=3 SV=1;sp|Q96KK5|H2A1H_HUMAN Histone H2A type 1-H
of Amyloid-beta precursor protein OS=Homo sapiens OX=9606 GN=APP;sp|P05067|A4_HUMAN Amy

4, highly similar to Homo sapiens serpin peptidase inhibitor, clade B (ovalbumin), member 2 (SERPINI
pin peptidase inhibitor, clade E (Nexin, plasminogen activator inhibitor type 1), member 1, isoform CF
pin peptidase inhibitor clade G member 1 OS=Homo sapiens OX=9606 GN=SERPING1 PE=2 SV=1;sp|F
osomal protein, large, P2, isoform CRA_a OS=Homo sapiens OX=9606 GN=RPLP2 PE=3 SV=1;sp|P053
S=Homo sapiens OX=9606 GN=ITGB1 PE=1 SV=2;sp|P05556-2|ITB1_HUMAN Isoform 2 of Integrin be
o sapiens OX=9606 GN=GSN PE=1 SV=1;sp|P06396-2|GELS_HUMAN Isoform 2 of Gelsolin OS=Homo
OS=Homo sapiens OX=9606 PE=2 SV=1;sp|P06702|S10A9_HUMAN Protein S100-A9 OS=Homo sapie
(Fragment) OS=Homo sapiens OX=9606 GN=S100A6 PE=1 SV=1;sp|P06703|S10A6_HUMAN Protein S
S=Homo sapiens OX=9606 GN=ENO1 PE=1 SV=2;tr|A0A024R4F1|A0A024R4F1_HUMAN 2-phospho-D
cose-6-phosphate isomerase OS=Homo sapiens OX=9606 GN=GPI PE=1 SV=1;sp|P06744-2|G6PI_HU
eophosmin (Fragment) OS=Homo sapiens OX=9606 GN=NPM1 PE=2 SV=1;sp|P06748-2|NPM_HUM
ropomyosin alpha-3 chain OS=Homo sapiens OX=9606 GN=TPM3;tr|A0A087WWU8|A0A087WWU8_
96, highly similar to Glia-derived nexin OS=Homo sapiens OX=9606 PE=2 SV=1;tr|A0A024R498|A0A
actate dehydrogenase OS=Homo sapiens OX=9606 GN=LDHB PE=1 SV=1;sp|P07195|LDHB_HUMAN L
-isomerase OS=Homo sapiens OX=9606 GN=P4HB PE=1 SV=3;tr|A0A024R8S5|A0A024R8S5_HUMAN
Homo sapiens OX=9606 GN=CTSD PE=1 SV=1;tr|V9HWI3|V9HWI3_HUMAN Cathepsin D OS=Homo s
=Homo sapiens OX=9606 GN=ANXA2 PE=1 SV=2;tr|A0A024R5Z7|A0A024R5Z7_HUMAN Annexin OS=
iant Gaucher disease and variant metachromatic leukodystrophy) variant (Fragment) OS=Homo sapie
S=Homo sapiens OX=9606 GN=CTSL PE=1 SV=1;tr|B3KQK4|B3KQK4_HUMAN cDNA FLJ90619 fis, clo
omo sapiens OX=9606 GN=PFN1 PE=1 SV=2;tr|K7EJ44|K7EJ44_HUMAN Profilin OS=Homo sapiens O
Homo sapiens OX=9606 GN=CTSB PE=1 SV=3;tr|A0A024R374|A0A024R374_HUMAN Cathepsin B OS=
t beta-1 OS=Homo sapiens OX=9606 GN=LAMB1 PE=1 SV=2;tr|G3XAI2|G3XAI2_HUMAN Laminin sub
-1 OS=Homo sapiens OX=9606 GN=THBS1 PE=1 SV=2;sp|P07996-2|TSP1_HUMAN Isoform 2 of Thro
t shock protein 90kDa alpha (Cytosolic), class B member 1, isoform CRA_a OS=Homo sapiens OX=960
chain (Fragment) OS=Homo sapiens OX=9606 PE=2 SV=1;tr|A4D1W7|A4D1W7_HUMAN Activin bet
ollagen, type IV, alpha 2, isoform CRA_a OS=Homo sapiens OX=9606 GN=COL4A2 PE=4 SV=1;sp|P085
patocyte growth factor receptor OS=Homo sapiens OX=9606 GN=MET;sp|P08581|MET_HUMAN He
gment) OS=Homo sapiens OX=9606 PE=2 SV=1;sp|P08670|VIME_HUMAN Vimentin OS=Homo sapie
Homo sapiens OX=9606 GN=HEL-S-7 PE=2 SV=1;sp|P08758|ANXA5_HUMAN Annexin A5 OS=Homo s
OS=Homo sapiens OX=9606 GN=GSTP1 PE=1 SV=1;tr|V9HWE9|V9HWE9_HUMAN GST class-pi OS=Ho

lectin OS=Homo sapiens OX=9606 PE=2 SV=1;sp|P09382|LEG1_HUMAN Galectin-1 OS=Homo sapie
sapiens OX=9606 GN=SPARC PE=1 SV=1;tr|D3DQH8|D3DQH8_HUMAN Osteonectin OS=Homo sapie
C1s subcomponent OS=Homo sapiens OX=9606 GN=C1S PE=1 SV=1;tr|A0A087X232|A0A087X232_H
ariant (Fragment) OS=Homo sapiens OX=9606 PE=2 SV=1;sp|P62979|RS27A_HUMAN Ubiquitin-40S
tein SET OS=Homo sapiens OX=9606 GN=SET;tr|A0A0C4DFV9|A0A0C4DFV9_HUMAN Protein SET OS

: shock 70 kDa protein 1B OS=Homo sapiens OX=9606 GN=HSPA1B PE=1 SV=1;sp|P0DMV8|HS71A_H
:Homo sapiens OX=9606 GN=HEL-S-72 PE=2 SV=1;sp|P0DP23|CALM1_HUMAN Calmodulin-1 OS=Ho
eceptor-type tyrosine-protein phosphatase F OS=Homo sapiens OX=9606 GN=PTPRF;sp|P10586|PTF
Homo sapiens OX=9606 GN=TXN PE=1 SV=3;tr|H9ZYJ2|H9ZYJ2_HUMAN Thioredoxin OS=Homo sapie
lum chaperone BiP OS=Homo sapiens OX=9606 GN=HSPA5 PE=1 SV=2;tr|V9HWB4|V9HWB4_HUMA
inin, gamma 1 (Formerly LAMB2), isoform CRA_a OS=Homo sapiens OX=9606 GN=LAMC1 PE=4 SV=1
nate 71 kDa protein OS=Homo sapiens OX=9606 GN=HSPA8 PE=1 SV=1;tr|V9HW22|V9HW22_HUMA
gen alpha-1(VI) chain OS=Homo sapiens OX=9606 GN=COL6A1 PE=1 SV=1;tr|A0A384P5H7|A0A384P
a-actinin cytoskeletal isoform OS=Homo sapiens OX=9606 GN=ACTN1 PE=2 SV=1;sp|P12814|ACTN1
adherin OS=Homo sapiens OX=9606 GN=CDH1 PE=2 SV=1;tr|D3XNU5|D3XNU5_HUMAN E-cadherin
ıma-inducible protein 30 preproprotein OS=Homo sapiens OX=9606 PE=2 SV=1;tr|A8K686|A8K686_
didymis secretory sperm binding protein OS=Homo sapiens OX=9606 PE=2 SV=1;tr|B4DPU3|B4DPU3
:ein disulfide-isomerase A4 OS=Homo sapiens OX=9606 GN=ERP70 PE=2 SV=1;sp|P13667|PDIA4_HU
controlled tumor protein OS=Homo sapiens OX=9606 GN=TPT1 PE=1 SV=1;tr|E9PJF7|E9PJF7_HUMA
cretory protein Li 37 OS=Homo sapiens OX=9606 GN=HEL-S-37 PE=2 SV=1;tr|Q53FI1|Q53FI1_HUMA
)59 glycoprotein OS=Homo sapiens OX=9606 GN=CD59;tr|E9PNW4|E9PNW4_HUMAN CD59 glycopr
ein kinase C substrate 80K-H isoform 4 (Fragment) OS=Homo sapiens OX=9606 GN=PRKCSH PE=2 SV=
dogen-1 OS=Homo sapiens OX=9606 GN=NID1;sp|P14543|NID1_HUMAN Nidogen-1 OS=Homo sapie
PKM OS=Homo sapiens OX=9606 GN=PKM PE=1 SV=4;tr|V9HWB8|V9HWB8_HUMAN Pyruvate kinas
:cretory sperm binding protein OS=Homo sapiens OX=9606 GN=TRA1 PE=2 SV=1;tr|V9HWP2|V9HWF
fis, clone ASTRO2015185, highly similar to POLIOVIRUS RECEPTOR OS=Homo sapiens OX=9606 PE=2
'328, highly similar to Beta-1,4-galactosyltransferase 1 OS=Homo sapiens OX=9606 PE=2 SV=1;tr|B7Z
., highly similar to Ezrin OS=Homo sapiens OX=9606 PE=2 SV=1;tr|V9HW42|V9HW42_HUMAN Epidic
=Homo sapiens OX=9606 GN=AREG PE=1 SV=2;tr|D6RFX5|D6RFX5_HUMAN Amphiregulin OS=Homo
alloproteinase inhibitor 2 OS=Homo sapiens OX=9606 GN=TIMP2 PE=3 SV=1;tr|A0A140VK57|A0A14
=Homo sapiens OX=9606 GN=CD44 PE=1 SV=3;sp|P16070-8|CD44_HUMAN Isoform 8 of CD44 antig
: of Integrin beta-4 OS=Homo sapiens OX=9606 GN=ITGB4;tr|Q59H46|Q59H46_HUMAN Integrin bet

tavinculin OS=Homo sapiens OX=9606 PE=2 SV=1;tr|A0A024QZN4|A0A024QZN4_HUMAN Metavinc
:rate mutase (Fragment) OS=Homo sapiens OX=9606 GN=PGAM1 PE=2 SV=1;tr|Q6FHK8|Q6FHK8_HU
statin OS=Homo sapiens OX=9606 GN=FST;tr|A0A024QZU6|A0A024QZU6_HUMAN Follistatin, isofo

Homo sapiens OX=9606 GN=EFNA1 PE=1 SV=2;tr|B2R7U1|B2R7U1_HUMAN Ephrin RBD domain-cont
Homo sapiens OX=9606 GN=FLNA PE=2 SV=1;sp|P21333|FLNA_HUMAN Filamin-A OS=Homo sapiens
ent) OS=Homo sapiens OX=9606 GN=MDK PE=1 SV=1;sp|P21741|MK_HUMAN Midkine OS=Homo sa
no sapiens OX=9606 GN=BGN PE=1 SV=2;tr|B4DNL4|B4DNL4_HUMAN Biglycan OS=Homo sapiens O
2, highly similar to CD9 antigen OS=Homo sapiens OX=9606 PE=2 SV=1;tr|A6NNI4|A6NNI4_HUMAN
Cadherin-3 OS=Homo sapiens OX=9606 GN=CDH3;sp|P22223|CADH3_HUMAN Cadherin-3 OS=Homo
ucleoside diphosphate kinase B OS=Homo sapiens OX=9606 GN=NME2;tr|Q32Q12|Q32Q12_HUMA
omo sapiens OX=9606 GN=SPRR1B PE=1 SV=2;tr|Q2I377|Q2I377_HUMAN Small proline rich protein
n factor-binding protein 4 OS=Homo sapiens OX=9606 GN=IGFBP4 PE=1 SV=2;tr|A0A024R1U8|A0A0
Homo sapiens OX=9606 PE=2 SV=1;sp|P23142-4|FBLN1_HUMAN Isoform C of Fibulin-1 OS=Homo sa
iX2A of Integrin alpha-6 OS=Homo sapiens OX=9606 GN=ITGA6;sp|P23229-2|ITA6_HUMAN Isoform
lyl cis-trans isomerase OS=Homo sapiens OX=9606 GN=HEL-S-39 PE=2 SV=1;sp|P23284|PPIB_HUMA
scle isoform OS=Homo sapiens OX=9606 GN=CFL1 PE=1 SV=1;tr|E9PP50|E9PP50_HUMAN Cofilin, n
no sapiens OX=9606 GN=TNC PE=1 SV=3;sp|P24821-3|TENA_HUMAN Isoform 3 of Tenascin OS=Ho

oteasome subunit alpha type-3 OS=Homo sapiens OX=9606 GN=PSMA3;tr|Q6IB71|Q6IB71_HUMAN
oteasome subunit alpha type-4 OS=Homo sapiens OX=9606 GN=PSMA4;tr|H0YN18|H0YN18_HUMA
3 isoform b, variant (Fragment) OS=Homo sapiens OX=9606 PE=2 SV=1;tr|B4E0H8|B4E0H8_HUMAN

uminal protein 70 OS=Homo sapiens OX=9606 GN=HEL70 PE=2 SV=1;sp|P26038|MOES_HUMAN Mo
riant (Fragment) OS=Homo sapiens OX=9606 PE=2 SV=1;tr|V9HW88|V9HW88_HUMAN Calreticulin
eptor type 2 OS=Homo sapiens OX=9606 GN=IL1R2 PE=1 SV=1;sp|P27930-2|IL1R2_HUMAN Isoform
ubunit alpha type OS=Homo sapiens OX=9606 PE=2 SV=1;tr|A0A109NGN6|A0A109NGN6_HUMAN F
omo sapiens OX=9606 GN=GRN PE=1 SV=2;tr|B4DJI2|B4DJI2_HUMAN cDNA FLJ53342, highly similar
OS=Homo sapiens OX=9606 GN=S100A2 PE=1 SV=3;tr|R4GN49|R4GN49_HUMAN Protein S100-A2
phrin type-A receptor 2 OS=Homo sapiens OX=9606 GN=EPHA2;tr|A0A024QZA8|A0A024QZA8_HU
uminal protein 107 OS=Homo sapiens OX=9606 GN=HEL107 PE=2 SV=1;tr|Q53EM5|Q53EM5_HUMA
tory protein Li 34 OS=Homo sapiens OX=9606 GN=HEL-S-34 PE=2 SV=1;sp|P30086|PEBP1_HUMAN P
de-isomerase OS=Homo sapiens OX=9606 PE=2 SV=1;tr|B3KQT2|B3KQT2_HUMAN Protein disulfide-
kinase receptor UFO OS=Homo sapiens OX=9606 GN=AXL PE=1 SV=4;sp|P30530-2|UFO_HUMAN Isof
ndecan-4 OS=Homo sapiens OX=9606 GN=SDC4;sp|P31431|SDC4_HUMAN Syndecan-4 OS=Homo sa
3, highly similar to Heterogeneous nuclear ribonucleoprotein H OS=Homo sapiens OX=9606 PE=2 SV=
of 14-3-3 protein beta/alpha OS=Homo sapiens OX=9606 GN=YWHAB;tr|V9HWD6|V9HWD6_HUMA
igma OS=Homo sapiens OX=9606 GN=SFN PE=1 SV=1;sp|P31947-2|1433S_HUMAN Isoform 2 of 14-
OS=Homo sapiens OX=9606 GN=HEL-S-43 PE=2 SV=1;tr|B2R5H0|B2R5H0_HUMAN Protein S100 OS
Neural cell adhesion molecule L1 OS=Homo sapiens OX=9606 GN=L1CAM;sp|P32004-2|L1CAM_HU
pican-1 OS=Homo sapiens OX=9606 PE=2 SV=1;sp|P35052|GPC1_HUMAN Glypican-1 OS=Homo sapi

-2 OS=Homo sapiens OX=9606 GN=THBS2 PE=1 SV=2;tr|A0A3B3ITK0|A0A3B3ITK0_HUMAN Thromb
mo sapiens OX=9606 GN=FBN2 PE=1 SV=3;tr|D6RJI3|D6RJI3_HUMAN Fibrillin-2 OS=Homo sapiens O
osin, heavy polypeptide 9, non-muscle, isoform CRA_a OS=Homo sapiens OX=9606 GN=MYH9 PE=3 S
in peptidase inhibitor, clade B (Ovalbumin), member 5, isoform CRA_b OS=Homo sapiens OX=9606 G
agment) OS=Homo sapiens OX=9606 GN=TAGLN2 PE=1 SV=1;tr|A0A384MTL2|A0A384MTL2_HUMA
e XVIII, alpha 1, isoform CRA_d OS=Homo sapiens OX=9606 GN=COL18A1 PE=4 SV=1;sp|P39060-2|C
drogenase OS=Homo sapiens OX=9606 GN=HEL-S-32 PE=2 SV=1;sp|P40925|MDHC_HUMAN Malate
in (Fragment) OS=Homo sapiens OX=9606 GN=IQGAP1 PE=2 SV=1;tr|A0A0J9YXZ5|A0A0J9YXZ5_HU
protein subunit beta OS=Homo sapiens OX=9606 GN=CAPZB PE=1 SV=1;tr|B1AK85|B1AK85_HUMA
e factor pathway inhibitor 2 (Fragment) OS=Homo sapiens OX=9606 PE=2 SV=1;sp|P48307-2|TFPI2_
OS=Homo sapiens OX=9606 GN=FASN PE=1 SV=3;tr|A0A0U1RQF0|A0A0U1RQF0_HUMAN 3-hydro
OS=Homo sapiens OX=9606 GN=VEGFC PE=2 SV=1;sp|P49767|VEGFC_HUMAN Vascular endothelia
ciation inhibitor (Fragment) OS=Homo sapiens OX=9606 GN=GDI2 PE=1 SV=1;sp|P50395-2|GDIB_HU
ion molecule OS=Homo sapiens OX=9606 GN=BCAM PE=1 SV=2;tr|A0A068W6H0|A0A068W6H0_HU
ation inhibitor 1 (Fragment) OS=Homo sapiens OX=9606 GN=ARHGDIA PE=1 SV=8;tr|V9HWE8|V9HW

lase 1 OS=Homo sapiens OX=9606 GN=CTSC PE=1 SV=2;tr|B4DJQ8|B4DJQ8_HUMAN Cathepsin C OS
Microfibrillar-associated protein 2 OS=Homo sapiens OX=9606 GN=MFAP2;sp|P55001-2|MFAP2_HU
sfer protein OS=Homo sapiens OX=9606 GN=PLTP PE=1 SV=1;tr|B3KUE5|B3KUE5_HUMAN Phospho
gment) OS=Homo sapiens OX=9606 GN=VCP PE=2 SV=2;tr|V9HW80|V9HW80_HUMAN 15S Mg(2+)-
astrocyte-derived neurotrophic factor OS=Homo sapiens OX=9606 GN=MANF PE=1 SV=3;tr|A8K878|

adherin-13 OS=Homo sapiens OX=9606 GN=CDH13;sp|P55290|CAD13_HUMAN Cadherin-13 OS=Ho
Homo sapiens OX=9606 GN=CD81 PE=1 SV=1;tr|E9PRJ8|E9PRJ8_HUMAN Tetraspanin (Fragment) OS
e isomerase OS=Homo sapiens OX=9606 PE=2 SV=1;tr|V9HWK1|V9HWK1_HUMAN Triosephosphate
unit alpha type OS=Homo sapiens OX=9606 GN=PSMA6 PE=1 SV=1;tr|G3V295|G3V295_HUMAN Pro
bulin OS=Homo sapiens OX=9606 GN=B2M PE=1 SV=1;tr|B4E0X1|B4E0X1_HUMAN Beta-2-microglo
cretory protein E1 (Fragment) OS=Homo sapiens OX=9606 PE=2 SV=1;tr|A0A024R6C0|A0A024R6C0_
gamma OS=Homo sapiens OX=9606 GN=YWHAG PE=1 SV=2;tr|B3KNB4|B3KNB4_HUMAN cDNA FLJ1
psilon OS=Homo sapiens OX=9606 GN=YWHAE PE=1 SV=1;tr|V9HW98|V9HW98_HUMAN Epididymi
OS=Homo sapiens OX=9606 GN=TMSB4X PE=2 SV=1;sp|P62328|TYB4_HUMAN Thymosin beta-4 OS

;=Homo sapiens OX=9606 GN=HIST1H4H PE=2 SV=1;tr|B2R4R0|B2R4R0_HUMAN Histone H4 OS=Hoı
-trans isomerase A OS=Homo sapiens OX=9606 GN=PPIA PE=1 SV=2;tr|A8K486|A8K486_HUMAN Pe
:is-trans isomerase FKBP1A OS=Homo sapiens OX=9606 GN=FKBP1A PE=1 SV=2;tr|Q5W0X3|Q5W0X
inal protein 4 OS=Homo sapiens OX=9606 GN=YWHAZ PE=2 SV=1;sp|P63104|1433Z_HUMAN 14-3-3
36, highly similar to Actin, cytoplasmic 2 OS=Homo sapiens OX=9606 PE=2 SV=1;tr|B4E3A4|B4E3A4_
10 OS=Homo sapiens OX=9606 GN=TMSB10 PE=1 SV=2;tr|D6W5K2|D6W5K2_HUMAN Thymosin, be
yrosine-protein phosphatase non-receptor type substrate 1 OS=Homo sapiens OX=9606 GN=SIRPA;s
OS=Homo sapiens OX=9606 GN=JAG1 PE=1 SV=3;tr|B2R6U9|B2R6U9_HUMAN Delta-like protein OS
inase-associated lipocalin OS=Homo sapiens OX=9606 GN=LCN2 PE=1 SV=2;tr|B2ZDQ1|B2ZDQ1_HUI
2 OS=Homo sapiens OX=9606 GN=NUCB2 PE=1 SV=2;sp|P80303-2|NUCB2_HUMAN Isoform 2 of Nuc
lomo sapiens OX=9606 GN=FBLN2 PE=2 SV=1;sp|P98095|FBLN2_HUMAN Fibulin-2 OS=Homo sapien

se-associated protein 1 (Fragment) OS=Homo sapiens OX=9606 GN=CAP1 PE=1 SV=1;tr|Q5T0R6|Q5T
. isoform HRG-beta2 OS=Homo sapiens OX=9606 GN=NRG1 PE=2 SV=1;tr|B0FYA7|B0FYA7_HUMAN I
-1(VII) chain OS=Homo sapiens OX=9606 GN=COL7A1 PE=1 SV=2;sp|Q02388-2|CO7A1_HUMAN Isofo
ysine 5-dioxygenase OS=Homo sapiens OX=9606 PE=2 SV=1;sp|Q02809|PLOD1_HUMAN Procollager
1 OS=Homo sapiens OX=9606 GN=NUCB1 PE=1 SV=4;tr|A8K7Q1|A8K7Q1_HUMAN Nucleobindin-1 O
yloid beta (A4)-like protein 2, isoform CRA_a OS=Homo sapiens OX=9606 GN=APLP2 PE=4 SV=1;tr|A(
didymis secretory sperm binding protein OS=Homo sapiens OX=9606 PE=2 SV=1;sp|Q06830|PRDX1_
ı-1(XVI) chain OS=Homo sapiens OX=9606 GN=COL16A1 PE=1 SV=1;sp|Q07092-2|COGA1_HUMAN Is
pithelial discoidin domain-containing receptor 1 OS=Homo sapiens OX=9606 GN=DDR1;sp|Q08345-2
:tin-3-binding protein (Fragment) OS=Homo sapiens OX=9606 GN=LGALS3BP PE=2 SV=1;sp|Q08380|
-acetylgalactosaminyltransferase OS=Homo sapiens OX=9606 GN=GALNT2 PE=3 SV=1;tr|B7Z6K2|B7Z
g fibulin-like extracellular matrix protein 1 isoform b variant (Fragment) OS=Homo sapiens OX=9606
:d protein 1 OS=Homo sapiens OX=9606 GN=FSTL1 PE=1 SV=1;sp|Q12841-2|FSTL1_HUMAN Isoform
Contactin-1 OS=Homo sapiens OX=9606 GN=CNTN1;tr|A0A024R104|A0A024R104_HUMAN Contacti
(Fragment) OS=Homo sapiens OX=9606 GN=SEMA3F PE=1 SV=8;tr|Q59G50|Q59G50_HUMAN Sema
yrosine kinase 7 isoform a variant (Fragment) OS=Homo sapiens OX=9606 PE=2 SV=1;sp|Q13308-5|I
agment) OS=Homo sapiens OX=9606 GN=MSLN PE=1 SV=8;sp|Q13421-4|MSLN_HUMAN Isoform 4 (
ıd metalloproteinase domain-containing protein 9 OS=Homo sapiens OX=9606 GN=ADAM9 PE=1 SV=
nin beta 3 isoform 1 (Fragment) OS=Homo sapiens OX=9606 GN=LAMB3 PE=2 SV=1;sp|Q13751|LAM
it gamma-2 OS=Homo sapiens OX=9606 GN=LAMC2 PE=1 SV=2;sp|Q13753-2|LAMC2_HUMAN Isofoı
pha-dystroglycan OS=Homo sapiens OX=9606 GN=DAG1 PE=4 SV=1;sp|Q14118|DAG1_HUMAN Dystı
!, highly similar to Growth-arrest-specific protein 6 OS=Homo sapiens OX=9606 PE=2 SV=1;sp|Q1439

rming growth factor beta-binding protein 2 OS=Homo sapiens OX=9606 GN=LTBP2 PE=1 SV=1;tr|G3\
'rotein disulfide-isomerase A6 OS=Homo sapiens OX=9606 GN=PDIA6;tr|A0A384NPU5|A0A384NPU5
ectin OS=Homo sapiens OX=9606 GN=PLEC;sp|Q15149-8|PLEC_HUMAN Isoform 8 of Plectin OS=Hoı
ptor-related 1 (Fragment) OS=Homo sapiens OX=9606 GN=PVRL1 PE=4 SV=1;sp|Q15223-3|NECT1_H
e phosphatase, receptor type, K variant (Fragment) OS=Homo sapiens OX=9606 PE=2 SV=1;tr|B4DH(
ısforming growth factor-beta-induced protein ig-h3 (Fragment) OS=Homo sapiens OX=9606 GN=TGFI
h factor-binding protein 7 OS=Homo sapiens OX=9606 GN=IGFBP7 PE=1 SV=1;sp|Q16270-2|IBP7_HU
ılar tissue protein Li 61 OS=Homo sapiens OX=9606 PE=2 SV=1;sp|Q16610|ECM1_HUMAN Extracellu
ıo sapiens OX=9606 PE=2 SV=1;tr|A0A384MEG1|A0A384MEG1_HUMAN Fascin OS=Homo sapiens O
ide cyclotransferase OS=Homo sapiens OX=9606 GN=QPCT PE=1 SV=1;sp|Q16769-2|QPCT_HUMAN
1811249, isoform CRA_e OS=Homo sapiens OX=9606 GN=LAMA3 PE=1 SV=1;sp|Q16787-1|LAMA3_I
e protein 132A OS=Homo sapiens OX=9606 GN=TMEM132A PE=1 SV=1;sp|Q24JP5-2|T132A_HUMAN
ɔmo sapiens OX=9606 GN=ANXA8L1 PE=1 SV=1;sp|Q5VT79|AXA81_HUMAN Annexin A8-like protein
protein 5 OS=Homo sapiens OX=9606 GN=RAET1G PE=1 SV=1;sp|Q6H3X3-2|ULBP5_HUMAN Isoform
Willebrand factor A domain containing 1, isoform CRA_c OS=Homo sapiens OX=9606 GN=VWA1 PE=

CD109 antigen OS=Homo sapiens OX=9606 GN=CD109;sp|Q6YHK3|CD109_HUMAN CD109 antigen (
2 fis, clone HLUNG2001507, highly similar to Amphoterin-induced protein 2 OS=Homo sapiens OX=96
olysis-stimulated lipoprotein receptor OS=Homo sapiens OX=9606 GN=LSR;sp|Q86X29-5|LSR_HUMA
ncer-binding protein 1 OS=Homo sapiens OX=9606 GN=AEBP1 PE=1 SV=1;tr|B4DJB3|B4DJB3_HUMA
Extracellular sulfatase Sulf-2 OS=Homo sapiens OX=9606 GN=SULF2;sp|Q8IWU5|SULF2_HUMAN Ext
vertase subtilisin/kexin type 9 OS=Homo sapiens OX=9606 GN=PCSK9 PE=1 SV=3;tr|A0A669KBG0|A(
, highly similar to Programmed cell death 6-interacting protein OS=Homo sapiens OX=9606 PE=2 SV=
C OS=Homo sapiens OX=9606 GN=FAM3C PE=1 SV=1;tr|C9JP35|C9JP35_HUMAN Protein FAM3C (Fra
nolog OS=Homo sapiens OX=9606 GN=PXDN PE=1 SV=2;tr|H7C1W1|H7C1W1_HUMAN Peroxidasin h
e HTRA1 OS=Homo sapiens OX=9606 GN=HTRA1 PE=1 SV=1;tr|Q05DJ8|Q05DJ8_HUMAN HTRA1 prot
f Neuronal cell adhesion molecule OS=Homo sapiens OX=9606 GN=NRCAM;sp|Q92823-3|NRCAM_HU
s protein 1 OS=Homo sapiens OX=9606 GN=GLG1 PE=1 SV=2;sp|Q92896-3|GSLG1_HUMAN Isoform 3
ide-3-kinase-interacting protein 1 (Fragment) OS=Homo sapiens OX=9606 GN=PIK3IP1 PE=1 SV=8;sp
lycase OS=Homo sapiens OX=9606 GN=HEL-S-67p PE=2 SV=1;sp|Q99497|PARK7_HUMAN Parkinson
lopeptidase OS=Homo sapiens OX=9606 PE=2 SV=1;tr|Q6I9U9|Q6I9U9_HUMAN Asparaginyl endope
Collagen alpha-1(XII) chain OS=Homo sapiens OX=9606 GN=COL12A1;tr|D6RGG3|D6RGG3_HUMAN
C OS=Homo sapiens OX=9606 GN=SEMA3C PE=2 SV=2;tr|B4E2I9|B4E2I9_HUMAN Semaphorin-3C OS
edoxin domain-containing protein 17 OS=Homo sapiens OX=9606 PE=2 SV=1;sp|Q9BRA2|TXD17_HU
45 kDa calcium-binding protein OS=Homo sapiens OX=9606 GN=SDF4;tr|G3V1E2|G3V1E2_HUMAN 4
Tubulointerstitial nephritis antigen-like OS=Homo sapiens OX=9606 GN=TINAGL1;sp|Q9GZM7|TINAL
nding glutamic acid-rich-like protein 3 OS=Homo sapiens OX=9606 GN=SH3BGRL3 PE=1 SV=1;tr|Q86Z
Semaphorin-4B OS=Homo sapiens OX=9606 GN=SEMA4B;sp|Q9NPR2|SEM4B_HUMAN Semaphorin
S=Homo sapiens OX=9606 GN=TENM2 PE=1 SV=1;sp|Q9NT68-2|TEN2_HUMAN Isoform 2 of Teneurin
ed protein CRIM1 (Fragment) OS=Homo sapiens OX=9606 GN=CRIM1 PE=4 SV=1;sp|Q9NZV1|CRIM1_
receptor negative regulator OS=Homo sapiens OX=9606 GN=PTGFRN PE=1 SV=2;tr|Q1WWL2|Q1W
d protein 3 OS=Homo sapiens OX=9606 GN=DKK3 PE=1 SV=2;tr|F6SYF8|F6SYF8_HUMAN Dickkopf-re
S=Homo sapiens OX=9606 PE=2 SV=1;sp|Q9UBR2|CATZ_HUMAN Cathepsin Z OS=Homo sapiens OX=
se A4 OS=Homo sapiens OX=9606 GN=CPA4 PE=1 SV=1;sp|Q9UI42-2|CBPA4_HUMAN Isoform 2 of Ca
f Collagen alpha-1(XVII) chain OS=Homo sapiens OX=9606 GN=COL17A1;tr|D3DRA2|D3DRA2_HUMA
tein C receptor OS=Homo sapiens OX=9606 GN=PROCR PE=1 SV=1;tr|A0A0U1RQQ4|A0A0U1RQQ4_H
krein-related peptidase 5 transcript variant 5 OS=Homo sapiens OX=9606 GN=KLK5 PE=2 SV=1;tr|A0
omolog OS=Homo sapiens OX=9606 GN=LOXL2 PE=2 SV=1;sp|Q9Y4K0|LOXL2_HUMAN Lysyl oxidase
tumorigenicity 14 protein homolog OS=Homo sapiens OX=9606 PE=2 SV=1;sp|Q9Y5Y6|ST14_HUMAN
nesion molecule 1 OS=Homo sapiens OX=9606 GN=F11R PE=3 SV=1;tr|Q6FIB4|Q6FIB4_HUMAN Junct
onectin OS=Homo sapiens OX=9606 GN=FN1 PE=4 SV=1;tr|B7ZLE5|B7ZLE5_HUMAN Fibronectin OS=
0 protein 9 (Fragment) OS=Homo sapiens OX=9606 GN=KLK10 PE=2 SV=1;tr|A0A024R4N7|A0A024R
ascin C (Hexabrachion), isoform CRA_a OS=Homo sapiens OX=9606 GN=TNC PE=3 SV=1;tr|Q4LE33|Q
aran sulfate proteoglycan 2 (Perlecan), isoform CRA_b OS=Homo sapiens OX=9606 GN=HSPG2 PE=4
olony-stimulating factor (Fragment) OS=Homo sapiens OX=9606 GN=CSF3 PE=2 SV=1;tr|A0A0B4U5E
class I antigen OS=Homo sapiens OX=9606 GN=HLA-C PE=3 SV=1;tr|A0A0S4T3H1|A0A0S4T3H1_HUN
0|A0A0S4XRM0_HUMAN;tr|A0A0U5PUM0|A0A0U5PUM0_HUMAN;tr|A0A6B9VT42|A0A6B9VT42_H

ronectin OS=Homo sapiens OX=9606 PE=2 SV=1;sp|P02751-10|FINC_HUMAN Isoform 10 of Fibrone
ubcomponent C1r OS=Homo sapiens OX=9606 GN=C1R PE=1 SV=3;tr|Q53HU9|Q53HU9_HUMAN Co
4, highly similar to Homo sapiens desmocollin type 4 OS=Homo sapiens OX=9606 PE=2 SV=1
A E1 OS=Homo sapiens OX=9606 GN=RNASE7 PE=3 SV=1;tr|A8K9I2|A8K9I2_HUMAN cDNA FLJ75277

, highly similar to Matrix metalloproteinase-9 (EC3.4.24.35) OS=Homo sapiens OX=9606 PE=2 SV=1;s

plasminogen activator OS=Homo sapiens OX=9606 GN=PLAU PE=1 SV=1;tr|Q5PY49|Q5PY49_HUMA
ntigen (Fragment) OS=Homo sapiens OX=9606 GN=HLA-C PE=3 SV=1;tr|F6IQL9|F6IQL9_HUMAN MH
elial growth factor A (Fragment) OS=Homo sapiens OX=9606 GN=VEGFA PE=1 SV=1;sp|P15692-2|VE
issue growth factor OS=Homo sapiens OX=9606 GN=CTGF PE=2 SV=1;tr|B3KRV6|B3KRV6_HUMAN c

MAN Keratin 14 (Epidermolysis bullosa simplex, Dowling-Meara, Koebner), isoform CRA_a OS=Homo
3|K2C6A_HUMAN Keratin, type II cytoskeletal 6A OS=Homo sapiens OX=9606 GN=KRT6A PE=1 SV=3;t

J54081, highly similar to Keratin, type II cytoskeletal 5 OS=Homo sapiens OX=9606 PE=2 SV=1;tr|B4D

ratin, type I cytoskeletal 17 OS=Homo sapiens OX=9606 GN=KRT17 PE=1 SV=2;;tr|Q14666|Q14666_I
MAN Uncharacterized protein DKFZp781M0386 OS=Homo sapiens OX=9606 GN=DKFZp781M0386 PE
AN Chloride intracellular channel protein OS=Homo sapiens OX=9606 GN=CLIC1 PE=2 SV=2;tr|Q53FB
fhydryl oxidase OS=Homo sapiens OX=9606 GN=QSCN6 PE=2 SV=1;sp|O00391-2|QSOX1_HUMAN Iso
sidase OS=Homo sapiens OX=9606 PE=2 SV=1;tr|A8K6D3|A8K6D3_HUMAN Beta-mannosidase OS=H
omo sapiens OX=9606 GN=AGRN;sp|O00468-3|AGRIN_HUMAN Isoform 3 of Agrin OS=Homo sapiens

OS=Homo sapiens OX=9606 GN=CCN1 PE=1 SV=1;tr|B4DI61|B4DI61_HUMAN cDNA FLJ58182, highly
V=1;tr|A0A024R5U5|A0A024R5U5_HUMAN Disintegrin and metalloproteinase domain-containing pr
A_HUMAN Myosin regulatory light chain 12A OS=Homo sapiens OX=9606 GN=MYL12A PE=1 SV=2;sp

nitz-type protease inhibitor 1 (Fragment) OS=Homo sapiens OX=9606 GN=SPINT1 PE=1 SV=1;tr|B4D
N4_HUMAN Alpha-actinin-4 OS=Homo sapiens OX=9606 GN=ACTN4 PE=1 SV=2;tr|Q96BG6|Q96BG6

stone H2B OS=Homo sapiens OX=9606 GN=HIST1H2BN PE=3 SV=1;tr|I6L9F7|I6L9F7_HUMAN Histone
1;tr|A0A024R141|A0A024R141_HUMAN Cathepsin L2, isoform CRA_a OS=Homo sapiens OX=9606 G
_HUMAN Isoform 2 of WD repeat-containing protein 1 OS=Homo sapiens OX=9606 GN=WDR1;tr|B3K
emaphorin-7A OS=Homo sapiens OX=9606 GN=SEMA7A;tr|F5GYX3|F5GYX3_HUMAN Semaphorin-7A
S=Homo sapiens OX=9606 GN=FLNB;sp|O75369|FLNB_HUMAN Filamin-B OS=Homo sapiens OX=960
apiens OX=9606 GN=SERPINB7 PE=1 SV=1;tr|A8K3Q8|A8K3Q8_HUMAN cDNA FLJ75069, highly simil
form 3 of Attractin OS=Homo sapiens OX=9606 GN=ATRN;sp|O75882-2|ATRN_HUMAN Isoform 2 of
1;tr|Q6FHC9|Q6FHC9_HUMAN STC2 protein (Fragment) OS=Homo sapiens OX=9606 GN=STC2 PE=2

V=1;sp|O95084|PRS23_HUMAN Serine protease 23 OS=Homo sapiens OX=9606 GN=PRSS23 PE=1 SV
2RBR3_HUMAN LY6/PLAUR domain containing 3 OS=Homo sapiens OX=9606 GN=LYPD3 PE=2 SV=1;t
MAN Follistatin-like 3 (Secreted glycoprotein), isoform CRA_a OS=Homo sapiens OX=9606 GN=FSTL3
ctate dehydrogenase A chain OS=Homo sapiens OX=9606 GN=LDHA PE=1 SV=2;sp|P00338-3|LDHA_H
artate aminotransferase, mitochondrial OS=Homo sapiens OX=9606 GN=GOT2;tr|A0A024R6W0|A0A
phoglycerate kinase OS=Homo sapiens OX=9606 GN=HEL-S-68p PE=2 SV=1;sp|P00558|PGK1_HUMA
minogen activator OS=Homo sapiens OX=9606 GN=PLAT PE=1 SV=1;tr|B4DNJ1|B4DNJ1_HUMAN T-pla
Homo sapiens OX=9606 GN=C3 PE=1 SV=2;tr|B4E216|B4E216_HUMAN C3-beta-c OS=Homo sapiens
etalloproteinase inhibitor 1 OS=Homo sapiens OX=9606 GN=TIMP1 PE=2 SV=1;tr|B3KQF4|B3KQF4_H

N Low density lipoprotein receptor (Fragment) OS=Homo sapiens OX=9606 GN=LDLR PE=4 SV=1;tr|J3
sapiens OX=9606 GN=INS PE=1 SV=1;tr|I3WAC9|I3WAC9_HUMAN Insulin OS=Homo sapiens OX=960
MAN Immunoglobulin kappa constant (Fragment) OS=Homo sapiens OX=9606 GN=IGKC PE=1 SV=1;tr
PE=2 SV=1;sp|P01876|IGHA1_HUMAN Immunoglobulin heavy constant alpha 1 OS=Homo sapiens O
Collagen alpha-1(IV) chain (Fragment) OS=Homo sapiens OX=9606 GN=COL4A1 PE=1 SV=1;tr|A0A6G9
is secretory sperm binding protein OS=Homo sapiens OX=9606 PE=2 SV=1;sp|P02545-6|LMNA_HUM
OE_HUMAN Apolipoprotein E OS=Homo sapiens OX=9606 GN=APOE PE=1 SV=1;tr|A0A346DBY2|A0A

Homo sapiens OX=9606 GN=FN1;sp|P02751-11|FINC_HUMAN Isoform 11 of Fibronectin OS=Homo s
ding protein OS=Homo sapiens OX=9606 GN=GC;tr|V9HWI6|V9HWI6_HUMAN Gc-globulin OS=Homo

UMAN Matrix metalloproteinase 1 preproprotein variant (Fragment) OS=Homo sapiens OX=9606 PE
JMAN Fructose-bisphosphate aldolase OS=Homo sapiens OX=9606 GN=HEL-S-87p PE=2 SV=1;tr|J3KP
iens OX=9606 GN=CSTB PE=1 SV=2;tr|A0A1W2PS52|A0A1W2PS52_HUMAN Cystatin-B OS=Homo sa
iens OX=9606 GN=ANXA1 PE=2 SV=1;tr|A0A4D5RAI5|A0A4D5RAI5_HUMAN Annexin OS=Homo sapi
otein (Fragment) OS=Homo sapiens OX=9606 GN=PRNP PE=3 SV=1;tr|Q6SES1|Q6SES1_HUMAN Maj
omo sapiens OX=9606 GN=KRT1 PE=3 SV=1;tr|H6VRF9|H6VRF9_HUMAN Cytokeratin-1 OS=Homo sa
_HUMAN Isoform 2 of Glyceraldehyde-3-phosphate dehydrogenase OS=Homo sapiens OX=9606 GN
OS=Homo sapiens OX=9606 GN=H2AC12 PE=1 SV=3;sp|P04908|H2A1B_HUMAN Histone H2A type
loid-beta precursor protein OS=Homo sapiens OX=9606 GN=APP PE=1 SV=3;sp|P05067-11|A4_HUM

32), mRNA OS=Homo sapiens OX=9606 PE=2 SV=1;sp|P05120|PAI2_HUMAN Plasminogen activator i
A_b OS=Homo sapiens OX=9606 GN=SERPINE1 PE=3 SV=1;sp|P05121|PAI1_HUMAN Plasminogen a
05155-2|IC1_HUMAN Isoform 2 of Plasma protease C1 inhibitor OS=Homo sapiens OX=9606 GN=SE
37|RLA2_HUMAN 60S acidic ribosomal protein P2 OS=Homo sapiens OX=9606 GN=RPLP2 PE=1 SV=1
ta-1 OS=Homo sapiens OX=9606 GN=ITGB1;sp|P05556-5|ITB1_HUMAN Isoform 5 of Integrin beta-1
sapiens OX=9606 GN=GSN;tr|A0A384MEF1|A0A384MEF1_HUMAN Actin-depolymerizing factor OS

100-A6 OS=Homo sapiens OX=9606 GN=S100A6 PE=1 SV=1;tr|B2R577|B2R577_HUMAN Protein S1
)-glycerate hydro-lyase OS=Homo sapiens OX=9606 GN=HEL-S-17 PE=2 SV=1;tr|A0A2R8Y6G6|A0A2
MAN Isoform 2 of Glucose-6-phosphate isomerase OS=Homo sapiens OX=9606 GN=GPI;tr|A0A1W6
N Isoform 2 of Nucleophosmin OS=Homo sapiens OX=9606 GN=NPM1;tr|A0A0S2Z491|A0A0S2Z491
HUMAN Tropomyosin alpha-3 chain OS=Homo sapiens OX=9606 GN=TPM3 PE=1 SV=1;sp|P06753-2
24R498_HUMAN Serpin peptidase inhibitor, clade E (Nexin, plasminogen activator inhibitor type 1),
-lactate dehydrogenase B chain OS=Homo sapiens OX=9606 GN=LDHB PE=1 SV=2;tr|Q5U077|Q5U0
Protein disulfide-isomerase OS=Homo sapiens OX=9606 GN=P4HB PE=2 SV=1;tr|H7BZ94|H7BZ94_
apiens OX=9606 GN=HEL-S-130P PE=2 SV=1;tr|A0A1B0GWE8|A0A1B0GWE8_HUMAN Cathepsin D O
Homo sapiens OX=9606 GN=ANXA2 PE=2 SV=1;tr|V9HW65|V9HW65_HUMAN Annexin OS=Homo sa
ens OX=9606 PE=2 SV=1;tr|A0A024QZQ2|A0A024QZQ2_HUMAN Prosaposin (Variant Gaucher disea
ne PLACE1002374, highly similar to Cathepsin L OS=Homo sapiens OX=9606 PE=2 SV=1;tr|A5PLM9|

=Homo sapiens OX=9606 GN=CTSB PE=2 SV=1;tr|B4DL49|B4DL49_HUMAN Cathepsin B OS=Homo sa
unit beta-1 OS=Homo sapiens OX=9606 GN=LAMB1 PE=1 SV=1;tr|Q8TAS6|Q8TAS6_HUMAN LAMB1

6 GN=HSP90AB1 PE=3 SV=1;sp|P08238|HS90B_HUMAN Heat shock protein HSP 90-beta OS=Homo
a-A chain OS=Homo sapiens OX=9606 GN=INHBA PE=2 SV=1;sp|P08476|INHBA_HUMAN Inhibin bet
72|CO4A2_HUMAN Collagen alpha-2(IV) chain OS=Homo sapiens OX=9606 GN=COL4A2 PE=1 SV=4;t
patocyte growth factor receptor OS=Homo sapiens OX=9606 GN=MET PE=1 SV=4;tr|A0A024R728|A
ns OX=9606 GN=VIM PE=1 SV=4;tr|B0YJC4|B0YJC4_HUMAN Vimentin OS=Homo sapiens OX=9606 G
apiens OX=9606 GN=ANXA5 PE=1 SV=2;tr|E9PHT9|E9PHT9_HUMAN Annexin OS=Homo sapiens OX
mo sapiens OX=9606 GN=HEL-S-22 PE=2 SV=1;sp|P09211|GSTP1_HUMAN Glutathione S-transferas

ens OX=9606 GN=SPARC PE=3 SV=1;tr|B2RDL6|B2RDL6_HUMAN Osteonectin OS=Homo sapiens OX
UMAN Complement C1s subcomponent OS=Homo sapiens OX=9606 GN=C1S PE=1 SV=1;tr|B3KNX0|
ribosomal protein S27a OS=Homo sapiens OX=9606 GN=RPS27A PE=1 SV=2;tr|F5GXK7|F5GXK7_HU
=Homo sapiens OX=9606 GN=SET PE=1 SV=1;sp|Q01105-4|SET_HUMAN Isoform 4 of Protein SET O

HUMAN Heat shock 70 kDa protein 1A OS=Homo sapiens OX=9606 GN=HSPA1A PE=1 SV=1;sp|P0DM
mo sapiens OX=9606 GN=CALM1 PE=1 SV=1;sp|P0DP24|CALM2_HUMAN Calmodulin-2 OS=Homo sa
PRF_HUMAN Receptor-type tyrosine-protein phosphatase F OS=Homo sapiens OX=9606 GN=PTPRF P
ns OX=9606 GN=TXN PE=2 SV=1;sp|P10599-2|THIO_HUMAN Isoform 2 of Thioredoxin OS=Homo sa
N 78 kDa glucose-regulated protein OS=Homo sapiens OX=9606 GN=HEL-S-89n PE=2 SV=1
;sp|P11047|LAMC1_HUMAN Laminin subunit gamma-1 OS=Homo sapiens OX=9606 GN=LAMC1 PE=
N Epididymis luminal protein 33 OS=Homo sapiens OX=9606 GN=HEL-S-72p PE=2 SV=1;tr|E9PKE3|E
5H7_HUMAN Collagen, type VI, alpha 1, isoform CRA_b OS=Homo sapiens OX=9606 GN=COL6A1 PE=
_HUMAN Alpha-actinin-1 OS=Homo sapiens OX=9606 GN=ACTN1 PE=1 SV=2;sp|P12814-3|ACTN1_H
1 OS=Homo sapiens OX=9606 GN=CDH1 PE=4 SV=1;tr|A0A0U2ZQU7|A0A0U2ZQU7_HUMAN E-cadh
HUMAN cDNA FLJ77316, highly similar to Homo sapiens interferon, gamma-inducible protein 30 (IFI3
_HUMAN cDNA FLJ56548, highly similar to Elongation factor 2 OS=Homo sapiens OX=9606 PE=2 SV=
MAN Protein disulfide-isomerase A4 OS=Homo sapiens OX=9606 GN=PDIA4 PE=1 SV=2;tr|A0A499FI
N Translationally-controlled tumor protein (Fragment) OS=Homo sapiens OX=9606 GN=TPT1 PE=1 S
N L-plastin variant (Fragment) OS=Homo sapiens OX=9606 PE=2 SV=1;tr|A0A024RDT4|A0A024RDT4
otein OS=Homo sapiens OX=9606 PE=1 SV=1;tr|A0A2U3TZL5|A0A2U3TZL5_HUMAN CD59 glycoprot
=1;tr|A2VCQ4|A2VCQ4_HUMAN PRKCSH protein (Fragment) OS=Homo sapiens OX=9606 GN=PRKCS
ns OX=9606 GN=NID1 PE=1 SV=3;tr|A0A384MR25|A0A384MR25_HUMAN Epididymis secretory spe
se OS=Homo sapiens OX=9606 GN=HEL-S-30 PE=2 SV=1;sp|P14618-2|KPYM_HUMAN Isoform M1 of
2_HUMAN Epididymis luminal protein 35 OS=Homo sapiens OX=9606 GN=HEL-S-125m PE=2 SV=1;s
SV=1;sp|P15151-3|PVR_HUMAN Isoform Gamma of Poliovirus receptor OS=Homo sapiens OX=960
ZAH9|B7ZAH9_HUMAN cDNA, FLJ79193, highly similar to Beta-1,4-galactosyltransferase 1 OS=Homo
dymis secretory protein Li 105 OS=Homo sapiens OX=9606 GN=HEL-S-105 PE=2 SV=1;tr|Q6NUR7|Q6

0VK57_HUMAN Metalloproteinase inhibitor 2 OS=Homo sapiens OX=9606 PE=2 SV=1;sp|P16035|TI
en OS=Homo sapiens OX=9606 GN=CD44;sp|P16070-5|CD44_HUMAN Isoform 5 of CD44 antigen OS
a (Fragment) OS=Homo sapiens OX=9606 PE=2 SV=1;sp|P16144-4|ITB4_HUMAN Isoform Beta-4D of

ulin OS=Homo sapiens OX=9606 GN=VCL PE=3 SV=1;sp|P18206-2|VINC_HUMAN Isoform 1 of Vincul
JMAN Phosphoglycerate mutase OS=Homo sapiens OX=9606 GN=PGAM1 PE=2 SV=1;tr|Q53G35|Q5
rm CRA_c OS=Homo sapiens OX=9606 GN=FST PE=4 SV=1;sp|P19883|FST_HUMAN Follistatin OS=Ho

aining protein OS=Homo sapiens OX=9606 PE=2 SV=1;tr|A0A384ME06|A0A384ME06_HUMAN Epidi
OX=9606 GN=FLNA PE=1 SV=4;sp|P21333-2|FLNA_HUMAN Isoform 2 of Filamin-A OS=Homo sapien
piens OX=9606 GN=MDK PE=1 SV=1;tr|E9PLM6|E9PLM6_HUMAN Midkine OS=Homo sapiens OX=9
X=9606 PE=2 SV=1;tr|A8K7E0|A8K7E0_HUMAN Biglycan OS=Homo sapiens OX=9606 PE=2 SV=1;tr|
Tetraspanin OS=Homo sapiens OX=9606 GN=CD9 PE=1 SV=1;tr|A0A2R8Y478|A0A2R8Y478_HUMAN
sapiens OX=9606 GN=CDH3 PE=1 SV=2;tr|B4DLF0|B4DLF0_HUMAN cDNA FLJ50795, highly similar
N Nucleoside diphosphate kinase OS=Homo sapiens OX=9606 GN=NME1-NME2 PE=1 SV=1;tr|Q6FHN
OS=Homo sapiens OX=9606 PE=2 SV=1;sp|P35321|SPR1A_HUMAN Cornifin-A OS=Homo sapiens O
24R1U8_HUMAN Insulin-like growth factor-binding protein 4 OS=Homo sapiens OX=9606 GN=IGFBP
piens OX=9606 GN=FBLN1;tr|B1AHL2|B1AHL2_HUMAN Fibulin-1 OS=Homo sapiens OX=9606 GN=F
Alpha-6X1A of Integrin alpha-6 OS=Homo sapiens OX=9606 GN=ITGA6;sp|P23229-5|ITA6_HUMAN I

on-muscle isoform (Fragment) OS=Homo sapiens OX=9606 GN=CFL1 PE=1 SV=8;sp|P23528|COF1_H
no sapiens OX=9606 GN=TNC;tr|F5H7V9|F5H7V9_HUMAN Tenascin OS=Homo sapiens OX=9606 GN

Proteasome subunit alpha type OS=Homo sapiens OX=9606 GN=PSMA3 PE=2 SV=1;tr|A0A140VK43
N Proteasome endopeptidase complex OS=Homo sapiens OX=9606 GN=PSMA4 PE=1 SV=1;tr|B2RDG
cDNA FLJ60385, highly similar to Integrin alpha-3 OS=Homo sapiens OX=9606 PE=2 SV=1;tr|A0A140

OS=Homo sapiens OX=9606 GN=HEL-S-99n PE=2 SV=1;sp|P27797|CALR_HUMAN Calreticulin OS=Ho

'roteasome subunit alpha type OS=Homo sapiens OX=9606 PE=2 SV=1;sp|P28066|PSA5_HUMAN Pr
to Granulins OS=Homo sapiens OX=9606 PE=2 SV=1;sp|P28799-2|GRN_HUMAN Isoform 2 of Progr

MAN Receptor protein-tyrosine kinase OS=Homo sapiens OX=9606 GN=EPHA2 PE=4 SV=1;sp|P29317
N Transketolase variant (Fragment) OS=Homo sapiens OX=9606 PE=2 SV=1;sp|P29401|TKT_HUMAN
'hosphatidylethanolamine-binding protein 1 OS=Homo sapiens OX=9606 GN=PEBP1 PE=1 SV=3;tr|V
isomerase OS=Homo sapiens OX=9606 PE=2 SV=1;tr|V9HVY3|V9HVY3_HUMAN Protein disulfide-iso

apiens OX=9606 GN=SDC4 PE=1 SV=2;tr|M1VE83|M1VE83_HUMAN Tyrosine-protein kinase recepto
=1;tr|H0YBG7|H0YBG7_HUMAN Heterogeneous nuclear ribonucleoprotein H (Fragment) OS=Homo s
N Epididymis secretory protein Li 1 OS=Homo sapiens OX=9606 GN=HEL-S-1 PE=2 SV=1;sp|P31946|

=Homo sapiens OX=9606 PE=2 SV=1;sp|P31949|S10AB_HUMAN Protein S100-A11 OS=Homo sapie
MAN Isoform 2 of Neural cell adhesion molecule L1 OS=Homo sapiens OX=9606 GN=L1CAM;tr|Q7Z3Z
iens OX=9606 GN=GPC1 PE=1 SV=2;tr|H7C410|H7C410_HUMAN Glypican-1 OS=Homo sapiens OX=9

X=9606 GN=FBN2 PE=1 SV=1;sp|P35556-2|FBN2_HUMAN Isoform 2 of Fibrillin-2 OS=Homo sapiens
V=1;sp|P35579|MYH9_HUMAN Myosin-9 OS=Homo sapiens OX=9606 GN=MYH9 PE=1 SV=4;tr|A0A
N=SERPINB5 PE=3 SV=1;sp|P36952|SPB5_HUMAN Serpin B5 OS=Homo sapiens OX=9606 GN=SERPI
Transgelin OS=Homo sapiens OX=9606 PE=2 SV=1;sp|P37802|TAGL2_HUMAN Transgelin-2 OS=Ho
OIA1_HUMAN Isoform 3 of Collagen alpha-1(XVIII) chain OS=Homo sapiens OX=9606 GN=COL18A1;t
dehydrogenase, cytoplasmic OS=Homo sapiens OX=9606 GN=MDH1 PE=1 SV=4;sp|P40925-3|MDHC
MAN Ras GTPase-activating-like protein IQGAP1 OS=Homo sapiens OX=9606 GN=IQGAP1 PE=1 SV=1;t
N F-actin-capping protein subunit beta OS=Homo sapiens OX=9606 GN=CAPZB PE=1 SV=1;tr|B2R7T
_HUMAN Isoform 2 of Tissue factor pathway inhibitor 2 OS=Homo sapiens OX=9606 GN=TFPI2;sp|P4
yacyl-[acyl-carrier-protein] dehydratase OS=Homo sapiens OX=9606 GN=FASN PE=1 SV=1

MAN Isoform 2 of Rab GDP dissociation inhibitor beta OS=Homo sapiens OX=9606 GN=GDI2;tr|Q6I
MAN Basal cell adhesion molecule (Lutheran blood group) OS=Homo sapiens OX=9606 GN=BCAM PE
/E8_HUMAN Epididymis secretory sperm binding protein Li 47e OS=Homo sapiens OX=9606 GN=HEL

MAN Isoform A of Microfibrillar-associated protein 2 OS=Homo sapiens OX=9606 GN=MFAP2;tr|A0
lipid transfer protein, isoform CRA_c OS=Homo sapiens OX=9606 GN=PLTP PE=2 SV=1;sp|P55058-4|
ATPase p97 subunit OS=Homo sapiens OX=9606 GN=HEL-S-70 PE=2 SV=1;sp|P55072|TERA_HUMAN
A8K878_HUMAN cDNA FLJ77177, highly similar to Homo sapiens arginine-rich, mutated in early stag

mo sapiens OX=9606 GN=CDH13 PE=1 SV=1;tr|B7Z9B1|B7Z9B1_HUMAN Cadherin-13 OS=Homo sa
=Homo sapiens OX=9606 GN=CD81 PE=1 SV=1;tr|H0YDL9|H0YDL9_HUMAN Tetraspanin (Fragment
isomerase OS=Homo sapiens OX=9606 GN=HEL-S-49 PE=2 SV=1;tr|Q53HE2|Q53HE2_HUMAN Trios
teasome subunit alpha type OS=Homo sapiens OX=9606 GN=PSMA6 PE=1 SV=1;tr|A0A140VK44|A0
bulin OS=Homo sapiens OX=9606 PE=2 SV=1;tr|A6XMH4|A6XMH4_HUMAN Beta-2-microglobulin O
_HUMAN Epididymal secretory protein E1 OS=Homo sapiens OX=9606 GN=NPC2 PE=2 SV=1;sp|P61
4168 fis, clone NT2RP2001440, highly similar to 14-3-3 protein gamma OS=Homo sapiens OX=9606 P
s luminal protein 2 OS=Homo sapiens OX=9606 GN=HEL2 PE=2 SV=1;tr|G9K389|G9K389_HUMAN Y
=Homo sapiens OX=9606 GN=TMSB4X PE=1 SV=2;tr|Q0P5T0|Q0P5T0_HUMAN TMSB4X protein (Fr

no sapiens OX=9606 GN=HIST1H4J PE=2 SV=1;sp|P62805|H4_HUMAN Histone H4 OS=Homo sapien
ptidyl-prolyl cis-trans isomerase OS=Homo sapiens OX=9606 PE=2 SV=1;tr|V9HWF5|V9HWF5_HUM
3_HUMAN Peptidylprolyl isomerase OS=Homo sapiens OX=9606 GN=FKBP1A PE=1 SV=1;tr|A0A087V
protein zeta/delta OS=Homo sapiens OX=9606 GN=YWHAZ PE=1 SV=1;tr|E7EX29|E7EX29_HUMAN
HUMAN cDNA FLJ57283, highly similar to Actin, cytoplasmic 2 OS=Homo sapiens OX=9606 PE=2 SV=

p|P78324|SHPS1_HUMAN Tyrosine-protein phosphatase non-receptor type substrate 1 OS=Homo s
=Homo sapiens OX=9606 PE=2 SV=1;tr|Q4KMR2|Q4KMR2_HUMAN Delta-like protein OS=Homo sap
MAN Neutrophil gelatinase-associated lipocalin OS=Homo sapiens OX=9606 GN=NGAL PE=2 SV=1;tr|
leobindin-2 OS=Homo sapiens OX=9606 GN=NUCB2;sp|P80303|NUCB2_HUMAN Nucleobindin-2 OS
s OX=9606 GN=FBLN2 PE=1 SV=2;tr|B7Z9B8|B7Z9B8_HUMAN cDNA FLJ56912, highly similar to Fibu

T0R6_HUMAN Adenylyl cyclase-associated protein 1 (Fragment) OS=Homo sapiens OX=9606 GN=CA
Heregulin-gamma protein isoform 1 (Fragment) OS=Homo sapiens OX=9606 GN=NRG1 PE=2 SV=1;sp

n-lysine,2-oxoglutarate 5-dioxygenase 1 OS=Homo sapiens OX=9606 GN=PLOD1 PE=1 SV=2;sp|Q028
S=Homo sapiens OX=9606 PE=2 SV=1;tr|Q53GX6|Q53GX6_HUMAN Nucleobindin-1 (Fragment) OS=
A140VJE9|A0A140VJE9_HUMAN Testicular tissue protein Li 23 OS=Homo sapiens OX=9606 PE=2 SV
_HUMAN Peroxiredoxin-1 OS=Homo sapiens OX=9606 GN=PRDX1 PE=1 SV=1;tr|A0A0A0MSI0|A0A0
oform 2 of Collagen alpha-1(XVI) chain OS=Homo sapiens OX=9606 GN=COL16A1;sp|Q07092|COGA
|DDR1_HUMAN Isoform 2 of Epithelial discoidin domain-containing receptor 1 OS=Homo sapiens O
LG3BP_HUMAN Galectin-3-binding protein OS=Homo sapiens OX=9606 GN=LGALS3BP PE=1 SV=1;tr|
6K2_HUMAN Polypeptide N-acetylgalactosaminyltransferase OS=Homo sapiens OX=9606 PE=2 SV=1
PE=4 SV=1;sp|Q12805-2|FBLN3_HUMAN Isoform 2 of EGF-containing fibulin-like extracellular matri
2 of Follistatin-related protein 1 OS=Homo sapiens OX=9606 GN=FSTL1;tr|B4DTZ8|B4DTZ8_HUMAN
in 1, isoform CRA_a OS=Homo sapiens OX=9606 GN=CNTN1 PE=3 SV=1;sp|Q12860|CNTN1_HUMAN
phorin 3F variant (Fragment) OS=Homo sapiens OX=9606 PE=2 SV=1;sp|Q13275-2|SEM3F_HUMAN
TK7_HUMAN Isoform 5 of Inactive tyrosine-protein kinase 7 OS=Homo sapiens OX=9606 GN=PTK7;s
of Mesothelin OS=Homo sapiens OX=9606 GN=MSLN;sp|Q13421-3|MSLN_HUMAN Isoform 2 of Me
1;sp|Q13443-2|ADAM9_HUMAN Isoform 2 of Disintegrin and metalloproteinase domain-containing

rm Short of Laminin subunit gamma-2 OS=Homo sapiens OX=9606 GN=LAMC2;tr|B4DLD1|B4DLD1_

3-3|GAS6_HUMAN Isoform 3 of Growth arrest-specific protein 6 OS=Homo sapiens OX=9606 GN=G

/3X5|G3V3X5_HUMAN Latent-transforming growth factor beta-binding protein 2 OS=Homo sapiens
_HUMAN Epididymis secretory sperm binding protein OS=Homo sapiens OX=9606 PE=2 SV=1;sp|Q1
no sapiens OX=9606 GN=PLEC;sp|Q15149-9|PLEC_HUMAN Isoform 9 of Plectin OS=Homo sapiens O
IUMAN Isoform Gamma of Nectin-1 OS=Homo sapiens OX=9606 GN=NECTIN1;sp|Q15223-2|NECT1_
3|B4DHC3_HUMAN cDNA FLJ61254, highly similar to Receptor-type tyrosine-protein phosphatase k
I PE=2 SV=1;sp|Q15582|BGH3_HUMAN Transforming growth factor-beta-induced protein ig-h3 OS=
JMAN Isoform 2 of Insulin-like growth factor-binding protein 7 OS=Homo sapiens OX=9606 GN=IGFB
ular matrix protein 1 OS=Homo sapiens OX=9606 GN=ECM1 PE=1 SV=2;sp|Q16610-4|ECM1_HUMAN
X=9606 PE=2 SV=1;sp|Q16658|FSCN1_HUMAN Fascin OS=Homo sapiens OX=9606 GN=FSCN1 PE=1
Isoform 2 of Glutaminyl-peptide cyclotransferase OS=Homo sapiens OX=9606 GN=QPCT;tr|B5MCZ9|
HUMAN Isoform 1 of Laminin subunit alpha-3 OS=Homo sapiens OX=9606 GN=LAMA3;sp|Q16787|L
Isoform 2 of Transmembrane protein 132A OS=Homo sapiens OX=9606 GN=TMEM132A;sp|Q24JP5
1 OS=Homo sapiens OX=9606 GN=ANXA8L1 PE=2 SV=2;tr|A0A075B752|A0A075B752_HUMAN Ann
2 of UL-16 binding protein 5 OS=Homo sapiens OX=9606 GN=RAET1G;sp|Q5VY80|ULBP6_HUMAN
4 SV=1;sp|Q6PCB0|VWA1_HUMAN von Willebrand factor A domain-containing protein 1 OS=Homo

)S=Homo sapiens OX=9606 GN=CD109 PE=1 SV=2;sp|Q6YHK3-2|CD109_HUMAN Isoform 2 of CD10
606 PE=2 SV=1;tr|A0A024R127|A0A024R127_HUMAN Adhesion molecule with Ig-like domain 2, isof
N Isoform 5 of Lipolysis-stimulated lipoprotein receptor OS=Homo sapiens OX=9606 GN=LSR;tr|S4R
N cDNA FLJ55979, highly similar to Homo sapiens AE binding protein 1 (AEBP1), mRNA OS=Homo sa

0A669KBG0_HUMAN Proprotein convertase 9 OS=Homo sapiens OX=9606 GN=PCSK9 PE=1 SV=1
1;tr|B4DHD2|B4DHD2_HUMAN cDNA FLJ55458, highly similar to Programmed cell death 6-interacti
gment) OS=Homo sapiens OX=9606 GN=FAM3C PE=1 SV=1;tr|C9JMN4|C9JMN4_HUMAN Protein F
omolog (Fragment) OS=Homo sapiens OX=9606 GN=PXDN PE=1 SV=1;sp|Q92626-2|PXDN_HUMAN
ein (Fragment) OS=Homo sapiens OX=9606 GN=HTRA1 PE=2 SV=1;tr|B3KRN4|B3KRN4_HUMAN cD
UMAN Isoform 3 of Neuronal cell adhesion molecule OS=Homo sapiens OX=9606 GN=NRCAM;sp|Q9
3 of Golgi apparatus protein 1 OS=Homo sapiens OX=9606 GN=GLG1;sp|Q92896-2|GSLG1_HUMAN I
|Q96FE7-4|P3IP1_HUMAN Isoform 4 of Phosphoinositide-3-kinase-interacting protein 1 OS=Homo s
disease protein 7 OS=Homo sapiens OX=9606 GN=PARK7 PE=1 SV=2;tr|K7ELW0|K7ELW0_HUMAN
ptidase OS=Homo sapiens OX=9606 GN=LGMN PE=2 SV=1;tr|Q53XC6|Q53XC6_HUMAN Asparaginyl
Collagen alpha-1(XII) chain OS=Homo sapiens OX=9606 GN=COL12A1 PE=1 SV=1;sp|Q99715|COCA1

MAN Thioredoxin domain-containing protein 17 OS=Homo sapiens OX=9606 GN=TXNDC17 PE=1 SV
45 kDa calcium-binding protein OS=Homo sapiens OX=9606 GN=SDF4 PE=1 SV=1;sp|Q9BRK5-3|CAB4
_HUMAN Tubulointerstitial nephritis antigen-like OS=Homo sapiens OX=9606 GN=TINAGL1 PE=1 SV
Z22|Q86Z22_HUMAN Epididymis secretory protein Li 297 OS=Homo sapiens OX=9606 GN=HEL-S-297
-4B OS=Homo sapiens OX=9606 GN=SEMA4B PE=1 SV=4;tr|H0YMZ3|H0YMZ3_HUMAN Semaphorin-
1-2 OS=Homo sapiens OX=9606 GN=TENM2;tr|G3V106|G3V106_HUMAN HCG38403, isoform CRA_
_HUMAN Cysteine-rich motor neuron 1 protein OS=Homo sapiens OX=9606 GN=CRIM1 PE=1 SV=1
WL2_HUMAN PTGFRN protein (Fragment) OS=Homo sapiens OX=9606 GN=PTGFRN PE=2 SV=1;tr|Q4
lated protein 3 OS=Homo sapiens OX=9606 GN=DKK3 PE=1 SV=1;tr|O43532|O43532_HUMAN RIG-li

arboxypeptidase A4 OS=Homo sapiens OX=9606 GN=CPA4;tr|A4D1M3|A4D1M3_HUMAN Carboxyp
N Collagen, type XVII, alpha 1, isoform CRA_b OS=Homo sapiens OX=9606 GN=COL17A1 PE=2 SV=1;
HUMAN Endothelial protein C receptor (Fragment) OS=Homo sapiens OX=9606 GN=PROCR PE=1 SV=
A2I2MP49|A0A2I2MP49_HUMAN Kallikrein-related peptidase 5 transcript variant 6 OS=Homo sapie
homolog 2 OS=Homo sapiens OX=9606 GN=LOXL2 PE=1 SV=1;tr|W8QRJ0|W8QRJ0_HUMAN Lysyl o
Suppressor of tumorigenicity 14 protein OS=Homo sapiens OX=9606 GN=ST14 PE=1 SV=2;tr|Q8W
tional adhesion molecule 1 OS=Homo sapiens OX=9606 GN=F11R PE=2 SV=1;sp|Q9Y624|JAM1_HU

4N7_HUMAN Kallikrein 10, isoform CRA_a OS=Homo sapiens OX=9606 GN=KLK10 PE=4 SV=1;tr|W8
4LE33_HUMAN TNC variant protein (Fragment) OS=Homo sapiens OX=9606 GN=TNC variant protein
SV=1;sp|P98160|PGBM_HUMAN Basement membrane-specific heparan sulfate proteoglycan core p
3|A0A0B4U5E3_HUMAN Granulocyte colony-stimulating factor (Fragment) OS=Homo sapiens OX=9
MAN MHC class I antigen OS=Homo sapiens OX=9606 GN=HLA-C PE=3 SV=1;tr|A0A5H2UZM2|A0A5H
HUMAN;tr|E5FQ47|E5FQ47_HUMAN;tr|A0A0F7RPE2|A0A0F7RPE2_HUMAN;tr|D1MYY9|D1MYY9_

mplement subcomponent C1r (Fragment) OS=Homo sapiens OX=9606 PE=2 SV=1;tr|Q53HT9|Q53H

, highly similar to Homo sapiens ribonuclease, RNase A family, 7 (RNASE7), mRNA OS=Homo sapiens

p|P14780|MMP9_HUMAN Matrix metalloproteinase-9 OS=Homo sapiens OX=9606 GN=MMP9 PE=1

.N Urokinase-type plasminogen activator (Fragment) OS=Homo sapiens OX=9606 PE=2 SV=1;sp|P007
C class I antigen (Fragment) OS=Homo sapiens OX=9606 GN=HLA-C PE=3 SV=1;tr|B4DVY0|B4DVY0_H
:GFA_HUMAN Isoform VEGF189 of Vascular endothelial growth factor A OS=Homo sapiens OX=9606
DNA FLJ34961 fis, clone NTONG2003839, highly similar to CONNECTIVE TISSUE GROWTH FACTOR OS

HUMAN;tr|A0A5H2UFF7|A0A5H2UFF7_HUMAN;tr|A0A3S6RFC0|A0A3S6RFC0_HUMAN;tr|A0A6B7H

GP6|A0A6B7HGP6_HUMAN;tr|A0A6B9VTX5|A0A6B9VTX5_HUMAN;tr|A0A5H2UF70|A0A5H2UF70_

_HUMAN;tr|A0A4Y5P8Y7|A0A4Y5P8Y7_HUMAN;tr|A0A4P2SQZ3|A0A4P2SQZ3_HUMAN;tr|A0A4P2

SQQ2|A0A4P2SQQ2_HUMAN;tr|A0A3S6RIB1|A0A3S6RIB1_HUMAN;tr|A0A6B7HGP2|A0A6B7HGP2_

_HUMAN;tr|A0A583ZAW1|A0A583ZAW1_HUMAN;tr|A0A3G2Y4K4|A0A3G2Y4K4_HUMAN;tr|A0A6I

B9VR96|A0A6B9VR96_HUMAN;tr|A0A6B7FTB7|A0A6B7FTB7_HUMAN;tr|A0A223FL72|A0A223FL72

2_HUMAN;tr|A0A6C0SE86|A0A6C0SE86_HUMAN;tr|A0A6C0SEA1|A0A6C0SEA1_HUMAN;tr|B1PKY3

|B1PKY3_HUMAN;tr|A0A4Y5ULL4|A0A4Y5ULL4_HUMAN;tr|A0A4P2STA1|A0A4P2STA1_HUMAN;tr

|A0A4P2ST16|A0A4P2ST16_HUMAN;tr|A0A6B7HHR8|A0A6B7HHR8_HUMAN;tr|A0A3S6REZ3|A0A3

S6REZ3_HUMAN;tr|A0A2P9FIT1|A0A2P9FIT1_HUMAN;tr|A0A2U3QLE8|A0A2U3QLE8_HUMAN;tr|A

A0A2U3QLG4|A0A2U3QLG4_HUMAN;tr|A0A2U3QLL1|A0A2U3QLL

LFQ intens	LFQ intens	LFQ intens	LFQ intens	LFQ intens	LFQ intens	LFQ intens	LFQ intens	LFQ intens
31,03118	32,02697	32,30549	28,31095	28,08035	29,06669	28,91336	20,79574	24,09746
24,83543	25,70187	25,88129	28,09989	27,96157	27,57521	27,98994	28,46045	28,40068
23,09327	22,72077	24,42926	29,01081	29,04226	29,36077	29,08563	28,76284	29,60442
30,29351	30,37098	30,32034	29,59586	29,41542	29,22338	29,18959	28,92872	28,58043
22,48696	23,48598	22,96295	25,59269	25,5305	25,10185	25,35279	24,66536	25,05383
23,67721	22,78137	23,41876	26,10536	26,18569	25,36903	25,84886	23,16877	21,86169
22,7308	23,63123	24,27583	26,40868	26,53424	27,85412	27,98462	27,96505	27,55311
27,87473	26,84828	26,64403	24,01014	22,0047	23,69696	22,98111	23,15847	24,53848
32,43964	31,66072	31,17665	34,3535	34,38985	33,92344	33,84763	34,53907	34,4746
24,86815	25,55318	24,44979	26,60399	26,9215	27,41828	26,91969	25,86976	26,79687
24,52641	24,32745	22,21216	29,76836	29,95754	30,88103	31,05601	30,82533	29,67459

LFQ intens	LFQ intens	Potential c	Student's T	Student's T	Peptides	Razor + un	Unique pe	Sequence c
22,47785	30,51427	+	+	1_2	29	17	3	46,1
27,99145	27,2674		+	1_2	26	26	24	21,6
29,71363	21,02804		+	1_2	6	6	3	21,1
28,83533	29,82773		+	1_2	11	10	10	39,3
24,57306	24,00873		+	1_2	5	5	5	15
24,02359	22,4964		+	1_2	2	2	2	21,4
27,3946	27,05765		+	1_2	13	13	13	5,9
23,00949	22,52667		+	1_2	3	3	3	9,7
34,5739	34,16121		+	1_2	22	22	22	49,7
26,64072	25,56842		+	1_2	6	6	6	34,8
29,61569	29,87948		+	1_2	18	18	17	36,4

Unique + r	Unique sec	Mol. weigh	Q-value	Score	Intensity	MS/MS co	iBAQ 1-1	iBAQ 1-3
31,7	6,7	58,536	0	132,71	9,64E+09	86	53050000	60895000
21,6	20,5	187,15	0	220,13	1,57E+09	99	227840	107200
21,1	12,1	28,955	0	12,282	3,05E+09	20	2358100	0
35,8	35,8	37,294	0	196,48	6,44E+09	90	1,13E+08	60047000
15	15	57,519	0	7,4178	1,92E+08	16	0	0
21,4	21,4	12,269	0	4,7231	1,89E+08	9	0	0
5,9	5,9	337,08	0	41,513	1,03E+09	34	237820	160850
9,7	9,7	51,853	0	5,2514	3,99E+08	5	9752800	3496400
49,7	49,7	65,33	0	323,31	1,23E+11	243	2E+08	59886000
34,8	34,8	24,093	0	11,912	6,03E+08	32	722330	1656600
36,4	35	76,613	0	102,9	6,63E+09	108	572730	319880

iBAQ 1-5	iBAQ 2-1	iBAQ 2-2	iBAQ 2-3	iBAQ 2-4	iBAQ 3-1	iBAQ 3-2	iBAQ 3-3	iBAQ 3-4
97959000	7555900	4620300	20505000	16035000	28207	21636	24610	71577000
403000	2500100	1334000	1378500	1308900	3850300	1884600	2576400	658530
0	20937000	13857000	32459000	26807000	26709000	25082000	22270000	9153000
47984000	33719000	11325000	13995000	28474000	15008000	19226000	12486000	23964000
0	1918000	1511600	552490	640380	1071300	1375000	792110	150430
1063800	9016000	7426100	3955500	7096900	2074800	517490	0	362010
114580	511800	515550	841040	596960	1513900	1613200	382330	370260
3898700	426320	0	239380	520440	662200	0	0	0
37044000	6,17E+08	6,56E+08	3,42E+08	3,78E+08	8,27E+08	5,07E+08	6,61E+08	4,61E+08
2198700	9511400	9987700	12240000	5983300	440490	4866300	4471200	2736900
32887	11261000	14385000	31679000	46276000	30816000	12062000	21324000	10422000

Student's T	Student's T	Student's T	Student's T	Majority pı	Fasta headers
0,000678	0,0104	3,195041	6,05605	tr|B4DRU6	tr|B4DRU6|B4DRU6_HUMAN cDNA FLJ5465
0,000478	0,008667	-2,43379	-6,05135	tr|V9HWA	tr|V9HWA9|V9HWA9_HUMAN C3-beta-c OS
5,07E-05	0	-5,71043	-10,4934	sp|P06753	sp|P06753-3|TPM3_HUMAN Isoform 3 of Tr
0,00035	0,041455	0,972214	4,564527	tr|A0A5F9	tr|A0A5F9ZHM4|A0A5F9ZHM4_HUMAN L-la
0,000313	0,017333	-2,41583	-6,45563	tr|F8WCZ6	tr|F8WCZ6|F8WCZ6_HUMAN Complement (
0,000415	0,013	-2,58479	-6,2821	sp|P19957	sp|P19957|ELAF_HUMAN Elafin OS=Homo s
0,002042	0,030222	-3,64947	-5,0539	sp|P25391	sp|P25391|LAMA1_HUMAN Laminin subuni
0,001365	0,016571	3,949118	5,519495	sp|P53634	sp|P53634|CATC_HUMAN Dipeptidyl peptid
0,001073	0,0275	-2,3696	-5,26204	tr|A0A0S2	tr|A0A0S2Z3Y1|A0A0S2Z3Y1_HUMAN Galec
0,001844	0,0324	-2,00883	-4,62096	tr|W8GRM	tr|W8GRM7|W8GRM7_HUMAN Kallikrein 1
0,00025	0	-6,72706	-8,12234	tr|F5H2D0	tr|F5H2D0|F5H2D0_HUMAN Complement s

;7, highly similar to Keratin, type II cytoskeletal 6A OS=Homo sapiens OX=9606 PE=2 SV=1;sp|P02538
S=Homo sapiens OX=9606 GN=HEL-S-62p PE=2 SV=1;sp|P01024|CO3_HUMAN Complement C3 OS=H
ropomyosin alpha-3 chain OS=Homo sapiens OX=9606 GN=TPM3;tr|A0A087WWU8|A0A087WWU8_
actate dehydrogenase OS=Homo sapiens OX=9606 GN=LDHB PE=1 SV=1;sp|P07195|LDHB_HUMAN L
C1s subcomponent OS=Homo sapiens OX=9606 GN=C1S PE=1 SV=1;tr|A0A087X232|A0A087X232_H

lase 1 OS=Homo sapiens OX=9606 GN=CTSC PE=1 SV=2;tr|B4DJQ8|B4DJQ8_HUMAN Cathepsin C OS
:tin-3-binding protein (Fragment) OS=Homo sapiens OX=9606 GN=LGALS3BP PE=2 SV=1;sp|Q08380|
0 protein 9 (Fragment) OS=Homo sapiens OX=9606 GN=KLK10 PE=2 SV=1;tr|A0A024R4N7|A0A024R
ubcomponent C1r OS=Homo sapiens OX=9606 GN=C1R PE=1 SV=3;tr|Q53HU9|Q53HU9_HUMAN Cc

3|K2C6A_HUMAN Keratin, type II cytoskeletal 6A OS=Homo sapiens OX=9606 GN=KRT6A PE=1 SV=3;t
Homo sapiens OX=9606 GN=C3 PE=1 SV=2;tr|B4E216|B4E216_HUMAN C3-beta-c OS=Homo sapiens
_HUMAN Tropomyosin alpha-3 chain OS=Homo sapiens OX=9606 GN=TPM3 PE=1 SV=1;sp|P06753-2
L-lactate dehydrogenase B chain OS=Homo sapiens OX=9606 GN=LDHB PE=1 SV=2;tr|Q5U077|Q5U0
UMAN Complement C1s subcomponent OS=Homo sapiens OX=9606 GN=C1S PE=1 SV=1;tr|B3KNX0|

LG3BP_HUMAN Galectin-3-binding protein OS=Homo sapiens OX=9606 GN=LGALS3BP PE=1 SV=1;tr|
4N7_HUMAN Kallikrein 10, isoform CRA_a OS=Homo sapiens OX=9606 GN=KLK10 PE=4 SV=1;tr|W8C
omplement subcomponent C1r (Fragment) OS=Homo sapiens OX=9606 PE=2 SV=1;tr|Q53HT9|Q53HT

|TPM3_HUMAN Isoform 2 of Tropomyosin alpha-3 chain O
77_HUMAN L-lactate dehydrogenase OS=Homo sapiens O
B3KNX0_HUMAN cDNA FLJ30621 fis, clone CTONG2001681, hi

T9_HUMAN Complement subcomponent C1r (Fragment) OS=Hom

[illegible]

LFQ intens	LFQ intens	LFQ intens	LFQ intens	LFQ intens	LFQ intens	LFQ intens	LFQ intens	LFQ intens
31,03118	32,02697	32,30549	28,31095	28,08035	29,06669	28,91336	20,79574	24,09746
24,49698	28,37501	30,17415	21,70664	24,67246	22,18999	22,98011	22,38854	22,66983
33,83921	33,80713	34,3397	31,56883	31,73376	32,68712	32,62625	29,26591	27,52992
21,99236	23,77998	23,67256	20,3393	20,89156	21,14923	20,90991	20,98905	21,36959
22,19592	22,21376	21,9892	22,62654	23,66371	22,6054	22,51538	23,07883	23,2337
33,43204	33,12132	33,52426	34,14444	34,1734	33,66034	33,77686	34,1496	34,19638
33,44233	33,07312	33,15658	32,61617	32,74256	33,55408	33,48424	32,51371	32,86356
24,54144	24,05358	24,24405	26,21647	26,88285	23,95601	23,44199	25,95476	26,61077
27,09295	26,39926	26,58606	28,29824	28,05274	26,49989	26,80193	28,56384	28,69026
28,65492	30,31646	29,05628	27,62944	27,36328	25,98713	27,53282	24,71282	25,02129
22,87401	22,50927	23,7104	24,00533	25,3654	23,46208	26,69856	27,58857	27,57975
24,83543	25,70187	25,88129	28,09989	27,96157	27,57521	27,98994	28,46045	28,40068
30,49037	30,49486	30,10011	30,22458	30,02314	29,36208	29,68611	29,85046	29,70337
30,69594	30,59067	30,49295	30,91663	30,91748	30,831	30,68361	31,33527	31,499
34,12325	32,30138	33,52869	33,55225	33,47255	32,45425	32,37485	31,58352	31,52687
25,75524	24,58153	26,69803	29,68005	28,98682	23,46787	24,53582	27,83545	27,79228
30,29351	30,37098	30,32034	29,59586	29,41542	29,22338	29,18959	28,92872	28,58043
28,33769	28,78279	28,03837	26,8832	27,00131	27,44979	27,41224	27,20602	27,0276
31,38469	31,11873	31,19578	31,53723	31,60116	31,29576	31,11471	32,09704	32,07728
28,95074	29,18386	29,2963	28,62383	28,84107	29,17331	29,1775	28,1155	28,13951
25,5526	23,09792	24,06357	27,97301	27,77335	26,13055	25,72093	27,91296	27,27293
22,48696	23,48598	22,96295	25,59269	25,5305	25,10185	25,35279	24,66536	25,05383
27,49579	26,13406	25,21182	26,31403	26,73376	27,57087	27,8052	28,65363	28,81427
24,22059	25,35528	25,29018	24,60156	24,26556	24,5428	24,76157	24,10556	22,01563
27,54399	25,67411	26,14766	27,58628	27,5059	27,32174	27,3808	28,95366	29,51783
22,40098	23,81693	23,63272	26,63145	26,90392	27,20909	22,62445	26,7656	26,85222
22,22519	24,05714	23,08479	20,84115	20,80986	20,17731	20,20192	21,1201	21,69238
25,24107	23,69519	23,87995	25,01504	24,50355	23,90812	24,18526	26,31443	26,06079
27,68759	27,50385	28,24354	28,02189	26,97913	26,97978	27,68746	26,87467	26,90323
27,75513	28,21183	27,7604	26,90529	26,82905	27,53646	26,94905	26,56261	26,16398
29,76917	28,95427	28,57236	29,82902	30,14722	29,61994	30,10762	31,08759	31,39622
22,7308	23,63123	24,27583	26,40868	26,53424	27,85412	27,98462	27,96505	27,55311
26,73104	25,19689	24,38988	22,31128	25,25555	24,68581	23,04007	23,24094	22,1104
23,23996	24,51734	23,0489	24,38555	22,96906	23,09225	23,3615	21,63014	21,64979
28,62048	28,59463	28,16322	28,52099	28,36035	28,34617	28,57015	27,47212	27,3232
30,49895	30,54563	31,03636	31,73884	31,96558	31,36124	31,4488	31,45983	31,59072
23,25643	23,04572	22,66345	23,46148	22,90094	23,43582	23,54321	24,35057	24,77258
30,53399	31,64255	31,45988	32,47361	31,34022	21,94433	30,41901	30,48693	23,17258
23,27266	23,05515	24,49419	28,20527	23,44089	22,41971	23,52586	28,58692	28,11098
27,87473	26,84828	26,64403	24,01014	22,0047	23,69696	22,98111	23,15847	24,53848
26,06747	26,14028	26,19261	25,5405	24,27242	24,804	22,52962	23,97202	24,54699
33,84633	33,18176	32,9191	34,94026	34,86277	34,7928	34,7456	34,83479	34,94576
30,52673	30,50503	30,39479	30,92741	30,84236	31,31484	31,244	31,09213	31,13589
28,80999	28,71509	28,60918	28,03239	28,31825	28,30138	28,43583	29,45138	29,59557
21,08503	22,76161	23,87801	23,80747	22,82011	21,57761	22,86011	27,84356	27,88681
29,0598	29,72306	29,90124	28,15528	28,18253	29,42274	29,04757	27,12495	27,30496
22,41892	21,9556	26,00719	26,80587	27,00089	26,6245	26,67681	26,63506	26,22015
31,72082	30,41649	30,87298	32,31355	32,22556	30,76975	31,00691	32,54494	32,64534
32,43964	31,66072	31,17665	34,3535	34,38985	33,92344	33,84763	34,53907	34,4746

31,91812	31,49729	31,76746	31,74021	32,05985	32,19759	32,46127	32,38785	32,43547
28,07572	28,64599	30,51625	25,26263	24,3812	26,74098	27,54458	24,61745	25,62589
28,97509	28,84161	27,99226	28,70902	29,11279	29,50673	29,20676	29,74953	29,41581
32,24073	32,4552	32,24505	30,98346	31,16423	32,62991	32,65057	31,34409	31,77999
28,89357	28,32582	28,6976	29,45996	29,7921	29,70604	29,07184	30,20455	30,12842
22,69794	22,9823	25,89069	26,70807	25,99596	21,42328	25,08148	26,87091	26,11504
28,86847	25,22255	23,93872	28,67949	28,82271	28,34875	28,94827	29,42582	30,35174
27,409	29,02929	28,78812	26,86962	27,06589	28,87678	28,89235	26,98	26,92343
21,14364	22,45959	21,6066	23,11883	23,52409	21,33205	22,62649	23,46276	23,12591
28,45591	27,97115	25,94675	30,13931	30,33543	29,29721	29,07702	30,74671	30,57362
29,18348	29,40099	28,37952	29,30898	29,31954	29,29999	29,52232	30,40304	30,03486
22,35912	23,95871	21,64258	23,57819	22,99253	21,29182	21,36832	24,78731	24,35057
29,74069	27,99857	28,44221	29,78396	29,52427	29,45252	29,43796	30,38915	30,22768
23,89384	24,98298	24,33845	23,1695	22,83973	24,19528	24,15267	22,20413	22,43987
27,79371	27,19094	27,70372	28,74622	29,1228	28,4729	28,49102	29,57782	29,8098
28,4394	29,1112	29,40734	28,72994	28,32444	28,37886	27,92914	26,89504	27,72286
29,42906	29,21403	29,45862	29,81929	30,12657	29,93551	29,57002	29,90985	30,32152
25,23356	26,33668	24,68886	24,4084	24,56209	26,16092	25,99401	23,71963	24,19764
24,86815	25,55318	24,44979	26,60399	26,9215	27,41828	26,91969	25,86976	26,79687
22,39099	22,82568	23,34163	29,5508	30,38618	27,79123	22,99408	31,32254	31,28312
23,98237	23,15892	23,08046	28,98454	28,68949	22,54555	22,31535	29,65041	29,91613
22,87294	22,8583	23,72993	27,1518	23,52378	22,30649	21,8716	28,1249	28,01036
24,52641	24,32745	22,21216	29,76836	29,95754	30,88103	31,05601	30,82533	29,67459
27,32672	27,07816	27,07908	23,25214	21,07985	26,08558	22,13194	20,47863	22,74585
27,87079	28,04323	27,51668	27,61996	27,93326	27,3379	27,7082	29,23938	29,22041

LFQ intens	LFQ intens	Potential c	Student's T	Student's T	Peptides	Razor + un	Unique pe	Sequence c
22,47785	30,51427	+	+	1_3	29	17	3	46,1
22,98594	25,6666	+	+	1_3	24	13	13	55
27,7845	34,07933	+	+	1_3	34	34	8	45,3
21,7265	20,35057		+	1_3	2	2	2	18,8
23,09436	24,24235		+	1_3	3	3	3	5,1
34,17042	33,84652		+	1_3	70	70	70	51,8
32,66465	32,65151		+	1_3	21	21	6	63,5
26,17122	25,20165		+	1_3	5	5	5	6,2
28,50199	28,15764		+	1_3	11	11	11	20,3
21,99211	27,43643		+	1_3	10	10	10	29,8
28,20741	25,89396		+	1_3	8	8	8	14,8
27,99145	27,2674		+	1_3	26	26	24	21,6
29,78757	29,64566		+	1_3	7	7	7	50,7
31,20432	31,15551		+	1_3	15	15	7	46,7
31,4446	31,74186		+	1_3	16	16	16	42
28,11083	27,75443		+	1_3	11	11	11	39
28,83533	29,82773		+	1_3	11	10	10	39,3
27,06497	27,54687		+	1_3	9	9	9	30,1
32,10793	32,37233		+	1_3	22	22	22	54,9
27,87085	28,74871		+	1_3	9	9	9	36,2
27,05258	26,74098		+	1_3	5	5	5	45,8
24,57306	24,00873		+	1_3	5	5	5	15
28,65591	27,46544		+	1_3	8	8	8	11,1
22,38684	22,3639		+	1_3	4	4	3	10,7
28,57841	27,46669		+	1_3	15	15	15	18
27,10059	25,79726		+	1_3	7	7	7	16
21,42224	19,70838		+	1_3	2	2	2	26,9
25,89375	25,32711		+	1_3	6	6	6	35,2
26,86962	27,13985		+	1_3	14	14	14	17,7
26,27837	27,31769		+	1_3	25	24	24	15,3
30,8017	30,96237		+	1_3	8	8	8	53,4
27,3946	27,05765		+	1_3	13	13	13	5,9
22,87529	22,73595		+	1_3	4	4	4	14,1
21,57464	22,0971		+	1_3	2	2	2	12
27,91239	27,94665		+	1_3	12	12	12	25,8
31,81867	31,29975		+	1_3	16	16	13	59,3
24,82182	23,49875		+	1_3	5	5	5	4,1
22,25546	22,98968		+	1_3	1	1	1	4,9
28,78924	27,59662		+	1_3	3	3	3	25,7
23,00949	22,52667		+	1_3	3	3	3	9,7
23,93957	25,17268		+	1_3	4	4	4	25,8
34,96456	35,41516		+	1_3	5	5	5	43,7
31,04785	30,74214		+	1_3	14	11	9	55,5
29,28701	29,04679		+	1_3	7	7	7	17,1
27,65055	23,1283		+	1_3	7	5	5	18,3
27,57795	29,18182		+	1_3	6	6	1	46,6
27,09225	28,22246		+	1_3	3	3	3	5,5
32,44586	31,76228		+	1_3	24	24	22	52,5
34,5739	34,16121		+	1_3	22	22	22	49,7

32,60213	32,57786	+	1_3	16	16	16	58,4
22,94758	26,84122	+	1_3	13	13	13	15,3
30,00945	29,20506	+	1_3	15	15	14	11,9
31,54209	30,68813	+	1_3	22	22	22	41,3
29,96472	30,12312	+	1_3	8	8	8	29,9
25,49308	26,83062	+	1_3	3	3	3	8,4
30,30726	29,43418	+	1_3	28	28	28	17,4
26,47011	27,54532	+	1_3	16	16	16	17,9
22,41431	22,69217	+	1_3	5	5	5	11,5
30,34742	29,24407	+	1_3	14	14	14	15,9
29,72728	30,19507	+	1_3	9	9	9	29,4
24,67632	24,27049	+	1_3	7	7	7	7,9
30,00223	29,88217	+	1_3	16	16	16	28,4
22,24566	23,55415	+	1_3	3	3	3	34,1
28,83843	28,87059	+	1_3	7	7	7	37,1
27,25422	27,97208	+	1_3	18	18	18	27,5
30,01492	30,15846	+	1_3	10	10	1	43,1
23,18006	22,52028	+	1_3	4	4	4	17,4
26,64072	25,56842	+	1_3	6	6	6	34,8
30,96747	22,67339	+	1_3	3	3	1	20,3
29,75681	27,0701	+	1_3	13	8	0	39,6
28,1209	25,79025	+	1_3	2	1	0	12,2
29,61569	29,87948	+	1_3	18	18	17	36,4
22,94437	23,02712	+	1_3	8	1	1	82,7
29,24386	28,63159	+	1_3	12	12	12	41

Unique + r	Unique sec	Mol. weigh	Q-value	Score	Intensity	MS/MS co	iBAQ 1-1	iBAQ 1-3
31,7	6,7	58,536	0	132,71	9,64E+09	86	53050000	60895000
34,5	34,5	51,267	0	187,1	1,04E+09	26	589190	5860500
45,3	9,3	62,378	0	323,31	6,27E+10	186	4,88E+08	2,73E+08
18,8	18,8	26,793	0	4,8618	17190000	16	64148	44043
5,1	5,1	94,411	0	5,6156	61725000	10	111380	169530
51,8	51,8	214,84	0	323,31	1,21E+11	873	1,62E+08	41900000
63,5	12,3	42,026	0	299,05	6,5E+10	215	4,69E+08	1,99E+08
6,2	6,2	116,43	0	15,039	3,87E+08	18	871770	230690
20,3	20,3	74,823	0	15,573	1,83E+09	39	3527200	1954600
29,8	29,8	41,174	0	13,75	1,99E+09	32	16969000	32253000
14,8	14,8	62,965	0	23,956	6,21E+08	21	0	0
21,6	20,5	187,15	0	220,13	1,57E+09	99	227840	107200
50,7	50,7	15,799	0	135,39	9,29E+09	132	2,94E+08	1,11E+08
46,7	15,5	36,154	0	269,99	1,65E+10	146	97461000	12038000
42	42	53,981	0	323,31	6,47E+10	186	1,07E+09	1,67E+08
39	39	46,611	0	119,95	1,95E+09	76	3136400	373360
35,8	35,8	37,294	0	196,48	6,44E+09	90	1,13E+08	60047000
30,1	30,1	44,552	0	30,727	1,61E+09	49	25298000	13145000
54,9	54,9	47,472	0	221,05	2,57E+10	201	56633000	29258000
36,2	36,2	35,936	0	24,174	3,46E+09	64	22675000	15380000
45,8	45,8	11,301	0	50,679	9,18E+08	36	4136500	37048
15	15	57,519	0	7,4178	1,92E+08	16	0	0
11,1	11,1	108,34	0	106,17	1,71E+09	49	3170500	255080
10,7	8,6	70,289	0	49,458	1,44E+08	16	321640	435020
18	18	122,02	0	73,637	2,05E+09	74	2874900	72081
16	16	57,936	0	6,1243	6,01E+08	19	733900	0
26,9	26,9	16,729	0	13,145	12704000	11	100530	0
35,2	35,2	20,195	0	36,023	2,77E+08	23	1245600	1639600
17,7	17,7	106,78	0	51,324	1,47E+09	59	6993100	634840
15	15	277,5	0	47,411	1,13E+09	69	869610	274400
53,4	53,4	14,374	0	29,647	1E+10	38	95637000	32647000
5,9	5,9	337,08	0	41,513	1,03E+09	34	237820	160850
14,1	14,1	45,421	0	6,8391	2,13E+08	8	9112400	44635
12	12	26,425	0	6,001	47823000	6	1015500	284770
25,8	25,8	67,877	0	41,025	2,24E+09	49	10391000	13131000
59,3	49,2	27,774	0	215,13	2,23E+10	164	1,68E+08	80143000
4,1	4,1	138,91	0	11,174	99376000	11	709760	0
4,9	4,9	53,119	0,007813	1,5315	1,76E+10	10	87305000	1,11E+08
25,7	25,7	19,365	0	17,92	1,25E+09	12	0	0
9,7	9,7	51,853	0	5,2514	3,99E+08	5	9752800	3496400
25,8	25,8	20,7	0	11,647	2,9E+08	17	7189200	2780300
43,7	43,7	13,714	0	140,96	2,01E+11	100	2,73E+09	7,9E+08
49,4	45,3	27,745	0	106,42	1,49E+10	87	74083000	51614000
17,1	17,1	55,394	0	28,686	3,58E+09	38	14707000	10814000
16	16	40,368	0	40,17	7,29E+08	18	1903000	277100
46,6	13,2	19,202	0	84,558	3,47E+09	35	37246000	61871000
5,5	5,5	70,169	0	9,1631	8,52E+08	16	1516000	725840
52,5	50,8	53,879	0	323,31	3,09E+10	218	1,29E+08	17027000
49,7	49,7	65,33	0	323,31	1,23E+11	243	2E+08	59886000

58,4	58,4	34,985	0	175,99	3,66E+10	166	1,12E+08	53009000
15,3	15,3	111,87	0	40,253	1,86E+09	33	9239900	5783600
11,9	11,5	189,24	0	122,13	4,78E+09	82	6085800	1337000
41,3	41,3	74,68	0	323,31	3,07E+10	260	2,4E+08	1,12E+08
29,9	29,9	40,876	0	39,226	6,46E+09	63	26180000	6088600
8,4	8,4	37,106	0	3,1167	4,69E+08	15	1428400	0
17,4	17,4	250,53	0	117,47	4,52E+09	135	9374900	102170
17,9	17,9	159,69	0	141,77	1,87E+09	82	3631400	4628600
11,5	11,5	57,929	0	5,5525	49544000	11	61890	0
15,9	15,9	130,93	0	106,13	6,76E+09	78	13404000	2328500
29,4	29,4	51,286	0	275,36	6,29E+09	60	36163000	13834000
7,9	7,9	144,37	0	15,203	91054000	25	40472	2135,8
28,4	28,4	85,206	0	183,26	6,39E+09	115	11127000	2828300
34,1	34,1	13,941	0	7,3546	98880000	17	457540	529600
37,1	37,1	23,031	0	11,535	3,44E+09	24	4439100	2042900
27,5	27,5	83,432	0	92,302	2,46E+09	69	8607800	13751000
43,1	2,6	38,39	0	226,3	7,48E+09	127	29603000	7991300
17,4	17,4	35,634	0	13,115	2,33E+08	21	4258900	6014000
34,8	34,8	24,093	0	11,912	6,03E+08	32	722330	1656600
20,3	11,6	14,813	0	41,996	6,83E+09	16	22583000	0
26,5	0	40,853	0	43,39	2,7E+09	34	0	0
6,1	0	21,216	0	6,2742	8,31E+08	8	3560700	839600
36,4	35	76,613	0	102,9	6,63E+09	108	572730	319880
20	20	7,99	0	16,266	6,66E+08	6	29338000	18754000
41	41	38,069	0	64,295	2,72E+09	54	13368000	2663600

iBAQ 1-5	iBAQ 2-1	iBAQ 2-2	iBAQ 2-3	iBAQ 2-4	iBAQ 3-1	iBAQ 3-2	iBAQ 3-3	iBAQ 3-4
97959000	7555900	4620300	20505000	16035000	28207	21636	24610	71577000
27575000	210850	461720	8027,8	43448	0	0	0	1083900
4,03E+08	65898000	50950000	1,09E+08	1,12E+08	11830000	4731800	3983700	5,01E+08
78521	64080	139190	143620	124760	89228	164760	177490	56133
0	230370	126850	164700	135830	159120	179900	151870	334010
52278000	1,62E+08	1,32E+08	81273000	82041000	2,05E+08	1,59E+08	1,5E+08	1,14E+08
2E+08	2,18E+08	1,91E+08	2,59E+08	2,69E+08	1,77E+08	1,89E+08	1,61E+08	1,68E+08
0	1967000	1524400	0	4279,3	1223100	902590	1676000	13886
639750	6368500	5430700	1430400	2615000	11815000	9264400	10239000	2180000
18283000	4233900	650300	226370	3595400	58313	0	0	10344000
0	9343,1	141610	9715,8	686790	5932600	4764000	7840100	24739
403000	2500100	1334000	1378500	1308900	3850300	1884600	2576400	658530
1,11E+08	1,08E+08	1,19E+08	1,06E+08	82367000	81481000	99246000	1,37E+08	79296000
38363000	96996000	89981000	48083000	39776000	1,26E+08	98471000	67365000	36460000
1,59E+08	3,83E+08	5,26E+08	1,52E+08	1,34E+08	1,12E+08	1,32E+08	40595000	65369000
4899900	37985000	38978000	131790	148490	4619400	5197000	12166000	6786300
47984000	33719000	11325000	13995000	28474000	15008000	19226000	12486000	23964000
9474200	4466100	4969200	4706900	4571000	4657000	4688800	3378700	5604800
30019000	65485000	89792000	52195000	55961000	1,92E+08	1,2E+08	1,97E+08	1E+08
17152000	23998000	13087000	14632000	14478000	7597800	4544800	5377200	18418000
643540	15559000	11083000	4065100	2373300	13966000	67739000	6657100	4849400
0	1918000	1511600	552490	640380	1071300	1375000	792110	150430
398550	1362900	584540	2554100	1024400	5081000	9786300	6024300	2069500
509920	1673100	350350	253220	301970	27477	10296	0	0
564620	2742100	951150	1184800	1042700	14862000	8506800	7509700	2337300
64855	1795100	4334600	229810	107300	3928000	4247600	2324300	432860
0	235270	204010	117010	93420	221170	313830	216280	86530
232680	886330	483090	178550	1638200	11482000	1409300	9706600	1869100
5683200	6872300	1801300	458650	1399800	1234300	1101400	2277800	2742400
1250000	726740	334760	1807200	721410	794420	429520	106020	1253900
29674000	80375000	1,16E+08	42079000	81811000	1,9E+08	2,94E+08	3,28E+08	1,43E+08
114580	511800	515550	841040	596960	1513900	1613200	382330	370260
17609	54107	1699900	897600	19699	6072,3	0	0	0
865890	939080	67069	33608	92605	237110	204290	169660	437960
7034500	12217000	2674500	8963800	9819600	5250600	2834400	8120600	8996500
1,01E+08	1,54E+08	1,76E+08	68020000	1,05E+08	1,41E+08	1,21E+08	1,93E+08	85416000
0	0	0	0	0	919880	91628	84405	69351
1,34E+08	2,39E+08	1,61E+08	71774000	45707000	84781000	39352000	80158000	46462000
105440	34258000	9122300	0	0	31512000	25213000	40969000	14786000
3898700	426320	0	239380	520440	662200	0	0	0
4583300	1771200	708920	423580	135310	1041400	2859000	1657300	5877900
8,16E+08	4,44E+09	3,85E+09	3E+09	3,04E+09	3,67E+09	3,43E+09	3,97E+09	3,71E+09
64358000	1,26E+08	1,05E+08	1,03E+08	1E+08	1,47E+08	1,21E+08	1,1E+08	59192000
11216000	10729000	6854500	7890300	8859200	22486000	31864000	9694800	7987600
0	6970200	1768200	1349200	785500	13768000	12798000	3233600	0
38834000	24155000	14584000	58162000	27051000	14253000	23726000	14500000	32792000
1262900	4497700	3815300	1429400	2603200	4132900	953940	4290500	3173600
32548000	1,44E+08	1,37E+08	29788000	30909000	1,87E+08	1,77E+08	1,51E+08	71265000
37044000	6,17E+08	6,56E+08	3,42E+08	3,78E+08	8,27E+08	5,07E+08	6,61E+08	4,61E+08

89459000	2,27E+08	1,72E+08	80458000	1,9E+08	2,73E+08	77437000	2,05E+08	1,84E+08
10602000	682660	107950	3928300	1997000	157980	575110	13801	2641500
1267800	2882900	6371400	4784800	4056400	15235000	4018500	21550000	2641200
79332000	38383000	35204000	1,24E+08	93233000	64160000	79940000	1,1E+08	12496000
8828800	18841000	39162000	31162000	18598000	70020000	52721000	78125000	53814000
606330	1428800	705030	385330	366140	6432200	5349700	7451000	1874600
11247	2089000	4025700	2553100	389920	8701000	8097200	8049000	10453000
4508100	1681400	1779200	4359700	4883800	125270	1068700	781010	1739900
169680	356090	276600	30010	112440	333970	228640	180750	155470
594070	19758000	19612000	11722000	4948700	14865000	27437000	21406000	10862000
8268700	40633000	37509000	16472000	19337000	50362000	39359000	30740000	21783000
21210	126400	78488	13311	4421,6	397330	213590	275180	296080
4598400	18907000	12056000	11895000	10116000	32147000	25670000	21787000	12655000
380930	394450	233370	333960	298120	154210	2507900	5769400	1300500
8304200	29925000	25528000	23943000	6387100	53006000	50748000	40329000	20034000
5932800	8989800	4447200	8801900	5473300	4462300	4106700	282780	1566800
18015000	42766000	51836000	25624000	15732000	49831000	47011000	44505000	41083000
2969700	211360	210550	320570	293730	95702	57033	50182	59157
2198700	9511400	9987700	12240000	5983300	440490	4866300	4471200	2736900
17176000	2,09E+08	3,59E+08	33559000	14240	4,98E+08	8,76E+08	2,59E+08	1914200
0	22733000	25546000	16728	19658	31133000	41316000	29162000	229830
831260	10998000	2704600	1052500	0	23233000	16291000	17599000	5953100
32887	11261000	14385000	31679000	46276000	30816000	12062000	21324000	10422000
17654000	34429000	9474100	6613300	4293100	27189	5624200	2548800	4417600
3127200	10215000	6946400	1788900	3031000	21819000	14093000	21828000	9978000

Student's T	Student's T	Student's T	Student's T	Majority pr	Fasta headers
0,034293	0,022694	7,316549	2,777731	tr|B4DRU6	tr|B4DRU6|B4DRU6_HUMAN cDNA FLJ5465
0,050732	0,037134	4,254315	2,413468	sp|P08779	sp|P08779|K1C16_HUMAN Keratin, type I cy
0,061664	0,047726	4,330429	2,273466	CON__P13	;sp|P13647|K2C5_HUMAN Keratin, type II cy
0,01885	0,01561	2,039369	2,928409	tr|A0A1U9	tr|A0A1U9X8Y4|A0A1U9X8Y4_HUMAN Chlo
0,012447	0,016	-1,27935	-2,93821	tr|E9PFW2	tr|E9PFW2|E9PFW2_HUMAN Beta-mannosi
0,003473	0,015077	-0,73153	-3,03878	sp|O00468	sp|O00468-6|AGRIN_HUMAN Isoform 6 of A
0,007343	0,033818	0,550653	2,430953	tr|Q6FI18|	tr|Q6FI18|Q6FI18_HUMAN CYR61 protein C
0,005679	0,013217	-1,70491	-3,6418	sp|O60245	sp|O60245-2|PCDH7_HUMAN Isoform B of
0,000456	0,022667	-1,78568	-5,58742	sp|O75326	sp|O75326|SEM7A_HUMAN Semaphorin-7A
0,021682	0,016333	4,551895	3,06938	sp|O75635	sp|O75635-2|SPB7_HUMAN Isoform 2 of Se
0,00128	0,0255	-4,28619	-5,64933	tr|B8ZX62	tr|B8ZX62|B8ZX62_HUMAN Plasminogen ac
0,001779	0,018462	-2,55714	-4,89241	tr|V9HWA	tr|V9HWA9|V9HWA9_HUMAN C3-beta-c OS
0,003941	0,02328	0,615016	2,772365	tr|A0A0K0	tr|A0A0K0K1J1|A0A0K0K1J1_HUMAN Epidic
0,001028	0,010857	-0,70534	-3,46927	tr|A0A0S2	tr|A0A0S2Z3D5|A0A0S2Z3D5_HUMAN Apol
0,01221	0,015294	1,74356	3,142239	tr|Q5TZP0	tr|Q5TZP0|Q5TZP0_HUMAN Matrix metallo
0,008388	0,011692	-2,19498	-3,53422	tr|B2R7Y0	tr|B2R7Y0|B2R7Y0_HUMAN cDNA, FLJ93654
0,01042	0,015892	1,285225	3,04511	tr|A0A5F9	tr|A0A5F9ZHM4|A0A5F9ZHM4_HUMAN L-la
0,003655	0,012667	1,174918	3,574166	sp|P07339	sp|P07339|CATD_HUMAN Cathepsin D OS=H
0,000316	0,015	-0,93058	-4,5211	tr|Q53H39	tr|Q53H39|Q53H39_HUMAN Activin beta-A
0,011353	0,026769	0,924987	2,745744	tr|V9HWE0	tr|V9HWE0|V9HWE0_HUMAN Annexin OS=
0,006294	0,014105	-3,00684	-3,92786	sp|P09341	sp|P09341|GROA_HUMAN Growth-regulate
0,006137	0,01216	-1,59661	-3,53822	tr|F8WCZ6	tr|F8WCZ6|F8WCZ6_HUMAN Complement C
0,024739	0,024314	-2,11675	-2,75918	tr|A0A087	tr|A0A087X0S5|A0A087X0S5_HUMAN Colla
0,016944	0,015474	2,237365	3,04046	tr|V9HWJ7	tr|V9HWJ7|V9HWJ7_HUMAN Epididymis se
0,026007	0,027245	-2,17389	-2,73478	sp|P14543	sp|P14543-2|NID1_HUMAN Isoform 2 of Nic
0,001158	0,0204	-3,34537	-5,54856	sp|P14618	sp|P14618|KPYM_HUMAN Pyruvate kinase
0,026253	0,026741	2,136597	2,722433	tr|B4DMM	tr|B4DMM8|B4DMM8_HUMAN cDNA FLJ57
0,019047	0,021778	-1,62695	-2,81901	tr|A0A024	tr|A0A024R8V7|A0A024R8V7_HUMAN Met
0,007728	0,016	0,86482	2,871235	sp|P16144	sp|P16144-5|ITB4_HUMAN Isoform Beta-4E
0,010336	0,0168	1,328457	3,073596	tr|Q60FE6	tr|Q60FE6|Q60FE6_HUMAN Filamin A OS=H
0,001936	0,016	-1,96337	-4,55844	tr|E9PPJ5|	tr|E9PPJ5|E9PPJ5_HUMAN Midkine (Fragme
0,000275	0,022667	-3,94665	-7,36183	sp|P25391	sp|P25391|LAMA1_HUMAN Laminin subuni
0,008275	0,014476	2,698631	3,654264	sp|P27930	sp|P27930|IL1R2_HUMAN Interleukin-1 rec
0,006211	0,013818	1,86415	3,646028	tr|Q5U0A0	tr|Q5U0A0|Q5U0A0_HUMAN Proteasome s
0,016082	0,032369	0,795854	2,463424	tr|V9HWD	tr|V9HWD9|V9HWD9_HUMAN Epididymis l
0,00715	0,016372	-0,84859	-2,88922	sp|P31947	sp|P31947|1433S_HUMAN 14-3-3 protein s
0,016995	0,022043	-1,3724	-2,79881	sp|P32004	sp|P32004-3|L1CAM_HUMAN Isoform 3 of I
0,037222	0,026316	6,485979	2,700111	sp|P35408	sp|P35408|PE2R4_HUMAN Prostaglandin E2
0,000215	0	-4,66361	-7,91369	tr|Q8NE89	tr|Q8NE89|Q8NE89_HUMAN Similar to tissu
0,001445	0,018545	3,814066	5,433379	sp|P53634	sp|P53634|CATC_HUMAN Dipeptidyl peptid
0,004109	0,0134	1,725639	3,876007	sp|P55145	sp|P55145|MANF_HUMAN Mesencephalic a
0,001546	0,017143	-1,72434	-4,58269	sp|P61769	sp|P61769|B2MG_HUMAN Beta-2-microglo
0,00504	0,030159	-0,52899	-2,50669	tr|D0PNI1	tr|D0PNI1|D0PNI1_HUMAN Epididymis lum
0,007747	0,031593	-0,63377	-2,56031	sp|P78324	sp|P78324-2|SHPS1_HUMAN Isoform 2 of T
0,046381	0,031313	-4,05242	-2,47211	tr|E9PKG6	tr|E9PKG6|E9PKG6_HUMAN Nucleobindin-2
0,031674	0,031148	1,763947	2,531562	tr|Q5T0R7	tr|Q5T0R7|Q5T0R7_HUMAN Adenylyl cyclas
0,029477	0,023167	-3,58191	-2,78364	tr|Q7RTW	tr|Q7RTW3|Q7RTW3_HUMAN Neuregulin 1
0,019577	0,027273	-1,34618	-2,70457	sp|Q02818	sp|Q02818|NUCB1_HUMAN Nucleobindin-1
0,000444	0,017	-2,6782	-6,26647	tr|A0A0S2	tr|A0A0S2Z3Y1|A0A0S2Z3Y1_HUMAN Galec

0,001357	0,011259	-0,7732	-3,50888	sp|Q1284[illegible]	sp|Q12841|FSTL1_HUMAN Follistatin-relate
0,016736	0,014133	4,07128	3,24855	sp|Q1286[illegible]	sp|Q12860-2|CNTN1_HUMAN Isoform 2 of
0,030712	0,044889	-0,99197	-2,29305	tr|G3V511	tr|G3V511|G3V511_HUMAN Latent-transfo
0,018473	0,031067	0,975089	2,541871	tr|A0A0S2	tr|A0A0S2Z4Q2|A0A0S2Z4Q2_HUMAN Tran
0,0002	0,019429	-1,4662	-5,82935	sp|Q1676[illegible]	sp|Q16769|QPCT_HUMAN Glutaminyl-pept
0,046254	0,039072	-2,47044	-2,38103	sp|Q6H3X[illegible]	sp|Q6H3X3|ULBP5_HUMAN UL-16 binding [illegible]
0,028757	0,022522	-3,86984	-2,81843	sp|Q6UVK	sp|Q6UVK1|CSPG4_HUMAN Chondroitin su
0,034609	0,037353	1,429087	2,396592	sp|Q6YHK[illegible]	sp|Q6YHK3-4|CD109_HUMAN Isoform 4 of
0,037786	0,047081	-1,18718	-2,26868	tr|B3KRK1	tr|B3KRK1|B3KRK1_HUMAN cDNA FLJ34442
0,014619	0,01325	-2,77002	-3,23162	sp|Q8IUX7	sp|Q8IUX7|AEBP1_HUMAN Adipocyte enha
0,01626	0,027034	-1,10207	-2,68928	sp|Q9274[illegible]	sp|Q92743|HTRA1_HUMAN Serine protease
0,025166	0,026786	-1,8677	-2,70089	sp|Q9282[illegible]	sp|Q92823-5|NRCAM_HUMAN Isoform 5 of
0,028217	0,030645	-1,39815	-2,50812	sp|Q9998[illegible]	sp|Q99985|SEM3C_HUMAN Semaphorin-3C
0,011454	0,014303	1,794139	3,200996	tr|A0A140	tr|A0A140VJY7|A0A140VJY7_HUMAN Thior
0,003589	0,014889	-1,71137	-3,96387	sp|Q9BRK[illegible]	sp|Q9BRK5-4|CAB45_HUMAN Isoform 4 of
0,00934	0,013677	1,524933	3,232225	tr|Q4ZG85	tr|Q4ZG85|Q4ZG85_HUMAN Uncharacteriz
0,001953	0,012276	-0,73395	-3,27842	sp|Q9UBP[illegible]	sp|Q9UBP4|DKK3_HUMAN Dickkopf-related
0,018767	0,015238	2,015296	2,926387	tr|B7Z5J4|	tr|B7Z5J4|B7Z5J4_HUMAN Carboxypeptidas
0,035723	0,042479	-1,2619	-2,32651	tr|W8GRM	tr|W8GRM7|W8GRM7_HUMAN Kallikrein 1
0,057767	0,040057	-6,20886	-2,3592	tr|D7RVA3	tr|D7RVA3|D7RVA3_HUMAN Granulocyte c
0,001054	0,0136	-5,69111	-6,06688	tr|A0A1V1	tr|A0A1V1IFQ2|A0A1V1IFQ2_HUMAN MHC
0,001788	0,017	-4,35788	-5,3072	tr|A0A288	tr|A0A288Q9P9|A0A288Q9P9_HUMAN MH
0,000287	0	-6,31009	-7,85561	tr|F5H2D0	tr|F5H2D0|F5H2D0_HUMAN Complement s
0,001113	0,022667	4,862326	5,896164	tr|C9JB55|	tr|C9JB55|C9JB55_HUMAN Serotransferrin
0,002192	0,015765	-1,27358	-3,97254	tr|Q5M8T[illegible]	tr|Q5M8T4|Q5M8T4_HUMAN Connective ti

i7, highly similar to Keratin, type II cytoskeletal 6A OS=Homo sapiens OX=9606 PE=2 SV=1;sp|P02538

ytoskeletal 5 OS=Homo sapiens OX=9606 GN=KRT5 PE=1 SV=3;tr|B4E1T1|B4E1T1_HUMAN cDNA FL
oride intracellular channel protein OS=Homo sapiens OX=9606 PE=3 SV=1;tr|Q5SRT3|Q5SRT3_HUMA
dase OS=Homo sapiens OX=9606 GN=MANBA PE=1 SV=1;tr|B4DT18|B4DT18_HUMAN Beta-mannos
Agrin OS=Homo sapiens OX=9606 GN=AGRN;sp|O00468-7|AGRIN_HUMAN Isoform 7 of Agrin OS=Hc
)S=Homo sapiens OX=9606 GN=CYR61 PE=1 SV=1;sp|O00622|CCN1_HUMAN CCN family member 1 (
Protocadherin-7 OS=Homo sapiens OX=9606 GN=PCDH7;sp|O60245|PCDH7_HUMAN Protocadherin
\ OS=Homo sapiens OX=9606 GN=SEMA7A PE=1 SV=1;sp|O75326-2|SEM7A_HUMAN Isoform 2 of Se
erpin B7 OS=Homo sapiens OX=9606 GN=SERPINB7;sp|O75635|SPB7_HUMAN Serpin B7 OS=Homo s
ctivator OS=Homo sapiens OX=9606 GN=PLAT PE=2 SV=1;sp|P00750|TPA_HUMAN Tissue-type plasm
S=Homo sapiens OX=9606 GN=HEL-S-62p PE=2 SV=1;sp|P01024|CO3_HUMAN Complement C3 OS=H
dymis secretory protein Li 2 OS=Homo sapiens OX=9606 GN=HEL-S-2 PE=2 SV=1;sp|P01034|CYTC_HU
ipoprotein E isoform 1 (Fragment) OS=Homo sapiens OX=9606 GN=APOE PE=2 SV=1;sp|P02649|APC
proteinase 1 (Interstitial collagenase) OS=Homo sapiens OX=9606 PE=2 SV=1;tr|Q53G96|Q53G96_H
4, highly similar to Homo sapiens serpin peptidase inhibitor, clade B (ovalbumin), member 2 (SERPINI
actate dehydrogenase OS=Homo sapiens OX=9606 GN=LDHB PE=1 SV=1;sp|P07195|LDHB_HUMAN L
Homo sapiens OX=9606 GN=CTSD PE=1 SV=1;tr|V9HWI3|V9HWI3_HUMAN Cathepsin D OS=Homo sa
chain (Fragment) OS=Homo sapiens OX=9606 PE=2 SV=1;tr|A4D1W7|A4D1W7_HUMAN Activin bet
Homo sapiens OX=9606 GN=HEL-S-7 PE=2 SV=1;sp|P08758|ANXA5_HUMAN Annexin A5 OS=Homo s

C1s subcomponent OS=Homo sapiens OX=9606 GN=C1S PE=1 SV=1;tr|A0A087X232|A0A087X232_H
gen alpha-1(VI) chain OS=Homo sapiens OX=9606 GN=COL6A1 PE=1 SV=1;tr|A0A384P5H7|A0A384P
cretory protein Li 37 OS=Homo sapiens OX=9606 GN=HEL-S-37 PE=2 SV=1;tr|Q53FI1|Q53FI1_HUMA
dogen-1 OS=Homo sapiens OX=9606 GN=NID1;sp|P14543|NID1_HUMAN Nidogen-1 OS=Homo sapie
PKM OS=Homo sapiens OX=9606 GN=PKM PE=1 SV=4;tr|V9HWB8|V9HWB8_HUMAN Pyruvate kinas
'328, highly similar to Beta-1,4-galactosyltransferase 1 OS=Homo sapiens OX=9606 PE=2 SV=1;tr|B7Z
alloproteinase inhibitor 2 OS=Homo sapiens OX=9606 GN=TIMP2 PE=3 SV=1;tr|A0A140VK57|A0A14
of Integrin beta-4 OS=Homo sapiens OX=9606 GN=ITGB4;tr|Q59H46|Q59H46_HUMAN Integrin bet
Homo sapiens OX=9606 GN=FLNA PE=2 SV=1;sp|P21333|FLNA_HUMAN Filamin-A OS=Homo sapiens
ent) OS=Homo sapiens OX=9606 GN=MDK PE=1 SV=1;sp|P21741|MK_HUMAN Midkine OS=Homo sa

eptor type 2 OS=Homo sapiens OX=9606 GN=IL1R2 PE=1 SV=1;sp|P27930-2|IL1R2_HUMAN Isoform
subunit alpha type OS=Homo sapiens OX=9606 PE=2 SV=1;tr|A0A109NGN6|A0A109NGN6_HUMAN F
uminal protein 107 OS=Homo sapiens OX=9606 GN=HEL107 PE=2 SV=1;tr|Q53EM5|Q53EM5_HUMA
igma OS=Homo sapiens OX=9606 GN=SFN PE=1 SV=1;sp|P31947-2|1433S_HUMAN Isoform 2 of 14-3
Neural cell adhesion molecule L1 OS=Homo sapiens OX=9606 GN=L1CAM;sp|P32004-2|L1CAM_HUN

ue factor pathway inhibitor 2 (Fragment) OS=Homo sapiens OX=9606 PE=2 SV=1;sp|P48307-2|TFPI2_
lase 1 OS=Homo sapiens OX=9606 GN=CTSC PE=1 SV=2;tr|B4DJQ8|B4DJQ8_HUMAN Cathepsin C OS
astrocyte-derived neurotrophic factor OS=Homo sapiens OX=9606 GN=MANF PE=1 SV=3;tr|A8K878|
bulin OS=Homo sapiens OX=9606 GN=B2M PE=1 SV=1;tr|B4E0X1|B4E0X1_HUMAN Beta-2-microglol
inal protein 4 OS=Homo sapiens OX=9606 GN=YWHAZ PE=2 SV=1;sp|P63104|1433Z_HUMAN 14-3-3
yrosine-protein phosphatase non-receptor type substrate 1 OS=Homo sapiens OX=9606 GN=SIRPA;s
2 OS=Homo sapiens OX=9606 GN=NUCB2 PE=1 SV=2;sp|P80303-2|NUCB2_HUMAN Isoform 2 of Nuc
se-associated protein 1 (Fragment) OS=Homo sapiens OX=9606 GN=CAP1 PE=1 SV=1;tr|Q5T0R6|Q5T
isoform HRG-beta2 OS=Homo sapiens OX=9606 GN=NRG1 PE=2 SV=1;tr|B0FYA7|B0FYA7_HUMAN I
L OS=Homo sapiens OX=9606 GN=NUCB1 PE=1 SV=4;tr|A8K7Q1|A8K7Q1_HUMAN Nucleobindin-1 O
ctin-3-binding protein (Fragment) OS=Homo sapiens OX=9606 GN=LGALS3BP PE=2 SV=1;sp|Q08380|

:d protein 1 OS=Homo sapiens OX=9606 GN=FSTL1 PE=1 SV=1;sp|Q12841-2|FSTL1_HUMAN Isoform
Contactin-1 OS=Homo sapiens OX=9606 GN=CNTN1;tr|A0A024R104|A0A024R104_HUMAN Contacti
rming growth factor beta-binding protein 2 OS=Homo sapiens OX=9606 GN=LTBP2 PE=1 SV=1;tr|G3\
ısforming growth factor-beta-induced protein ig-h3 (Fragment) OS=Homo sapiens OX=9606 GN=TGFI
ide cyclotransferase OS=Homo sapiens OX=9606 GN=QPCT PE=1 SV=1;sp|Q16769-2|QPCT_HUMAN
›rotein 5 OS=Homo sapiens OX=9606 GN=RAET1G PE=1 SV=1;sp|Q6H3X3-2|ULBP5_HUMAN Isoform

CD109 antigen OS=Homo sapiens OX=9606 GN=CD109;sp|Q6YHK3|CD109_HUMAN CD109 antigen (
! fis, clone HLUNG2001507, highly similar to Amphoterin-induced protein 2 OS=Homo sapiens OX=96
ncer-binding protein 1 OS=Homo sapiens OX=9606 GN=AEBP1 PE=1 SV=1;tr|B4DJB3|B4DJB3_HUMA
: HTRA1 OS=Homo sapiens OX=9606 GN=HTRA1 PE=1 SV=1;tr|Q05DJ8|Q05DJ8_HUMAN HTRA1 prot
' Neuronal cell adhesion molecule OS=Homo sapiens OX=9606 GN=NRCAM;sp|Q92823-3|NRCAM_HI
: OS=Homo sapiens OX=9606 GN=SEMA3C PE=2 SV=2;tr|B4E2I9|B4E2I9_HUMAN Semaphorin-3C OS
edoxin domain-containing protein 17 OS=Homo sapiens OX=9606 PE=2 SV=1;sp|Q9BRA2|TXD17_HU
45 kDa calcium-binding protein OS=Homo sapiens OX=9606 GN=SDF4;tr|G3V1E2|G3V1E2_HUMAN 4
ed protein CRIM1 (Fragment) OS=Homo sapiens OX=9606 GN=CRIM1 PE=4 SV=1;sp|Q9NZV1|CRIM1_
I protein 3 OS=Homo sapiens OX=9606 GN=DKK3 PE=1 SV=2;tr|F6SYF8|F6SYF8_HUMAN Dickkopf-re
se A4 OS=Homo sapiens OX=9606 GN=CPA4 PE=1 SV=1;sp|Q9UI42-2|CBPA4_HUMAN Isoform 2 of Ca
0 protein 9 (Fragment) OS=Homo sapiens OX=9606 GN=KLK10 PE=2 SV=1;tr|A0A024R4N7|A0A024R
olony-stimulating factor (Fragment) OS=Homo sapiens OX=9606 GN=CSF3 PE=2 SV=1;tr|A0A0B4U5E
class I antigen OS=Homo sapiens OX=9606 GN=HLA-C PE=3 SV=1;tr|A0A0S4T3H1|A0A0S4T3H1_HUI

ubcomponent C1r OS=Homo sapiens OX=9606 GN=C1R PE=1 SV=3;tr|Q53HU9|Q53HU9_HUMAN Cc

issue growth factor OS=Homo sapiens OX=9606 GN=CTGF PE=2 SV=1;tr|B3KRV6|B3KRV6_HUMAN cl

;|K2C6A_HUMAN Keratin, type II cytoskeletal 6A OS=Homo sapiens OX=9606 GN=KRT6A PE=1 SV=3;t

J54081, highly similar to Keratin, type II cytoskeletal 5 OS=Homo sapiens OX=9606 PE=2 SV=1;tr|B4D
\N Chloride intracellular channel protein OS=Homo sapiens OX=9606 GN=CLIC1 PE=2 SV=2;tr|Q53FB
;idase OS=Homo sapiens OX=9606 PE=2 SV=1;tr|A8K6D3|A8K6D3_HUMAN Beta-mannosidase OS=H
omo sapiens OX=9606 GN=AGRN;sp|O00468-3|AGRIN_HUMAN Isoform 3 of Agrin OS=Homo sapiens
OS=Homo sapiens OX=9606 GN=CCN1 PE=1 SV=1;tr|B4DI61|B4DI61_HUMAN cDNA FLJ58182, highly

emaphorin-7A OS=Homo sapiens OX=9606 GN=SEMA7A;tr|F5GYX3|F5GYX3_HUMAN Semaphorin-7
apiens OX=9606 GN=SERPINB7 PE=1 SV=1;tr|A8K3Q8|A8K3Q8_HUMAN cDNA FLJ75069, highly simil
ninogen activator OS=Homo sapiens OX=9606 GN=PLAT PE=1 SV=1;tr|B4DNJ1|B4DNJ1_HUMAN T-pl
Homo sapiens OX=9606 GN=C3 PE=1 SV=2;tr|B4E216|B4E216_HUMAN C3-beta-c OS=Homo sapiens

OE_HUMAN Apolipoprotein E OS=Homo sapiens OX=9606 GN=APOE PE=1 SV=1;tr|A0A346DBY2|A0A
UMAN Matrix metalloproteinase 1 preproprotein variant (Fragment) OS=Homo sapiens OX=9606 PE
32), mRNA OS=Homo sapiens OX=9606 PE=2 SV=1;sp|P05120|PAI2_HUMAN Plasminogen activator i
L-lactate dehydrogenase B chain OS=Homo sapiens OX=9606 GN=LDHB PE=1 SV=2;tr|Q5U077|Q5U0
apiens OX=9606 GN=HEL-S-130P PE=2 SV=1;tr|A0A1B0GWE8|A0A1B0GWE8_HUMAN Cathepsin D O
a-A chain OS=Homo sapiens OX=9606 GN=INHBA PE=2 SV=1;sp|P08476|INHBA_HUMAN Inhibin bet
sapiens OX=9606 GN=ANXA5 PE=1 SV=2;tr|E9PHT9|E9PHT9_HUMAN Annexin OS=Homo sapiens OX

UMAN Complement C1s subcomponent OS=Homo sapiens OX=9606 GN=C1S PE=1 SV=1;tr|B3KNX0|
5H7_HUMAN Collagen, type VI, alpha 1, isoform CRA_b OS=Homo sapiens OX=9606 GN=COL6A1 PE
N L-plastin variant (Fragment) OS=Homo sapiens OX=9606 PE=2 SV=1;tr|A0A024RDT4|A0A024RDT4
ens OX=9606 GN=NID1 PE=1 SV=3;tr|A0A384MR25|A0A384MR25_HUMAN Epididymis secretory spe
se OS=Homo sapiens OX=9606 GN=HEL-S-30 PE=2 SV=1;sp|P14618-2|KPYM_HUMAN Isoform M1 of
ZAH9|B7ZAH9_HUMAN cDNA, FLJ79193, highly similar to Beta-1,4-galactosyltransferase 1 OS=Homo
0VK57_HUMAN Metalloproteinase inhibitor 2 OS=Homo sapiens OX=9606 PE=2 SV=1;sp|P16035|TI
a (Fragment) OS=Homo sapiens OX=9606 PE=2 SV=1;sp|P16144-4|ITB4_HUMAN Isoform Beta-4D of
OX=9606 GN=FLNA PE=1 SV=4;sp|P21333-2|FLNA_HUMAN Isoform 2 of Filamin-A OS=Homo sapien
piens OX=9606 GN=MDK PE=1 SV=1;tr|E9PLM6|E9PLM6_HUMAN Midkine OS=Homo sapiens OX=9

Proteasome subunit alpha type OS=Homo sapiens OX=9606 PE=2 SV=1;sp|P28066|PSA5_HUMAN Pr
N Transketolase variant (Fragment) OS=Homo sapiens OX=9606 PE=2 SV=1;sp|P29401|TKT_HUMAN

MAN Isoform 2 of Neural cell adhesion molecule L1 OS=Homo sapiens OX=9606 GN=L1CAM;tr|Q7Z3Z

_HUMAN Isoform 2 of Tissue factor pathway inhibitor 2 OS=Homo sapiens OX=9606 GN=TFPI2;sp|P4

A8K878_HUMAN cDNA FLJ77177, highly similar to Homo sapiens arginine-rich, mutated in early stag
bulin OS=Homo sapiens OX=9606 PE=2 SV=1;tr|A6XMH4|A6XMH4_HUMAN Beta-2-microglobulin O
protein zeta/delta OS=Homo sapiens OX=9606 GN=YWHAZ PE=1 SV=1;tr|E7EX29|E7EX29_HUMAN
p|P78324|SHPS1_HUMAN Tyrosine-protein phosphatase non-receptor type substrate 1 OS=Homo s
leobindin-2 OS=Homo sapiens OX=9606 GN=NUCB2;sp|P80303|NUCB2_HUMAN Nucleobindin-2 OS
T0R6_HUMAN Adenylyl cyclase-associated protein 1 (Fragment) OS=Homo sapiens OX=9606 GN=CA
Heregulin-gamma protein isoform 1 (Fragment) OS=Homo sapiens OX=9606 GN=NRG1 PE=2 SV=1;sp
S=Homo sapiens OX=9606 PE=2 SV=1;tr|Q53GX6|Q53GX6_HUMAN Nucleobindin-1 (Fragment) OS=
LG3BP_HUMAN Galectin-3-binding protein OS=Homo sapiens OX=9606 GN=LGALS3BP PE=1 SV=1;tr|

2 of Follistatin-related protein 1 OS=Homo sapiens OX=9606 GN=FSTL1;tr|B4DTZ8|B4DTZ8_HUMAN
in 1, isoform CRA_a OS=Homo sapiens OX=9606 GN=CNTN1 PE=3 SV=1;sp|Q12860|CNTN1_HUMAN
V3X5|G3V3X5_HUMAN Latent-transforming growth factor beta-binding protein 2 OS=Homo sapiens
3I PE=2 SV=1;sp|Q15582|BGH3_HUMAN Transforming growth factor-beta-induced protein ig-h3 OS=
Isoform 2 of Glutaminyl-peptide cyclotransferase OS=Homo sapiens OX=9606 GN=QPCT;tr|B5MCZ9|
2 of UL-16 binding protein 5 OS=Homo sapiens OX=9606 GN=RAET1G;sp|Q5VY80|ULBP6_HUMAN U

OS=Homo sapiens OX=9606 GN=CD109 PE=1 SV=2;sp|Q6YHK3-2|CD109_HUMAN Isoform 2 of CD109
06 PE=2 SV=1;tr|A0A024R127|A0A024R127_HUMAN Adhesion molecule with Ig-like domain 2, isofo
N cDNA FLJ55979, highly similar to Homo sapiens AE binding protein 1 (AEBP1), mRNA OS=Homo sap
tein (Fragment) OS=Homo sapiens OX=9606 GN=HTRA1 PE=2 SV=1;tr|B3KRN4|B3KRN4_HUMAN cDN
UMAN Isoform 3 of Neuronal cell adhesion molecule OS=Homo sapiens OX=9606 GN=NRCAM;sp|Q9

UMAN Thioredoxin domain-containing protein 17 OS=Homo sapiens OX=9606 GN=TXNDC17 PE=1 SV=
45 kDa calcium-binding protein OS=Homo sapiens OX=9606 GN=SDF4 PE=1 SV=1;sp|Q9BRK5-3|CAB4
_HUMAN Cysteine-rich motor neuron 1 protein OS=Homo sapiens OX=9606 GN=CRIM1 PE=1 SV=1
lated protein 3 OS=Homo sapiens OX=9606 GN=DKK3 PE=1 SV=1;tr|O43532|O43532_HUMAN RIG-li
arboxypeptidase A4 OS=Homo sapiens OX=9606 GN=CPA4;tr|A4D1M3|A4D1M3_HUMAN Carboxype
4N7_HUMAN Kallikrein 10, isoform CRA_a OS=Homo sapiens OX=9606 GN=KLK10 PE=4 SV=1;tr|W8C
3|A0A0B4U5E3_HUMAN Granulocyte colony-stimulating factor (Fragment) OS=Homo sapiens OX=96
MAN MHC class I antigen OS=Homo sapiens OX=9606 GN=HLA-C PE=3 SV=1;tr|A0A5H2UZM2|A0A5H

omplement subcomponent C1r (Fragment) OS=Homo sapiens OX=9606 PE=2 SV=1;tr|Q53HT9|Q53HT

DNA FLJ34961 fis, clone NTONG2003839, highly similar to CONNECTIVE TISSUE GROWTH FACTOR OS

=9606 GN=ANXA5 PE=1 SV=1;tr|B4DNG6|B4DNG6_HUMAN Annexin

LFQ intens 1	LFQ intens 1	LFQ intens 1	LFQ intens 2	LFQ intens 2	LFQ intens 2	LFQ intens 2	LFQ intens 3	LFQ intens 3
29,78377	31,4543	31,45292	30,42302	31,36656	30,96987	31,01065	30,13394	29,97937
31,83876	32,27556	33,19985	30,84416	30,55536	31,14108	31,12127	28,38928	27,05992
31,03118	32,02697	32,30549	28,31095	28,08035	29,06669	28,91336	20,79574	24,09746
24,49698	28,37501	30,17415	21,70664	24,67246	22,18999	22,98011	22,38854	22,66983
25,30247	23,26814	30,43599	25,17741	22,8621	24,30911	24,60071	22,0442	20,70717
33,83921	33,80713	34,3397	31,56883	31,73376	32,68712	32,62625	29,26591	27,52992
24,74222	24,60337	30,64418	25,10153	25,00449	21,73413	23,56802	21,70214	22,83614
28,93188	30,30051	31,04999	27,05144	26,39643	28,41143	27,3339	23,9612	24,71225
22,99402	22,92194	23,06814	24,61234	24,17456	23,74875	29,0791	27,32199	28,00646
21,99236	23,77998	23,67256	20,3393	20,89156	21,14923	20,90991	20,98905	21,36959
30,68529	30,28703	30,85964	30,81879	30,90928	30,77941	30,85823	30,37399	30,38577
22,19592	22,21376	21,9892	22,62654	23,66371	22,6054	22,51538	23,07883	23,2337
33,43204	33,12132	33,52426	34,14444	34,1734	33,66034	33,77686	34,1496	34,19638
24,80228	24,53925	22,37404	22,42995	25,92134	25,04222	24,20963	24,18624	20,9857
33,44233	33,07312	33,15658	32,61617	32,74256	33,55408	33,48424	32,51371	32,86356
23,0782	24,21573	24,30058	23,73355	23,35205	23,34027	24,0616	23,02624	23,14763
22,09261	23,93129	25,10321	24,61857	23,12668	22,78339	22,55286	22,82993	22,60608
33,00556	32,62901	32,61581	32,94073	32,86133	32,7856	33,02602	33,07253	33,08569
25,96016	26,23316	28,2069	28,04411	27,45591	27,88495	27,38714	26,86667	26,94492
28,59754	29,47365	29,71357	28,63596	28,62502	29,0331	28,81403	28,41393	28,11629
24,54144	24,05358	24,24405	26,21647	26,88285	23,95601	23,44199	25,95476	26,61077
31,73759	30,27275	29,1043	30,81215	30,58262	30,93818	30,58414	32,10942	31,34757
26,97235	27,53875	23,70641	22,08734	23,46811	22,2817	22,63029	27,70695	27,51427
22,93884	22,8041	29,19943	28,65775	22,67563	28,79383	29,29743	22,48849	23,56157
27,09295	26,39926	26,58606	28,29824	28,05274	26,49989	26,80193	28,56384	28,69026
28,13104	28,25503	28,74641	28,37977	28,43305	28,94693	28,85878	27,75085	27,60159
28,65492	30,31646	29,05628	27,62944	27,36328	25,98713	27,53282	24,71282	25,02129
24,86585	25,66858	26,89342	24,55467	24,4985	24,65529	21,50152	24,3995	26,34206
28,38961	29,04604	27,39198	27,05734	28,2615	27,86071	27,11128	28,68201	29,20016
29,42252	28,33654	28,53126	29,20744	28,26616	28,82299	28,99418	29,84019	30,15798
32,96418	32,67141	32,896	33,34889	33,30157	32,60907	33,00887	33,36307	33,28444
28,45391	23,5664	23,91694	23,14386	27,57788	21,32798	23,06441	27,5285	28,11277
26,35839	28,33151	26,54073	25,614	26,20596	27,04458	26,9249	21,48138	26,02842
29,36146	28,99612	29,57028	29,23779	28,91288	28,17327	28,51661	29,26282	28,85243
30,43559	30,01983	30,76928	30,10749	29,93579	30,29734	30,09572	29,60846	29,72095
22,9875	22,37928	25,87683	23,99438	24,1868	26,35328	25,0115	21,9154	24,04902
25,27824	25,41796	23,60397	25,33149	25,43039	26,54541	26,21545	25,56404	25,33248
22,87401	22,50927	23,7104	24,00533	25,3654	23,46208	26,69856	27,58857	27,57975
24,83543	25,70187	25,88129	28,09989	27,96157	27,57521	27,98994	28,46045	28,40068
31,3198	32,1689	30,87943	30,74903	30,78659	30,80301	30,76256	31,63901	31,60342
30,49037	30,49486	30,10011	30,22458	30,02314	29,36208	29,68611	29,85046	29,70337
29,46919	29,0311	30,18648	29,65763	29,75707	28,64516	28,96063	28,84972	29,1438
32,04986	32,27134	32,02815	32,79498	32,75521	31,31802	31,96716	31,79052	32,39749
22,55595	24,25068	24,56715	23,0456	23,46548	22,989	23,09163	22,47135	22,52411
25,95821	26,75468	26,51845	26,03626	26,50314	25,78497	25,75491	23,51093	25,66473
27,68646	22,77694	24,11508	27,24998	26,84374	26,66519	26,03526	27,90133	26,9982
29,76029	28,75646	28,52832	29,17926	28,34744	27,79631	27,73098	27,82171	27,83864
30,69594	30,59067	30,49295	30,91663	30,91748	30,831	30,68361	31,33527	31,499

23,29637	25,09152	25,01103	23,23882	23,89069	25,0916	25,45848	23,61908	24,25263
22,49253	22,52481	25,14794	23,05078	22,8054	21,69381	22,78836	22,47301	22,89599
39,82963	40,3972	40,3675	39,97216	40,09383	39,7945	39,99011	39,55767	39,61191
28,40186	29,72688	29,72761	28,52862	29,12443	28,77982	28,26808	27,7348	28,20983
34,12325	32,30138	33,52869	33,55225	33,47255	32,45425	32,37485	31,58352	31,52687
30,13883	29,47183	28,8308	29,95934	29,9157	29,73702	29,175	29,41877	29,20156
22,62466	25,91079	25,23092	22,80388	24,75694	24,96521	25,17489	22,48554	23,17973
25,66971	25,48395	23,57503	26,97126	26,78694	26,20247	26,27021	26,45172	26,68565
26,47663	27,08861	28,3572	27,35481	27,29658	25,45707	25,58337	26,46857	26,36766
27,38303	28,39734	32,13302	27,33253	26,12108	25,9608	27,51307	23,24587	21,69751
25,05519	28,31765	30,25214	28,23571	26,87373	28,07923	27,23425	27,06764	29,22977
28,35342	27,33415	26,39274	27,92875	27,98555	26,77706	26,85115	27,75793	28,13335
34,81926	34,74259	34,96942	35,25993	35,31477	34,69872	34,8409	35,07112	34,91665
26,38739	29,42734	29,03265	28,95795	27,89891	30,17985	28,8887	28,28105	27,13134
25,75524	24,58153	26,69803	29,68005	28,98682	23,46787	24,53582	27,83545	27,79228
31,47581	29,98983	30,45582	31,25259	31,19366	29,44123	28,88757	32,26411	32,34884
23,90181	23,45889	22,8481	23,49253	23,7263	23,40093	23,49521	23,95961	24,07227
23,96913	23,94351	25,28884	24,42714	23,9736	24,42822	25,31517	24,33688	24,59429
26,46247	26,39802	26,11337	27,12495	26,4502	26,37867	25,8764	26,75824	27,07745
27,74175	26,71686	26,68284	27,62485	28,0773	27,45834	28,0267	27,94447	27,14248
26,32468	28,36611	28,62708	26,59861	26,49697	27,7252	27,80107	26,69432	26,16611
32,49028	32,54522	32,02716	32,55034	32,41956	33,49118	32,99525	31,95491	31,90081
30,75422	30,03604	31,20005	30,95088	30,94831	30,04218	29,87449	29,93227	29,90297
24,87891	25,97367	25,76981	25,20105	24,93405	25,19959	24,85771	23,93373	24,59821
29,45912	29,6348	29,13973	29,22299	29,29959	29,25986	29,29769	28,45709	28,59715
23,09327	22,72077	24,42926	29,01081	29,04226	29,36077	29,08563	28,76284	29,60442
29,35548	22,51285	27,11108	30,4184	29,97528	22,12187	27,80931	31,11166	30,12854
30,29351	30,37098	30,32034	29,59586	29,41542	29,22338	29,18959	28,92872	28,58043
30,80209	30,56074	30,1162	30,4565	30,12842	30,64134	30,5539	30,16949	30,31008
28,33769	28,78279	28,03837	26,8832	27,00131	27,44979	27,41224	27,20602	27,0276
30,47373	29,53393	29,67622	30,88358	30,62104	30,64581	30,31149	29,83079	30,10974
31,64736	31,49781	31,01824	31,2683	31,09339	30,62052	31,00618	31,18943	30,85712
26,02485	22,60765	26,55919	26,79699	23,39697	26,79476	25,26446	22,918	27,45403
30,69917	30,19084	29,30687	30,18459	30,73287	31,3022	31,05705	30,66619	30,62043
29,70017	31,17516	30,40669	29,74439	29,73148	30,72525	31,16249	29,19221	29,52684
32,1478	31,99044	31,91071	32,20597	32,28532	32,09276	32,07431	32,46709	32,60202
36,66924	36,67477	37,06567	36,23622	36,39741	36,92138	36,83975	36,54697	36,51467
28,83641	27,71673	28,26006	29,13582	28,71823	28,22366	28,1248	27,88332	27,59399
31,38469	31,11873	31,19578	31,53723	31,60116	31,29576	31,11471	32,09704	32,07728
29,73119	27,79749	28,15938	27,87701	28,09628	28,40255	28,18778	28,98829	29,14239
26,75468	27,26141	28,06712	27,51209	27,91102	27,01723	27,34706	27,24164	26,99949
24,06817	22,51959	22,60184	26,4321	24,59241	23,57057	24,31043	23,02665	24,03884
28,95074	29,18386	29,2963	28,62383	28,84107	29,17331	29,1775	28,1155	28,13951
23,45989	24,27916	24,54799	24,41886	21,94694	24,67745	24,417	24,42873	24,85529
25,5526	23,09792	24,06357	27,97301	27,77335	26,13055	25,72093	27,91296	27,27293
29,78544	27,05631	26,84074	30,28824	29,64406	28,51537	28,73463	30,31959	30,06958
31,08772	30,64736	31,39719	31,11787	31,49815	31,23026	31,08715	31,30883	31,2161
22,48696	23,48598	22,96295	25,59269	25,5305	25,10185	25,35279	24,66536	25,05383
26,77568	23,67122	27,37426	26,84182	26,88344	26,01937	25,8469	26,18799	26,702
23,47735	23,90008	23,88922	23,2737	23,33624	23,76486	23,2089	23,51364	24,24561

22,53613	23,74615	23,54647	25,32358	24,49582	24,10509	23,78548	23,13526	24,43243
28,64038	28,47406	29,14611	29,47485	28,93306	28,28498	28,2889	27,83286	28,09053
29,46527	28,86555	29,29649	28,86938	29,17762	28,87132	28,94419	29,59793	29,31106
27,06938	28,89143	28,26526	27,44261	27,41973	27,19639	27,51796	26,59974	26,78207
31,45405	31,32876	30,25327	31,0185	31,25175	30,93227	31,12368	31,43251	31,40009
32,5143	31,86172	32,04274	32,38851	32,54153	32,39657	32,43649	32,79676	32,60309
30,11496	29,17999	28,50233	30,25777	29,8096	29,40576	29,48535	30,18707	29,51655
27,49579	26,13406	25,21182	26,31403	26,73376	27,57087	27,8052	28,65363	28,81427
31,02373	31,7677	31,75561	31,30721	31,30948	31,69499	31,41825	30,72972	31,02281
27,14092	26,77228	27,16959	27,53349	27,53705	27,61034	27,64972	27,46521	27,64113
28,46443	27,67445	27,16615	27,87496	22,43601	28,38059	27,90029	29,15318	29,11966
26,15194	26,13663	26,40391	26,1744	25,58695	25,60925	25,44594	24,83359	24,6608
28,86977	28,23768	28,17874	27,6006	27,57167	27,58914	27,88367	28,00774	27,87121
27,03601	26,83581	26,9506	22,51562	27,15808	26,49095	26,48205	27,01041	26,90633
24,22059	25,35528	25,29018	24,60156	24,26556	24,5428	24,76157	24,10556	22,01563
28,37248	27,89389	29,32772	28,45128	27,78457	27,89192	27,72858	28,12623	27,99205
26,14332	23,01189	24,33834	27,70088	23,52517	23,64966	22,34579	27,47839	28,50415
27,54399	25,67411	26,14766	27,58628	27,5059	27,32174	27,3808	28,95366	29,51783
22,40098	23,81693	23,63272	26,63145	26,90392	27,20909	22,62445	26,7656	26,85222
28,62429	26,96345	27,71476	28,46848	28,2867	27,39526	27,73841	28,50469	28,57838
28,73053	24,52453	28,43924	28,86414	28,93092	28,77878	22,56012	23,16895	28,88824
22,22519	24,05714	23,08479	20,84115	20,80986	20,17731	20,20192	21,1201	21,69238
21,90548	23,68897	23,85056	25,06817	23,37174	25,15623	24,58164	22,24401	21,58573
27,4773	26,69458	28,40807	28,08233	27,94078	26,12778	26,64045	27,4259	27,39395
25,24107	23,69519	23,87995	25,01504	24,50355	23,90812	24,18526	26,31443	26,06079
30,49486	31,13663	32,0949	30,66195	29,75248	32,0186	31,615	30,53557	30,70471
27,68759	27,50385	28,24354	28,02189	26,97913	26,97978	27,68746	26,87467	26,90323
27,59634	27,47057	27,37923	28,11753	27,47908	28,82642	28,21933	28,18834	28,32693
22,38697	23,94143	25,0925	25,21749	22,11606	22,6314	25,75771	24,90724	22,14161
23,78216	26,74496	26,26912	26,66655	26,46883	26,35175	26,35805	26,64733	26,30196
33,49441	32,92128	32,59539	32,3817	32,26994	32,90683	32,90919	33,27711	33,37036
23,67721	22,78137	23,41876	26,10536	26,18569	25,36903	25,84886	23,16877	21,86169
25,58569	23,1495	22,57251	24,56248	27,52709	26,79613	26,39562	25,28355	24,47191
27,75513	28,21183	27,7604	26,90529	26,82905	27,53646	26,94905	26,56261	26,16398
29,76917	28,95427	28,57236	29,82902	30,14722	29,61994	30,10762	31,08759	31,39622
27,97104	24,13355	23,53183	25,66793	26,47122	22,09738	26,35919	29,65397	28,37335
24,58526	25,44518	25,80934	25,38788	25,90371	25,1848	25,36262	25,01636	25,70197
26,35753	25,14492	26,15178	27,13575	27,41579	25,93001	25,80102	26,41308	26,95005
29,00715	28,8387	29,76729	29,54305	29,31948	28,25399	29,28131	28,55421	28,74156
29,21792	32,58394	30,50654	28,12623	30,26919	29,19049	29,2372	26,63409	22,71205
29,22757	23,924	26,58721	27,54399	21,78727	27,06548	26,48171	29,63819	30,59495
29,96939	29,87926	28,24984	28,92269	29,53094	29,31214	29,26177	30,53853	30,19531
27,03149	27,33142	27,70081	27,11038	27,41716	26,63048	27,27622	26,92785	27,31829
30,34467	30,25372	30,40761	30,49343	30,13198	29,22607	29,44213	29,93312	29,39983
26,32978	25,5773	25,67737	27,30042	27,01595	27,28569	26,81115	26,51752	27,29413
23,30494	25,02753	24,20769	21,66731	21,06474	24,80292	24,66129	23,87211	23,80055
22,7308	23,63123	24,27583	26,40868	26,53424	27,85412	27,98462	27,96505	27,55311
23,01668	23,20372	24,27764	26,49803	26,36123	21,74912	26,1163	23,94667	22,99179
23,00669	23,45689	22,91417	22,25534	24,40763	23,71639	23,2798	23,6228	24,35692
23,84414	24,56426	24,14361	26,90047	27,01797	23,04612	26,99194	23,29207	21,7728

27,69737	26,80772	26,85555	28,44892	27,86455	28,01143	27,57427	28,09119	28,61376
32,21095	32,23803	32,37464	32,07061	31,97004	32,08949	31,83217	31,84176	31,17146
26,73104	25,19689	24,38988	22,31128	25,25555	24,68581	23,04007	23,24094	22,1104
23,23996	24,51734	23,0489	24,38555	22,96906	23,09225	23,3615	21,63014	21,64979
33,1712	32,76987	31,82775	32,78755	32,74049	32,65612	32,74585	33,03581	32,32381
33,56207	33,87646	33,62843	33,68301	34,18844	34,17913	34,08417	33,73721	32,55218
23,21048	24,95903	25,34147	24,46065	26,02003	22,13707	22,23981	25,55888	25,82129
28,62048	28,59463	28,16322	28,52099	28,36035	28,34617	28,57015	27,47212	27,3232
27,19563	26,77944	26,91718	26,77291	27,0135	27,20844	26,25686	26,69485	26,17672
29,05012	28,27177	27,92156	28,84554	27,93123	28,2327	28,08618	28,54197	28,47119
28,27728	24,215	24,57643	27,95427	26,79699	22,89161	26,77744	28,58499	28,03601
30,5313	28,74301	29,35727	30,95387	30,81261	29,55379	28,98414	30,88664	30,98956
22,72538	22,34425	21,81765	23,43149	21,9234	21,65298	21,76786	22,38853	21,80982
25,93488	26,56552	26,27538	27,25953	26,7604	27,72702	27,55186	27,5742	27,85792
30,49895	30,54563	31,03636	31,73884	31,96558	31,36124	31,4488	31,45983	31,59072
29,78499	29,14092	28,8289	29,47299	29,28615	29,43408	29,58163	29,46194	29,64397
23,25643	23,04572	22,66345	23,46148	22,90094	23,43582	23,54321	24,35057	24,77258
29,98251	29,46474	29,52045	29,93818	30,00638	29,49331	29,8263	30,06111	29,98861
30,53399	31,64255	31,45988	32,47361	31,34022	21,94433	30,41901	30,48693	23,17258
29,03701	27,4182	27,61744	30,29428	30,01478	29,87616	30,71498	26,92717	27,37103
29,41945	28,1755	27,99572	28,05388	27,88745	28,8979	28,90722	28,08811	28,4265
29,86488	29,6667	29,86606	30,12139	29,96637	29,59092	29,0759	29,22869	29,25798
26,29667	25,03846	24,52492	25,97248	25,33504	25,60096	26,30412	24,09491	24,71654
27,38492	28,44091	27,86685	27,42926	27,12416	28,70606	28,12297	27,77769	27,13065
29,24704	28,01919	28,46486	27,12475	27,50127	27,40162	27,56761	28,76853	29,01962
26,1668	27,14763	26,99043	26,60442	26,65335	27,12839	27,13193	25,5854	25,88229
22,12114	23,35111	23,77726	22,72528	23,21424	22,32888	23,98546	24,90405	23,08309
21,43329	21,26316	23,21829	21,77338	21,94389	23,43033	22,17335	21,88558	23,09263
23,27266	23,05515	24,49419	28,20527	23,44089	22,41971	23,52586	28,58692	28,11098
25,57245	23,58268	25,70585	23,47914	23,83335	25,23169	24,42541	23,34204	24,55397
27,6803	22,83008	27,2553	26,95638	27,37625	28,4215	27,46886	28,86166	27,3435
26,15642	26,49176	26,30198	26,3562	26,16638	26,56796	26,51907	26,16265	26,14602
27,24336	28,7107	23,02098	27,99916	28,12204	28,44762	28,63786	28,07086	28,57532
22,48635	23,09693	24,33101	23,50749	23,4279	23,46438	23,98442	22,792	22,30644
26,7406	27,39648	27,47885	26,64636	26,88367	27,06569	27,16873	27,61147	27,93061
27,87473	26,84828	26,64403	24,01014	22,0047	23,69696	22,98111	23,15847	24,53848
31,02538	28,0608	26,95283	29,20121	28,55677	20,3209	23,47313	29,68667	28,82845
27,63319	25,07255	25,58253	28,68067	28,78544	27,80101	27,63409	28,96416	29,14397
23,18387	21,93488	23,89936	22,62006	22,4368	23,5451	22,93451	22,33549	22,08537
26,06747	26,14028	26,19261	25,5405	24,27242	24,804	22,52962	23,97202	24,54699
20,66865	22,29856	22,86931	21,98116	22,22385	21,91947	22,37928	22,00295	21,68611
22,87826	27,17693	26,35365	27,31077	22,16631	26,01827	23,9734	28,36415	26,89446
22,46193	24,14566	23,93981	22,21463	22,41504	22,0996	22,17139	22,28238	22,73144
29,34995	30,30791	29,86489	29,35098	29,56202	29,8237	30,01265	29,09605	29,22467
25,43825	25,71874	26,0371	25,00924	25,57306	25,1314	25,22902	23,40778	24,62851
33,84633	33,18176	32,9191	34,94026	34,86277	34,7928	34,7456	34,83479	34,94576
30,32571	30,38061	29,69471	28,77445	28,88037	28,9153	29,22858	29,26152	29,23364
26,60484	26,35755	27,0202	27,37708	27,16029	27,31363	27,36853	25,76352	26,29381
28,24173	28,04682	28,11981	28,26481	28,57275	28,65615	28,37683	27,85293	27,32852
28,94824	27,49694	26,82177	27,39534	26,58663	28,36507	28,20186	27,2893	27,09305

31,21714	31,15509	31,74727	29,25818	28,87643	29,77217	30,3184	29,67677	28,89209
23,78097	24,24307	25,10033	24,19753	23,75817	24,66096	24,51147	24,51244	24,22118
25,80181	27,30374	27,68117	28,20662	28,06332	27,5666	27,60625	26,79959	27,09073
30,52673	30,50503	30,39479	30,92741	30,84236	31,31484	31,244	31,09213	31,13589
30,80186	30,69835	30,70347	31,23186	31,23181	30,83996	30,74166	31,29082	31,40202
20,94663	25,10793	29,54775	29,44782	23,03416	29,40538	29,19777	29,74551	28,54878
28,80999	28,71509	28,60918	28,03239	28,31825	28,30138	28,43583	29,45138	29,59557
32,08724	31,52272	31,39018	31,12805	31,05349	30,89829	31,05226	32,28653	32,35935
21,37512	22,7043	21,79578	23,34055	23,29488	23,16682	23,1216	21,44602	21,38864
21,08503	22,76161	23,87801	23,80747	22,82011	21,57761	22,86011	27,84356	27,88681
26,98512	23,88829	25,92445	25,79927	25,93492	27,40965	27,26428	27,23726	27,24245
28,7162	28,86865	28,6635	28,6732	28,22605	28,45517	28,25948	28,63775	28,65458
29,0598	29,72306	29,90124	28,15528	28,18253	29,42274	29,04757	27,12495	27,30496
22,41892	21,9556	26,00719	26,80587	27,00089	26,6245	26,67681	26,63506	26,22015
29,99105	30,73731	30,59566	28,90779	28,90976	30,09043	30,21048	29,82221	29,62558
26,36351	24,09426	23,43975	21,1175	25,6198	24,95832	25,10386	25,21593	25,46588
31,72082	30,41649	30,87298	32,31355	32,22556	30,76975	31,00691	32,54494	32,64534
32,07333	31,20705	31,79579	32,15551	32,04954	31,58289	31,57808	31,72428	31,43226
30,47383	30,22205	30,35374	30,26953	29,9057	30,05892	30,18966	29,8763	29,84151
27,64952	27,24627	27,75015	26,81982	27,20051	27,89735	27,65895	26,94213	27,03276
28,17693	22,669	27,6284	28,16111	28,41679	28,05352	27,52716	28,37902	28,13536
32,43964	31,66072	31,17665	34,3535	34,38985	33,92344	33,84763	34,53907	34,4746
27,63437	24,60421	23,3362	27,51187	27,37152	22,66718	24,74349	27,94436	27,13663
28,76202	29,10482	27,51923	29,5136	29,89116	30,42722	29,85855	29,03499	29,16816
31,91812	31,49729	31,76746	31,74021	32,05985	32,19759	32,46127	32,38785	32,43547
28,07572	28,64599	30,51625	25,26263	24,3812	26,74098	27,54458	24,61745	25,62589
27,91536	26,85626	27,82019	27,62401	27,78625	27,58771	27,64746	26,86207	27,31173
27,46544	27,27044	28,29281	28,24812	28,54374	27,47576	27,75251	27,67721	27,64856
23,619	22,80758	24,78621	24,2452	21,4702	22,93478	22,62632	27,23781	26,85875
27,90621	23,60109	25,8839	28,06358	28,09984	22,3937	22,48816	28,32225	27,60406
31,3891	32,20644	31,21784	31,12712	31,16075	32,05462	31,98217	31,1827	31,06676
35,727	35,08001	34,21993	35,31125	35,36708	34,7692	34,89767	35,99507	36,21522
27,78075	25,62995	26,9862	28,32822	27,9165	26,24567	26,04947	28,63682	27,9645
26,8405	27,13956	27,20359	26,94044	27,01073	26,72546	26,91319	27,10179	26,94949
33,14163	33,0862	33,01676	32,73808	32,75444	32,81086	32,49651	32,88815	32,58511
28,97509	28,84161	27,99226	28,70902	29,11279	29,50673	29,20676	29,74953	29,41581
26,53551	26,10412	25,55915	26,66384	25,96404	26,32924	26,47822	26,15151	25,37518
24,86117	24,58233	24,77077	24,16615	21,33634	22,8082	23,02755	24,55356	23,5323
27,41345	27,08192	26,77027	26,86479	27,41401	25,27459	25,02644	26,20646	25,87446
27,04302	26,42065	26,76307	27,92224	27,7395	26,83509	26,89018	27,84667	27,64746
32,24073	32,4552	32,24505	30,98346	31,16423	32,62991	32,65057	31,34409	31,77999
32,34393	32,45435	32,4553	32,94348	32,95486	32,43572	32,60366	32,71596	32,97266
30,05672	29,71406	30,27453	30,81138	30,90792	29,45875	29,50932	30,14819	30,21037
25,77042	26,13144	26,3175	26,07843	26,08483	26,78818	26,09887	24,87235	25,29915
28,89357	28,32582	28,6976	29,45996	29,7921	29,70604	29,07184	30,20455	30,12842
32,37023	33,27378	33,19432	32,67712	32,71778	33,38317	33,51136	32,40586	32,37746
27,82457	22,22908	26,8411	28,13467	27,67101	27,37815	26,85958	27,07449	26,65567
22,98498	23,10019	24,09997	22,65718	23,60543	21,79398	23,99501	22,06209	23,02199
22,69794	22,9823	25,89069	26,70807	25,99596	21,42328	25,08148	26,87091	26,11504
22,44216	22,29515	21,8745	26,8918	27,93343	22,3351	23,1682	27,71803	27,88821

28,86847	25,22255	23,93872	28,67949	28,82271	28,34875	28,94827	29,42582	30,35174
27,409	29,02929	28,78812	26,86962	27,06589	28,87678	28,89235	26,98	26,92343
21,14364	22,45959	21,6066	23,11883	23,52409	21,33205	22,62649	23,46276	23,12591
28,06902	26,75175	22,9078	28,69942	28,62457	26,73854	27,21234	28,70547	28,86124
28,45591	27,97115	25,94675	30,13931	30,33543	29,29721	29,07702	30,74671	30,57362
28,08396	28,29772	27,10669	27,45772	27,76989	21,23318	27,06969	28,09018	28,31385
32,25367	31,42842	31,05543	32,6382	32,8189	30,53751	30,67152	33,03574	32,60869
21,83059	22,35942	23,95503	22,99011	22,52471	22,67241	22,55497	23,16306	23,2048
24,49363	25,39369	25,63425	25,80092	25,88462	26,39831	26,72988	23,14286	25,68035
28,25142	28,18698	28,13892	27,08172	26,78731	28,6101	28,57236	27,50385	27,46599
29,18348	29,40099	28,37952	29,30898	29,31954	29,29999	29,52232	30,40304	30,03486
22,35912	23,95871	21,64258	23,57819	22,99253	21,29182	21,36832	24,78731	24,35057
28,59399	28,64935	28,3438	28,34164	28,56107	28,7376	28,03249	28,91088	28,1472
23,26826	27,42005	26,16066	26,02789	26,17493	27,22894	27,662	25,26964	22,28789
27,09154	27,17693	23,85863	22,72165	27,13438	26,72078	26,47979	21,88856	25,99466
26,93348	25,91684	25,40669	24,97456	26,01767	26,10015	26,38017	26,146	25,63702
29,70532	30,88729	30,16854	28,60038	28,53267	30,07915	30,23421	28,67074	28,63737
29,74069	27,99857	28,44221	29,78396	29,52427	29,45252	29,43796	30,38915	30,22768
23,89384	24,98298	24,33845	23,1695	22,83973	24,19528	24,15267	22,20413	22,43987
27,79371	27,19094	27,70372	28,74622	29,1228	28,4729	28,49102	29,57782	29,8098
28,50881	27,51089	27,90828	28,44557	28,73841	28,99021	28,80557	28,76819	28,57578
27,09778	28,37061	27,34087	27,58606	28,19315	29,02324	28,3803	27,94921	28,35094
26,33748	23,30331	25,61288	27,87596	27,00689	25,76532	25,80087	26,28348	26,59548
23,76749	27,1333	26,10571	22,37297	24,27405	25,9091	26,6363	24,42316	24,67955
28,4394	29,1112	29,40734	28,72994	28,32444	28,37886	27,92914	26,89504	27,72286
27,38278	28,32813	27,62819	28,76294	27,96752	27,93703	27,80538	27,67324	28,35288
29,42906	29,21403	29,45862	29,81929	30,12657	29,93551	29,57002	29,90985	30,32152
29,70731	30,41084	27,82766	27,80655	27,55391	26,67101	28,73386	28,55757	28,7679
25,23356	26,33668	24,68886	24,4084	24,56209	26,16092	25,99401	23,71963	24,19764
28,70514	29,27173	29,10652	29,75592	29,60495	27,91085	29,09439	23,45103	29,32959
28,92337	29,32068	29,82935	28,1907	29,30967	28,75875	28,64375	28,84852	28,08639
28,39334	29,27344	27,98462	25,62171	25,95491	26,39537	27,39174	27,75786	27,45301
28,35368	27,61267	26,77128	27,12001	23,49497	27,9723	28,18461	28,13506	27,65928
26,15244	28,02205	27,40251	22,93626	21,8841	27,4373	28,38414	22,43506	21,28574
28,20592	29,57266	28,76135	28,06327	28,50116	28,72306	29,16674	27,72468	28,35069
37,42421	38,73302	38,14632	37,56347	37,76457	39,12461	39,19818	38,10963	38,10559
24,86815	25,55318	24,44979	26,60399	26,9215	27,41828	26,91969	25,86976	26,79687
30,00437	30,95768	31,37932	29,33731	29,04497	31,8546	31,86924	29,74962	29,76882
33,4021	33,25462	32,80859	33,53125	33,70419	33,37244	33,57678	33,78107	33,82231
22,39099	22,82568	23,34163	29,5508	30,38618	27,79123	22,99408	31,32254	31,28312
23,98237	23,15892	23,08046	28,98454	28,68949	22,54555	22,31535	29,65041	29,91613
25,87103	22,70381	27,51443	28,61765	29,86532	24,29221	26,28829	29,95421	28,29167
6MJE0_HU	tr|A0A1D6ZNI8|A0A1D6ZNI8_HUMAN MHC class I antigen (Fragment) OS=Homo sapiens							
22,87294	22,8583	23,72993	27,1518	23,52378	22,30649	21,8716	28,1249	28,01036
23,24184	25,25133	23,87793	22,94744	22,44208	24,57404	24,97657	23,29908	22,72538
24,52641	24,32745	22,21216	29,76836	29,95754	30,88103	31,05601	30,82533	29,67459
30,33947	28,09894	28,59385	30,34794	29,98726	29,70336	30,16591	29,10734	29,01404
28,38681	28,48585	28,70879	29,32706	30,13516	28,76816	28,2122	29,11647	28,88899
28,80649	28,7424	28,63395	28,24127	28,88405	28,61041	28,39844	28,49209	28,58223
33,24076	33,12498	33,48015	33,36071	33,09248	32,49522	32,63644	32,6707	32,82423

30,90821	30,49895	28,22628	29,54652	29,25636	29,20595	29,30136	30,01185	30,42352
27,32672	27,07816	27,07908	23,25214	21,07985	26,08558	22,13194	20,47863	22,74585
30,37223	31,0658	30,05453	30,20233	30,64341	30,85162	22,18038	30,42542	30,56047
32,99998	30,75756	31,38283	34,14186	33,95593	33,23891	32,67272	33,22777	33,01876
23,78987	22,73767	24,36073	22,48009	28,41708	23,01492	22,59114	29,18362	29,21988
27,43913	28,14739	24,51552	28,59602	28,23759	22,3943	27,99841	22,1329	27,55054
27,87079	28,04323	27,51668	27,61996	27,93326	27,3379	27,7082	29,23938	29,22041

LFQ intens	LFQ intens	Potential c	Student's T	Student's T	Peptides	Razor + un	Unique pe	Sequence c
3	3							
30,2014	30,51841	+			11	11	11	75,3
27,47568	32,87364	+			28	28	6	64,6
22,47785	30,51427	+			29	17	3	46,1
22,98594	25,6666	+			24	13	13	55
24,41796	24,93535	+			20	17	16	43,5
27,7845	34,07933	+			34	34	8	45,3
25,49537	22,65014	+			16	15	15	41,6
20,61423	30,23261	+			25	13	12	57,6
27,20592	22,63946				2	2	2	28,6
21,7265	20,35057				2	2	2	18,8
30,30171	30,06201		+	2_3	18	18	3	26,5
23,09436	24,24235				3	3	3	5,1
34,17042	33,84652				70	70	70	51,8
24,45632	25,07292				4	4	1	18,2
32,66465	32,65151				21	21	6	63,5
22,90845	23,64265				2	2	2	5,9
21,30471	22,27878				2	2	2	12,3
33,04968	32,51022				105	105	105	39,2
26,48228	27,71646				11	11	1	22,7
28,66848	29,82502				30	22	22	42,2
26,17122	25,20165				5	5	5	6,2
31,53765	30,2683				5	5	5	39,7
27,15528	27,30669		+	2_3	8	8	6	30,8
21,97597	22,60404		+	2_3	3	3	3	11,3
28,50199	28,15764				11	11	11	20,3
27,83786	29,16813				27	27	26	14,9
21,99211	27,43643				10	10	10	29,8
22,73737	24,74796				6	6	6	7,4
28,69604	29,52217		+	2_3	5	5	5	31,7
29,86137	27,6372				6	6	6	25,6
33,09179	33,19459				37	37	1	43,9
28,13501	22,22175				5	5	5	20,9
23,22935	26,05658				4	4	4	18,4
28,74945	29,10052				8	8	8	43
29,64069	30,2934				15	15	14	51,5
22,70791	24,7093				4	4	4	10,8
25,40788	25,35387				3	3	3	12,3
28,20741	25,89396				8	8	8	14,8
27,99145	27,2674				26	26	24	21,6
31,44618	31,4631		+	2_3	8	8	8	83,2
29,78757	29,64566				7	7	7	50,7
28,67182	28,42146				11	11	11	15,4
32,01475	31,72012				2	2	2	29,8
22,40927	24,00084				2	2	2	30,8
22,03864	25,182				4	4	4	11
28,0004	27,1547				3	3	3	17
27,53148	28,49377				16	16	16	24,1
31,20432	31,15551				15	15	7	46,7

23,92379	23,30522				110	2	0	59,9
22,62042	23,47148				1	1	1	6,3
39,64786	40,11942				83	83	1	86,4
27,70807	29,21914				9	9	9	29,7
31,4446	31,74186		+	2_3	16	16	16	42
29,4308	29,30269				12	12	10	34,2
23,66297	23,16938				3	3	3	58,2
26,87853	25,42037				7	7	7	29,2
26,64623	27,34528				6	6	6	28,4
27,07296	27,94229	+			25	19	14	37,6
29,44583	25,93578				9	9	9	37,3
27,75168	23,33238				3	3	3	20,6
34,82941	34,81855				27	27	25	47,5
28,28476	28,96832				4	4	4	39,8
28,11083	27,75443				11	11	11	39
32,45405	31,01618				10	10	10	39,6
24,05117	25,27188				6	6	6	15,6
24,60162	24,63229				3	3	3	37,4
26,54038	26,44435				7	7	7	10,9
27,71581	28,31799	+			10	10	10	14,5
26,22245	28,21919				4	4	4	44,7
31,98512	33,03262				4	4	4	45,9
30,21188	29,05245				18	18	18	51,6
24,57097	25,78114				7	7	7	15,4
28,89888	29,60247				6	6	6	27,9
29,71363	21,02804				6	6	3	21,1
30,15231	27,78332				12	12	12	40,7
28,83533	29,82773				11	10	10	39,3
30,02472	31,08278				17	17	17	39,8
27,06497	27,54687				9	9	9	30,1
29,83017	30,49486				22	22	22	65,2
30,98352	31,59513				18	18	18	43,9
22,2937	25,29489				5	4	4	16,9
30,88074	30,92825				9	9	9	82,1
29,10702	29,76517				12	12	12	42,5
32,33111	31,71759				51	51	51	36,5
36,31932	36,28361				72	72	1	67,9
28,00443	28,85103				14	14	6	24,7
32,10793	32,37233		+	2_3	22	22	22	54,9
29,07694	28,57261		+	2_3	12	12	12	10
26,96939	28,13036				10	10	10	7,7
23,22929	22,0688				8	7	6	16,5
27,87085	28,74871				9	9	9	36,2
25,1225	24,67659				3	3	3	25,9
27,05258	26,74098				5	5	5	45,8
30,39489	27,65663				6	6	6	60
31,25496	30,96279				13	13	13	49,5
24,57306	24,00873				5	5	5	15
26,72481	27,25836				5	5	5	61
24,13085	22,86091				2	2	2	9,1

23,92615	23,37663			9	6	4	16,8
28,17312	28,36102			7	7	7	57,7
29,11195	29,22637			28	28	28	20,2
26,89631	29,84933			3	3	3	32,4
31,51267	32,05937			26	26	26	45
32,76289	32,05148			57	57	57	48,3
29,70504	29,26924			15	15	12	28,5
28,65591	27,46544			8	8	8	11,1
30,71367	32,10659			31	31	23	45,9
27,3973	27,80489			8	8	8	17,3
28,72972	22,85296			2	2	2	10,4
25,12684	26,26031			9	9	9	15,2
28,37927	29,38264			15	15	15	23,4
27,25638	27,37219			4	4	4	25,6
22,38684	22,3639	+	2_3	4	4	3	10,7
27,63235	27,59278			4	4	4	36,9
28,08208	23,7703			1	1	1	8,2
28,57841	27,46669			15	15	15	18
27,10059	25,79726			7	7	7	16
28,42266	27,61982			14	13	4	23,6
22,04314	28,46303			4	4	4	15,5
21,42224	19,70838			2	2	2	26,9
26,05842	25,92295			3	3	3	6,1
28,12357	28,34177			7	7	7	24,2
25,89375	25,32711	+	2_3	6	6	6	35,2
30,21547	31,61986			5	5	5	6,6
26,86962	27,13985			14	14	14	17,7
27,85822	27,64877			6	6	6	51,3
25,46955	25,74981			8	8	8	9,4
26,56551	26,61526			5	5	5	26
33,19407	32,88736			18	18	18	65,3
24,02359	22,4964	+	2_3	2	2	2	21,4
26,76648	26,63948			4	4	4	20
26,27837	27,31769			25	24	24	15,3
30,8017	30,96237	+	2_3	8	8	8	53,4
28,90058	26,61583			5	5	5	17,7
25,07916	26,10211			3	3	3	29,8
26,33712	26,60964			7	7	7	13,5
28,69315	30,06625			5	5	2	31,8
27,77335	31,74053			5	5	3	70,8
30,15906	27,11862			5	5	5	23,6
30,52318	30,37389	+	2_3	13	13	4	30,9
28,05698	27,26785			15	15	15	19,5
29,78983	31,10905			7	7	7	33,2
27,1057	25,89901			7	7	7	38,2
23,78566	25,39061			58	1	0	42,8
27,3946	27,05765			13	13	13	5,9
21,65471	23,13136			5	5	5	21,4
23,61605	24,59007			4	4	4	26,8
23,14555	22,7834	+	2_3	2	2	2	2,7

28,86994	27,17207			11	11	11	19,1
31,98077	32,86765			15	15	15	56,2
22,87529	22,73595			4	4	4	14,1
21,57464	22,0971	+	2_3	2	2	2	12
32,67082	32,5158			21	21	21	46,5
34,00018	33,87317			7	7	7	46,9
25,26338	22,54016			4	4	4	18,5
27,91239	27,94665	+	2_3	12	12	12	25,8
26,32445	27,66452			4	4	4	24,1
28,50994	28,98076			11	11	9	25,8
28,68656	28,03889			5	5	5	6,9
30,83771	30,31084			5	5	5	32,7
21,06007	22,18177			2	2	2	17
27,67283	26,34415			10	4	4	43,4
31,81867	31,29975			16	16	13	59,3
29,81967	29,82792			5	5	5	41,9
24,82182	23,49875			5	5	5	4,1
30,07202	30,27487			16	16	16	39,6
22,25546	22,98968			1	1	1	4,9
27,52447	26,34316	+	2_3	21	18	18	24,7
27,55772	25,58446			23	23	23	8,7
29,45405	30,24762			32	32	32	21,5
24,94864	24,56889	+	2_3	3	3	1	9,6
27,70299	28,13775			10	10	10	52,9
28,78176	29,24681	+	2_3	7	7	7	6,7
25,67694	26,49496	+	2_3	5	5	5	20,7
23,256	22,54659			5	5	5	8,3
23,00335	21,34792			1	1	1	9,6
28,78924	27,59662	+	2_3	3	3	3	25,7
24,47914	25,04635			7	7	7	4,8
27,32002	22,81877			7	7	7	20,5
26,27046	26,74638			4	4	4	24,1
28,38718	28,44249			8	8	8	15,9
22,26167	24,22538			3	3	3	36,3
27,73596	27,54768	+	2_3	5	5	5	21
23,00949	22,52667			3	3	3	9,7
29,14936	26,91319			2	2	2	12,4
28,95333	26,63533			10	10	10	29
22,11832	21,33434			5	5	5	13,2
23,93957	25,17268			4	4	4	25,8
22,14252	20,9939			3	3	3	3
26,73195	26,98663			3	3	3	4,5
22,43952	22,41341			2	2	2	21,8
29,07181	30,04101			8	8	8	52,6
24,93472	26,07812			4	4	4	27,7
34,96456	35,41516			5	5	5	43,7
29,41357	29,77698			8	8	8	60,3
26,50303	27,1378			11	8	7	46,6
28,1601	27,92818			15	12	12	54,9
27,64678	27,53942			5	5	5	70,5

29,54481	31,90203			5	5	5	38,8
24,96899	21,43893			7	7	7	53,9
27,2516	28,17016			3	3	3	41,7
31,04785	30,74214			14	11	9	55,5
31,69387	30,24853			17	17	1	61,6
29,0922	28,78609			2	2	2	50
29,28701	29,04679	+	2_3	7	7	7	17,1
32,14659	31,15158			24	24	24	30,1
21,26774	22,32003	+	2_3	4	4	4	31,3
27,65055	23,1283	+	2_3	7	5	5	18,3
26,42686	26,03687			5	5	5	5,7
28,51273	28,56779			4	4	4	21,7
27,57795	29,18182			6	6	1	46,6
27,09225	28,22246			3	3	3	5,5
29,84894	30,19507			26	26	26	14,1
25,31316	25,29635			9	9	9	15,5
32,44586	31,76228			24	24	22	52,5
31,15672	31,89638			20	18	18	35,5
29,70914	30,1343			7	7	6	39,2
26,46579	27,45513			4	4	4	7,2
27,99394	28,1456			10	10	10	11,9
34,5739	34,16121			22	22	22	49,7
27,55223	26,41745			8	8	8	14,8
28,96058	28,41216	+	2_3	11	11	11	35,6
32,60213	32,57786			16	16	16	58,4
22,94758	26,84122			13	13	13	15,3
26,62324	25,73244			7	7	7	28,2
27,94559	28,32633			10	10	10	18,4
27,50945	21,8165			3	3	3	12,7
27,4753	21,95672			6	6	6	11,3
31,08087	31,5073			44	44	44	56,1
36,06305	35,62631	+	2_3	61	61	61	61,7
28,53118	27,55538			10	10	10	17,7
26,15883	27,18782			9	9	9	22,3
32,42542	33,33165			8	8	8	34,6
30,00945	29,20506			15	15	14	11,9
25,21656	26,79588			5	5	5	20,4
21,35252	22,27447			15	15	13	3,9
26,00071	26,82784			3	3	3	13,3
27,75983	26,20253			11	11	11	21,4
31,54209	30,68813			22	22	22	41,3
32,87197	32,17173			14	14	14	56,4
30,22159	30,5139			21	21	21	47,2
25,88159	25,53074			7	7	7	20,1
29,96472	30,12312			8	8	8	29,9
32,42114	32,80205			60	60	60	45,6
26,91547	26,26469			9	9	9	13,7
21,97225	24,02431			4	4	1	23,3
25,49308	26,83062			3	3	3	8,4
27,33151	21,88122			4	4	4	10,7

30,30726	29,43418	+	2_3	28	28	28	17,4
26,47011	27,54532			16	16	16	17,9
22,41431	22,69217			5	5	5	11,5
28,52671	28,23622			10	10	10	21,5
30,34742	29,24407			14	14	14	15,9
28,11709	26,58892			13	13	13	20,8
32,61489	30,55344			19	19	19	42,2
22,95408	22,2762			5	5	5	13,7
24,72858	26,61933			5	5	5	25,6
27,61253	28,66075			20	20	20	18,2
29,72728	30,19507	+	2_3	9	9	9	29,4
24,67632	24,27049	+	2_3	7	7	7	7,9
28,45254	29,0643			17	17	17	14,8
25,69238	26,59235			2	2	2	12,5
26,92411	23,56164			4	4	4	26,5
26,54432	25,27501			5	5	5	16,8
28,464	30,06381			46	46	46	21,2
30,00223	29,88217			16	16	16	28,4
22,24566	23,55415			3	3	3	34,1
28,83843	28,87059			7	7	7	37,1
28,83699	28,58405			11	11	11	36
28,34401	22,16493			4	4	4	74,2
26,7057	26,33868			8	8	8	10,1
22,79165	25,97412			9	9	9	5,5
27,25422	27,97208			18	18	18	27,5
28,57124	28,5634			9	9	9	9,7
30,01492	30,15846			10	10	1	43,1
27,71967	29,03572			4	4	4	15,2
23,18006	22,52028	+	2_3	4	4	4	17,4
24,1877	29,62633			10	10	10	7,1
29,00566	21,58528			5	5	5	25,6
26,76497	27,97405			7	7	7	39,7
27,5837	27,0662			10	10	10	16,5
22,62	27,64911			8	8	8	11,6
28,01845	28,46404			6	6	6	17,9
38,09501	37,49905			111	111	0	62,5
26,64072	25,56842			6	6	6	34,8
29,53185	31,83665			59	59	1	43,7
33,70913	33,52402			106	106	49	36,7
30,96747	22,67339			3	3	1	20,3
29,75681	27,0701			13	8	0	39,6
29,24266	26,092			14	14	0	56
; OX=9606 GN=HLA-A PE=3 SV=1;tr|A0A0S4XRM0|A0A0S4XRM0_HUMAN MHC class I antigen OS=Ho							
28,1209	25,79025			2	1	0	12,2
23,26055	23,22372			110	2	0	67
29,61569	29,87948			18	18	17	36,4
29,35323	29,58923	+	2_3	5	5	1	4,8
28,84999	29,16181			6	6	1	50
28,45846	28,02797			7	7	0	19,1
32,39971	32,94805			82	3	2	86,1

30,16651	29,49446			20	20	20	36,6
22,94437	23,02712			8	1	1	82,7
30,73651	31,21477			11	1	1	57,5
33,11114	32,84277			24	24	24	64,3
30,15279	22,7509			13	2	0	41,5
28,35001	28,34202			3	3	3	27
29,24386	28,63159	+	2_3	12	12	12	41

Unique + r	Unique sec	Mol. weigh	Q-value	Score	Intensity	MS/MS co	iBAQ 1-1	iBAQ 1-3
75,3	75,3	24,409	0	97,144	1,29E+10	56	72499000	1,6E+08
64,6	21,4	51,621	0	323,31	2,44E+10	173	1,75E+08	1,43E+08
31,7	6,7	58,536	0	132,71	9,64E+09	86	53050000	60895000
34,5	34,5	51,267	0	187,1	1,04E+09	26	589190	5860500
39,6	37,7	58,826	0	285,48	1,13E+09	36	7393700	413300
45,3	9,3	62,378	0	323,31	6,27E+10	186	4,88E+08	2,73E+08
40,4	40,4	62,129	0	323,31	1,21E+09	33	57599	1043400
37,7	35,4	48,105	0	292,46	3,9E+09	76	18414000	28835000
28,6	28,6	11,29	0	91,897	8,87E+08	19	0	100850
18,8	18,8	26,793	0	4,8618	17190000	16	64148	44043
26,5	4,4	82,577	0	195,5	1,26E+10	179	54165000	12210000
5,1	5,1	94,411	0	5,6156	61725000	10	111380	169530
51,8	51,8	214,84	0	323,31	1,21E+11	873	1,62E+08	41900000
18,2	5,7	32,316	0	18,095	3,54E+08	21	224290	69058
63,5	12,3	42,026	0	299,05	6,5E+10	215	4,69E+08	1,99E+08
5,9	5,9	57,896	0	5,1365	40824000	8	31181	10329
12,3	12,3	19,793	0	2,4636	52130000	8	452110	0
39,2	39,2	399,73	0	323,31	6,03E+10	783	50947000	19382000
22,7	1,5	58,397	0	50,188	1,25E+09	50	1198200	736720
33,2	33,2	104,85	0	182,3	3,99E+09	98	9785600	10522000
6,2	6,2	116,43	0	15,039	3,87E+08	18	871770	230690
39,7	39,7	13,89	0	101,98	1,68E+10	52	3,75E+08	1,23E+08
30,8	28,4	37,371	0	10,842	9,27E+08	27	8190000	9313400
11,3	11,3	47,984	0	2,6777	2,77E+09	7	23685000	17189000
20,3	20,3	74,823	0	15,573	1,83E+09	39	3527200	1954600
14,9	14,5	275,66	0	134,65	2,73E+09	111	2464100	2379800
29,8	29,8	41,174	0	13,75	1,99E+09	32	16969000	32253000
7,4	7,4	129,83	0	7,7814	3,1E+08	11	101600	915650
31,7	31,7	32,212	0	108,45	2,79E+09	30	31201000	18398000
25,6	25,6	28,671	0	92,323	4,49E+09	38	33758000	15949000
43,9	0,9	108,64	0	311,9	6,94E+10	333	2,32E+08	91592000
20,9	20,9	43,059	0	25,883	1,15E+09	14	25800000	0
18,4	18,4	30,497	0	31,254	6,3E+08	30	964530	11473000
43	43	24,96	0	187,45	4,25E+09	58	64130000	10672000
51,5	47,9	36,688	0	145,06	8,98E+09	81	89583000	18618000
10,8	10,8	41,326	0	4,3518	1,84E+08	10	126200	949770
12,3	12,3	41,428	0	9,2444	3,64E+08	10	1428400	864140
14,8	14,8	62,965	0	23,956	6,21E+08	21	0	0
21,6	20,5	187,15	0	220,13	1,57E+09	99	227840	107200
83,2	83,2	16,057	0	60,299	2,01E+10	126	3,41E+08	2,58E+08
50,7	50,7	15,799	0	135,39	9,29E+09	132	2,94E+08	1,11E+08
15,4	15,4	104,75	0	92,771	4,66E+09	75	14286000	13547000
29,8	29,8	10,16	0	89,507	5,61E+10	478	2,06E+09	40180000
30,8	30,8	11,765	0	10,41	66256000	18	1266400	2177600
11	11	53,224	0	6,1669	4,54E+08	15	5082400	3095100
17	17	25,331	0	251,01	1,1E+09	30	26111000	1344800
24,1	24,1	74,139	0	55,115	3,22E+09	79	35741000	5348700
46,7	15,5	36,154	0	269,99	1,65E+10	146	97461000	12038000

1,4	0	272,32	0	52,354	1,58E+08	36	81907	203620
6,3	6,3	38,797	0	15,898	31980000	11	288510	0
86,4	2,7	77,063	0	323,31	8,7E+12	4262	2,62E+10	2,22E+10
29,7	29,7	51,676	0	36,438	3,51E+09	51	15293000	6793500
42	42	53,981	0	323,31	6,47E+10	186	1,07E+09	1,67E+08
34,2	30,9	45,26	0	52,492	5,88E+09	63	32043000	18202000
58,2	58,2	11,139	0	13,186	1,18E+08	12	655760	8601400
29,2	29,2	38,714	0	13,819	6,39E+08	30	4519500	649710
28,4	28,4	30,768	0	62,195	1,06E+09	39	11794000	6004700
32,6	24,5	66,11	0	323,31	4,02E+09	57	10021000	6480000
37,3	37,3	27,87	0	38,935	2,62E+09	45	63316	23093000
20,6	20,6	18,552	0	10,841	1,58E+09	19	62550000	14557000
47,5	46,3	84,818	0	323,31	2,7E+11	562	9,95E+08	4,66E+08
39,8	39,8	10,834	0	13,423	3,12E+09	30	32816000	61618000
39	39	46,611	0	119,95	1,95E+09	76	3136400	373360
39,6	39,6	45,059	0	127,92	2,24E+10	127	2,23E+08	20886000
15,6	15,6	49,757	0	14,252	1,03E+08	14	0	319500
37,4	37,4	11,665	0	5,8479	1,88E+08	16	4794900	1871800
10,9	10,9	88,414	0	13,238	7,89E+08	24	4905700	1217500
14,5	14,5	85,696	0	32,006	1,57E+09	42	6595000	2566100
44,7	44,7	13,21	0	24,415	1,31E+09	48	10455000	24562000
45,9	45,9	9,681	0	50,598	4,66E+10	33	1,25E+09	8,32E+08
51,6	51,6	47,168	0	201,88	1,16E+10	125	1,5E+08	13146000
15,4	15,4	67,285	0	236,24	2,66E+08	39	889080	2386100
27,9	27,9	29,464	0	87,992	4,77E+09	67	94962000	39584000
21,1	12,1	28,955	0	12,282	3,05E+09	20	2358100	0
40,7	40,7	37,157	0	219,27	6,22E+09	74	12932000	69736
35,8	35,8	37,294	0	196,48	6,44E+09	90	1,13E+08	60047000
39,8	39,8	57,116	0	185,88	1,13E+10	159	65829000	24810000
30,1	30,1	44,552	0	30,727	1,61E+09	49	25298000	13145000
65,2	65,2	38,604	0	316,74	1,02E+10	128	92081000	17935000
43,9	43,9	58,14	0	218,1	1,81E+10	133	84055000	39637000
16,9	16,9	25,5	0	7,43	6,56E+08	24	16929000	2931000
82,1	82,1	15,054	0	86,434	1,37E+10	72	3,27E+08	61809000
42,5	42,5	37,821	0	195,99	8,29E+09	87	32655000	78236000
36,5	36,5	198,04	0	323,31	3,76E+10	518	70880000	25573000
67,9	2,2	129,38	0	323,31	7,85E+11	1688	1,87E+09	1,15E+09
24,7	13,1	83,263	0	65,326	2,62E+09	73	9861500	1149200
54,9	54,9	47,472	0	221,05	2,57E+10	201	56633000	29258000
10	10	167,55	0	175,05	3,33E+09	86	10641000	1712800
7,7	7,7	157,71	0	17,316	1,41E+09	56	1046700	500800
15	13,1	53,683	0	6,8444	1,57E+08	12	1028000	0
36,2	36,2	35,936	0	24,174	3,46E+09	64	22675000	15380000
25,9	25,9	19,48	0	14,533	1,6E+08	25	286380	1611900
45,8	45,8	11,301	0	50,679	9,18E+08	36	4136500	37048
60	60	14,716	0	91,859	5,69E+09	40	35649000	3951300
49,5	49,5	34,632	0	110,35	2,06E+10	148	2,45E+08	1,22E+08
15	15	57,519	0	7,4178	1,92E+08	16	0	0
61	61	147,33	0	9,749	7,98E+08	21	1586300	116090
9,1	9,1	30,992	0	2,8125	71957000	14	1216100	501650

12,6	7,6	70,108	0	24,943	1,03E+08	17	95421	130300
57,7	57,7	16,837	0	49,202	2,96E+09	42	13041000	35847000
20,2	20,2	211,68	0	110,05	4,69E+09	150	12781000	5521300
32,4	32,4	11,737	0	9,2184	2,13E+09	33	78400000	39687000
45	45	72,332	0	232,6	1,97E+10	192	81451000	57684000
48,3	48,3	174,01	0	323,31	4,34E+10	395	81565000	25649000
28,5	24,3	70,897	0	95,29	6,82E+09	84	58448000	3691400
11,1	11,1	108,34	0	106,17	1,71E+09	49	3170500	255080
45,9	36,7	103,06	0	246,79	2,14E+10	294	60622000	45274000
17,3	17,3	83,822	0	53,973	1,36E+09	74	7130600	2397500
10,4	10,4	25,673	0	46,705	2,25E+09	21	19435000	8106900
15,2	15,2	95,337	0	17,914	4,32E+08	31	1411300	452630
23,4	23,4	72,932	0	26,028	2,34E+09	43	7107400	7619200
25,6	25,6	18,108	0	3,2074	9,67E+08	8	31137000	2594300
10,7	8,6	70,289	0	49,458	1,44E+08	16	321640	435020
36,9	36,9	14,529	0	11,593	2,24E+09	36	48669000	23314000
8,2	8,2	15,523	0	7,339	8,77E+08	12	7983200	0
18	18	122,02	0	73,637	2,05E+09	74	2874900	72081
16	16	57,936	0	6,1243	6,01E+08	19	733900	0
22,1	7,7	92,339	0	47,732	2,33E+09	52	16121000	2518100
15,5	15,5	39,579	0	23,514	2,55E+09	7	33801000	165960
26,9	26,9	16,729	0	13,145	12704000	11	100530	0
6,1	6,1	65,568	0	4,645	1,59E+08	11	27737	28526
24,2	24,2	27,895	0	127,02	1,66E+09	84	17222000	5135500
35,2	35,2	20,195	0	36,023	2,77E+08	23	1245600	1639600
6,6	6,6	81,537	0	11,376	1,74E+10	43	72686000	63612000
17,7	17,7	106,78	0	51,324	1,47E+09	59	6993100	634840
51,3	51,3	20,838	0	120,62	2,01E+09	73	40160000	12528000
9,4	9,4	116,74	0	15,067	1,84E+08	21	7116,7	45561
26	26	28,804	0	45,273	6E+08	26	0	6306000
65,3	65,3	34,803	0	323,31	6,47E+10	239	6,73E+08	2,1E+08
21,4	21,4	12,269	0	4,7231	1,89E+08	9	0	0
20	20	23,787	0	40,708	6,22E+08	10	4803700	388220
15	15	277,5	0	47,411	1,13E+09	69	869610	274400
53,4	53,4	14,374	0	29,647	1E+10	38	95637000	32647000
17,7	17,7	41,654	0	53,085	1,64E+09	15	12191000	0
29,8	29,8	15,906	0	38,944	3,47E+08	25	3365700	2167200
13,5	13,5	86,551	0	43,375	7,7E+08	41	4116200	399030
31,8	6,4	30,137	0	19,122	4,56E+09	37	53133000	44254000
70,8	46,1	9,8875	0	57,927	9,4E+09	36	1,37E+08	5,28E+08
23,6	23,6	27,934	0	72,268	3,57E+09	35	77047000	360870
30,9	8,7	70,154	0	67,45	7,24E+09	83	33291000	12880000
19,5	19,5	119,09	0	76,539	1,35E+09	63	3356300	677960
33,2	33,2	22,742	0	40,946	9,11E+09	63	1,15E+08	48321000
38,2	38,2	22,728	0	84,366	8,11E+08	41	1048000	659540
1	0	240,85	0	8,211	1,33E+08	13	97468	160780
5,9	5,9	337,08	0	41,513	1,03E+09	34	237820	160850
21,4	21,4	27,647	0	6,5667	4,4E+08	13	182900	13376000
26,8	26,8	21,364	0	4,6214	75850000	12	285670	217270
2,7	2,7	81,996	0	4,2977	5,09E+08	7	1133600	804460

19,1	19,1	67,819	0	20,715	1,83E+09	28	673190	378960
56,2	56,2	46,918	0	154,86	3,41E+10	164	2,46E+08	1,94E+08
14,1	14,1	45,421	0	6,8391	2,13E+08	8	9112400	44635
12	12	26,425	0	6,001	47823000	6	1015500	284770
46,5	46,5	63,544	0	263,9	5,14E+10	181	3,42E+08	1,85E+08
46,9	46,9	11,117	0	49,284	1,11E+11	70	3,71E+09	2,29E+09
18,5	18,5	54,305	0	10,937	2,61E+08	8	1329200	457450
25,8	25,8	67,877	0	41,025	2,24E+09	49	10391000	13131000
24,1	24,1	21,057	0	7,7377	9,21E+08	21	15632000	7729400
25,8	21	54,102	0	37,765	2,89E+09	64	18071000	5014800
6,9	6,9	98,336	0	57,528	1,7E+09	18	2439600	936830
32,7	32,7	16,545	0	83,634	1,09E+10	41	1,97E+08	29201000
17	17	16,849	0	3,6318	22655000	11	1840300	106640
25,4	25,4	27,85	0	49,181	1,03E+09	25	3581700	677050
59,3	49,2	27,774	0	215,13	2,23E+10	164	1,68E+08	80143000
41,9	41,9	11,74	0	13,605	5,67E+09	49	1,68E+08	58536000
4,1	4,1	138,91	0	11,174	99376000	11	709760	0
39,6	39,6	61,68	0	48,744	7,68E+09	132	35864000	11411000
4,9	4,9	53,119	0,007813	1,5315	1,76E+10	10	87305000	1,11E+08
21,6	21,6	129,99	0	141,34	4,38E+09	75	12295000	1243100
8,7	8,7	314,77	0	58,895	2,49E+09	80	3768900	1025300
21,5	21,5	226,53	0	277,72	6,5E+09	134	8367300	11191000
9,6	4,5	42,1	0	23,912	3,13E+08	26	2549200	869580
52,9	52,9	21,086	0	46,253	1,65E+09	55	15973000	17529000
6,7	6,7	135,51	0	79,196	2,89E+09	58	9983300	823220
20,7	20,7	36,426	0	23,244	7,43E+08	26	11018000	8268400
8,3	8,3	107,54	0	9,187	50587000	16	75691	163590
9,6	9,6	29,295	0	13,229	36130000	13	194420	82660
25,7	25,7	19,365	0	17,92	1,25E+09	12	0	0
4,8	4,8	273,42	0	11,931	2,09E+08	14	370200	158520
20,5	20,5	46,943	0	62,298	1,45E+09	20	8382800	14420
24,1	24,1	23,115	0	7,4712	6,13E+08	21	2225700	934220
15,9	15,9	67,404	0	49,821	2,18E+09	33	1943300	9855400
36,3	36,3	21,517	0	13,334	80023000	20	383350	290690
21	21	36,923	0	31,222	1,28E+09	33	9219400	3546100
9,7	9,7	51,853	0	5,2514	3,99E+08	5	9752800	3496400
12,4	12,4	17,444	0	51,051	4,71E+09	38	3,59E+08	28579000
29	29	54,739	0	63,493	2,43E+09	78	5752400	150420
13,2	13,2	71,065	0	7,7924	36665000	13	55056	100110
25,8	25,8	20,7	0	11,647	2,9E+08	17	7189200	2780300
3	3	195,98	0	5,6753	24215000	19	14529	2561,9
4,5	4,5	73,786	0	5,2505	1,18E+09	11	8541600	2047500
21,8	21,8	17,963	0	22,148	32376000	13	358710	233820
52,6	52,6	22,871	0	233,96	6,11E+09	70	47126000	43673000
27,7	27,7	16,645	0	6,3143	3,04E+08	10	933470	744580
43,7	43,7	13,714	0	140,96	2,01E+11	100	2,73E+09	7,9E+08
60,3	60,3	16,584	0	157,16	5,94E+09	75	1,54E+08	91582000
40,5	36,8	28,302	0	93,449	9,49E+08	45	4728000	1208100
49	49	29,174	0	81,485	2,29E+09	60	23407000	7546000
70,5	70,5	5,0526	0	70,109	1,73E+09	26	2,51E+08	25921000

38,8	38,8	11,314	0	44,884	1,23E+10	45	5,87E+08	2,47E+08
53,9	53,9	18,012	0	26,594	1,78E+08	42	989130	664610
41,7	41,7	11,951	0	14,532	1,37E+09	28	6076500	18143000
49,4	45,3	27,745	0	106,42	1,49E+10	87	74083000	51614000
61,6	5,1	37,348	0	122,74	1,67E+10	148	95256000	42202000
50	50	5,0256	0	14,666	4,96E+09	9	3,79E+08	62006000
17,1	17,1	55,394	0	28,686	3,58E+09	38	14707000	10814000
30,1	30,1	133,8	0	279,35	2,58E+10	168	73337000	29399000
31,3	31,3	22,588	0	13,402	44157000	17	369740	405650
16	16	40,368	0	40,17	7,29E+08	18	1903000	277100
5,7	5,7	126,51	0	24,245	7,95E+08	20	2787600	188930
21,7	21,7	38,006	0	39,377	3,04E+09	49	23799000	7785700
46,6	13,2	19,202	0	84,558	3,47E+09	35	37246000	61871000
5,5	5,5	70,169	0	9,1631	8,52E+08	16	1516000	725840
14,1	14,1	295,22	0	323,31	7,67E+09	103	6780300	10068000
15,5	15,5	79,398	0	10,877	3,16E+08	11	2991600	274270
52,5	50,8	53,879	0	323,31	3,09E+10	218	1,29E+08	17027000
34,2	34,2	81,979	0	323,31	2,81E+10	245	1,37E+08	43015000
39,2	35,7	22,11	0	48,825	8,22E+09	59	46930000	57910000
7,2	7,2	96,792	0	74,085	1,2E+09	34	3371300	3494100
11,9	11,9	101,8	0	46,993	2,02E+09	27	4384600	2244400
49,7	49,7	65,33	0	323,31	1,23E+11	243	2E+08	59886000
14,8	14,8	60,793	0	53,174	9,6E+08	35	5597000	25649
35,6	35,6	46,231	0	107,1	4,97E+09	88	27149000	12938000
58,4	58,4	34,985	0	175,99	3,66E+10	166	1,12E+08	53009000
15,3	15,3	111,87	0	40,253	1,86E+09	33	9239900	5783600
28,2	28,2	43,078	0	21,772	1,42E+09	34	22682000	318850
18,4	18,4	84,364	0	71,859	1,91E+09	53	989460	1780200
12,7	12,7	36,728	0	2,525	3,23E+08	7	0	0
11,3	11,3	69,323	0	14,499	1,31E+09	16	10780000	1747700
56,1	56,1	129,57	0	323,31	2,13E+10	348	41793000	53764000
61,7	61,7	130,97	0	323,31	3,73E+11	747	9,16E+08	3,04E+08
17,7	17,7	97,539	0	105,58	1,73E+09	58	3312800	1348600
22,3	22,3	59,589	0	24,957	9,8E+08	49	6123200	2927000
34,6	34,6	26,264	0	210,73	6,09E+10	166	1,24E+09	3,29E+08
11,9	11,5	189,24	0	122,13	4,78E+09	82	6085800	1337000
20,4	20,4	47,837	0	23,371	5,8E+08	25	6615500	2035400
3,9	3,3	512,6	0	16,414	2,28E+08	24	145970	34076
13,3	13,3	26,697	0	4,324	8,07E+08	15	12477000	4058600
21,4	21,4	83,073	0	78,704	1,26E+09	62	440140	182870
41,3	41,3	74,68	0	323,31	3,07E+10	260	2,4E+08	1,12E+08
56,4	56,4	29,13	0	309,45	4,96E+10	175	3,44E+08	2,37E+08
47,2	47,2	60,673	0	221,31	9,75E+09	204	33085000	11616000
20,1	20,1	52,276	0	16,912	4,77E+08	33	3097400	2109200
29,9	29,9	40,876	0	39,226	6,46E+09	63	26180000	6088600
45,6	45,6	190,46	0	323,31	5,7E+10	534	63105000	70025000
13,7	13,7	110,11	0	57,344	1,24E+09	31	4179400	1404600
23,3	9,3	30,492	0	9,3771	37428000	6	520210	371950
8,4	8,4	37,106	0	3,1167	4,69E+08	15	1428400	0
10,7	10,7	43,235	0	7,9497	7,18E+08	19	0	0

17,4	17,4	250,53	0	117,47	4,52E+09	135	9374900	102170
17,9	17,9	159,69	0	141,77	1,87E+09	82	3631400	4628600
11,5	11,5	57,929	0	5,5525	49544000	11	61890	0
21,5	21,5	54,498	0	43,512	2,24E+09	50	6513900	610540
15,9	15,9	130,93	0	106,13	6,76E+09	78	13404000	2328500
20,8	20,8	100,16	0	110,82	1,7E+09	35	13206000	3207300
42,2	42,2	74,285	0	323,31	3,73E+10	210	2,28E+08	45913000
13,7	13,7	71,819	0	21,691	49831000	18	57178	43985
25,6	25,6	24,68	0	25,144	4E+08	41	2347200	5327500
18,2	18,2	165,27	0	139,2	2,04E+09	84	6033500	3353800
29,4	29,4	51,286	0	275,36	6,29E+09	60	36163000	13834000
7,9	7,9	144,37	0	15,203	91054000	25	40472	2135,8
14,8	14,8	134,55	0	37,994	3,02E+09	58	6407300	1396500
12,5	12,5	16,003	0	10,168	6,84E+08	16	13945000	14251000
26,5	26,5	19,891	0	1,9773	6,06E+08	7	14385000	10464000
16,8	16,8	47,071	0	40,784	6,02E+08	30	15576000	1127500
21,2	21,2	324,57	0	114,5	6,01E+09	170	7573900	8479800
28,4	28,4	85,206	0	183,26	6,39E+09	115	11127000	2828300
34,1	34,1	13,941	0	7,3546	98880000	17	457540	529600
37,1	37,1	23,031	0	11,535	3,44E+09	24	4439100	2042900
36	36	48,807	0	65,254	3,1E+09	74	7718800	1785000
74,2	74,2	10,438	0	14,13	2,16E+09	22	72547000	26657000
10,1	10,1	81,975	0	48,042	7,82E+08	23	2081800	55795
5,5	5,5	281,52	0	15,522	3,09E+08	20	131140	794250
27,5	27,5	83,432	0	92,302	2,46E+09	69	8607800	13751000
9,7	9,7	98,555	0	24,87	2,07E+09	43	859650	4680400
43,1	2,6	38,39	0	226,3	7,48E+09	127	29603000	7991300
15,2	15,2	33,868	0	70,212	3,58E+09	24	81727000	33833000
17,4	17,4	35,634	0	13,115	2,33E+08	21	4258900	6014000
7,1	7,1	142,34	0	72,862	4,15E+09	33	11716000	2358300
25,6	25,6	26,671	0	139,34	3,39E+09	29	21554000	25555000
39,7	39,7	24,341	0	52,488	1,61E+09	32	28141000	33562000
16,5	16,5	81,196	0	89,655	1,64E+09	33	20461000	2345900
11,6	11,6	94,75	0	31,508	7,01E+08	23	688780	5539500
17,9	17,9	32,227	0	5,4462	2,75E+09	22	10291000	41498000
62,5	0	259,21	0	323,31	2,36E+12	2794	2,17E+09	2,96E+09
34,8	34,8	24,093	0	11,912	6,03E+08	32	722330	1656600
43,7	0,5	240,91	0	323,31	1,33E+10	363	12119000	10214000
36,7	16,3	464,01	0	323,31	9,26E+10	944	64082000	28890000
20,3	11,6	14,813	0	41,996	6,83E+09	16	22583000	0
26,5	0	40,853	0	43,39	2,7E+09	34	0	0
56	0	31,468	0	94,489	2,75E+09	51	10619000	40222
ɔmo sapiens OX=9606 GN=HLA-A PE=3 SV=1;tr|A0A0U5PUM0|A0A0U5PUM0_HUMAN MHC class I a								
6,1	0	21,216	0	6,2742	8,31E+08	8	3560700	839600
1,8	0	239,62	0	3,6111	1,24E+08	16	129590	219870
36,4	35	76,613	0	102,9	6,63E+09	108	572730	319880
4,8	1,2	99,978	0	22,836	6,46E+09	38	29561000	133310
50	9,6	17,471	0	26,583	4,16E+09	55	57192000	20338000
19,1	0	61,94	0	17,079	2,9E+09	37	21728000	24443000
4,7	1,9	74,831	0	323,31	6,66E+10	267	2,63E+08	1,09E+08

36,6	36,6	71,553	0	77,483	8E+09	128	71519000	22670000
20	20	7,99	0	16,266	6,66E+08	6	29338000	18754000
7,5	7,5	14,691	0	25,049	1,19E+10	30	2,77E+08	2,32E+08
64,3	64,3	46,924	0	323,31	7,22E+10	229	2,83E+08	20022000
7,7	0	38,865	0	26,998	1,99E+09	17	0	0
27	27	12,759	0	5,4929	1,72E+09	8	9018300	39553000
41	41	38,069	0	64,295	2,72E+09	54	13368000	2663600

iBAQ 1-5	iBAQ 2-1	iBAQ 2-2	iBAQ 2-3	iBAQ 2-4	iBAQ 3-1	iBAQ 3-2	iBAQ 3-3	iBAQ 3-4
1,82E+08	1,05E+08	1,32E+08	91384000	1,47E+08	60927000	48299000	59657000	1,09E+08
2,19E+08	40525000	34509000	46834000	41358000	6615700	3411300	7267300	1,24E+08
97959000	7555900	4620300	20505000	16035000	28207	21636	24610	71577000
27575000	210850	461720	8027,8	43448	0	0	0	1083900
31740000	916690	25936	172870	519910	258390	33832	1652600	318810
4,03E+08	65898000	50950000	1,09E+08	1,12E+08	11830000	4731800	3983700	5,01E+08
35007000	4229200	177750	23580	3145600	31924	4447,6	2518700	164010
49636000	4218700	3013200	5472900	3077900	103020	989190	26555	20837000
114900	111040	83312	21802000	25206000	29277000	29182000	20714000	88344
78521	64080	139190	143620	124760	89228	164760	177490	56133
29242000	39609000	32490000	17230000	26972000	24950000	20929000	14283000	8368400
0	230370	126850	164700	135830	159120	179900	151870	334010
52278000	1,62E+08	1,32E+08	81273000	82041000	2,05E+08	1,59E+08	1,5E+08	1,14E+08
15244000	0	7053000	710930	3327300	584480	3818100	535120	639020
2E+08	2,18E+08	1,91E+08	2,59E+08	2,69E+08	1,77E+08	1,89E+08	1,61E+08	1,68E+08
23838	646900	239000	8401,8	9439,1	223790	174350	122860	21909
402960	2153600	957970	499750	324580	135480	397720	275690	192390
13930000	36281000	28205000	20357000	22626000	51405000	40424000	34476000	16936000
7473700	6273400	6123300	4653000	5297700	7930100	3129100	5662800	5957500
13357000	10353000	4761500	3139500	1882700	4061700	2044300	3612200	8934000
0	1967000	1524400	0	4279,3	1223100	902590	1676000	13886
27551000	2,16E+08	1,35E+08	1,74E+08	1,39E+08	3,79E+08	3,46E+08	3,5E+08	1,36E+08
2033800	3624300	2735800	2599600	2297300	400020	8990700	4583100	6714600
39423000	14621000	14565000	7251600	14615000	0	9435800	0	12914000
639750	6368500	5430700	1430400	2615000	11815000	9264400	10239000	2180000
2381400	3688500	1138000	2197900	677430	1350200	391630	741190	1984800
18283000	4233900	650300	226370	3595400	58313	0	0	10344000
996690	600200	469310	588320	22679	631890	675290	767890	314280
10513000	204400	19545000	13218000	7775800	34127000	30843000	47751000	39640000
12031000	15576000	17121000	28386000	28861000	72395000	66779000	53269000	926910
1,05E+08	2,35E+08	2,02E+08	74989000	1,19E+08	2,89E+08	1,86E+08	1,94E+08	2,01E+08
287240	791970	1207000	0	37384	9170000	7950400	5776700	1162200
2702800	3771500	4837300	6209300	4407500	76335	521850	105240	4324600
29487000	44635000	35712000	14131000	16104000	63096000	31838000	35204000	41604000
50316000	49505000	39921000	33384000	39247000	45506000	45180000	35977000	51711000
1059000	832450	469850	1629300	1511600	147640	128010	32707	1121900
528490	1602400	1269100	1637300	1446200	1718100	1045700	1251000	1190500
0	9343,1	141610	9715,8	686790	5932600	4764000	7840100	24739
403000	2500100	1334000	1378500	1308900	3850300	1884600	2576400	658530
1,55E+08	1,92E+08	2,04E+08	99544000	77229000	4,11E+08	2,89E+08	2,88E+08	2,03E+08
1,11E+08	1,08E+08	1,19E+08	1,06E+08	82367000	81481000	99246000	1,37E+08	79296000
14854000	8103400	6415800	4846500	8272300	4474100	4081400	2620200	9955200
34340000	3,36E+09	4,73E+09	1,05E+08	3,91E+08	2,37E+09	3,45E+09	2,13E+09	54176000
3699900	167900	187620	730820	669660	783590	644580	639950	2283300
1324400	2133900	3518100	1255100	137430	0	2258900	0	97747
2116500	16270000	5176900	6898000	6057200	18663000	10432000	25804000	3824200
3519900	7383300	3734000	3580000	3516900	3812000	4163400	3820100	3919300
38363000	96996000	89981000	48083000	39776000	1,26E+08	98471000	67365000	36460000

215050	82966	118100	209690	252820	97615	131470	104580	68452
0	384920	256860	0	0	219380	246270	203040	0
1,64E+10	2,1E+10	2,18E+10	1,47E+10	1,69E+10	1,81E+10	1,47E+10	1,73E+10	2,28E+10
11786000	2062800	20092000	21291000	937280	15160000	13408000	15271000	30671000
1,59E+08	3,83E+08	5,26E+08	1,52E+08	1,34E+08	1,12E+08	1,32E+08	40595000	65369000
13949000	36794000	31254000	25819000	20322000	22993000	19090000	21822000	13156000
2872900	919430	412820	2399700	3166900	107420	157110	117220	291650
498610	5557800	3948700	1168700	1162100	4346800	3264600	5956900	859320
15815000	13400000	5847300	1085600	1082200	10154000	8089200	11397000	4013100
93590000	5586300	1520900	1372100	2987600	199960	0	2127200	5784700
54993000	18533000	9151300	10682000	10186000	15632000	14026000	43835000	1177000
3650900	35490000	20408000	8419600	8554800	14660000	15677000	12225000	1850700
6,06E+08	1,01E+09	8,81E+08	4,96E+08	5,35E+08	9,67E+08	8,24E+08	7,77E+08	6,33E+08
36601000	12336000	12853000	1,1E+08	43006000	17690000	8951600	24023000	29304000
4899900	37985000	38978000	131790	148490	4619400	5197000	12166000	6786300
17827000	72562000	87642000	6827700	4905300	2,53E+08	1,85E+08	1,89E+08	56359000
72864	310110	507650	342760	145580	816210	661340	722970	1255800
4854700	4967600	1954400	1952600	3393500	5390100	525350	3756900	4089900
1397300	4730900	2081000	989720	460010	1671600	1966200	2068000	1717600
1931500	3552300	1911800	3709800	4191900	5640800	2131000	5751900	8249900
22127000	6350800	7001900	14533000	15337000	22374000	11025000	6624900	22859000
4,36E+08	9,68E+08	7,57E+08	1,3E+09	9,24E+08	7,15E+08	4,39E+08	5,81E+08	1,12E+09
71416000	72631000	54177000	24272000	10427000	54317000	27292000	42971000	5229700
1024000	995490	393310	1554500	256150	141220	213180	318400	994590
29321000	47318000	30098000	36169000	21226000	23021000	9606500	28188000	37924000
0	20937000	13857000	32459000	26807000	26709000	25082000	22270000	9153000
5355200	56101000	32330000	890400	2406500	85524000	33565000	50027000	3390700
47984000	33719000	11325000	13995000	28474000	15008000	19226000	12486000	23964000
14943000	36456000	27635000	24211000	18630000	26005000	13553000	15377000	56741000
9474200	4466100	4969200	4706900	4571000	4657000	4688800	3378700	5604800
13821000	68859000	48922000	29689000	32317000	35765000	31455000	26640000	44134000
34779000	64872000	41657000	19132000	85064000	49624000	42816000	45368000	59032000
9935300	14082000	4720400	11029000	7304500	7058600	7131500	5887300	6701100
47917000	1,37E+08	84217000	1,63E+08	1,09E+08	1,79E+08	1,55E+08	1,34E+08	1,26E+08
41287000	34238000	22387000	56231000	97100000	37127000	26358000	3075000	7745900
26884000	51580000	46058000	26581000	32880000	80598000	61567000	61938000	23160000
1,09E+09	8,96E+08	1,03E+09	1,09E+09	1,17E+09	1,39E+09	8,77E+08	1,22E+09	8,8E+08
3996500	10678000	7514000	7969000	7600600	8394300	6119500	4601800	11384000
30019000	65485000	89792000	52195000	55961000	1,92E+08	1,2E+08	1,97E+08	1E+08
2335900	1838500	1734200	4253600	4157300	3882100	3745000	2287600	2609700
1110300	6232000	874710	530190	583580	1026300	6464700	478740	1867200
0	2524300	485520	134320	215520	44103	153150	32416	0
17152000	23998000	13087000	14632000	14478000	7597800	4544800	5377200	18418000
2166900	2997500	333290	1959700	1475600	1963800	2219100	2752000	2278200
643540	15559000	11083000	4065100	2373300	13966000	67739000	6657100	4849400
5049200	48052000	69258000	42977000	22012000	1,67E+08	79332000	1,52E+08	6511000
99425000	1,95E+08	2,17E+08	1,1E+08	55882000	1,5E+08	84413000	2,42E+08	65920000
0	1918000	1511600	552490	640380	1071300	1375000	792110	150430
903880	1206500	353830	152650	428070	988020	1181700	299400	2060300
224780	984650	100190	668190	437400	996490	2071600	1760900	32627

0	539650	165920	312850	164500	28977	82198	1649000	56252
49852000	55434000	37191000	16565000	16506000	30389000	31779000	19848000	22718000
1886200	3050900	3429400	2659200	2091000	4235900	3263200	4408800	3995900
30113000	46096000	44319000	2552800	34726000	21436000	687200	1394500	5528800
15875000	59735000	43419000	42222000	43375000	76475000	68665000	65412000	1,03E+08
24177000	48974000	51668000	42281000	45663000	74211000	75251000	77066000	39901000
3131100	14883000	17426000	16066000	11594000	36215000	7593400	19921000	17679000
398550	1362900	584540	2554100	1024400	5081000	9786300	6024300	2069500
47336000	43580000	30528000	31958000	20996000	25448000	27765000	10688000	67320000
4628100	4498000	7374400	3586200	5312300	5616300	5550400	5534600	7339800
4885900	23180000	4513400	24004000	17549000	64303000	44772000	33758000	5754600
2615000	991100	408750	359690	243830	345670	175420	790890	1808100
3975000	8148100	3356300	1891400	3577800	3188300	7263400	6039700	9536400
5614500	4036800	13823000	13295000	1016900	31728000	25435000	4436300	4996400
509920	1673100	350350	253220	301970	27477	10296	0	0
47341000	59775000	20268000	45713000	16222000	54921000	56264000	44206000	31483000
0	26785000	8278600	0	0	20994000	35778000	25478000	0
564620	2742100	951150	1184800	1042700	14862000	8506800	7509700	2337300
64855	1795100	4334600	229810	107300	3928000	4247600	2324300	432860
2354200	6287000	8251300	2771200	2881600	4306200	4876000	4217800	3785900
15350000	23824000	22523000	15958000	0	0	26174000	22580000	21862000
0	235270	204010	117010	93420	221170	313830	216280	86530
15951	699830	50694	543750	221810	57689	31837	837520	2973200
19608000	24480000	21652000	2876400	3734300	31635000	9583700	15300000	33276000
232680	886330	483090	178550	1638200	11482000	1409300	9706600	1869100
64845000	55280000	29663000	58580000	69350000	54209000	68709000	40992000	68165000
5683200	6872300	1801300	458650	1399800	1234300	1101400	2277800	2742400
19174000	32335000	18562000	25684000	21937000	20368000	22750000	20584000	17590000
245000	440740	95521	10158	68303	26037	11112	362620	1138400
5628800	2703100	2577600	5474900	3243100	9301700	2998400	5052100	2902200
1,97E+08	2,47E+08	1,82E+08	2,31E+08	2,42E+08	5,8E+08	4,18E+08	3,61E+08	2,5E+08
1063800	9016000	7426100	3955500	7096900	2074800	517490	0	362010
434000	12303000	23079000	3843400	4492300	11940000	431740	3274900	4103200
1250000	726740	334760	1807200	721410	794420	429520	106020	1253900
29674000	80375000	1,16E+08	42079000	81811000	1,9E+08	2,94E+08	3,28E+08	1,43E+08
13047	422500	51700	4837,5	34500	36906000	20182000	26490000	201000
3614400	7568800	5390300	2761500	3545000	4585800	9450600	5826200	9483400
2465300	6076100	3567000	955480	778580	1794700	2394200	1778600	1349800
4611800	25697000	7099600	1403200	30219000	26981000	11932000	3735300	31170000
1,33E+08	44361000	89486000	56402000	46069000	37914000	5106200	25516000	2,4E+08
2865700	6671000	973290	1618900	6551600	44378000	92984000	83365000	7422900
4528100	11167000	17090000	19611000	10555000	45536000	30795000	35279000	28766000
1863300	2148400	11368000	463350	294690	859240	207440	627230	552630
54653000	97647000	59853000	45166000	48324000	72823000	66026000	51302000	1E+08
711780	14984000	2980100	8875500	9888600	7429900	5712700	6512200	3582000
131200	26045	20893	155490	137210	92771	97682	80599	283420
114580	511800	515550	841040	596960	1513900	1613200	382330	370260
4186700	8284600	3395700	430240	2567300	288600	176800	0	955330
134240	84157	384880	267430	167620	78571	40390	1410100	3825100
0	2944800	2909900	600990	2468800	984840	2354600	0	1706800

850390	2653000	4397100	12391000	5459600	13679000	8532700	5094100	1362200
89312000	1,75E+08	1,39E+08	1,23E+08	1,2E+08	1,17E+08	97577000	1,15E+08	2,06E+08
17609	54107	1699900	897600	19699	6072,3	0	0	0
865890	939080	67069	33608	92605	237110	204290	169660	437960
1,04E+08	2,04E+08	2,28E+08	2,67E+08	1,77E+08	2,61E+08	1,31E+08	2,83E+08	1,52E+08
1,64E+09	3,16E+09	1,83E+09	3,26E+09	1,94E+09	3,29E+09	1,38E+09	2,63E+09	2,7E+09
585070	1175000	925850	603810	67625	3331200	2385300	2152100	54472
7034500	12217000	2674500	8963800	9819600	5250600	2834400	8120600	8996500
7730500	9172200	5510200	7147000	5013000	6152000	5342100	7172500	15451000
5143900	12384000	5910400	6025400	5157600	13138000	9538500	8454600	14261000
4478500	3657700	3046000	457790	1572500	6894300	14098000	7553800	3517800
50739000	2,22E+08	1,68E+08	52830000	38762000	2,59E+08	2,28E+08	1,97E+08	1,12E+08
549670	239930	730550	380080	375020	165840	173360	187520	914720
6440000	2250300	8533100	17587000	12336000	2962000	14845000	2730300	1560900
1,01E+08	1,54E+08	1,76E+08	68020000	1,05E+08	1,41E+08	1,21E+08	1,93E+08	85416000
41910000	99454000	1,08E+08	90809000	1,01E+08	1,14E+08	1,28E+08	1,21E+08	1,04E+08
0	0	0	0	0	919880	91628	84405	69351
11069000	21667000	30476000	19059000	16835000	37319000	25814000	31706000	33210000
1,34E+08	2,39E+08	1,61E+08	71774000	45707000	84781000	39352000	80158000	46462000
1423900	19339000	18337000	16780000	23172000	911920	1128200	1135700	1642200
1112100	1382700	682400	2302300	2577100	1542200	3030300	876070	123680
2997300	6878200	5493100	7455200	8357000	2349300	2498300	3578300	3349100
419210	1406200	748630	5639800	955750	214840	430000	552740	456350
1609100	2429400	1953900	32063000	31407000	14608000	9713000	5981700	4378200
1948900	1929500	1741400	879280	1289500	8670700	8390800	7596200	6608100
3462600	4445400	3489800	3876100	5970400	2786800	1432100	116750	4681100
143140	149220	273720	18487	93938	9996,6	109620	10666	28236
90297	261310	239340	336910	229650	240560	306520	317480	109540
105440	34258000	9122300	0	0	31512000	25213000	40969000	14786000
101120	136900	106230	69655	69513	172580	36870	260470	190810
181910	1416100	3324200	3688600	3941100	18496000	4072000	10159000	0
1706300	1617500	1001600	1201400	122270	1171500	1065600	9231700	30839000
15978	10850000	7294600	3097500	4566800	8477300	3311600	11317000	12047000
2188600	1023200	865370	948010	1120500	546660	680510	624150	1331900
10790000	2615500	12031000	409580	9383900	19149000	1070800	9065700	7795300
3898700	426320	0	239380	520440	662200	0	0	0
25231000	1,21E+08	83370000	16603000	17858000	1,4E+08	64703000	77697000	8362400
829560	31504000	14057000	3685700	2937800	18960000	17700000	18595000	1688900
39489	117180	160360	180700	115960	96832	63601	75511	42765
4583300	1771200	708920	423580	135310	1041400	2859000	1657300	5877900
0	33527	51927	17977	26782	29206	19278	49526	15066
2108400	3685500	1731300	1011400	2108400	6948300	2919600	3027700	3947300
171190	846360	958140	437690	461990	755570	834130	690680	726870
29990000	34102000	26451000	39245000	36520000	32588000	23544000	39796000	54007000
1439800	3694900	3112200	343170	380430	126050	195770	2566000	14129000
8,16E+08	4,44E+09	3,85E+09	3E+09	3,04E+09	3,67E+09	3,43E+09	3,97E+09	3,71E+09
51006000	46953000	35582000	27638000	29204000	29268000	35476000	45700000	47358000
1738300	20937000	1656400	12164000	1293400	471300	2559900	9277100	3266000
8671100	15272000	14231000	21665000	8360200	5621200	6073100	15873000	8153000
17469000	78262000	16449000	1,11E+08	94139000	61383000	57606000	89905000	60342000

2,74E+08	80414000	63561000	1,24E+08	1,64E+08	1,21E+08	2390300	40798000	3,53E+08
4368600	2286800	493440	2026700	1070600	1865300	1526300	2359100	182630
20475000	40651000	82397000	45655000	48278000	2749100	33811000	54151000	1,06E+08
64358000	1,26E+08	1,05E+08	1,03E+08	1E+08	1,47E+08	1,21E+08	1,1E+08	59192000
53292000	1,23E+08	76969000	68361000	56861000	95065000	1,11E+08	1,3E+08	27577000
2,66E+08	3,27E+08	3,29E+08	2,01E+08	1,52E+08	3,37E+08	1,21E+08	1,86E+08	1,22E+08
11216000	10729000	6854500	7890300	8859200	22486000	31864000	9694800	7987600
24071000	36136000	29285000	19162000	37122000	82755000	84533000	73413000	26313000
343140	951700	842810	444170	405350	250270	232330	198010	463180
0	6970200	1768200	1349200	785500	13768000	12798000	3233600	0
331050	1003900	1501600	1061100	1817800	3108300	983480	2412300	1370700
12470000	16906000	9237500	11047000	10283000	28313000	19470000	11638000	9270000
38834000	24155000	14584000	58162000	27051000	14253000	23726000	14500000	32792000
1262900	4497700	3815300	1429400	2603200	4132900	953940	4290500	3173600
10039000	1208500	963310	2047900	1612300	4468700	3812100	3891800	3642700
229220	55399	509900	186320	214500	1593000	365360	535810	386630
32548000	1,44E+08	1,37E+08	29788000	30909000	1,87E+08	1,77E+08	1,51E+08	71265000
73951000	1,26E+08	93276000	58259000	49557000	77373000	52941000	56885000	58555000
41721000	22917000	55426000	50588000	51872000	66047000	52326000	54662000	47693000
6041600	1539200	1123000	4216800	2592600	3228500	1910400	957120	2338700
3359300	8412800	5438900	6098600	4135700	4105700	5691200	4894700	2899900
37044000	6,17E+08	6,56E+08	3,42E+08	3,78E+08	8,27E+08	5,07E+08	6,61E+08	4,61E+08
12228	3302700	2706200	154290	101840	9393100	3185000	6156400	348900
4480700	35552000	46108000	43277000	22996000	22419000	18940000	21083000	6627200
89459000	2,27E+08	1,72E+08	80458000	1,9E+08	2,73E+08	77437000	2,05E+08	1,84E+08
10602000	682660	107950	3928300	1997000	157980	575110	13801	2641500
1647400	15260000	10844000	815300	6598700	553060	6074900	7715300	6578000
1436400	9821700	3074100	3137100	4096500	9289600	5015100	7267100	706370
0	0	12259	0	0	9337300	8358200	208090	46366
483740	7300200	6309000	0	0	8564500	4318000	4088100	0
22271000	22491000	15471000	50178000	46881000	24443000	19879000	19544000	22028000
1,39E+08	5,7E+08	5,05E+08	2,63E+08	2,72E+08	9,2E+08	8,35E+08	7,71E+08	5,14E+08
1596000	10125000	7702300	1641200	446320	5853600	5587800	3250500	1408100
2978200	3826700	3431600	1643600	1541200	2518900	1878200	447200	6480900
4,76E+08	8,1E+08	6,21E+08	2,57E+08	3,1E+08	8,77E+08	5,27E+08	5,37E+08	7,81E+08
1267800	2882900	6371400	4784800	4056400	15235000	4018500	21550000	2641200
1868100	4454000	2506500	2349600	3137800	3103200	1558800	1506700	5009900
90036	15394	329530	6993,4	4909,4	135270	54222	0	0
5629400	10354000	6730300	3115900	3156800	4972800	3287100	3246900	5035400
2008500	9008000	2518100	1584600	1880700	7710800	6280400	6192800	2710700
79332000	38383000	35204000	1,24E+08	93233000	64160000	79940000	1,1E+08	12496000
2,23E+08	3,06E+08	4,34E+08	2,56E+08	2,26E+08	3,54E+08	5,09E+08	3,99E+08	2,56E+08
17027000	50769000	43176000	10246000	12801000	34611000	22162000	38012000	41163000
1002300	2291100	298270	4330600	1230900	1098200	31634	2128700	45655
8828800	18841000	39162000	31162000	18598000	70020000	52721000	78125000	53814000
64480000	61455000	47967000	81579000	79961000	39820000	31420000	45717000	47305000
796200	11279000	1882200	2097100	2292300	1681000	1691800	1994000	2372100
627300	0	259280	0	106910	199590	134440	0	119610
606330	1428800	705030	385330	366140	6432200	5349700	7451000	1874600
1145700	3091000	7271200	23391	24282	5657100	14586000	4065100	33088

11247	2089000	4025700	2553100	389920	8701000	8097200	8049000	10453000
4508100	1681400	1779200	4359700	4883800	125270	1068700	781010	1739900
169680	356090	276600	30010	112440	333970	228640	180750	155470
666160	11817000	12244000	473290	507440	17952000	11642000	17567000	9736500
594070	19758000	19612000	11722000	4948700	14865000	27437000	21406000	10862000
2757500	3006000	1305800	131070	1043500	10562000	6756600	9589000	15967
46266000	1,76E+08	1,62E+08	38799000	44761000	1,91E+08	1,43E+08	77791000	12925000
139740	117150	68191	63067	54501	139180	112320	239270	431040
1388700	4070700	1956000	2171900	4670600	129090	1783300	217560	6742400
2400400	3246700	880040	2253100	2151800	2390600	1228800	1277400	2739700
8268700	40633000	37509000	16472000	19337000	50362000	39359000	30740000	21783000
21210	126400	78488	13311	4421,6	397330	213590	275180	296080
2275500	2467700	1610100	1462700	1821500	11595000	6207100	10618000	3689400
7526900	10733000	8014900	14101000	17800000	4814100	2890200	6051100	13808000
513650	931210	897700	6536200	7923900	400690	4095300	496770	3853400
1255100	1123100	1425600	1134700	658420	4209400	2148600	2700100	341170
4103600	1020700	491180	6317600	5408000	1910900	1219800	1699900	3490100
4598400	18907000	12056000	11895000	10116000	32147000	25670000	21787000	12655000
380930	394450	233370	333960	298120	154210	2507900	5769400	1300500
8304200	29925000	25528000	23943000	6387100	53006000	50748000	40329000	20034000
6558500	22155000	28806000	5749200	8895100	22967000	18273000	24302000	7720700
34223000	53591000	84912000	49173000	51800000	709250	56249000	73459000	36044000
407710	11692000	2004100	601500	533500	1029100	894900	1298100	1116900
180960	5026,6	71172	447070	489760	97372	93446	3427,8	325230
5932800	8989800	4447200	8801900	5473300	4462300	4106700	282780	1566800
2005400	3735400	3343100	4371100	2649200	1479700	2865200	8327400	6255300
18015000	42766000	51836000	25624000	15732000	49831000	47011000	44505000	41083000
29774000	19025000	14395000	11715000	20917000	29962000	29046000	32674000	22030000
2969700	211360	210550	320570	293730	95702	57033	50182	59157
3991300	19748000	8706300	3972100	3031300	555610	12290000	872070	8137400
25922000	16702000	78997000	40511000	42579000	848060	2288400	83522000	255490
7578700	2440000	4405000	5696500	4542000	13366000	7409900	7991700	8534100
1814000	3075400	199930	6636400	4079000	5406000	3141300	2235200	242170
1581800	0	122590	458910	9392300	0	0	0	2258000
9785800	13476000	17330000	15479000	20954000	4618500	13932000	12040000	12251000
1,03E+09	1,52E+09	1,4E+09	3,32E+09	3,3E+09	2,41E+09	1,93E+09	2,37E+09	1,15E+09
2198700	9511400	9987700	12240000	5983300	440490	4866300	4471200	2736900
6838400	2331300	2585400	22674000	23935000	7904300	7630300	4619600	25856000
19128000	57285000	51440000	34573000	34508000	67250000	62687000	56468000	32542000
17176000	2,09E+08	3,59E+08	33559000	14240	4,98E+08	8,76E+08	2,59E+08	1914200
0	22733000	25546000	16728	19658	31133000	41316000	29162000	229830
63748	22608000	38373000	133460	1171200	44600000	35582000	18577000	375850
ntigen OS=Hom								
831260	10998000	2704600	1052500	0	23233000	16291000	17599000	5953100
87889	73687	65350	251030	281800	45721	51231	38103	60169
32887	11261000	14385000	31679000	46276000	30816000	12062000	21324000	10422000
171330	17590000	16631000	12359000	19762000	13544000	7727300	6778300	7545500
15246000	58785000	96481000	42391000	17423000	99906000	79547000	82599000	24076000
1871000	9836600	6318200	5023200	4078400	3430000	9821800	17080000	91666
2,21E+08	2,64E+08	1,87E+08	77783000	96763000	1,29E+08	1,09E+08	70512000	1,38E+08

887020	19300000	10887000	4722400	8407000	19072000	28634000	32527000	10037000
17654000	34429000	9474100	6613300	4293100	27189	5624200	2548800	4417600
1,43E+08	2,12E+08	2,47E+08	2,1E+08	1,1E+08	2,03E+08	2,09E+08	2,3E+08	3,12E+08
46364000	6,46E+08	4,47E+08	2,12E+08	1,39E+08	3,49E+08	3,66E+08	3,06E+08	1,93E+08
0	1844100	7713300	0	670990	29065000	22905000	45292000	3167000
14813000	68825000	27235000	5365200	26930000	7431400	23949000	42821000	20424000
3127200	10215000	6946400	1788900	3031000	21819000	14093000	21828000	9978000

Student's T	Student's T	Student's T	Student's T	Majority pr	Fasta headers
0,017277	0,105191	0,734247	2,256997	CON__P00761	
0,193944	0,29702	1,965836	1,361167	CON__P02	;sp|P02533|K1C14_HUMAN Keratin, type I c
0,102059	0,171882	4,121507	1,842281	tr|B4DRU6	tr|B4DRU6|B4DRU6_HUMAN cDNA FLJ5465
0,607449	0,700106	-0,54043	-0,49246	sp|P08779	sp|P08779|K1C16_HUMAN Keratin, type I cy
0,317845	0,420865	1,21116	0,999376	sp|P13645	sp|P13645|K1C10_HUMAN Keratin, type I cy
0,159043	0,246	2,489075	1,510088	CON__P13	;sp|P13647|K2C5_HUMAN Keratin, type II cy
0,569681	0,675037	0,681096	0,552473	CON__P35	;sp|P35527|K1C9_HUMAN Keratin, type I cy
0,280215	0,346351	2,418231	1,131149	sp|Q04695	sp|Q04695|K1C17_HUMAN Keratin, type I c
0,628426	0,703668	-0,88977	-0,48213	tr|A0M8Q	tr|A0M8Q9|A0M8Q9_HUMAN C1 segment
0,432506	0,61436	-0,28643	-0,6502	tr|A0A1U9	tr|A0A1U9X8Y4|A0A1U9X8Y4_HUMAN Chlo
0,000428	0,0246	0,560557	3,11013	sp|O00391	sp|O00391|QSOX1_HUMAN Sulfhydryl oxida
0,20048	0,34614	-0,55955	-1,14399	tr|E9PFW2	tr|E9PFW2|E9PFW2_HUMAN Beta-mannosi
0,359581	0,650899	-0,15197	-0,60014	sp|O00468	sp|O00468-6|AGRIN_HUMAN Isoform 6 of A
0,561178	0,669371	0,725492	0,566887	sp|O00560	sp|O00560-2|SDCB1_HUMAN Isoform 2 of S
0,145399	0,332168	0,425902	1,201053	tr|Q6FI18|	tr|Q6FI18|Q6FI18_HUMAN CYR61 protein C
0,111575	0,302953	0,440625	1,309967	tr|A0AV88	tr|A0AV88|A0AV88_HUMAN Disintegrin and
0,127033	0,245079	1,015501	1,507656	tr|Q53HL1	tr|Q53HL1|Q53HL1_HUMAN Myosin regula
0,866847	0,942397	-0,02611	-0,10477	sp|O15230	sp|O15230|LAMA5_HUMAN Laminin subun
0,063834	0,186316	0,690444	1,707268	sp|O43278	sp|O43278|SPIT1_HUMAN Kunitz-type prot
0,958178	0,978061	0,021094	0,043416	tr|A0A0S2	tr|A0A0S2Z3G9|A0A0S2Z3G9_HUMAN Actin
0,371452	0,485786	-0,86027	-0,8683	sp|O60245	sp|O60245-2|PCDH7_HUMAN Isoform B of
0,187962	0,333311	-0,58646	-1,18526	sp|O60814	sp|O60814|H2B1K_HUMAN Histone H2B typ
6,37E-06	0	-4,80394	-11,2219	tr|B2R717	tr|B2R717|B2R717_HUMAN cDNA, FLJ93235
0,02611	0,0492	4,698645	2,762629	tr|B4DY05	tr|B4DY05|B4DY05_HUMAN cDNA FLJ58303
0,060415	0,167619	-1,06523	-1,89714	sp|O75326	sp|O75326|SEM7A_HUMAN Semaphorin-7A
0,198214	0,342656	0,565024	1,151586	sp|O75369	sp|O75369-2|FLNB_HUMAN Isoform 2 of Fil
0,094547	0,16693	2,337507	1,828464	sp|O75635	sp|O75635-2|SPB7_HUMAN Isoform 2 of Se
0,505694	0,614706	-0,75423	-0,64697	tr|B4DZ36	tr|B4DZ36|B4DZ36_HUMAN cDNA FLJ58441
0,00669	0,022824	-1,45239	-3,16983	tr|B3KNF2	tr|B3KNF2|B3KNF2_HUMAN cDNA FLJ14484
0,406069	0,543689	-0,5515	-0,76882	tr|I1W660	tr|I1W660|I1W660_HUMAN Dickkopf-like p
0,390859	0,650163	-0,16637	-0,5943	sp|O94985	sp|O94985-2|CSTN1_HUMAN Isoform 2 of C
0,213927	0,299962	-2,72098	-1,32237	tr|B3KQQ9	tr|B3KQQ9|B3KQQ9_HUMAN cDNA PSEC00
0,1033	0,174378	2,24843	1,768744	tr|Q5JPJ9|	tr|Q5JPJ9|Q5JPJ9_HUMAN Uncharacterized
0,320346	0,534369	-0,28117	-0,78192	sp|O95633	sp|O95633-2|FSTL3_HUMAN Isoform 2 of Fo
0,148895	0,39429	0,293211	1,058106	tr|V9HWB	tr|V9HWB9|V9HWB9_HUMAN L-lactate deh
0,112599	0,186146	1,541084	1,657767	tr|B3KUZ8	tr|B3KUZ8|B3KUZ8_HUMAN Aspartate amin
0,173112	0,3456	0,466118	1,160827	sp|P00558	sp|P00558-2|PGK1_HUMAN Isoform 2 of Ph
0,032481	0,076114	-2,43458	-2,48593	tr|B8ZX62	tr|B8ZX62|B8ZX62_HUMAN Plasminogen ac
0,692955	0,799985	-0,12335	-0,31021	tr|V9HWA	tr|V9HWA9|V9HWA9_HUMAN C3-beta-c OS
5,15E-06	0,011	-0,76263	-5,07655	tr|Q5H9A7	tr|Q5H9A7|Q5H9A7_HUMAN Metalloprotei
0,706059	0,833624	0,07721	0,261591	tr|A0A0K0	tr|A0A0K0K1J1|A0A0K0K1J1_HUMAN Epidid
0,169394	0,333919	0,483426	1,180354	tr|H0YMD	tr|H0YMD1|H0YMD1_HUMAN Low-density
0,574535	0,703502	0,228123	0,470957	tr|F6MZK5	tr|F6MZK5|F6MZK5_HUMAN Insulin (Fragm
0,485164	0,65275	0,296539	0,594568	sp|P01834	sp|P01834|IGKC_HUMAN Immunoglobulin k
0,063472	0,140857	1,920743	2,031874	tr|Q8NCL6	tr|Q8NCL6|Q8NCL6_HUMAN cDNA FLJ9017
0,063713	0,176	-0,81512	-1,77522	tr|A9LSU1	tr|A9LSU1|A9LSU1_HUMAN Type IV collage
0,416379	0,582922	0,342096	0,695322	sp|P02545	sp|P02545|LMNA_HUMAN Prelamin-A/C OS
0,002766	0,0901	-0,46134	-2,37147	tr|A0A0S2	tr|A0A0S2Z3D5|A0A0S2Z3D5_HUMAN Apol

0,289719	0,426184	0,64472	0,983874	sp|P02751	sp|P02751|FINC_HUMAN Fibronectin OS=H
0,482017	0,650057	-0,28064	-0,59139	tr|D6RBJ7	tr|D6RBJ7|D6RBJ7_HUMAN Gc-globulin OS=
0,163368	0,443725	0,228437	0,936814	sp|P02787	sp|P02787|TRFE_HUMAN Serotransferrin O
0,29374	0,453951	0,457274	0,919187	sp|P0279C	sp|P02790|HEMO_HUMAN Hemopexin OS=
0,005153	0,013143	1,389258	3,277427	tr|Q5TZP0	tr|Q5TZP0|Q5TZP0_HUMAN Matrix metallo
0,105826	0,319754	0,358313	1,242614	sp|P04075	sp|P04075-2|ALDOA_HUMAN Isoform 2 of I
0,072641	0,171576	1,300831	1,862835	tr|Q76LA1	tr|Q76LA1|Q76LA1_HUMAN CSTB protein C
0,616459	0,738041	0,198647	0,417118	sp|P04083	sp|P04083|ANXA1_HUMAN Annexin A1 OS=
0,63409	0,736067	-0,28398	-0,42601	tr|O75942	tr|O75942|O75942_HUMAN Major prion pr
0,304309	0,389943	1,74221	1,055096	tr|H6VRG3	tr|H6VRG3|H6VRG3_HUMAN Cytokeratin-1
0,742614	0,797154	-0,31402	-0,31	tr|E7EUT5	tr|E7EUT5|E7EUT5_HUMAN Glyceraldehyde
0,608366	0,700498	0,64179	0,498451	tr|A0A0U1	tr|A0A0U1RRH7|A0A0U1RRH7_HUMAN His
0,491584	0,715034	0,119644	0,454266	sp|P05067	sp|P05067-8|A4_HUMAN Isoform APP751 c
0,225035	0,344508	0,814988	1,159814	sp|P05109	sp|P05109|S10A8_HUMAN Protein S100-A8
0,469776	0,576761	-1,20561	-0,72497	tr|B2R7Y0	tr|B2R7Y0|B2R7Y0_HUMAN cDNA, FLJ9365
0,038657	0,097273	-1,82703	-2,30497	tr|A0A024	tr|A0A024QYT5|A0A024QYT5_HUMAN Serp
0,044433	0,1596	-0,80999	-1,93023	tr|A0A348	tr|A0A348GSH7|A0A348GSH7_HUMAN Serp
0,986142	0,994413	-0,00524	-0,01345	tr|A0A024	tr|A0A024RCA7|A0A024RCA7_HUMAN Ribo
0,429685	0,627862	-0,24755	-0,63085	sp|P05556	sp|P05556|ITB1_HUMAN Integrin beta-1 OS
0,956052	0,975959	0,016607	0,042684	sp|P06396	sp|P06396|GELS_HUMAN Gelsolin OS=Hom
0,599045	0,702931	0,329946	0,474988	tr|B2R4M6	tr|B2R4M6|B2R4M6_HUMAN Protein S100
0,126842	0,2925	0,645719	1,390192	tr|R4GN98	tr|R4GN98|R4GN98_HUMAN Protein S100 (
0,125746	0,282936	0,679073	1,40899	sp|P06733	sp|P06733|ENOA_HUMAN Alpha-enolase O
0,439854	0,615898	0,327084	0,660132	tr|A0A2U3	tr|A0A2U3TZU2|A0A2U3TZU2_HUMAN Glu
0,18672	0,383766	0,381137	1,0714	tr|A0A0S2	tr|A0A0S2Z4G7|A0A0S2Z4G7_HUMAN Nucl
0,411835	0,5064	1,847633	0,841594	sp|P06753	sp|P06753-3|TPM3_HUMAN Isoform 3 of Tr
0,318456	0,407234	-2,21274	-1,03679	tr|B4DMR	tr|B4DMR3|B4DMR3_HUMAN cDNA FLJ518
0,318153	0,513831	0,313011	0,80764	tr|A0A5F9	tr|A0A5F9ZHM4|A0A5F9ZHM4_HUMAN L-la
0,85949	0,92038	0,048272	0,133627	sp|P07237	sp|P07237|PDIA1_HUMAN Protein disulfide
0,89849	0,950083	-0,02473	-0,08651	sp|P07339	sp|P07339|CATD_HUMAN Cathepsin D OS=
0,03123	0,174448	0,549093	1,853687	sp|P07355	sp|P07355|ANXA2_HUMAN Annexin A2 OS=
0,480463	0,694726	-0,1592	-0,51078	tr|Q53FJ5	tr|Q53FJ5|Q53FJ5_HUMAN Prosaposin (Var
0,481455	0,581708	1,07314	0,701262	tr|Q5T8F0	tr|Q5T8F0|Q5T8F0_HUMAN Cathepsin L1 O
0,864072	0,921923	0,045274	0,128115	sp|P07737	sp|P07737|PROF1_HUMAN Profilin-1 OS=H
0,052141	0,156258	0,943094	1,923344	sp|P07858	sp|P07858|CATB_HUMAN Cathepsin B OS=H
0,589343	0,753474	-0,11486	-0,38097	sp|P07942	sp|P07942|LAMB1_HUMAN Laminin subuni
0,349124	0,614935	0,182549	0,652448	sp|P07996	sp|P07996|TSP1_HUMAN Thrombospondin
0,238923	0,411833	0,467437	1,021681	tr|A0A024	tr|A0A024RD80|A0A024RD80_HUMAN Hea
0,001075	0,014154	-0,77643	-3,34502	tr|Q53H39	tr|Q53H39|Q53H39_HUMAN Activin beta-A
0,003054	0,02887	-0,80415	-2,99913	tr|A0A024	tr|A0A024RDW8|A0A024RDW8_HUMAN Co
0,746212	0,83218	0,111631	0,25999	sp|P08581	sp|P08581-2|MET_HUMAN Isoform 2 of He
0,066441	0,151793	1,635481	1,969362	tr|Q53HU8	tr|Q53HU8|Q53HU8_HUMAN Vimentin (Fra
0,018902	0,105333	0,735286	2,223454	tr|V9HWE	tr|V9HWE0|V9HWE0_HUMAN Annexin OS=
0,218288	0,3339	-0,90572	-1,19371	tr|A8MX94	tr|A8MX94|A8MX94_HUMAN GST class-pi C
0,598442	0,706087	-0,3454	-0,47877	sp|P09341	sp|P09341|GROA_HUMAN Growth-regulate
0,698267	0,766518	-0,3146	-0,36022	tr|A0A384	tr|A0A384MR27|A0A384MR27_HUMAN Ga
0,706926	0,869358	0,047684	0,215858	sp|P09486	sp|P09486|SPRC_HUMAN SPARC OS=Homo
0,014785	0,089538	0,819217	2,394772	tr|F8WCZ6	tr|F8WCZ6|F8WCZ6_HUMAN Complement
0,392658	0,578894	-0,32041	-0,71527	tr|Q59EM9	tr|Q59EM9|Q59EM9_HUMAN Ubiquitin C v
0,427411	0,614558	-0,29183	-0,65888	sp|Q0110	sp|Q01105-3|SET_HUMAN Isoform 3 of Pro

0,158578	0,304302	0,709874	1,312189	tr|A0A0G2	tr|A0A0G2JIW1|A0A0G2JIW1_HUMAN Heat
0,085664	0,226744	0,631066	1,550113	tr|B4DJ51	tr|B4DJ51|B4DJ51_HUMAN Calmodulin OS=
0,034087	0,237747	-0,3462	-1,52705	sp|P10586	sp|P10586-2|PTPRF_HUMAN Isoform 2 of R
0,865341	0,907214	-0,13769	-0,15683	sp|P10599	sp|P10599|THIO_HUMAN Thioredoxin OS=H
0,021929	0,156984	-0,51961	-1,93001	sp|P11021	sp|P11021|BIP_HUMAN Endoplasmic reticu
0,545668	0,734727	-0,11278	-0,4084	tr|A0A024	tr|A0A024R972|A0A024R972_HUMAN Lami
0,806626	0,884823	0,070149	0,187464	sp|P11142	sp|P11142|HSP7C_HUMAN Heat shock cogr
0,03328	0,107911	-1,29135	-2,26737	tr|A0A087	tr|A0A087X0S5|A0A087X0S5_HUMAN Colla
0,429193	0,61499	0,289289	0,655505	tr|A0A024	tr|A0A024R694|A0A024R694_HUMAN Alph
0,956032	0,982742	0,005518	0,028152	tr|B3GN61	tr|B3GN61|B3GN61_HUMAN Truncated E-c
0,709359	0,756498	-0,81591	-0,37305	tr|A6XNE3	tr|A6XNE3|A6XNE3_HUMAN Interferon gam
0,265581	0,424338	0,483749	0,979109	tr|A0A384	tr|A0A384N6H1|A0A384N6H1_HUMAN Epi
0,075818	0,18642	-0,74895	-1,66633	tr|A0A090	tr|A0A090N8Y2|A0A090N8Y2_HUMAN Prot
0,215831	0,313286	-1,47465	-1,2647	tr|J3KPG2	tr|J3KPG2|J3KPG2_HUMAN Translationally-
0,009072	0,025895	1,824889	3,138353	tr|V9HWJ7	tr|V9HWJ7|V9HWJ7_HUMAN Epididymis se
0,56746	0,741107	0,128236	0,410976	sp|P13987	sp|P13987-2|CD59_HUMAN Isoform 2 of CD
0,147075	0,222024	-2,65336	-1,56622	tr|A0A0S2	tr|A0A0S2Z4A0|A0A0S2Z4A0_HUMAN Prote
0,035521	0,106415	-1,18047	-2,19817	sp|P14543	sp|P14543-2|NID1_HUMAN Isoform 2 of Nic
0,507419	0,611663	-0,78669	-0,64676	sp|P14618	sp|P14618|KPYM_HUMAN Pyruvate kinase
0,388619	0,573095	-0,30918	-0,7145	tr|Q5CAQ5	tr|Q5CAQ5|Q5CAQ5_HUMAN Epididymis se
0,513925	0,614503	1,642651	0,66542	tr|B3KSS4	tr|B3KSS4|B3KSS4_HUMAN cDNA FLJ36858
0,355954	0,505402	-0,47822	-0,827	tr|B4DMM	tr|B4DMM8|B4DMM8_HUMAN cDNA FLJ57
0,653664	0,729924	0,591667	0,437051	tr|B7Z5V2	tr|B7Z5V2|B7Z5V2_HUMAN cDNA FLJ54141
0,291675	0,42449	-0,62346	-0,9751	sp|P15514	sp|P15514|AREG_HUMAN Amphiregulin OS
0,00324	0,003667	-1,49603	-3,59101	tr|A0A024	tr|A0A024R8V7|A0A024R8V7_HUMAN Met
0,694538	0,774222	0,243108	0,352418	sp|P16070	sp|P16070|CD44_HUMAN CD44 antigen OS
0,131761	0,313658	0,470223	1,272221	sp|P16144	sp|P16144-5|ITB4_HUMAN Isoform Beta-4E
0,641214	0,754208	0,155025	0,372595	sp|P17900	sp|P17900|SAP3_HUMAN Ganglioside GM2
0,623698	0,703255	-0,63639	-0,4781	tr|A0A384	tr|A0A384NYN5|A0A384NYN5_HUMAN Me
0,532891	0,778961	-0,07122	-0,34294	tr|Q6FHU2	tr|Q6FHU2|Q6FHU2_HUMAN Phosphoglyce
0,029706	0,168188	-0,56531	-1,88875	sp|P19883	sp|P19883-2|FST_HUMAN Isoform 2 of Folli
0,000966	0,0088	2,989621	4,99568	sp|P19957	sp|P19957|ELAF_HUMAN Elafin OS=Homo s
0,550901	0,668822	0,529972	0,564392	sp|P20827	sp|P20827|EFNA1_HUMAN Ephrin-A1 OS=H
0,172346	0,344194	0,474299	1,167676	tr|Q60FE6	tr|Q60FE6|Q60FE6_HUMAN Filamin A OS=H
0,000671	0,0044	-1,13602	-4,10434	tr|E9PPJ5|	tr|E9PPJ5|E9PPJ5_HUMAN Midkine (Fragme
0,037637	0,076865	-3,237	-2,45609	sp|P21810	sp|P21810|PGS1_HUMAN Biglycan OS=Hom
0,961708	0,976377	-0,01515	-0,03762	tr|B4DPP0	tr|B4DPP0|B4DPP0_HUMAN cDNA FLJ5103
0,987957	0,992252	-0,00683	-0,01279	sp|P22223	sp|P22223-2|CADH3_HUMAN Isoform 2 of
0,856995	0,90706	0,085667	0,154238	sp|P22392	sp|P22392-2|NDKB_HUMAN Isoform 3 of N
0,337068	0,42344	1,990769	0,991223	sp|P22528	sp|P22528|SPR1B_HUMAN Cornifin-B OS=H
0,055077	0,10552	-3,65809	-2,23098	sp|P22692	sp|P22692|IBP4_HUMAN Insulin-like growth
0,000248	0,0055	-1,15084	-4,62101	tr|B4DUV1	tr|B4DUV1|B4DUV1_HUMAN Fibulin-1 OS=
0,369593	0,575243	-0,28418	-0,72306	sp|P23229	sp|P23229-4|ITA6_HUMAN Isoform Alpha-6
0,636814	0,738453	-0,23455	-0,41016	tr|V9HWC	tr|V9HWC6|V9HWC6_HUMAN Peptidyl-pro
0,280111	0,453129	0,399214	0,914916	tr|E9PK25	tr|E9PK25|E9PK25_HUMAN Cofilin, non-mu
0,312834	0,419537	-1,16317	-1,00634	sp|P24821	sp|P24821|TENA_HUMAN Tenascin OS=Hor
0,542097	0,676649	-0,29718	-0,53075	sp|P25391	sp|P25391|LAMA1_HUMAN Laminin subuni
0,119789	0,191949	2,250046	1,677741	sp|P25788	sp|P25788-2|PSA3_HUMAN Isoform 2 of Pr
0,266826	0,410797	-0,63167	-1,02533	sp|P25789	sp|P25789-2|PSA4_HUMAN Isoform 2 of Pr
0,020636	0,043143	3,240671	2,844386	tr|Q59F03	tr|Q59F03|Q59F03_HUMAN Integrin alpha

0,629394	0,736033	-0,21195	-0,40997	tr|V9HWC	tr|V9HWC0|V9HWC0_HUMAN Epididymis lu
0,9456	0,968272	0,025165	0,05546	tr|Q53G71	tr|Q53G71|Q53G71_HUMAN Calreticulin va
0,187596	0,301037	1,082535	1,30734	sp|P27930	sp|P27930|IL1R2_HUMAN Interleukin-1 rec
0,002483	0,004	1,714172	3,863522	tr|Q5U0A(	tr|Q5U0A0|Q5U0A0_HUMAN Proteasome s
0,5545	0,753694	0,095943	0,378748	sp|P28799	sp|P28799|GRN_HUMAN Progranulin OS=H
0,213708	0,376441	0,493003	1,084706	sp|P29034	sp|P29034|S10A2_HUMAN Protein S100-A2
0,404654	0,508139	-1,08154	-0,82764	sp|P29317	sp|P29317-2|EPHA2_HUMAN Isoform 2 of E
0,003263	0,037333	0,785824	2,948189	tr|V9HWD	tr|V9HWD9|V9HWD9_HUMAN Epididymis l
0,811699	0,875362	0,097795	0,198438	tr|D9IAI1|	tr|D9IAI1|D9IAI1_HUMAN Epididymis secret
0,18155	0,391453	-0,35205	-1,05721	tr|B3KQT9	tr|B3KQT9|B3KQT9_HUMAN Protein disulfid
0,093288	0,169314	-2,23153	-1,82985	sp|P30530	sp|P30530|UFO_HUMAN Tyrosine-protein k
0,226332	0,349576	-0,68009	-1,12499	sp|P31431	sp|P31431-2|SDC4_HUMAN Isoform 2 of Sy
0,535873	0,675908	0,333885	0,548604	tr|B4DP51	tr|B4DP51|B4DP51_HUMAN cDNA FLJ5453
0,928952	0,960096	-0,03757	-0,07453	sp|P31946	sp|P31946-2|1433B_HUMAN Isoform Short
0,641987	0,799938	0,086371	0,31236	sp|P31947	sp|P31947|1433S_HUMAN 14-3-3 protein s
0,060371	0,33362	-0,24466	-1,18784	tr|V9HWH	tr|V9HWH9|V9HWH9_HUMAN Protein S10(
0,023377	0,09181	-1,02557	-2,3334	sp|P32004	sp|P32004-3|L1CAM_HUMAN Isoform 3 of I
0,071149	0,321913	-0,28311	-1,23457	tr|A0A384	tr|A0A384NPH9|A0A384NPH9_HUMAN Gly
0,210849	0,291485	4,318131	1,356669	sp|P35408	sp|P35408|PE2R4_HUMAN Prostaglandin E
6,31E-05	0	3,183593	7,523012	sp|P35442	sp|P35442|TSP2_HUMAN Thrombospondin
0,189466	0,302691	1,022416	1,29256	sp|P35556	sp|P35556|FBN2_HUMAN Fibrillin-2 OS=Ho
0,685929	0,791172	0,141559	0,326632	tr|A0A024	tr|A0A024R1N1|A0A024R1N1_HUMAN Myo
0,004635	0,025867	1,220905	3,227093	tr|A0A024	tr|A0A024R2B6|A0A024R2B6_HUMAN Serp
0,713684	0,79528	0,158342	0,309543	tr|X6RJP6|	tr|X6RJP6|X6RJP6_HUMAN Transgelin-2 (Fra
4,62E-05	0	-1,55537	-6,23382	tr|D3DSM	tr|D3DSM4|D3DSM4_HUMAN Collagen, typ
0,00831	0,050897	0,969624	2,763653	tr|V9HWF	tr|V9HWF2|V9HWF2_HUMAN Malate dehy
0,559173	0,677104	-0,38397	-0,53246	tr|Q6P1N4	tr|Q6P1N4|Q6P1N4_HUMAN IQGAP1 prote
0,997131	0,996318	-0,00213	-0,00319	tr|B1AK87	tr|B1AK87|B1AK87_HUMAN F-actin-capping
0,026208	0,049125	-3,87301	-2,72592	tr|Q8NE89	tr|Q8NE89|Q8NE89_HUMAN Similar to tissu
0,83707	0,890331	-0,11298	-0,18046	sp|P49327	sp|P49327|FAS_HUMAN Fatty acid synthase
0,497292	0,608948	0,969762	0,672219	tr|Q6FH59	tr|Q6FH59|Q6FH59_HUMAN VEGFC protein
0,686737	0,835727	0,071025	0,26528	tr|Q5SX91	tr|Q5SX91|Q5SX91_HUMAN Rab GDP disso
0,723542	0,848506	-0,06729	-0,23906	sp|P50895	sp|P50895|BCAM_HUMAN Basal cell adhesi
0,192926	0,328305	0,699677	1,212252	tr|J3KTF8|	tr|J3KTF8|J3KTF8_HUMAN Rho GDP-dissoci
0,001696	0,021556	-0,76532	-3,15908	sp|P52799	sp|P52799|EFNB2_HUMAN Ephrin-B2 OS=H
1	1	0	0	sp|P53634	sp|P53634|CATC_HUMAN Dipeptidyl peptid
0,190027	0,284344	-3,25642	-1,41332	sp|P55001	sp|P55001-3|MFAP2_HUMAN Isoform B of
0,775644	0,830045	-0,1989	-0,25928	sp|P55058	sp|P55058|PLTP_HUMAN Phospholipid tran
0,031105	0,112582	0,915733	2,145181	tr|Q96IF9|	tr|Q96IF9|Q96IF9_HUMAN VCP protein (Fra
0,868919	0,908865	-0,12118	-0,1508	sp|P55145	sp|P55145|MANF_HUMAN Mesencephalic a
0,181197	0,358687	0,41957	1,111668	sp|P55268	sp|P55268|LAMB2_HUMAN Laminin subuni
0,093507	0,169391	-2,37711	-1,83765	sp|P55290	sp|P55290-5|CAD13_HUMAN Isoform 5 of (
0,083225	0,358256	-0,24152	-1,11632	tr|E9PJK1|	tr|E9PJK1|E9PJK1_HUMAN Tetraspanin OS=
0,272063	0,476843	0,328954	0,88419	tr|B4DUI5	tr|B4DUI5|B4DUI5_HUMAN Triosephosphat
0,431865	0,575831	0,473395	0,715128	tr|G3V3I1|	tr|G3V3I1|G3V3I1_HUMAN Proteasome sub
0,180506	0,486994	-0,20471	-0,87072	sp|P61769	sp|P61769|B2MG_HUMAN Beta-2-microglo
0,024804	0,165778	-0,47175	-1,82436	tr|Q53HV6	tr|Q53HV6|Q53HV6_HUMAN Epididymal se
0,022488	0,106696	0,880342	2,265666	sp|P61981	sp|P61981|1433G_HUMAN 14-3-3 protein g
0,016415	0,106	0,650204	2,188853	sp|P62258	sp|P62258|1433E_HUMAN 14-3-3 protein e
0,58762	0,707966	0,24509	0,464257	tr|A2VCK8	tr|A2VCK8|A2VCK8_HUMAN Thymosin beta

0,560481	0,680349	-0,44763	-0,54154	tr|Q0VAS5	tr|Q0VAS5|Q0VAS5_HUMAN Histone H4 OS
0,567725	0,67905	0,496645	0,538812	sp|P62937	sp|P62937|PPIA_HUMAN Peptidyl-prolyl cis
0,165006	0,327829	0,532677	1,219037	sp|P62942	sp|P62942|FKB1A_HUMAN Peptidyl-prolyl c
0,615059	0,800856	0,077648	0,31504	tr|D0PNI1	tr|D0PNI1|D0PNI1_HUMAN Epididymis lum
0,679991	0,784988	-0,14749	-0,33486	tr|B4DVQ(	tr|B4DVQ0|B4DVQ0_HUMAN cDNA FLJ5828
0,457242	0,561011	-1,27186	-0,74767	sp|P63313	sp|P63313|TYB10_HUMAN Thymosin beta-1
0,000317	0,004889	-1,07323	-4,3736	sp|P78324	sp|P78324-2|SHPS1_HUMAN Isoform 2 of T
0,015704	0,077333	-0,95299	-2,4706	sp|P78504	sp|P78504|JAG1_HUMAN Protein jagged-1
0,000585	0,006286	1,625356	4,690488	sp|P80188	sp|P80188|NGAL_HUMAN Neutrophil gelati
0,02168	0,044741	-3,86098	-2,85194	tr|E9PKG6	tr|E9PKG6|E9PKG6_HUMAN Nucleobindin-2
0,806291	0,868118	-0,13383	-0,2151	tr|Q86V58	tr|Q86V58|Q86V58_HUMAN Fibulin 2 OS=H
0,130072	0,45561	-0,18974	-0,91129	sp|P98172	sp|P98172|EFNB1_HUMAN Ephrin-B1 OS=H
0,16215	0,288941	0,904614	1,354864	tr|Q5T0R7	tr|Q5T0R7|Q5T0R7_HUMAN Adenylyl cycla
0,56823	0,697737	-0,26546	-0,49176	tr|Q7RTW	tr|Q7RTW3|Q7RTW3_HUMAN Neuregulin 1
0,399056	0,581419	-0,34333	-0,71786	sp|Q0238	sp|Q02388|CO7A1_HUMAN Collagen alpha-
0,321094	0,425086	-1,12296	-0,98633	tr|B4DGN	tr|B4DGN8|B4DGN8_HUMAN Procollagen-l
0,136929	0,285263	-0,77067	-1,40367	sp|Q0281	sp|Q02818|NUCB1_HUMAN Nucleobindin-1
0,242557	0,468194	0,289097	0,894899	tr|A0A024	tr|A0A024R3N3|A0A024R3N3_HUMAN Am
0,120882	0,424366	0,21564	0,982962	tr|A0A384	tr|A0A384NPQ2|A0A384NPQ2_HUMAN Epi
0,229604	0,417131	0,420208	1,014423	tr|A6NCT7	tr|A6NCT7|A6NCT7_HUMAN Collagen alpha
0,564846	0,732114	-0,12383	-0,40824	sp|Q0834	sp|Q08345-5|DDR1_HUMAN Isoform 4 of E
0,119272	0,343877	-0,30859	-1,14325	tr|A0A0S2	tr|A0A0S2Z3Y1|A0A0S2Z3Y1_HUMAN Gale
0,210309	0,304147	-1,68915	-1,29504	tr|G3V1S6	tr|G3V1S6|G3V1S6_HUMAN Polypeptide N-
0,006421	0,03792	1,028663	2,926995	tr|Q59G97	tr|Q59G97|Q59G97_HUMAN EGF-containin
0,051308	0,250711	-0,3861	-1,49058	sp|Q1284	sp|Q12841|FSTL1_HUMAN Follistatin-relate
0,405524	0,504273	0,974311	0,819251	sp|Q1286	sp|Q12860-2|CNTN1_HUMAN Isoform 2 of
0,021902	0,087902	1,028987	2,365388	tr|C9J4H5	tr|C9J4H5|C9J4H5_HUMAN Semaphorin-3F
0,725712	0,82984	0,10561	0,272746	tr|Q59FV9	tr|Q59FV9|Q59FV9_HUMAN PTK7 protein t
0,084112	0,159525	-3,0365	-1,9362	tr|H3BR90	tr|H3BR90|H3BR90_HUMAN Mesothelin (Fr
0,640813	0,701573	-1,07826	-0,46969	tr|F8WC5	tr|F8WC54|F8WC54_HUMAN Disintegrin an
0,222151	0,420779	0,371758	0,996735	tr|A0A0S2	tr|A0A0S2Z3R6|A0A0S2Z3R6_HUMAN Lami
0,003797	0,027818	-0,88861	-3,01943	sp|Q1375	sp|Q13753|LAMC2_HUMAN Laminin subun
0,151191	0,282348	-1,03701	-1,41935	tr|A0A024	tr|A0A024R2W4|A0A024R2W4_HUMAN Al
0,850123	0,918261	0,047975	0,139794	tr|B4DZY7	tr|B4DZY7|B4DZY7_HUMAN cDNA FLJ57022
0,628432	0,778403	-0,10761	-0,34589	sp|Q1451	sp|Q14512|FGFP1_HUMAN Fibroblast grow
0,10582	0,288427	-0,46114	-1,34678	tr|G3V511	tr|G3V511|G3V511_HUMAN Latent-transfo
0,275225	0,42795	0,474051	0,957847	sp|Q1508	sp|Q15084-3|PDIA6_HUMAN Isoform 3 of P
0,921523	0,948194	-0,09365	-0,09258	sp|Q1514	sp|Q15149-7|PLEC_HUMAN Isoform 7 of Ple
0,899263	0,932322	-0,08241	-0,11381	tr|Q6SYC2	tr|Q6SYC2|Q6SYC2_HUMAN Poliovirus rece
0,972354	0,984282	-0,01737	-0,02991	tr|Q59EZ1	tr|Q59EZ1|Q59EZ1_HUMAN Protein tyrosin
0,349164	0,502296	0,518468	0,849001	tr|A0A0S2	tr|A0A0S2Z4Q2|A0A0S2Z4Q2_HUMAN Tran
0,823204	0,907183	0,051346	0,160464	sp|Q1627	sp|Q16270|IBP7_HUMAN Insulin-like growt
0,810637	0,875171	-0,10167	-0,20091	tr|A0A140	tr|A0A140VJI7|A0A140VJI7_HUMAN Testicu
0,019721	0,097395	0,866619	2,312279	tr|B3KTA3	tr|B3KTA3|B3KTA3_HUMAN Fascin OS=Hor
0,012283	0,103308	-0,59771	-2,22156	sp|Q1676	sp|Q16769|QPCT_HUMAN Glutaminyl-pept
0,055094	0,189519	0,570727	1,677424	tr|A0A0A6	tr|A0A0A6YYF2|A0A0A6YYF2_HUMAN HCG
0,050138	0,174215	0,783273	1,863287	sp|Q24JP5	sp|Q24JP5|T132A_HUMAN Transmembrane
1	1	0	0	tr|B4DTF2	tr|B4DTF2|B4DTF2_HUMAN Annexin OS=Ho
0,257563	0,343496	-1,52522	-1,15602	sp|Q6H3X	sp|Q6H3X3|ULBP5_HUMAN UL-16 binding p
0,593792	0,677964	-1,12261	-0,53617	tr|A0A024	tr|A0A024R091|A0A024R091_HUMAN von

<table>
<tr><td>0,006606</td><td>0,026286</td><td>-1,17995</td><td>-3,02393</td><td>sp|Q6UVK</td><td>sp|Q6UVK1|CSPG4_HUMAN Chondroitin su</td></tr>
<tr><td>0,163959</td><td>0,295636</td><td>0,946445</td><td>1,357964</td><td>sp|Q6YHK</td><td>sp|Q6YHK3-4|CD109_HUMAN Isoform 4 of</td></tr>
<tr><td>0,624134</td><td>0,730269</td><td>-0,27342</td><td>-0,43427</td><td>tr|B3KRK1</td><td>tr|B3KRK1|B3KRK1_HUMAN cDNA FLJ34442</td></tr>
<tr><td>0,188183</td><td>0,322584</td><td>-0,76369</td><td>-1,24298</td><td>sp|Q86X2</td><td>sp|Q86X29-6|LSR_HUMAN Isoform 6 of Lip</td></tr>
<tr><td>0,303199</td><td>0,453342</td><td>-0,51571</td><td>-0,92417</td><td>sp|Q8IUX7</td><td>sp|Q8IUX7|AEBP1_HUMAN Adipocyte enha</td></tr>
<tr><td>0,282926</td><td>0,356859</td><td>-1,89489</td><td>-1,11018</td><td>sp|Q8IWU</td><td>sp|Q8IWU5-2|SULF2_HUMAN Isoform 2 of</td></tr>
<tr><td>0,542278</td><td>0,659509</td><td>-0,53666</td><td>-0,57647</td><td>sp|Q8NBP</td><td>sp|Q8NBP7|PCSK9_HUMAN Proprotein con</td></tr>
<tr><td>0,406554</td><td>0,626392</td><td>-0,21399</td><td>-0,62975</td><td>tr|B7Z5C1</td><td>tr|B7Z5C1|B7Z5C1_HUMAN cDNA FLJ56126</td></tr>
<tr><td>0,184139</td><td>0,297654</td><td>1,160653</td><td>1,328773</td><td>sp|Q9252</td><td>sp|Q92520|FAM3C_HUMAN Protein FAM3</td></tr>
<tr><td>0,934612</td><td>0,959329</td><td>-0,04791</td><td>-0,07258</td><td>sp|Q9262</td><td>sp|Q92626|PXDN_HUMAN Peroxidasin hom</td></tr>
<tr><td>0,003061</td><td>0,040769</td><td>-0,72736</td><td>-2,8846</td><td>sp|Q9274</td><td>sp|Q92743|HTRA1_HUMAN Serine protease</td></tr>
<tr><td>0,009528</td><td>0,02425</td><td>-2,21346</td><td>-3,20553</td><td>sp|Q9282</td><td>sp|Q92823-5|NRCAM_HUMAN Isoform 5 of</td></tr>
<tr><td>0,41829</td><td>0,625971</td><td>-0,22553</td><td>-0,62725</td><td>sp|Q9289</td><td>sp|Q92896|GSLG1_HUMAN Golgi apparatus</td></tr>
<tr><td>0,124142</td><td>0,19841</td><td>1,812882</td><td>1,626779</td><td>tr|C9JMK5</td><td>tr|C9JMK5|C9JMK5_HUMAN Phosphoinosit</td></tr>
<tr><td>0,474604</td><td>0,58139</td><td>1,171906</td><td>0,716009</td><td>tr|V9HWC</td><td>tr|V9HWC2|V9HWC2_HUMAN Maillard deg</td></tr>
<tr><td>0,9403</td><td>0,965734</td><td>-0,03245</td><td>-0,06294</td><td>tr|B7Z4S8</td><td>tr|B7Z4S8|B7Z4S8_HUMAN Asparaginyl end</td></tr>
<tr><td>0,521299</td><td>0,655962</td><td>0,402622</td><td>0,582446</td><td>sp|Q9971</td><td>sp|Q99715-4|COCA1_HUMAN Isoform 4 of</td></tr>
<tr><td>0,006068</td><td>0,087368</td><td>-0,57563</td><td>-2,40861</td><td>sp|Q9998</td><td>sp|Q99985|SEM3C_HUMAN Semaphorin-3</td></tr>
<tr><td>0,082093</td><td>0,184587</td><td>0,978343</td><td>1,719142</td><td>tr|A0A140</td><td>tr|A0A140VJY7|A0A140VJY7_HUMAN Thior</td></tr>
<tr><td>0,098662</td><td>0,262549</td><td>-0,56593</td><td>-1,45188</td><td>sp|Q9BRK</td><td>sp|Q9BRK5-4|CAB45_HUMAN Isoform 4 of</td></tr>
<tr><td>0,695855</td><td>0,852741</td><td>0,053686</td><td>0,232553</td><td>sp|Q9GZ</td><td>sp|Q9GZM7-3|TINAL_HUMAN Isoform 3 of</td></tr>
<tr><td>0,341855</td><td>0,422369</td><td>1,593416</td><td>0,969245</td><td>sp|Q9H29</td><td>sp|Q9H299|SH3L3_HUMAN SH3 domain-bir</td></tr>
<tr><td>0,809089</td><td>0,869436</td><td>0,131424</td><td>0,2118</td><td>sp|Q9NPR</td><td>sp|Q9NPR2-2|SEM4B_HUMAN Isoform 2 of</td></tr>
<tr><td>0,783335</td><td>0,833132</td><td>0,330984</td><td>0,264599</td><td>tr|F8VNQ</td><td>tr|F8VNQ3|F8VNQ3_HUMAN Teneurin-2 O</td></tr>
<tr><td>0,02329</td><td>0,1065</td><td>0,879547</td><td>2,250078</td><td>tr|Q4ZG85</td><td>tr|Q4ZG85|Q4ZG85_HUMAN Uncharacteriz</td></tr>
<tr><td>0,591832</td><td>0,733</td><td>-0,17197</td><td>-0,42592</td><td>sp|Q9P2B</td><td>sp|Q9P2B2|FPRP_HUMAN Prostaglandin F2</td></tr>
<tr><td>0,155429</td><td>0,42162</td><td>-0,23834</td><td>-0,96599</td><td>sp|Q9UBP</td><td>sp|Q9UBP4|DKK3_HUMAN Dickkopf-relate</td></tr>
<tr><td>0,155691</td><td>0,29388</td><td>-0,82888</td><td>-1,35733</td><td>tr|Q5U00</td><td>tr|Q5U000|Q5U000_HUMAN Cathepsin X O</td></tr>
<tr><td>0,018537</td><td>0,049548</td><td>1,876957</td><td>2,735875</td><td>tr|B7Z5J4|</td><td>tr|B7Z5J4|B7Z5J4_HUMAN Carboxypeptida</td></tr>
<tr><td>0,199365</td><td>0,300082</td><td>2,442866</td><td>1,361716</td><td>sp|Q9UM</td><td>sp|Q9UMD9-2|COHA1_HUMAN Isoform 2 o</td></tr>
<tr><td>0,342993</td><td>0,421769</td><td>1,844257</td><td>0,974972</td><td>sp|Q9UN</td><td>sp|Q9UNN8|EPCR_HUMAN Endothelial prot</td></tr>
<tr><td>0,049114</td><td>0,139158</td><td>-1,14654</td><td>-2,02548</td><td>tr|A0A2I2</td><td>tr|A0A2I2MP48|A0A2I2MP48_HUMAN Kalli</td></tr>
<tr><td>0,44071</td><td>0,554144</td><td>-0,91809</td><td>-0,75733</td><td>tr|W6I206</td><td>tr|W6I206|W6I206_HUMAN Lysyl oxidase h</td></tr>
<tr><td>0,467897</td><td>0,565727</td><td>1,662973</td><td>0,740285</td><td>tr|B2RAF9</td><td>tr|B2RAF9|B2RAF9_HUMAN Suppressor of t</td></tr>
<tr><td>0,146478</td><td>0,319966</td><td>0,474093</td><td>1,233564</td><td>tr|D3DVF0</td><td>tr|D3DVF0|D3DVF0_HUMAN Junctional adh</td></tr>
<tr><td>0,355535</td><td>0,504709</td><td>0,460387</td><td>0,822131</td><td>tr|A0A024</td><td>tr|A0A024R462|A0A024R462_HUMAN Fibr</td></tr>
<tr><td>0,071141</td><td>0,188727</td><td>0,746922</td><td>1,693064</td><td>tr|W8GRM</td><td>tr|W8GRM7|W8GRM7_HUMAN Kallikrein 1</td></tr>
<tr><td>0,757685</td><td>0,811084</td><td>0,30479</td><td>0,292017</td><td>tr|A0A024</td><td>tr|A0A024R884|A0A024R884_HUMAN Ten</td></tr>
<tr><td>0,137473</td><td>0,508047</td><td>-0,16297</td><td>-0,83525</td><td>tr|A0A024</td><td>tr|A0A024RAB6|A0A024RAB6_HUMAN Hep</td></tr>
<tr><td>0,62689</td><td>0,70246</td><td>-1,38106</td><td>-0,49377</td><td>tr|D7RVA3</td><td>tr|D7RVA3|D7RVA3_HUMAN Granulocyte c</td></tr>
<tr><td>0,129345</td><td>0,18715</td><td>-3,46463</td><td>-1,67264</td><td>tr|A0A1V1</td><td>tr|A0A1V1IFQ2|A0A1V1IFQ2_HUMAN MHC</td></tr>
<tr><td>0,478832</td><td>0,578325</td><td>-1,12927</td><td>-0,70768</td><td colspan="2">tr|A0A1D6ZNI8|A0A1D6ZNI8_HUMAN;tr|A0A0S4XRM</td></tr>
<tr><td></td><td></td><td></td><td></td><td></td><td></td></tr>
<tr><td>0,028866</td><td>0,056706</td><td>-3,79818</td><td>-2,65825</td><td>tr|A0A288</td><td>tr|A0A288Q9P9|A0A288Q9P9_HUMAN MH</td></tr>
<tr><td>0,371543</td><td>0,507209</td><td>0,607851</td><td>0,833224</td><td>tr|A0A384</td><td>tr|A0A384MR03|A0A384MR03_HUMAN Fib</td></tr>
<tr><td>0,368236</td><td>0,529955</td><td>0,416966</td><td>0,788755</td><td>tr|F5H2D0</td><td>tr|F5H2D0|F5H2D0_HUMAN Complement s</td></tr>
<tr><td>0,005931</td><td>0,049212</td><td>0,785157</td><td>2,72015</td><td>tr|A8K6T3</td><td>tr|A8K6T3|A8K6T3_HUMAN cDNA FLJ78674</td></tr>
<tr><td>0,807639</td><td>0,874</td><td>0,106332</td><td>0,205322</td><td>tr|D3DS13</td><td>tr|D3DS13|D3DS13_HUMAN Ribonuclease</td></tr>
<tr><td>0,470325</td><td>0,70075</td><td>0,143357</td><td>0,501087</td><td>tr|B3KNN</td><td>tr|B3KNN9|B3KNN9_HUMAN Activated leuk</td></tr>
<tr><td>0,455734</td><td>0,673042</td><td>0,185544</td><td>0,5576</td><td>tr|B4E1B2</td><td>tr|B4E1B2|B4E1B2_HUMAN Beta-1 metal-b</td></tr>
</table>

0,016053	0,104327	-0,69654	-2,24722	tr|B7Z507	tr|B7Z507|B7Z507_HUMAN cDNA FLJ51036
0,523705	0,624544	0,838384	0,626315	tr|C9JB55|	tr|C9JB55|C9JB55_HUMAN Serotransferrin (
0,323888	0,413239	-2,26486	-1,02586	tr|C9JVG0	tr|C9JVG0|C9JVG0_HUMAN Serotransferrin
0,24122	0,419479	0,452244	1,009903	tr|E7ET40	tr|E7ET40|E7ET40_HUMAN Urokinase-type
0,148041	0,215762	-3,70099	-1,58848	tr|V5NQE(	tr|V5NQE0|V5NQE0_HUMAN MHC class I ar
0,922746	0,947306	0,212711	0,096535	tr|H0YBI8|	tr|H0YBI8|H0YBI8_HUMAN Vascular endoth
0,000321	0,007333	-1,43398	-4,86586	tr|Q5M8T4	tr|Q5M8T4|Q5M8T4_HUMAN Connective ti

:ytoskeletal 14 OS=Homo sapiens OX=9606 GN=KRT14 PE=1 SV=4;tr|A0A024R1X6|A0A024R1X6_HU
i7, highly similar to Keratin, type II cytoskeletal 6A OS=Homo sapiens OX=9606 PE=2 SV=1;sp|P02538

ytoskeletal 5 OS=Homo sapiens OX=9606 GN=KRT5 PE=1 SV=3;tr|B4E1T1|B4E1T1_HUMAN cDNA FL
'toskeletal 9 OS=Homo sapiens OX=9606 GN=KRT9 PE=1 SV=3;tr|K7EQQ3|K7EQQ3_HUMAN Keratin,
:ytoskeletal 17 OS=Homo sapiens OX=9606 GN=KRT17 PE=1 SV=2;;tr|F5GWP8|F5GWP8_HUMAN Ke
protein (Fragment) OS=Homo sapiens OX=9606 GN=C1 segment PE=4 SV=1;tr|Q5CZ94|Q5CZ94_HU
oride intracellular channel protein OS=Homo sapiens OX=9606 PE=3 SV=1;tr|Q5SRT3|Q5SRT3_HUM
ase 1 OS=Homo sapiens OX=9606 GN=QSOX1 PE=1 SV=3;tr|A0A140VKE5|A0A140VKE5_HUMAN Sul
dase OS=Homo sapiens OX=9606 GN=MANBA PE=1 SV=1;tr|B4DT18|B4DT18_HUMAN Beta-mannos
Agrin OS=Homo sapiens OX=9606 GN=AGRN;sp|O00468-7|AGRIN_HUMAN Isoform 7 of Agrin OS=Ho
Syntenin-1 OS=Homo sapiens OX=9606 GN=SDCBP;tr|G5EA09|G5EA09_HUMAN Syndecan binding p
)S=Homo sapiens OX=9606 GN=CYR61 PE=1 SV=1;sp|O00622|CCN1_HUMAN CCN family member 1
d metalloproteinase domain-containing protein 10 OS=Homo sapiens OX=9606 GN=ADAM10 PE=2 S
tory light chain MRCL3 variant (Fragment) OS=Homo sapiens OX=9606 PE=2 SV=1;sp|P19105|ML12

ease inhibitor 1 OS=Homo sapiens OX=9606 GN=SPINT1 PE=1 SV=2;tr|H3BVD9|H3BVD9_HUMAN Ku
nin alpha 4 isoform 1 (Fragment) OS=Homo sapiens OX=9606 GN=ACTN4 PE=2 SV=1;sp|O43707|ACT
Protocadherin-7 OS=Homo sapiens OX=9606 GN=PCDH7;sp|O60245|PCDH7_HUMAN Protocadherin
pe 1-K OS=Homo sapiens OX=9606 GN=H2BC12 PE=1 SV=3;tr|A0A024RCJ9|A0A024RCJ9_HUMAN Hi
5, highly similar to Homo sapiens cathepsin L2 (CTSL2), mRNA OS=Homo sapiens OX=9606 PE=2 SV=1
3, highly similar to WD repeat protein 1 OS=Homo sapiens OX=9606 PE=2 SV=1;sp|O75083-3|WDR1_
\ OS=Homo sapiens OX=9606 GN=SEMA7A PE=1 SV=1;sp|O75326-2|SEM7A_HUMAN Isoform 2 of S
lamin-B OS=Homo sapiens OX=9606 GN=FLNB;sp|O75369-9|FLNB_HUMAN Isoform 9 of Filamin-B O
rpin B7 OS=Homo sapiens OX=9606 GN=SERPINB7;sp|O75635|SPB7_HUMAN Serpin B7 OS=Homo s
L, highly similar to Attractin OS=Homo sapiens OX=9606 PE=2 SV=1;sp|O75882-3|ATRN_HUMAN Iso
4 fis, clone MAMMA1002485, highly similar to Stanniocalcin-2 OS=Homo sapiens OX=9606 PE=2 SV=1
rotein 1 OS=Homo sapiens OX=9606 GN=DKK1 PE=2 SV=1;sp|O94907|DKK1_HUMAN Dickkopf-relat

48 fis, clone NT2RP2000028, highly similar to Serine protease 23 OS=Homo sapiens OX=9606 PE=2 S
protein DKFZp686D0114 OS=Homo sapiens OX=9606 GN=DKFZp686D0114 PE=4 SV=1;tr|B2RBR3|B
ollistatin-related protein 3 OS=Homo sapiens OX=9606 GN=FSTL3;tr|A0A024R1Y8|A0A024R1Y8_HU
iydrogenase OS=Homo sapiens OX=9606 GN=HEL-S-133P PE=2 SV=1;sp|P00338|LDHA_HUMAN L-la
notransferase OS=Homo sapiens OX=9606 PE=2 SV=1;sp|P00505-2|AATM_HUMAN Isoform 2 of Asp
iosphoglycerate kinase 1 OS=Homo sapiens OX=9606 GN=PGK1;tr|V9HWF4|V9HWF4_HUMAN Phos
:tivator OS=Homo sapiens OX=9606 GN=PLAT PE=2 SV=1;sp|P00750|TPA_HUMAN Tissue-type plasm
S=Homo sapiens OX=9606 GN=HEL-S-62p PE=2 SV=1;sp|P01024|CO3_HUMAN Complement C3 OS=
inase inhibitor 1 OS=Homo sapiens OX=9606 GN=TIMP1 PE=1 SV=1;tr|Q6FGX5|Q6FGX5_HUMAN Me
dymis secretory protein Li 2 OS=Homo sapiens OX=9606 GN=HEL-S-2 PE=2 SV=1;sp|P01034|CYTC_H
lipoprotein receptor OS=Homo sapiens OX=9606 GN=LDLR PE=1 SV=1;tr|Q9UH51|Q9UH51_HUMAN
ent) OS=Homo sapiens OX=9606 GN=INS PE=2 SV=1;tr|A6XGL2|A6XGL2_HUMAN Insulin OS=Homo
kappa constant OS=Homo sapiens OX=9606 GN=IGKC PE=1 SV=2;tr|A0A5H1ZRQ3|A0A5H1ZRQ3_HU
0 fis, clone MAMMA1000370, highly similar to Ig alpha-1 chain C region OS=Homo sapiens OX=9606
n alpha 1 (Fragment) OS=Homo sapiens OX=9606 PE=2 SV=1;tr|A0A3B3ISV3|A0A3B3ISV3_HUMAN
S=Homo sapiens OX=9606 GN=LMNA PE=1 SV=1;tr|A0A384MQX1|A0A384MQX1_HUMAN Epididymi
ipoprotein E isoform 1 (Fragment) OS=Homo sapiens OX=9606 GN=APOE PE=2 SV=1;sp|P02649|APO

omo sapiens OX=9606 GN=FN1 PE=1 SV=5;sp|P02751-7|FINC_HUMAN Isoform 7 of Fibronectin OS=
=Homo sapiens OX=9606 GN=GC PE=1 SV=1;sp|P02774-2|VTDB_HUMAN Isoform 2 of Vitamin D-bin
S=Homo sapiens OX=9606 GN=TF PE=1 SV=3;tr|Q06AH7|Q06AH7_HUMAN Beta-1 metal-binding glo

proteinase 1 (Interstitial collagenase) OS=Homo sapiens OX=9606 PE=2 SV=1;tr|Q53G96|Q53G96_H
ructose-bisphosphate aldolase A OS=Homo sapiens OX=9606 GN=ALDOA;tr|V9HWN7|V9HWN7_HU
S=Homo sapiens OX=9606 GN=CSTB PE=2 SV=1;sp|P04080|CYTB_HUMAN Cystatin-B OS=Homo sap
=Homo sapiens OX=9606 GN=ANXA1 PE=1 SV=2;tr|Q5TZZ9|Q5TZZ9_HUMAN Annexin OS=Homo sap
otein OS=Homo sapiens OX=9606 GN=PRNP PE=3 SV=2;tr|Q86XR1|Q86XR1_HUMAN Major prion pr
OS=Homo sapiens OX=9606 GN=KRT1 PE=3 SV=1;tr|H6VRG0|H6VRG0_HUMAN Cytokeratin-1 OS=H
e-3-phosphate dehydrogenase OS=Homo sapiens OX=9606 GN=GAPDH PE=1 SV=1;sp|P04406-2|G3P
tone H2A OS=Homo sapiens OX=9606 PE=3 SV=1;sp|Q96KK5|H2A1H_HUMAN Histone H2A type 1-H
f Amyloid-beta precursor protein OS=Homo sapiens OX=9606 GN=APP;sp|P05067|A4_HUMAN Amy

4, highly similar to Homo sapiens serpin peptidase inhibitor, clade B (ovalbumin), member 2 (SERPIN
in peptidase inhibitor, clade E (Nexin, plasminogen activator inhibitor type 1), member 1, isoform C
in peptidase inhibitor clade G member 1 OS=Homo sapiens OX=9606 GN=SERPING1 PE=2 SV=1;sp|
somal protein, large, P2, isoform CRA_a OS=Homo sapiens OX=9606 GN=RPLP2 PE=3 SV=1;sp|P053
=Homo sapiens OX=9606 GN=ITGB1 PE=1 SV=2;sp|P05556-2|ITB1_HUMAN Isoform 2 of Integrin be
o sapiens OX=9606 GN=GSN PE=1 SV=1;sp|P06396-2|GELS_HUMAN Isoform 2 of Gelsolin OS=Homo
OS=Homo sapiens OX=9606 PE=2 SV=1;sp|P06702|S10A9_HUMAN Protein S100-A9 OS=Homo sapie
Fragment) OS=Homo sapiens OX=9606 GN=S100A6 PE=1 SV=1;sp|P06703|S10A6_HUMAN Protein S
S=Homo sapiens OX=9606 GN=ENO1 PE=1 SV=2;tr|A0A024R4F1|A0A024R4F1_HUMAN 2-phospho-
cose-6-phosphate isomerase OS=Homo sapiens OX=9606 GN=GPI PE=1 SV=1;sp|P06744-2|G6PI_HU
eophosmin (Fragment) OS=Homo sapiens OX=9606 GN=NPM1 PE=2 SV=1;sp|P06748-2|NPM_HUM
ropomyosin alpha-3 chain OS=Homo sapiens OX=9606 GN=TPM3;tr|A0A087WWU8|A0A087WWU8_
96, highly similar to Glia-derived nexin OS=Homo sapiens OX=9606 PE=2 SV=1;tr|A0A024R498|A0A
actate dehydrogenase OS=Homo sapiens OX=9606 GN=LDHB PE=1 SV=1;sp|P07195|LDHB_HUMAN L
-isomerase OS=Homo sapiens OX=9606 GN=P4HB PE=1 SV=3;tr|A0A024R8S5|A0A024R8S5_HUMAN
Homo sapiens OX=9606 GN=CTSD PE=1 SV=1;tr|V9HWI3|V9HWI3_HUMAN Cathepsin D OS=Homo s
=Homo sapiens OX=9606 GN=ANXA2 PE=1 SV=2;tr|A0A024R5Z7|A0A024R5Z7_HUMAN Annexin OS=
iant Gaucher disease and variant metachromatic leukodystrophy) variant (Fragment) OS=Homo sapie
S=Homo sapiens OX=9606 GN=CTSL PE=1 SV=1;tr|B3KQK4|B3KQK4_HUMAN cDNA FLJ90619 fis, clo
omo sapiens OX=9606 GN=PFN1 PE=1 SV=2;tr|K7EJ44|K7EJ44_HUMAN Profilin OS=Homo sapiens O
Homo sapiens OX=9606 GN=CTSB PE=1 SV=3;tr|A0A024R374|A0A024R374_HUMAN Cathepsin B OS
t beta-1 OS=Homo sapiens OX=9606 GN=LAMB1 PE=1 SV=2;tr|G3XAI2|G3XAI2_HUMAN Laminin sub
-1 OS=Homo sapiens OX=9606 GN=THBS1 PE=1 SV=2;sp|P07996-2|TSP1_HUMAN Isoform 2 of Thro
t shock protein 90kDa alpha (Cytosolic), class B member 1, isoform CRA_a OS=Homo sapiens OX=960
chain (Fragment) OS=Homo sapiens OX=9606 PE=2 SV=1;tr|A4D1W7|A4D1W7_HUMAN Activin bet
llagen, type IV, alpha 2, isoform CRA_a OS=Homo sapiens OX=9606 GN=COL4A2 PE=4 SV=1;sp|P085
patocyte growth factor receptor OS=Homo sapiens OX=9606 GN=MET;sp|P08581|MET_HUMAN He
gment) OS=Homo sapiens OX=9606 PE=2 SV=1;sp|P08670|VIME_HUMAN Vimentin OS=Homo sapie
Homo sapiens OX=9606 GN=HEL-S-7 PE=2 SV=1;sp|P08758|ANXA5_HUMAN Annexin A5 OS=Homo s
S=Homo sapiens OX=9606 GN=GSTP1 PE=1 SV=1;tr|V9HWE9|V9HWE9_HUMAN GST class-pi OS=Ho

lectin OS=Homo sapiens OX=9606 PE=2 SV=1;sp|P09382|LEG1_HUMAN Galectin-1 OS=Homo sapie
sapiens OX=9606 GN=SPARC PE=1 SV=1;tr|D3DQH8|D3DQH8_HUMAN Osteonectin OS=Homo sapie
C1s subcomponent OS=Homo sapiens OX=9606 GN=C1S PE=1 SV=1;tr|A0A087X232|A0A087X232_H
ariant (Fragment) OS=Homo sapiens OX=9606 PE=2 SV=1;sp|P62979|RS27A_HUMAN Ubiquitin-40S
tein SET OS=Homo sapiens OX=9606 GN=SET;tr|A0A0C4DFV9|A0A0C4DFV9_HUMAN Protein SET OS

: shock 70 kDa protein 1B OS=Homo sapiens OX=9606 GN=HSPA1B PE=1 SV=1;sp|P0DMV8|HS71A_H
:Homo sapiens OX=9606 GN=HEL-S-72 PE=2 SV=1;sp|P0DP23|CALM1_HUMAN Calmodulin-1 OS=Hor
eceptor-type tyrosine-protein phosphatase F OS=Homo sapiens OX=9606 GN=PTPRF;sp|P10586|PTF
Homo sapiens OX=9606 GN=TXN PE=1 SV=3;tr|H9ZYJ2|H9ZYJ2_HUMAN Thioredoxin OS=Homo sapie
lum chaperone BiP OS=Homo sapiens OX=9606 GN=HSPA5 PE=1 SV=2;tr|V9HWB4|V9HWB4_HUMA
inin, gamma 1 (Formerly LAMB2), isoform CRA_a OS=Homo sapiens OX=9606 GN=LAMC1 PE=4 SV=1
nate 71 kDa protein OS=Homo sapiens OX=9606 GN=HSPA8 PE=1 SV=1;tr|V9HW22|V9HW22_HUMA
gen alpha-1(VI) chain OS=Homo sapiens OX=9606 GN=COL6A1 PE=1 SV=1;tr|A0A384P5H7|A0A384P
a-actinin cytoskeletal isoform OS=Homo sapiens OX=9606 GN=ACTN1 PE=2 SV=1;sp|P12814|ACTN1
adherin OS=Homo sapiens OX=9606 GN=CDH1 PE=2 SV=1;tr|D3XNU5|D3XNU5_HUMAN E-cadherin
ıma-inducible protein 30 preproprotein OS=Homo sapiens OX=9606 PE=2 SV=1;tr|A8K686|A8K686_
didymis secretory sperm binding protein OS=Homo sapiens OX=9606 PE=2 SV=1;tr|B4DPU3|B4DPU3
:ein disulfide-isomerase A4 OS=Homo sapiens OX=9606 GN=ERP70 PE=2 SV=1;sp|P13667|PDIA4_HU
controlled tumor protein OS=Homo sapiens OX=9606 GN=TPT1 PE=1 SV=1;tr|E9PJF7|E9PJF7_HUMA
cretory protein Li 37 OS=Homo sapiens OX=9606 GN=HEL-S-37 PE=2 SV=1;tr|Q53FI1|Q53FI1_HUMA
)59 glycoprotein OS=Homo sapiens OX=9606 GN=CD59;tr|E9PNW4|E9PNW4_HUMAN CD59 glycopr
ein kinase C substrate 80K-H isoform 4 (Fragment) OS=Homo sapiens OX=9606 GN=PRKCSH PE=2 SV=
dogen-1 OS=Homo sapiens OX=9606 GN=NID1;sp|P14543|NID1_HUMAN Nidogen-1 OS=Homo sapie
PKM OS=Homo sapiens OX=9606 GN=PKM PE=1 SV=4;tr|V9HWB8|V9HWB8_HUMAN Pyruvate kinas
:cretory sperm binding protein OS=Homo sapiens OX=9606 GN=TRA1 PE=2 SV=1;tr|V9HWP2|V9HWI
fis, clone ASTRO2015185, highly similar to POLIOVIRUS RECEPTOR OS=Homo sapiens OX=9606 PE=2
'328, highly similar to Beta-1,4-galactosyltransferase 1 OS=Homo sapiens OX=9606 PE=2 SV=1;tr|B7Z
., highly similar to Ezrin OS=Homo sapiens OX=9606 PE=2 SV=1;tr|V9HW42|V9HW42_HUMAN Epidic
=Homo sapiens OX=9606 GN=AREG PE=1 SV=2;tr|D6RFX5|D6RFX5_HUMAN Amphiregulin OS=Homo
alloproteinase inhibitor 2 OS=Homo sapiens OX=9606 GN=TIMP2 PE=3 SV=1;tr|A0A140VK57|A0A14
=Homo sapiens OX=9606 GN=CD44 PE=1 SV=3;sp|P16070-8|CD44_HUMAN Isoform 8 of CD44 antig
: of Integrin beta-4 OS=Homo sapiens OX=9606 GN=ITGB4;tr|Q59H46|Q59H46_HUMAN Integrin bet

tavinculin OS=Homo sapiens OX=9606 PE=2 SV=1;tr|A0A024QZN4|A0A024QZN4_HUMAN Metavinc
:rate mutase (Fragment) OS=Homo sapiens OX=9606 GN=PGAM1 PE=2 SV=1;tr|Q6FHK8|Q6FHK8_HU
statin OS=Homo sapiens OX=9606 GN=FST;tr|A0A024QZU6|A0A024QZU6_HUMAN Follistatin, isofo

Homo sapiens OX=9606 GN=EFNA1 PE=1 SV=2;tr|B2R7U1|B2R7U1_HUMAN Ephrin RBD domain-cont
Homo sapiens OX=9606 GN=FLNA PE=2 SV=1;sp|P21333|FLNA_HUMAN Filamin-A OS=Homo sapiens
ent) OS=Homo sapiens OX=9606 GN=MDK PE=1 SV=1;sp|P21741|MK_HUMAN Midkine OS=Homo sa
no sapiens OX=9606 GN=BGN PE=1 SV=2;tr|B4DNL4|B4DNL4_HUMAN Biglycan OS=Homo sapiens O
2, highly similar to CD9 antigen OS=Homo sapiens OX=9606 PE=2 SV=1;tr|A6NNI4|A6NNI4_HUMAN
Cadherin-3 OS=Homo sapiens OX=9606 GN=CDH3;sp|P22223|CADH3_HUMAN Cadherin-3 OS=Homo
ucleoside diphosphate kinase B OS=Homo sapiens OX=9606 GN=NME2;tr|Q32Q12|Q32Q12_HUMA
omo sapiens OX=9606 GN=SPRR1B PE=1 SV=2;tr|Q2I377|Q2I377_HUMAN Small proline rich protein
n factor-binding protein 4 OS=Homo sapiens OX=9606 GN=IGFBP4 PE=1 SV=2;tr|A0A024R1U8|A0A0
Homo sapiens OX=9606 PE=2 SV=1;sp|P23142-4|FBLN1_HUMAN Isoform C of Fibulin-1 OS=Homo sa
X2A of Integrin alpha-6 OS=Homo sapiens OX=9606 GN=ITGA6;sp|P23229-2|ITA6_HUMAN Isoform
lyl cis-trans isomerase OS=Homo sapiens OX=9606 GN=HEL-S-39 PE=2 SV=1;sp|P23284|PPIB_HUMA
scle isoform OS=Homo sapiens OX=9606 GN=CFL1 PE=1 SV=1;tr|E9PP50|E9PP50_HUMAN Cofilin, no
no sapiens OX=9606 GN=TNC PE=1 SV=3;sp|P24821-3|TENA_HUMAN Isoform 3 of Tenascin OS=Hor

oteasome subunit alpha type-3 OS=Homo sapiens OX=9606 GN=PSMA3;tr|Q6IB71|Q6IB71_HUMAN
oteasome subunit alpha type-4 OS=Homo sapiens OX=9606 GN=PSMA4;tr|H0YN18|H0YN18_HUMA
3 isoform b, variant (Fragment) OS=Homo sapiens OX=9606 PE=2 SV=1;tr|B4E0H8|B4E0H8_HUMAN

uminal protein 70 OS=Homo sapiens OX=9606 GN=HEL70 PE=2 SV=1;sp|P26038|MOES_HUMAN Mo
riant (Fragment) OS=Homo sapiens OX=9606 PE=2 SV=1;tr|V9HW88|V9HW88_HUMAN Calreticulin
eptor type 2 OS=Homo sapiens OX=9606 GN=IL1R2 PE=1 SV=1;sp|P27930-2|IL1R2_HUMAN Isoform
subunit alpha type OS=Homo sapiens OX=9606 PE=2 SV=1;tr|A0A109NGN6|A0A109NGN6_HUMAN F
omo sapiens OX=9606 GN=GRN PE=1 SV=2;tr|B4DJI2|B4DJI2_HUMAN cDNA FLJ53342, highly similar
OS=Homo sapiens OX=9606 GN=S100A2 PE=1 SV=3;tr|R4GN49|R4GN49_HUMAN Protein S100-A2
Ephrin type-A receptor 2 OS=Homo sapiens OX=9606 GN=EPHA2;tr|A0A024QZA8|A0A024QZA8_HU
uminal protein 107 OS=Homo sapiens OX=9606 GN=HEL107 PE=2 SV=1;tr|Q53EM5|Q53EM5_HUMA
tory protein Li 34 OS=Homo sapiens OX=9606 GN=HEL-S-34 PE=2 SV=1;sp|P30086|PEBP1_HUMAN P
de-isomerase OS=Homo sapiens OX=9606 PE=2 SV=1;tr|B3KQT2|B3KQT2_HUMAN Protein disulfide-
kinase receptor UFO OS=Homo sapiens OX=9606 GN=AXL PE=1 SV=4;sp|P30530-2|UFO_HUMAN Isof
ndecan-4 OS=Homo sapiens OX=9606 GN=SDC4;sp|P31431|SDC4_HUMAN Syndecan-4 OS=Homo sa
3, highly similar to Heterogeneous nuclear ribonucleoprotein H OS=Homo sapiens OX=9606 PE=2 SV=
of 14-3-3 protein beta/alpha OS=Homo sapiens OX=9606 GN=YWHAB;tr|V9HWD6|V9HWD6_HUMA
igma OS=Homo sapiens OX=9606 GN=SFN PE=1 SV=1;sp|P31947-2|1433S_HUMAN Isoform 2 of 14-
OS=Homo sapiens OX=9606 GN=HEL-S-43 PE=2 SV=1;tr|B2R5H0|B2R5H0_HUMAN Protein S100 OS
Neural cell adhesion molecule L1 OS=Homo sapiens OX=9606 GN=L1CAM;sp|P32004-2|L1CAM_HU
pican-1 OS=Homo sapiens OX=9606 PE=2 SV=1;sp|P35052|GPC1_HUMAN Glypican-1 OS=Homo sapi

-2 OS=Homo sapiens OX=9606 GN=THBS2 PE=1 SV=2;tr|A0A3B3ITK0|A0A3B3ITK0_HUMAN Thromb
mo sapiens OX=9606 GN=FBN2 PE=1 SV=3;tr|D6RJI3|D6RJI3_HUMAN Fibrillin-2 OS=Homo sapiens O
osin, heavy polypeptide 9, non-muscle, isoform CRA_a OS=Homo sapiens OX=9606 GN=MYH9 PE=3 S
in peptidase inhibitor, clade B (Ovalbumin), member 5, isoform CRA_b OS=Homo sapiens OX=9606 G
agment) OS=Homo sapiens OX=9606 GN=TAGLN2 PE=1 SV=1;tr|A0A384MTL2|A0A384MTL2_HUMA
e XVIII, alpha 1, isoform CRA_d OS=Homo sapiens OX=9606 GN=COL18A1 PE=4 SV=1;sp|P39060-2|C
drogenase OS=Homo sapiens OX=9606 GN=HEL-S-32 PE=2 SV=1;sp|P40925|MDHC_HUMAN Malate
in (Fragment) OS=Homo sapiens OX=9606 GN=IQGAP1 PE=2 SV=1;tr|A0A0J9YXZ5|A0A0J9YXZ5_HU
protein subunit beta OS=Homo sapiens OX=9606 GN=CAPZB PE=1 SV=1;tr|B1AK85|B1AK85_HUMA
e factor pathway inhibitor 2 (Fragment) OS=Homo sapiens OX=9606 PE=2 SV=1;sp|P48307-2|TFPI2_
OS=Homo sapiens OX=9606 GN=FASN PE=1 SV=3;tr|A0A0U1RQF0|A0A0U1RQF0_HUMAN 3-hydro
OS=Homo sapiens OX=9606 GN=VEGFC PE=2 SV=1;sp|P49767|VEGFC_HUMAN Vascular endothelia
ciation inhibitor (Fragment) OS=Homo sapiens OX=9606 GN=GDI2 PE=1 SV=1;sp|P50395-2|GDIB_HU
ion molecule OS=Homo sapiens OX=9606 GN=BCAM PE=1 SV=2;tr|A0A068W6H0|A0A068W6H0_HU
ation inhibitor 1 (Fragment) OS=Homo sapiens OX=9606 GN=ARHGDIA PE=1 SV=8;tr|V9HWE8|V9HW

lase 1 OS=Homo sapiens OX=9606 GN=CTSC PE=1 SV=2;tr|B4DJQ8|B4DJQ8_HUMAN Cathepsin C OS
Microfibrillar-associated protein 2 OS=Homo sapiens OX=9606 GN=MFAP2;sp|P55001-2|MFAP2_HU
sfer protein OS=Homo sapiens OX=9606 GN=PLTP PE=1 SV=1;tr|B3KUE5|B3KUE5_HUMAN Phospho
gment) OS=Homo sapiens OX=9606 GN=VCP PE=2 SV=2;tr|V9HW80|V9HW80_HUMAN 15S Mg(2+)-
astrocyte-derived neurotrophic factor OS=Homo sapiens OX=9606 GN=MANF PE=1 SV=3;tr|A8K878|

Cadherin-13 OS=Homo sapiens OX=9606 GN=CDH13;sp|P55290|CAD13_HUMAN Cadherin-13 OS=Ho
Homo sapiens OX=9606 GN=CD81 PE=1 SV=1;tr|E9PRJ8|E9PRJ8_HUMAN Tetraspanin (Fragment) OS
e isomerase OS=Homo sapiens OX=9606 PE=2 SV=1;tr|V9HWK1|V9HWK1_HUMAN Triosephosphate
unit alpha type OS=Homo sapiens OX=9606 GN=PSMA6 PE=1 SV=1;tr|G3V295|G3V295_HUMAN Pro
bulin OS=Homo sapiens OX=9606 GN=B2M PE=1 SV=1;tr|B4E0X1|B4E0X1_HUMAN Beta-2-microglo
cretory protein E1 (Fragment) OS=Homo sapiens OX=9606 PE=2 SV=1;tr|A0A024R6C0|A0A024R6C0
gamma OS=Homo sapiens OX=9606 GN=YWHAG PE=1 SV=2;tr|B3KNB4|B3KNB4_HUMAN cDNA FLJ1
psilon OS=Homo sapiens OX=9606 GN=YWHAE PE=1 SV=1;tr|V9HW98|V9HW98_HUMAN Epididymi
OS=Homo sapiens OX=9606 GN=TMSB4X PE=2 SV=1;sp|P62328|TYB4_HUMAN Thymosin beta-4 OS

;=Homo sapiens OX=9606 GN=HIST1H4H PE=2 SV=1;tr|B2R4R0|B2R4R0_HUMAN Histone H4 OS=Hor
-trans isomerase A OS=Homo sapiens OX=9606 GN=PPIA PE=1 SV=2;tr|A8K486|A8K486_HUMAN Pe
:is-trans isomerase FKBP1A OS=Homo sapiens OX=9606 GN=FKBP1A PE=1 SV=2;tr|Q5W0X3|Q5W0X
inal protein 4 OS=Homo sapiens OX=9606 GN=YWHAZ PE=2 SV=1;sp|P63104|1433Z_HUMAN 14-3-3
36, highly similar to Actin, cytoplasmic 2 OS=Homo sapiens OX=9606 PE=2 SV=1;tr|B4E3A4|B4E3A4_
10 OS=Homo sapiens OX=9606 GN=TMSB10 PE=1 SV=2;tr|D6W5K2|D6W5K2_HUMAN Thymosin, be
yrosine-protein phosphatase non-receptor type substrate 1 OS=Homo sapiens OX=9606 GN=SIRPA;s
OS=Homo sapiens OX=9606 GN=JAG1 PE=1 SV=3;tr|B2R6U9|B2R6U9_HUMAN Delta-like protein OS
inase-associated lipocalin OS=Homo sapiens OX=9606 GN=LCN2 PE=1 SV=2;tr|B2ZDQ1|B2ZDQ1_HUI
2 OS=Homo sapiens OX=9606 GN=NUCB2 PE=1 SV=2;sp|P80303-2|NUCB2_HUMAN Isoform 2 of Nuc
lomo sapiens OX=9606 GN=FBLN2 PE=2 SV=1;sp|P98095|FBLN2_HUMAN Fibulin-2 OS=Homo sapien

se-associated protein 1 (Fragment) OS=Homo sapiens OX=9606 GN=CAP1 PE=1 SV=1;tr|Q5T0R6|Q5T
. isoform HRG-beta2 OS=Homo sapiens OX=9606 GN=NRG1 PE=2 SV=1;tr|B0FYA7|B0FYA7_HUMAN I
-1(VII) chain OS=Homo sapiens OX=9606 GN=COL7A1 PE=1 SV=2;sp|Q02388-2|CO7A1_HUMAN Isofc
ysine 5-dioxygenase OS=Homo sapiens OX=9606 PE=2 SV=1;sp|Q02809|PLOD1_HUMAN Procollager
1 OS=Homo sapiens OX=9606 GN=NUCB1 PE=1 SV=4;tr|A8K7Q1|A8K7Q1_HUMAN Nucleobindin-1 O
yloid beta (A4)-like protein 2, isoform CRA_a OS=Homo sapiens OX=9606 GN=APLP2 PE=4 SV=1;tr|A(
didymis secretory sperm binding protein OS=Homo sapiens OX=9606 PE=2 SV=1;sp|Q06830|PRDX1_
i-1(XVI) chain OS=Homo sapiens OX=9606 GN=COL16A1 PE=1 SV=1;sp|Q07092-2|COGA1_HUMAN Is
pithelial discoidin domain-containing receptor 1 OS=Homo sapiens OX=9606 GN=DDR1;sp|Q08345-2
:tin-3-binding protein (Fragment) OS=Homo sapiens OX=9606 GN=LGALS3BP PE=2 SV=1;sp|Q08380|
-acetylgalactosaminyltransferase OS=Homo sapiens OX=9606 GN=GALNT2 PE=3 SV=1;tr|B7Z6K2|B7Z
g fibulin-like extracellular matrix protein 1 isoform b variant (Fragment) OS=Homo sapiens OX=9606
:d protein 1 OS=Homo sapiens OX=9606 GN=FSTL1 PE=1 SV=1;sp|Q12841-2|FSTL1_HUMAN Isoform
Contactin-1 OS=Homo sapiens OX=9606 GN=CNTN1;tr|A0A024R104|A0A024R104_HUMAN Contacti
(Fragment) OS=Homo sapiens OX=9606 GN=SEMA3F PE=1 SV=8;tr|Q59G50|Q59G50_HUMAN Sema
yrosine kinase 7 isoform a variant (Fragment) OS=Homo sapiens OX=9606 PE=2 SV=1;sp|Q13308-5|I
agment) OS=Homo sapiens OX=9606 GN=MSLN PE=1 SV=8;sp|Q13421-4|MSLN_HUMAN Isoform 4 (
id metalloproteinase domain-containing protein 9 OS=Homo sapiens OX=9606 GN=ADAM9 PE=1 SV=
nin beta 3 isoform 1 (Fragment) OS=Homo sapiens OX=9606 GN=LAMB3 PE=2 SV=1;sp|Q13751|LAM
it gamma-2 OS=Homo sapiens OX=9606 GN=LAMC2 PE=1 SV=2;sp|Q13753-2|LAMC2_HUMAN Isofoi
oha-dystroglycan OS=Homo sapiens OX=9606 GN=DAG1 PE=4 SV=1;sp|Q14118|DAG1_HUMAN Dysti
2, highly similar to Growth-arrest-specific protein 6 OS=Homo sapiens OX=9606 PE=2 SV=1;sp|Q1439

rming growth factor beta-binding protein 2 OS=Homo sapiens OX=9606 GN=LTBP2 PE=1 SV=1;tr|G3\
'rotein disulfide-isomerase A6 OS=Homo sapiens OX=9606 GN=PDIA6;tr|A0A384NPU5|A0A384NPU5
ectin OS=Homo sapiens OX=9606 GN=PLEC;sp|Q15149-8|PLEC_HUMAN Isoform 8 of Plectin OS=Hor
ptor-related 1 (Fragment) OS=Homo sapiens OX=9606 GN=PVRL1 PE=4 SV=1;sp|Q15223-3|NECT1_H
e phosphatase, receptor type, K variant (Fragment) OS=Homo sapiens OX=9606 PE=2 SV=1;tr|B4DH(
isforming growth factor-beta-induced protein ig-h3 (Fragment) OS=Homo sapiens OX=9606 GN=TGFI
h factor-binding protein 7 OS=Homo sapiens OX=9606 GN=IGFBP7 PE=1 SV=1;sp|Q16270-2|IBP7_HU
ılar tissue protein Li 61 OS=Homo sapiens OX=9606 PE=2 SV=1;sp|Q16610|ECM1_HUMAN Extracellu
no sapiens OX=9606 PE=2 SV=1;tr|A0A384MEG1|A0A384MEG1_HUMAN Fascin OS=Homo sapiens O
ide cyclotransferase OS=Homo sapiens OX=9606 GN=QPCT PE=1 SV=1;sp|Q16769-2|QPCT_HUMAN
1811249, isoform CRA_e OS=Homo sapiens OX=9606 GN=LAMA3 PE=1 SV=1;sp|Q16787-1|LAMA3_I
e protein 132A OS=Homo sapiens OX=9606 GN=TMEM132A PE=1 SV=1;sp|Q24JP5-2|T132A_HUMAN
omo sapiens OX=9606 GN=ANXA8L1 PE=1 SV=1;sp|Q5VT79|AXA81_HUMAN Annexin A8-like protein
orotein 5 OS=Homo sapiens OX=9606 GN=RAET1G PE=1 SV=1;sp|Q6H3X3-2|ULBP5_HUMAN Isoform
Willebrand factor A domain containing 1, isoform CRA_c OS=Homo sapiens OX=9606 GN=VWA1 PE=

CD109 antigen OS=Homo sapiens OX=9606 GN=CD109;sp|Q6YHK3|CD109_HUMAN CD109 antigen
fis, clone HLUNG2001507, highly similar to Amphoterin-induced protein 2 OS=Homo sapiens OX=96
lysis-stimulated lipoprotein receptor OS=Homo sapiens OX=9606 GN=LSR;sp|Q86X29-5|LSR_HUMA
ncer-binding protein 1 OS=Homo sapiens OX=9606 GN=AEBP1 PE=1 SV=1;tr|B4DJB3|B4DJB3_HUMA
Extracellular sulfatase Sulf-2 OS=Homo sapiens OX=9606 GN=SULF2;sp|Q8IWU5|SULF2_HUMAN Ext
vertase subtilisin/kexin type 9 OS=Homo sapiens OX=9606 GN=PCSK9 PE=1 SV=3;tr|A0A669KBG0|A
, highly similar to Programmed cell death 6-interacting protein OS=Homo sapiens OX=9606 PE=2 SV=
OS=Homo sapiens OX=9606 GN=FAM3C PE=1 SV=1;tr|C9JP35|C9JP35_HUMAN Protein FAM3C (Fra
nolog OS=Homo sapiens OX=9606 GN=PXDN PE=1 SV=2;tr|H7C1W1|H7C1W1_HUMAN Peroxidasin h
HTRA1 OS=Homo sapiens OX=9606 GN=HTRA1 PE=1 SV=1;tr|Q05DJ8|Q05DJ8_HUMAN HTRA1 prot
Neuronal cell adhesion molecule OS=Homo sapiens OX=9606 GN=NRCAM;sp|Q92823-3|NRCAM_H
protein 1 OS=Homo sapiens OX=9606 GN=GLG1 PE=1 SV=2;sp|Q92896-3|GSLG1_HUMAN Isoform
ide-3-kinase-interacting protein 1 (Fragment) OS=Homo sapiens OX=9606 GN=PIK3IP1 PE=1 SV=8;sp
lycase OS=Homo sapiens OX=9606 GN=HEL-S-67p PE=2 SV=1;sp|Q99497|PARK7_HUMAN Parkinson
lopeptidase OS=Homo sapiens OX=9606 PE=2 SV=1;tr|Q6I9U9|Q6I9U9_HUMAN Asparaginyl endope
Collagen alpha-1(XII) chain OS=Homo sapiens OX=9606 GN=COL12A1;tr|D6RGG3|D6RGG3_HUMAN
OS=Homo sapiens OX=9606 GN=SEMA3C PE=2 SV=2;tr|B4E2I9|B4E2I9_HUMAN Semaphorin-3C OS
edoxin domain-containing protein 17 OS=Homo sapiens OX=9606 PE=2 SV=1;sp|Q9BRA2|TXD17_HU
45 kDa calcium-binding protein OS=Homo sapiens OX=9606 GN=SDF4;tr|G3V1E2|G3V1E2_HUMAN
Tubulointerstitial nephritis antigen-like OS=Homo sapiens OX=9606 GN=TINAGL1;sp|Q9GZM7|TINAL
nding glutamic acid-rich-like protein 3 OS=Homo sapiens OX=9606 GN=SH3BGRL3 PE=1 SV=1;tr|Q86Z
Semaphorin-4B OS=Homo sapiens OX=9606 GN=SEMA4B;sp|Q9NPR2|SEM4B_HUMAN Semaphorin
=Homo sapiens OX=9606 GN=TENM2 PE=1 SV=1;sp|Q9NT68-2|TEN2_HUMAN Isoform 2 of Teneuri
ed protein CRIM1 (Fragment) OS=Homo sapiens OX=9606 GN=CRIM1 PE=4 SV=1;sp|Q9NZV1|CRIM1
receptor negative regulator OS=Homo sapiens OX=9606 GN=PTGFRN PE=1 SV=2;tr|Q1WWL2|Q1W
protein 3 OS=Homo sapiens OX=9606 GN=DKK3 PE=1 SV=2;tr|F6SYF8|F6SYF8_HUMAN Dickkopf-re
S=Homo sapiens OX=9606 PE=2 SV=1;sp|Q9UBR2|CATZ_HUMAN Cathepsin Z OS=Homo sapiens OX
e A4 OS=Homo sapiens OX=9606 GN=CPA4 PE=1 SV=1;sp|Q9UI42-2|CBPA4_HUMAN Isoform 2 of C
f Collagen alpha-1(XVII) chain OS=Homo sapiens OX=9606 GN=COL17A1;tr|D3DRA2|D3DRA2_HUM
ein C receptor OS=Homo sapiens OX=9606 GN=PROCR PE=1 SV=1;tr|A0A0U1RQQ4|A0A0U1RQQ4_
krein-related peptidase 5 transcript variant 5 OS=Homo sapiens OX=9606 GN=KLK5 PE=2 SV=1;tr|A0
omolog OS=Homo sapiens OX=9606 GN=LOXL2 PE=2 SV=1;sp|Q9Y4K0|LOXL2_HUMAN Lysyl oxidase
umorigenicity 14 protein homolog OS=Homo sapiens OX=9606 PE=2 SV=1;sp|Q9Y5Y6|ST14_HUMA
esion molecule 1 OS=Homo sapiens OX=9606 GN=F11R PE=3 SV=1;tr|Q6FIB4|Q6FIB4_HUMAN Junc
nectin OS=Homo sapiens OX=9606 GN=FN1 PE=4 SV=1;tr|B7ZLE5|B7ZLE5_HUMAN Fibronectin OS=
0 protein 9 (Fragment) OS=Homo sapiens OX=9606 GN=KLK10 PE=2 SV=1;tr|A0A024R4N7|A0A024R
ascin C (Hexabrachion), isoform CRA_a OS=Homo sapiens OX=9606 GN=TNC PE=3 SV=1;tr|Q4LE33|Q
aran sulfate proteoglycan 2 (Perlecan), isoform CRA_b OS=Homo sapiens OX=9606 GN=HSPG2 PE=4
olony-stimulating factor (Fragment) OS=Homo sapiens OX=9606 GN=CSF3 PE=2 SV=1;tr|A0A0B4U5E
class I antigen OS=Homo sapiens OX=9606 GN=HLA-C PE=3 SV=1;tr|A0A0S4T3H1|A0A0S4T3H1_HU
|A0A0S4XRM0_HUMAN;tr|A0A0U5PUM0|A0A0U5PUM0_HUMAN;tr|A0A6B9VT42|A0A6B9VT42_

ronectin OS=Homo sapiens OX=9606 PE=2 SV=1;sp|P02751-10|FINC_HUMAN Isoform 10 of Fibrone
ubcomponent C1r OS=Homo sapiens OX=9606 GN=C1R PE=1 SV=3;tr|Q53HU9|Q53HU9_HUMAN Co
, highly similar to Homo sapiens desmocollin type 4 OS=Homo sapiens OX=9606 PE=2 SV=1
E1 OS=Homo sapiens OX=9606 GN=RNASE7 PE=3 SV=1;tr|A8K9I2|A8K9I2_HUMAN cDNA FLJ75277

, highly similar to Matrix metalloproteinase-9 (EC3.4.24.35) OS=Homo sapiens OX=9606 PE=2 SV=1;s

plasminogen activator OS=Homo sapiens OX=9606 GN=PLAU PE=1 SV=1;tr|Q5PY49|Q5PY49_HUMA
ntigen (Fragment) OS=Homo sapiens OX=9606 GN=HLA-C PE=3 SV=1;tr|F6IQL9|F6IQL9_HUMAN MH
nelial growth factor A (Fragment) OS=Homo sapiens OX=9606 GN=VEGFA PE=1 SV=1;sp|P15692-2|VE
issue growth factor OS=Homo sapiens OX=9606 GN=CTGF PE=2 SV=1;tr|B3KRV6|B3KRV6_HUMAN cl

MAN Keratin 14 (Epidermolysis bullosa simplex, Dowling-Meara, Koebner), isoform CRA_a OS=Homo
3|K2C6A_HUMAN Keratin, type II cytoskeletal 6A OS=Homo sapiens OX=9606 GN=KRT6A PE=1 SV=3;t

J54081, highly similar to Keratin, type II cytoskeletal 5 OS=Homo sapiens OX=9606 PE=2 SV=1;tr|B4D

ratin, type I cytoskeletal 17 OS=Homo sapiens OX=9606 GN=KRT17 PE=1 SV=2;;tr|Q14666|Q14666_I
MAN Uncharacterized protein DKFZp781M0386 OS=Homo sapiens OX=9606 GN=DKFZp781M0386 PE
AN Chloride intracellular channel protein OS=Homo sapiens OX=9606 GN=CLIC1 PE=2 SV=2;tr|Q53FB
fhydryl oxidase OS=Homo sapiens OX=9606 GN=QSCN6 PE=2 SV=1;sp|O00391-2|QSOX1_HUMAN Iso
sidase OS=Homo sapiens OX=9606 PE=2 SV=1;tr|A8K6D3|A8K6D3_HUMAN Beta-mannosidase OS=H
omo sapiens OX=9606 GN=AGRN;sp|O00468-3|AGRIN_HUMAN Isoform 3 of Agrin OS=Homo sapiens

OS=Homo sapiens OX=9606 GN=CCN1 PE=1 SV=1;tr|B4DI61|B4DI61_HUMAN cDNA FLJ58182, highly
/=1;tr|A0A024R5U5|A0A024R5U5_HUMAN Disintegrin and metalloproteinase domain-containing pr
A_HUMAN Myosin regulatory light chain 12A OS=Homo sapiens OX=9606 GN=MYL12A PE=1 SV=2;sp

unitz-type protease inhibitor 1 (Fragment) OS=Homo sapiens OX=9606 GN=SPINT1 PE=1 SV=1;tr|B4D
N4_HUMAN Alpha-actinin-4 OS=Homo sapiens OX=9606 GN=ACTN4 PE=1 SV=2;tr|Q96BG6|Q96BG6

stone H2B OS=Homo sapiens OX=9606 GN=HIST1H2BN PE=3 SV=1;tr|I6L9F7|I6L9F7_HUMAN Histon
1;tr|A0A024R141|A0A024R141_HUMAN Cathepsin L2, isoform CRA_a OS=Homo sapiens OX=9606 G
_HUMAN Isoform 2 of WD repeat-containing protein 1 OS=Homo sapiens OX=9606 GN=WDR1;tr|B3K
emaphorin-7A OS=Homo sapiens OX=9606 GN=SEMA7A;tr|F5GYX3|F5GYX3_HUMAN Semaphorin-7A
S=Homo sapiens OX=9606 GN=FLNB;sp|O75369|FLNB_HUMAN Filamin-B OS=Homo sapiens OX=960
apiens OX=9606 GN=SERPINB7 PE=1 SV=1;tr|A8K3Q8|A8K3Q8_HUMAN cDNA FLJ75069, highly simil
form 3 of Attractin OS=Homo sapiens OX=9606 GN=ATRN;sp|O75882-2|ATRN_HUMAN Isoform 2 of
1;tr|Q6FHC9|Q6FHC9_HUMAN STC2 protein (Fragment) OS=Homo sapiens OX=9606 GN=STC2 PE=2

V=1;sp|O95084|PRS23_HUMAN Serine protease 23 OS=Homo sapiens OX=9606 GN=PRSS23 PE=1 SV
2RBR3_HUMAN LY6/PLAUR domain containing 3 OS=Homo sapiens OX=9606 GN=LYPD3 PE=2 SV=1;t
MAN Follistatin-like 3 (Secreted glycoprotein), isoform CRA_a OS=Homo sapiens OX=9606 GN=FSTL3
ctate dehydrogenase A chain OS=Homo sapiens OX=9606 GN=LDHA PE=1 SV=2;sp|P00338-3|LDHA_
artate aminotransferase, mitochondrial OS=Homo sapiens OX=9606 GN=GOT2;tr|A0A024R6W0|A0A
phoglycerate kinase OS=Homo sapiens OX=9606 GN=HEL-S-68p PE=2 SV=1;sp|P00558|PGK1_HUMA
minogen activator OS=Homo sapiens OX=9606 GN=PLAT PE=1 SV=1;tr|B4DNJ1|B4DNJ1_HUMAN T-pl
Homo sapiens OX=9606 GN=C3 PE=1 SV=2;tr|B4E216|B4E216_HUMAN C3-beta-c OS=Homo sapiens
etalloproteinase inhibitor 1 OS=Homo sapiens OX=9606 GN=TIMP1 PE=2 SV=1;tr|B3KQF4|B3KQF4_H

I Low density lipoprotein receptor (Fragment) OS=Homo sapiens OX=9606 GN=LDLR PE=4 SV=1;tr|J3
sapiens OX=9606 GN=INS PE=1 SV=1;tr|I3WAC9|I3WAC9_HUMAN Insulin OS=Homo sapiens OX=960
MAN Immunoglobulin kappa constant (Fragment) OS=Homo sapiens OX=9606 GN=IGKC PE=1 SV=1;t
PE=2 SV=1;sp|P01876|IGHA1_HUMAN Immunoglobulin heavy constant alpha 1 OS=Homo sapiens O
Collagen alpha-1(IV) chain (Fragment) OS=Homo sapiens OX=9606 GN=COL4A1 PE=1 SV=1;tr|A0A6G
is secretory sperm binding protein OS=Homo sapiens OX=9606 PE=2 SV=1;sp|P02545-6|LMNA_HUM
OE_HUMAN Apolipoprotein E OS=Homo sapiens OX=9606 GN=APOE PE=1 SV=1;tr|A0A346DBY2|A0A

Homo sapiens OX=9606 GN=FN1;sp|P02751-11|FINC_HUMAN Isoform 11 of Fibronectin OS=Homo s
ding protein OS=Homo sapiens OX=9606 GN=GC;tr|V9HWI6|V9HWI6_HUMAN Gc-globulin OS=Homo

UMAN Matrix metalloproteinase 1 preproprotein variant (Fragment) OS=Homo sapiens OX=9606 PE
JMAN Fructose-bisphosphate aldolase OS=Homo sapiens OX=9606 GN=HEL-S-87p PE=2 SV=1;tr|J3KP
iens OX=9606 GN=CSTB PE=1 SV=2;tr|A0A1W2PS52|A0A1W2PS52_HUMAN Cystatin-B OS=Homo sa
iens OX=9606 GN=ANXA1 PE=2 SV=1;tr|A0A4D5RAI5|A0A4D5RAI5_HUMAN Annexin OS=Homo sapi
rotein (Fragment) OS=Homo sapiens OX=9606 GN=PRNP PE=3 SV=1;tr|Q6SES1|Q6SES1_HUMAN Maj
omo sapiens OX=9606 GN=KRT1 PE=3 SV=1;tr|H6VRF9|H6VRF9_HUMAN Cytokeratin-1 OS=Homo sa
_HUMAN Isoform 2 of Glyceraldehyde-3-phosphate dehydrogenase OS=Homo sapiens OX=9606 GN
OS=Homo sapiens OX=9606 GN=H2AC12 PE=1 SV=3;sp|P04908|H2A1B_HUMAN Histone H2A type
loid-beta precursor protein OS=Homo sapiens OX=9606 GN=APP PE=1 SV=3;sp|P05067-11|A4_HUM

32), mRNA OS=Homo sapiens OX=9606 PE=2 SV=1;sp|P05120|PAI2_HUMAN Plasminogen activator i
RA_b OS=Homo sapiens OX=9606 GN=SERPINE1 PE=3 SV=1;sp|P05121|PAI1_HUMAN Plasminogen a
P05155-2|IC1_HUMAN Isoform 2 of Plasma protease C1 inhibitor OS=Homo sapiens OX=9606 GN=SE
87|RLA2_HUMAN 60S acidic ribosomal protein P2 OS=Homo sapiens OX=9606 GN=RPLP2 PE=1 SV=1
ta-1 OS=Homo sapiens OX=9606 GN=ITGB1;sp|P05556-5|ITB1_HUMAN Isoform 5 of Integrin beta-1
sapiens OX=9606 GN=GSN;tr|A0A384MEF1|A0A384MEF1_HUMAN Actin-depolymerizing factor OS

S100-A6 OS=Homo sapiens OX=9606 GN=S100A6 PE=1 SV=1;tr|B2R577|B2R577_HUMAN Protein S1
D-glycerate hydro-lyase OS=Homo sapiens OX=9606 GN=HEL-S-17 PE=2 SV=1;tr|A0A2R8Y6G6|A0A2F
MAN Isoform 2 of Glucose-6-phosphate isomerase OS=Homo sapiens OX=9606 GN=GPI;tr|A0A1W6A
AN Isoform 2 of Nucleophosmin OS=Homo sapiens OX=9606 GN=NPM1;tr|A0A0S2Z491|A0A0S2Z491
_HUMAN Tropomyosin alpha-3 chain OS=Homo sapiens OX=9606 GN=TPM3 PE=1 SV=1;sp|P06753-2
024R498_HUMAN Serpin peptidase inhibitor, clade E (Nexin, plasminogen activator inhibitor type 1),
L-lactate dehydrogenase B chain OS=Homo sapiens OX=9606 GN=LDHB PE=1 SV=2;tr|Q5U077|Q5U0
J Protein disulfide-isomerase OS=Homo sapiens OX=9606 GN=P4HB PE=2 SV=1;tr|H7BZ94|H7BZ94_H
apiens OX=9606 GN=HEL-S-130P PE=2 SV=1;tr|A0A1B0GWE8|A0A1B0GWE8_HUMAN Cathepsin D O
Homo sapiens OX=9606 GN=ANXA2 PE=2 SV=1;tr|V9HW65|V9HW65_HUMAN Annexin OS=Homo sa
ens OX=9606 PE=2 SV=1;tr|A0A024QZQ2|A0A024QZQ2_HUMAN Prosaposin (Variant Gaucher diseas
ne PLACE1002374, highly similar to Cathepsin L OS=Homo sapiens OX=9606 PE=2 SV=1;tr|A5PLM9|A

=Homo sapiens OX=9606 GN=CTSB PE=2 SV=1;tr|B4DL49|B4DL49_HUMAN Cathepsin B OS=Homo sa
bunit beta-1 OS=Homo sapiens OX=9606 GN=LAMB1 PE=1 SV=1;tr|Q8TAS6|Q8TAS6_HUMAN LAMB1

06 GN=HSP90AB1 PE=3 SV=1;sp|P08238|HS90B_HUMAN Heat shock protein HSP 90-beta OS=Homo
a-A chain OS=Homo sapiens OX=9606 GN=INHBA PE=2 SV=1;sp|P08476|INHBA_HUMAN Inhibin bet
72|CO4A2_HUMAN Collagen alpha-2(IV) chain OS=Homo sapiens OX=9606 GN=COL4A2 PE=1 SV=4;t
patocyte growth factor receptor OS=Homo sapiens OX=9606 GN=MET PE=1 SV=4;tr|A0A024R728|A0
ens OX=9606 GN=VIM PE=1 SV=4;tr|B0YJC4|B0YJC4_HUMAN Vimentin OS=Homo sapiens OX=9606 G
sapiens OX=9606 GN=ANXA5 PE=1 SV=2;tr|E9PHT9|E9PHT9_HUMAN Annexin OS=Homo sapiens OX
omo sapiens OX=9606 GN=HEL-S-22 PE=2 SV=1;sp|P09211|GSTP1_HUMAN Glutathione S-transferase

ens OX=9606 GN=SPARC PE=3 SV=1;tr|B2RDL6|B2RDL6_HUMAN Osteonectin OS=Homo sapiens OX=
UMAN Complement C1s subcomponent OS=Homo sapiens OX=9606 GN=C1S PE=1 SV=1;tr|B3KNX0|
ribosomal protein S27a OS=Homo sapiens OX=9606 GN=RPS27A PE=1 SV=2;tr|F5GXK7|F5GXK7_HUM
=Homo sapiens OX=9606 GN=SET PE=1 SV=1;sp|Q01105-4|SET_HUMAN Isoform 4 of Protein SET OS

HUMAN Heat shock 70 kDa protein 1A OS=Homo sapiens OX=9606 GN=HSPA1A PE=1 SV=1;sp|P0DM
mo sapiens OX=9606 GN=CALM1 PE=1 SV=1;sp|P0DP24|CALM2_HUMAN Calmodulin-2 OS=Homo sa
PRF_HUMAN Receptor-type tyrosine-protein phosphatase F OS=Homo sapiens OX=9606 GN=PTPRF P
ns OX=9606 GN=TXN PE=2 SV=1;sp|P10599-2|THIO_HUMAN Isoform 2 of Thioredoxin OS=Homo sa
N 78 kDa glucose-regulated protein OS=Homo sapiens OX=9606 GN=HEL-S-89n PE=2 SV=1
;sp|P11047|LAMC1_HUMAN Laminin subunit gamma-1 OS=Homo sapiens OX=9606 GN=LAMC1 PE=
N Epididymis luminal protein 33 OS=Homo sapiens OX=9606 GN=HEL-S-72p PE=2 SV=1;tr|E9PKE3|E
5H7_HUMAN Collagen, type VI, alpha 1, isoform CRA_b OS=Homo sapiens OX=9606 GN=COL6A1 PE=
_HUMAN Alpha-actinin-1 OS=Homo sapiens OX=9606 GN=ACTN1 PE=1 SV=2;sp|P12814-3|ACTN1_H
1 OS=Homo sapiens OX=9606 GN=CDH1 PE=4 SV=1;tr|A0A0U2ZQU7|A0A0U2ZQU7_HUMAN E-cadh
HUMAN cDNA FLJ77316, highly similar to Homo sapiens interferon, gamma-inducible protein 30 (IFI3
_HUMAN cDNA FLJ56548, highly similar to Elongation factor 2 OS=Homo sapiens OX=9606 PE=2 SV=
MAN Protein disulfide-isomerase A4 OS=Homo sapiens OX=9606 GN=PDIA4 PE=1 SV=2;tr|A0A499FI
N Translationally-controlled tumor protein (Fragment) OS=Homo sapiens OX=9606 GN=TPT1 PE=1 S
N L-plastin variant (Fragment) OS=Homo sapiens OX=9606 PE=2 SV=1;tr|A0A024RDT4|A0A024RDT4
otein OS=Homo sapiens OX=9606 PE=1 SV=1;tr|A0A2U3TZL5|A0A2U3TZL5_HUMAN CD59 glycoprot
=1;tr|A2VCQ4|A2VCQ4_HUMAN PRKCSH protein (Fragment) OS=Homo sapiens OX=9606 GN=PRKCS
ns OX=9606 GN=NID1 PE=1 SV=3;tr|A0A384MR25|A0A384MR25_HUMAN Epididymis secretory spe
se OS=Homo sapiens OX=9606 GN=HEL-S-30 PE=2 SV=1;sp|P14618-2|KPYM_HUMAN Isoform M1 of
2_HUMAN Epididymis luminal protein 35 OS=Homo sapiens OX=9606 GN=HEL-S-125m PE=2 SV=1;s
SV=1;sp|P15151-3|PVR_HUMAN Isoform Gamma of Poliovirus receptor OS=Homo sapiens OX=960
ZAH9|B7ZAH9_HUMAN cDNA, FLJ79193, highly similar to Beta-1,4-galactosyltransferase 1 OS=Homo
dymis secretory protein Li 105 OS=Homo sapiens OX=9606 GN=HEL-S-105 PE=2 SV=1;tr|Q6NUR7|Q6

0VK57_HUMAN Metalloproteinase inhibitor 2 OS=Homo sapiens OX=9606 PE=2 SV=1;sp|P16035|TI
en OS=Homo sapiens OX=9606 GN=CD44;sp|P16070-5|CD44_HUMAN Isoform 5 of CD44 antigen OS
a (Fragment) OS=Homo sapiens OX=9606 PE=2 SV=1;sp|P16144-4|ITB4_HUMAN Isoform Beta-4D of

ulin OS=Homo sapiens OX=9606 GN=VCL PE=3 SV=1;sp|P18206-2|VINC_HUMAN Isoform 1 of Vincul
JMAN Phosphoglycerate mutase OS=Homo sapiens OX=9606 GN=PGAM1 PE=2 SV=1;tr|Q53G35|Q5
rm CRA_c OS=Homo sapiens OX=9606 GN=FST PE=4 SV=1;sp|P19883|FST_HUMAN Follistatin OS=Ho

aining protein OS=Homo sapiens OX=9606 PE=2 SV=1;tr|A0A384ME06|A0A384ME06_HUMAN Epidi
OX=9606 GN=FLNA PE=1 SV=4;sp|P21333-2|FLNA_HUMAN Isoform 2 of Filamin-A OS=Homo sapien
piens OX=9606 GN=MDK PE=1 SV=1;tr|E9PLM6|E9PLM6_HUMAN Midkine OS=Homo sapiens OX=9
X=9606 PE=2 SV=1;tr|A8K7E0|A8K7E0_HUMAN Biglycan OS=Homo sapiens OX=9606 PE=2 SV=1;tr|C
Tetraspanin OS=Homo sapiens OX=9606 GN=CD9 PE=1 SV=1;tr|A0A2R8Y478|A0A2R8Y478_HUMAN
sapiens OX=9606 GN=CDH3 PE=1 SV=2;tr|B4DLF0|B4DLF0_HUMAN cDNA FLJ50795, highly similar
N Nucleoside diphosphate kinase OS=Homo sapiens OX=9606 GN=NME1-NME2 PE=1 SV=1;tr|Q6FHN
OS=Homo sapiens OX=9606 PE=2 SV=1;sp|P35321|SPR1A_HUMAN Cornifin-A OS=Homo sapiens O
24R1U8_HUMAN Insulin-like growth factor-binding protein 4 OS=Homo sapiens OX=9606 GN=IGFBP
piens OX=9606 GN=FBLN1;tr|B1AHL2|B1AHL2_HUMAN Fibulin-1 OS=Homo sapiens OX=9606 GN=F
Alpha-6X1A of Integrin alpha-6 OS=Homo sapiens OX=9606 GN=ITGA6;sp|P23229-5|ITA6_HUMAN I

on-muscle isoform (Fragment) OS=Homo sapiens OX=9606 GN=CFL1 PE=1 SV=8;sp|P23528|COF1_H
no sapiens OX=9606 GN=TNC;tr|F5H7V9|F5H7V9_HUMAN Tenascin OS=Homo sapiens OX=9606 GN

Proteasome subunit alpha type OS=Homo sapiens OX=9606 GN=PSMA3 PE=2 SV=1;tr|A0A140VK43
N Proteasome endopeptidase complex OS=Homo sapiens OX=9606 GN=PSMA4 PE=1 SV=1;tr|B2RDG
cDNA FLJ60385, highly similar to Integrin alpha-3 OS=Homo sapiens OX=9606 PE=2 SV=1;tr|A0A140

OS=Homo sapiens OX=9606 GN=HEL-S-99n PE=2 SV=1;sp|P27797|CALR_HUMAN Calreticulin OS=Ho

'roteasome subunit alpha type OS=Homo sapiens OX=9606 PE=2 SV=1;sp|P28066|PSA5_HUMAN Pr
· to Granulins OS=Homo sapiens OX=9606 PE=2 SV=1;sp|P28799-2|GRN_HUMAN Isoform 2 of Progr

MAN Receptor protein-tyrosine kinase OS=Homo sapiens OX=9606 GN=EPHA2 PE=4 SV=1;sp|P29317
N Transketolase variant (Fragment) OS=Homo sapiens OX=9606 PE=2 SV=1;sp|P29401|TKT_HUMAN
'hosphatidylethanolamine-binding protein 1 OS=Homo sapiens OX=9606 GN=PEBP1 PE=1 SV=3;tr|V9
isomerase OS=Homo sapiens OX=9606 PE=2 SV=1;tr|V9HVY3|V9HVY3_HUMAN Protein disulfide-iso

ıpiens OX=9606 GN=SDC4 PE=1 SV=2;tr|M1VE83|M1VE83_HUMAN Tyrosine-protein kinase recepto
=1;tr|H0YBG7|H0YBG7_HUMAN Heterogeneous nuclear ribonucleoprotein H (Fragment) OS=Homo s
N Epididymis secretory protein Li 1 OS=Homo sapiens OX=9606 GN=HEL-S-1 PE=2 SV=1;sp|P31946|

=Homo sapiens OX=9606 PE=2 SV=1;sp|P31949|S10AB_HUMAN Protein S100-A11 OS=Homo sapier
MAN Isoform 2 of Neural cell adhesion molecule L1 OS=Homo sapiens OX=9606 GN=L1CAM;tr|Q7Z3Z
iens OX=9606 GN=GPC1 PE=1 SV=2;tr|H7C410|H7C410_HUMAN Glypican-1 OS=Homo sapiens OX=9

X=9606 GN=FBN2 PE=1 SV=1;sp|P35556-2|FBN2_HUMAN Isoform 2 of Fibrillin-2 OS=Homo sapiens
V=1;sp|P35579|MYH9_HUMAN Myosin-9 OS=Homo sapiens OX=9606 GN=MYH9 PE=1 SV=4;tr|A0A
iN=SERPINB5 PE=3 SV=1;sp|P36952|SPB5_HUMAN Serpin B5 OS=Homo sapiens OX=9606 GN=SERPI
N Transgelin OS=Homo sapiens OX=9606 PE=2 SV=1;sp|P37802|TAGL2_HUMAN Transgelin-2 OS=Ho
:OIA1_HUMAN Isoform 3 of Collagen alpha-1(XVIII) chain OS=Homo sapiens OX=9606 GN=COL18A1;t
dehydrogenase, cytoplasmic OS=Homo sapiens OX=9606 GN=MDH1 PE=1 SV=4;sp|P40925-3|MDHC
MAN Ras GTPase-activating-like protein IQGAP1 OS=Homo sapiens OX=9606 GN=IQGAP1 PE=1 SV=1;t
N F-actin-capping protein subunit beta OS=Homo sapiens OX=9606 GN=CAPZB PE=1 SV=1;tr|B2R7T8
_HUMAN Isoform 2 of Tissue factor pathway inhibitor 2 OS=Homo sapiens OX=9606 GN=TFPI2;sp|P4
yacyl-[acyl-carrier-protein] dehydratase OS=Homo sapiens OX=9606 GN=FASN PE=1 SV=1

MAN Isoform 2 of Rab GDP dissociation inhibitor beta OS=Homo sapiens OX=9606 GN=GDI2;tr|Q6IA
MAN Basal cell adhesion molecule (Lutheran blood group) OS=Homo sapiens OX=9606 GN=BCAM PE
/E8_HUMAN Epididymis secretory sperm binding protein Li 47e OS=Homo sapiens OX=9606 GN=HEL

MAN Isoform A of Microfibrillar-associated protein 2 OS=Homo sapiens OX=9606 GN=MFAP2;tr|A0A
lipid transfer protein, isoform CRA_c OS=Homo sapiens OX=9606 GN=PLTP PE=2 SV=1;sp|P55058-4|
ATPase p97 subunit OS=Homo sapiens OX=9606 GN=HEL-S-70 PE=2 SV=1;sp|P55072|TERA_HUMAN
A8K878_HUMAN cDNA FLJ77177, highly similar to Homo sapiens arginine-rich, mutated in early stag

mo sapiens OX=9606 GN=CDH13 PE=1 SV=1;tr|B7Z9B1|B7Z9B1_HUMAN Cadherin-13 OS=Homo sa
=Homo sapiens OX=9606 GN=CD81 PE=1 SV=1;tr|H0YDL9|H0YDL9_HUMAN Tetraspanin (Fragment
e isomerase OS=Homo sapiens OX=9606 GN=HEL-S-49 PE=2 SV=1;tr|Q53HE2|Q53HE2_HUMAN Trios
oteasome subunit alpha type OS=Homo sapiens OX=9606 GN=PSMA6 PE=1 SV=1;tr|A0A140VK44|A0
bulin OS=Homo sapiens OX=9606 PE=2 SV=1;tr|A6XMH4|A6XMH4_HUMAN Beta-2-microglobulin OS
_HUMAN Epididymal secretory protein E1 OS=Homo sapiens OX=9606 GN=NPC2 PE=2 SV=1;sp|P619
4168 fis, clone NT2RP2001440, highly similar to 14-3-3 protein gamma OS=Homo sapiens OX=9606 P
s luminal protein 2 OS=Homo sapiens OX=9606 GN=HEL2 PE=2 SV=1;tr|G9K389|G9K389_HUMAN Y
=Homo sapiens OX=9606 GN=TMSB4X PE=1 SV=2;tr|Q0P5T0|Q0P5T0_HUMAN TMSB4X protein (Fra

mo sapiens OX=9606 GN=HIST1H4J PE=2 SV=1;sp|P62805|H4_HUMAN Histone H4 OS=Homo sapien
ptidyl-prolyl cis-trans isomerase OS=Homo sapiens OX=9606 PE=2 SV=1;tr|V9HWF5|V9HWF5_HUM
3_HUMAN Peptidylprolyl isomerase OS=Homo sapiens OX=9606 GN=FKBP1A PE=1 SV=1;tr|A0A087V
protein zeta/delta OS=Homo sapiens OX=9606 GN=YWHAZ PE=1 SV=1;tr|E7EX29|E7EX29_HUMAN
HUMAN cDNA FLJ57283, highly similar to Actin, cytoplasmic 2 OS=Homo sapiens OX=9606 PE=2 SV=

p|P78324|SHPS1_HUMAN Tyrosine-protein phosphatase non-receptor type substrate 1 OS=Homo s
=Homo sapiens OX=9606 PE=2 SV=1;tr|Q4KMR2|Q4KMR2_HUMAN Delta-like protein OS=Homo sap
MAN Neutrophil gelatinase-associated lipocalin OS=Homo sapiens OX=9606 GN=NGAL PE=2 SV=1;tr|
leobindin-2 OS=Homo sapiens OX=9606 GN=NUCB2;sp|P80303|NUCB2_HUMAN Nucleobindin-2 OS
s OX=9606 GN=FBLN2 PE=1 SV=2;tr|B7Z9B8|B7Z9B8_HUMAN cDNA FLJ56912, highly similar to Fibu

T0R6_HUMAN Adenylyl cyclase-associated protein 1 (Fragment) OS=Homo sapiens OX=9606 GN=CAF
Heregulin-gamma protein isoform 1 (Fragment) OS=Homo sapiens OX=9606 GN=NRG1 PE=2 SV=1;sp

ı-lysine,2-oxoglutarate 5-dioxygenase 1 OS=Homo sapiens OX=9606 GN=PLOD1 PE=1 SV=2;sp|Q028
S=Homo sapiens OX=9606 PE=2 SV=1;tr|Q53GX6|Q53GX6_HUMAN Nucleobindin-1 (Fragment) OS=
)A140VJE9|A0A140VJE9_HUMAN Testicular tissue protein Li 23 OS=Homo sapiens OX=9606 PE=2 SV
_HUMAN Peroxiredoxin-1 OS=Homo sapiens OX=9606 GN=PRDX1 PE=1 SV=1;tr|A0A0A0MSI0|A0A0
oform 2 of Collagen alpha-1(XVI) chain OS=Homo sapiens OX=9606 GN=COL16A1;sp|Q07092|COGA
|DDR1_HUMAN Isoform 2 of Epithelial discoidin domain-containing receptor 1 OS=Homo sapiens O
LG3BP_HUMAN Galectin-3-binding protein OS=Homo sapiens OX=9606 GN=LGALS3BP PE=1 SV=1;tr|
Z6K2_HUMAN Polypeptide N-acetylgalactosaminyltransferase OS=Homo sapiens OX=9606 PE=2 SV=1
PE=4 SV=1;sp|Q12805-2|FBLN3_HUMAN Isoform 2 of EGF-containing fibulin-like extracellular matri
2 of Follistatin-related protein 1 OS=Homo sapiens OX=9606 GN=FSTL1;tr|B4DTZ8|B4DTZ8_HUMAN
in 1, isoform CRA_a OS=Homo sapiens OX=9606 GN=CNTN1 PE=3 SV=1;sp|Q12860|CNTN1_HUMAN
phorin 3F variant (Fragment) OS=Homo sapiens OX=9606 PE=2 SV=1;sp|Q13275-2|SEM3F_HUMAN
PTK7_HUMAN Isoform 5 of Inactive tyrosine-protein kinase 7 OS=Homo sapiens OX=9606 GN=PTK7;s
of Mesothelin OS=Homo sapiens OX=9606 GN=MSLN;sp|Q13421-3|MSLN_HUMAN Isoform 2 of Me
1;sp|Q13443-2|ADAM9_HUMAN Isoform 2 of Disintegrin and metalloproteinase domain-containing

rm Short of Laminin subunit gamma-2 OS=Homo sapiens OX=9606 GN=LAMC2;tr|B4DLD1|B4DLD1_

3-3|GAS6_HUMAN Isoform 3 of Growth arrest-specific protein 6 OS=Homo sapiens OX=9606 GN=G

V3X5|G3V3X5_HUMAN Latent-transforming growth factor beta-binding protein 2 OS=Homo sapiens
_HUMAN Epididymis secretory sperm binding protein OS=Homo sapiens OX=9606 PE=2 SV=1;sp|Q1
no sapiens OX=9606 GN=PLEC;sp|Q15149-9|PLEC_HUMAN Isoform 9 of Plectin OS=Homo sapiens O
HUMAN Isoform Gamma of Nectin-1 OS=Homo sapiens OX=9606 GN=NECTIN1;sp|Q15223-2|NECT1_
C3|B4DHC3_HUMAN cDNA FLJ61254, highly similar to Receptor-type tyrosine-protein phosphatase k
3I PE=2 SV=1;sp|Q15582|BGH3_HUMAN Transforming growth factor-beta-induced protein ig-h3 OS=
JMAN Isoform 2 of Insulin-like growth factor-binding protein 7 OS=Homo sapiens OX=9606 GN=IGFB
ılar matrix protein 1 OS=Homo sapiens OX=9606 GN=ECM1 PE=1 SV=2;sp|Q16610-4|ECM1_HUMAN
X=9606 PE=2 SV=1;sp|Q16658|FSCN1_HUMAN Fascin OS=Homo sapiens OX=9606 GN=FSCN1 PE=1
Isoform 2 of Glutaminyl-peptide cyclotransferase OS=Homo sapiens OX=9606 GN=QPCT;tr|B5MCZ9|
HUMAN Isoform 1 of Laminin subunit alpha-3 OS=Homo sapiens OX=9606 GN=LAMA3;sp|Q16787|L
I Isoform 2 of Transmembrane protein 132A OS=Homo sapiens OX=9606 GN=TMEM132A;sp|Q24JP5
1 OS=Homo sapiens OX=9606 GN=ANXA8L1 PE=2 SV=2;tr|A0A075B752|A0A075B752_HUMAN Ann
2 of UL-16 binding protein 5 OS=Homo sapiens OX=9606 GN=RAET1G;sp|Q5VY80|ULBP6_HUMAN U
4 SV=1;sp|Q6PCB0|VWA1_HUMAN von Willebrand factor A domain-containing protein 1 OS=Homo

OS=Homo sapiens OX=9606 GN=CD109 PE=1 SV=2;sp|Q6YHK3-2|CD109_HUMAN Isoform 2 of CD10
606 PE=2 SV=1;tr|A0A024R127|A0A024R127_HUMAN Adhesion molecule with Ig-like domain 2, isof
N Isoform 5 of Lipolysis-stimulated lipoprotein receptor OS=Homo sapiens OX=9606 GN=LSR;tr|S4R
N cDNA FLJ55979, highly similar to Homo sapiens AE binding protein 1 (AEBP1), mRNA OS=Homo sa

0A669KBG0_HUMAN Proprotein convertase 9 OS=Homo sapiens OX=9606 GN=PCSK9 PE=1 SV=1
=1;tr|B4DHD2|B4DHD2_HUMAN cDNA FLJ55458, highly similar to Programmed cell death 6-interacti
gment) OS=Homo sapiens OX=9606 GN=FAM3C PE=1 SV=1;tr|C9JMN4|C9JMN4_HUMAN Protein FA
omolog (Fragment) OS=Homo sapiens OX=9606 GN=PXDN PE=1 SV=1;sp|Q92626-2|PXDN_HUMAN
tein (Fragment) OS=Homo sapiens OX=9606 GN=HTRA1 PE=2 SV=1;tr|B3KRN4|B3KRN4_HUMAN cD
UMAN Isoform 3 of Neuronal cell adhesion molecule OS=Homo sapiens OX=9606 GN=NRCAM;sp|Q9
3 of Golgi apparatus protein 1 OS=Homo sapiens OX=9606 GN=GLG1;sp|Q92896-2|GSLG1_HUMAN I
|Q96FE7-4|P3IP1_HUMAN Isoform 4 of Phosphoinositide-3-kinase-interacting protein 1 OS=Homo s
disease protein 7 OS=Homo sapiens OX=9606 GN=PARK7 PE=1 SV=2;tr|K7ELW0|K7ELW0_HUMAN
ptidase OS=Homo sapiens OX=9606 GN=LGMN PE=2 SV=1;tr|Q53XC6|Q53XC6_HUMAN Asparaginyl
Collagen alpha-1(XII) chain OS=Homo sapiens OX=9606 GN=COL12A1 PE=1 SV=1;sp|Q99715|COCA1

MAN Thioredoxin domain-containing protein 17 OS=Homo sapiens OX=9606 GN=TXNDC17 PE=1 SV=
45 kDa calcium-binding protein OS=Homo sapiens OX=9606 GN=SDF4 PE=1 SV=1;sp|Q9BRK5-3|CAB4
_HUMAN Tubulointerstitial nephritis antigen-like OS=Homo sapiens OX=9606 GN=TINAGL1 PE=1 SV=
Z22|Q86Z22_HUMAN Epididymis secretory protein Li 297 OS=Homo sapiens OX=9606 GN=HEL-S-297
-4B OS=Homo sapiens OX=9606 GN=SEMA4B PE=1 SV=4;tr|H0YMZ3|H0YMZ3_HUMAN Semaphorin-
n-2 OS=Homo sapiens OX=9606 GN=TENM2;tr|G3V106|G3V106_HUMAN HCG38403, isoform CRA_b
_HUMAN Cysteine-rich motor neuron 1 protein OS=Homo sapiens OX=9606 GN=CRIM1 PE=1 SV=1
WL2_HUMAN PTGFRN protein (Fragment) OS=Homo sapiens OX=9606 GN=PTGFRN PE=2 SV=1;tr|Q4
lated protein 3 OS=Homo sapiens OX=9606 GN=DKK3 PE=1 SV=1;tr|O43532|O43532_HUMAN RIG-li

arboxypeptidase A4 OS=Homo sapiens OX=9606 GN=CPA4;tr|A4D1M3|A4D1M3_HUMAN Carboxype
N Collagen, type XVII, alpha 1, isoform CRA_b OS=Homo sapiens OX=9606 GN=COL17A1 PE=2 SV=1;
HUMAN Endothelial protein C receptor (Fragment) OS=Homo sapiens OX=9606 GN=PROCR PE=1 SV=
A2I2MP49|A0A2I2MP49_HUMAN Kallikrein-related peptidase 5 transcript variant 6 OS=Homo sapie
homolog 2 OS=Homo sapiens OX=9606 GN=LOXL2 PE=1 SV=1;tr|W8QRJ0|W8QRJ0_HUMAN Lysyl o
N Suppressor of tumorigenicity 14 protein OS=Homo sapiens OX=9606 GN=ST14 PE=1 SV=2;tr|Q8W
tional adhesion molecule 1 OS=Homo sapiens OX=9606 GN=F11R PE=2 SV=1;sp|Q9Y624|JAM1_HUM

4N7_HUMAN Kallikrein 10, isoform CRA_a OS=Homo sapiens OX=9606 GN=KLK10 PE=4 SV=1;tr|W8
4LE33_HUMAN TNC variant protein (Fragment) OS=Homo sapiens OX=9606 GN=TNC variant protein
SV=1;sp|P98160|PGBM_HUMAN Basement membrane-specific heparan sulfate proteoglycan core p
3|A0A0B4U5E3_HUMAN Granulocyte colony-stimulating factor (Fragment) OS=Homo sapiens OX=96
MAN MHC class I antigen OS=Homo sapiens OX=9606 GN=HLA-C PE=3 SV=1;tr|A0A5H2UZM2|A0A5H
HUMAN;tr|E5FQ47|E5FQ47_HUMAN;tr|A0A0F7RPE2|A0A0F7RPE2_HUMAN;tr|D1MYY9|D1MYY9_H

mplement subcomponent C1r (Fragment) OS=Homo sapiens OX=9606 PE=2 SV=1;tr|Q53HT9|Q53H

, highly similar to Homo sapiens ribonuclease, RNase A family, 7 (RNASE7), mRNA OS=Homo sapiens

p|P14780|MMP9_HUMAN Matrix metalloproteinase-9 OS=Homo sapiens OX=9606 GN=MMP9 PE=1

N Urokinase-type plasminogen activator (Fragment) OS=Homo sapiens OX=9606 PE=2 SV=1;sp|P007
C class I antigen (Fragment) OS=Homo sapiens OX=9606 GN=HLA-C PE=3 SV=1;tr|B4DVY0|B4DVY0_H
GFA_HUMAN Isoform VEGF189 of Vascular endothelial growth factor A OS=Homo sapiens OX=9606
DNA FLJ34961 fis, clone NTONG2003839, highly similar to CONNECTIVE TISSUE GROWTH FACTOR OS

HUMAN;tr|A0A5H2UFF7|A0A5H2UFF7_HUMAN;tr|A0A3S6RFC0|A0A3S6RFC0_HUMAN;tr|A0A6B7H

GP6|A0A6B7HGP6_HUMAN;tr|A0A6B9VTX5|A0A6B9VTX5_HUMAN;tr|A0A5H2UF70|A0A5H2UF70_

_HUMAN;tr|A0A4Y5P8Y7|A0A4Y5P8Y7_HUMAN;tr|A0A4P2SQZ3|A0A4P2SQZ3_HUMAN;tr|A0A4P2

SQQ2|A0A4P2SQQ2_HUMAN;tr|A0A3S6RIB1|A0A3S6RIB1_HUMAN;tr|A0A6B7HGP2|A0A6B7HGP2_

_HUMAN;tr|A0A583ZAW1|A0A583ZAW1_HUMAN;tr|A0A3G2Y4K4|A0A3G2Y4K4_HUMAN;tr|A0A6[illegible]

B9VR96|A0A6B9VR96_HUMAN;tr|A0A6B7FTB7|A0A6B7FTB7_HUMAN;tr|A0A223FL72|A0A223FL72

2_HUMAN;tr|A0A6C0SE86|A0A6C0SE86_HUMAN;tr|A0A6C0SEA1|A0A6C0SEA1_HUMAN;tr|B1PKY3

|B1PKY3_HUMAN;tr|A0A4Y5ULL4|A0A4Y5ULL4_HUMAN;tr|A0A4P2STA1|A0A4P2STA1_HUMAN;tr

|A0A4P2ST16|A0A4P2ST16_HUMAN;tr|A0A6B7HHR8|A0A6B7HHR8_HUMAN;tr|A0A3S6REZ3|A0A3

S6REZ3_HUMAN;tr|A0A2P9FIT1|A0A2P9FIT1_HUMAN;tr|A0A2U3QLE8|A0A2U3QLE8_HUMAN;tr|A

A0A2U3QLG4|A0A2U3QLG4_HUMAN;tr|A0A2U3QLL1|A0A2U3QLL

LFQ intens	LFQ intens	LFQ intens	LFQ intens	LFQ intens	LFQ intens	LFQ intens	LFQ intens	LFQ intens
30,68529	30,28703	30,85964	30,81879	30,90928	30,77941	30,85823	30,37399	30,38577
26,97235	27,53875	23,70641	22,08734	23,46811	22,2817	22,63029	27,70695	27,51427
22,93884	22,8041	29,19943	28,65775	22,67563	28,79383	29,29743	22,48849	23,56157
28,38961	29,04604	27,39198	27,05734	28,2615	27,86071	27,11128	28,68201	29,20016
31,3198	32,1689	30,87943	30,74903	30,78659	30,80301	30,76256	31,63901	31,60342
34,12325	32,30138	33,52869	33,55225	33,47255	32,45425	32,37485	31,58352	31,52687
31,38469	31,11873	31,19578	31,53723	31,60116	31,29576	31,11471	32,09704	32,07728
29,73119	27,79749	28,15938	27,87701	28,09628	28,40255	28,18778	28,98829	29,14239
24,22059	25,35528	25,29018	24,60156	24,26556	24,5428	24,76157	24,10556	22,01563
25,24107	23,69519	23,87995	25,01504	24,50355	23,90812	24,18526	26,31443	26,06079
23,67721	22,78137	23,41876	26,10536	26,18569	25,36903	25,84886	23,16877	21,86169
29,76917	28,95427	28,57236	29,82902	30,14722	29,61994	30,10762	31,08759	31,39622
29,96939	29,87926	28,24984	28,92269	29,53094	29,31214	29,26177	30,53853	30,19531
23,84414	24,56426	24,14361	26,90047	27,01797	23,04612	26,99194	23,29207	21,7728
23,23996	24,51734	23,0489	24,38555	22,96906	23,09225	23,3615	21,63014	21,64979
28,62048	28,59463	28,16322	28,52099	28,36035	28,34617	28,57015	27,47212	27,3232
29,03701	27,4182	27,61744	30,29428	30,01478	29,87616	30,71498	26,92717	27,37103
26,29667	25,03846	24,52492	25,97248	25,33504	25,60096	26,30412	24,09491	24,71654
29,24704	28,01919	28,46486	27,12475	27,50127	27,40162	27,56761	28,76853	29,01962
26,1668	27,14763	26,99043	26,60442	26,65335	27,12839	27,13193	25,5854	25,88229
23,27266	23,05515	24,49419	28,20527	23,44089	22,41971	23,52586	28,58692	28,11098
26,7406	27,39648	27,47885	26,64636	26,88367	27,06569	27,16873	27,61147	27,93061
28,80999	28,71509	28,60918	28,03239	28,31825	28,30138	28,43583	29,45138	29,59557
21,37512	22,7043	21,79578	23,34055	23,29488	23,16682	23,1216	21,44602	21,38864
21,08503	22,76161	23,87801	23,80747	22,82011	21,57761	22,86011	27,84356	27,88681
28,76202	29,10482	27,51923	29,5136	29,89116	30,42722	29,85855	29,03499	29,16816
35,727	35,08001	34,21993	35,31125	35,36708	34,7692	34,89767	35,99507	36,21522
28,86847	25,22255	23,93872	28,67949	28,82271	28,34875	28,94827	29,42582	30,35174
29,18348	29,40099	28,37952	29,30898	29,31954	29,29999	29,52232	30,40304	30,03486
22,35912	23,95871	21,64258	23,57819	22,99253	21,29182	21,36832	24,78731	24,35057
25,23356	26,33668	24,68886	24,4084	24,56209	26,16092	25,99401	23,71963	24,19764
30,33947	28,09894	28,59385	30,34794	29,98726	29,70336	30,16591	29,10734	29,01404
27,87079	28,04323	27,51668	27,61996	27,93326	27,3379	27,7082	29,23938	29,22041

LFQ intens	LFQ intens	Potential c	Student's T	Student's T	Peptides	Razor + un	Unique pe	Sequence c
30,30171	30,06201		+	2_3	18	18	3	26,5
27,15528	27,30669		+	2_3	8	8	6	30,8
21,97597	22,60404		+	2_3	3	3	3	11,3
28,69604	29,52217		+	2_3	5	5	5	31,7
31,44618	31,4631		+	2_3	8	8	8	83,2
31,4446	31,74186		+	2_3	16	16	16	42
32,10793	32,37233		+	2_3	22	22	22	54,9
29,07694	28,57261		+	2_3	12	12	12	10
22,38684	22,3639		+	2_3	4	4	3	10,7
25,89375	25,32711		+	2_3	6	6	6	35,2
24,02359	22,4964		+	2_3	2	2	2	21,4
30,8017	30,96237		+	2_3	8	8	8	53,4
30,52318	30,37389		+	2_3	13	13	4	30,9
23,14555	22,7834		+	2_3	2	2	2	2,7
21,57464	22,0971		+	2_3	2	2	2	12
27,91239	27,94665		+	2_3	12	12	12	25,8
27,52447	26,34316		+	2_3	21	18	18	24,7
24,94864	24,56889		+	2_3	3	3	1	9,6
28,78176	29,24681		+	2_3	7	7	7	6,7
25,67694	26,49496		+	2_3	5	5	5	20,7
28,78924	27,59662		+	2_3	3	3	3	25,7
27,73596	27,54768		+	2_3	5	5	5	21
29,28701	29,04679		+	2_3	7	7	7	17,1
21,26774	22,32003		+	2_3	4	4	4	31,3
27,65055	23,1283		+	2_3	7	5	5	18,3
28,96058	28,41216		+	2_3	11	11	11	35,6
36,06305	35,62631		+	2_3	61	61	61	61,7
30,30726	29,43418		+	2_3	28	28	28	17,4
29,72728	30,19507		+	2_3	9	9	9	29,4
24,67632	24,27049		+	2_3	7	7	7	7,9
23,18006	22,52028		+	2_3	4	4	4	17,4
29,35323	29,58923		+	2_3	5	5	1	4,8
29,24386	28,63159		+	2_3	12	12	12	41

Unique + r	Unique sec	Mol. weigh	Q-value	Score	Intensity	MS/MS co	iBAQ 1-1	iBAQ 1-3
26,5	4,4	82,577	0	195,5	1,26E+10	179	54165000	12210000
30,8	28,4	37,371	0	10,842	9,27E+08	27	8190000	9313400
11,3	11,3	47,984	0	2,6777	2,77E+09	7	23685000	17189000
31,7	31,7	32,212	0	108,45	2,79E+09	30	31201000	18398000
83,2	83,2	16,057	0	60,299	2,01E+10	126	3,41E+08	2,58E+08
42	42	53,981	0	323,31	6,47E+10	186	1,07E+09	1,67E+08
54,9	54,9	47,472	0	221,05	2,57E+10	201	56633000	29258000
10	10	167,55	0	175,05	3,33E+09	86	10641000	1712800
10,7	8,6	70,289	0	49,458	1,44E+08	16	321640	435020
35,2	35,2	20,195	0	36,023	2,77E+08	23	1245600	1639600
21,4	21,4	12,269	0	4,7231	1,89E+08	9	0	0
53,4	53,4	14,374	0	29,647	1E+10	38	95637000	32647000
30,9	8,7	70,154	0	67,45	7,24E+09	83	33291000	12880000
2,7	2,7	81,996	0	4,2977	5,09E+08	7	1133600	804460
12	12	26,425	0	6,001	47823000	6	1015500	284770
25,8	25,8	67,877	0	41,025	2,24E+09	49	10391000	13131000
21,6	21,6	129,99	0	141,34	4,38E+09	75	12295000	1243100
9,6	4,5	42,1	0	23,912	3,13E+08	26	2549200	869580
6,7	6,7	135,51	0	79,196	2,89E+09	58	9983300	823220
20,7	20,7	36,426	0	23,244	7,43E+08	26	11018000	8268400
25,7	25,7	19,365	0	17,92	1,25E+09	12	0	0
21	21	36,923	0	31,222	1,28E+09	33	9219400	3546100
17,1	17,1	55,394	0	28,686	3,58E+09	38	14707000	10814000
31,3	31,3	22,588	0	13,402	44157000	17	369740	405650
16	16	40,368	0	40,17	7,29E+08	18	1903000	277100
35,6	35,6	46,231	0	107,1	4,97E+09	88	27149000	12938000
61,7	61,7	130,97	0	323,31	3,73E+11	747	9,16E+08	3,04E+08
17,4	17,4	250,53	0	117,47	4,52E+09	135	9374900	102170
29,4	29,4	51,286	0	275,36	6,29E+09	60	36163000	13834000
7,9	7,9	144,37	0	15,203	91054000	25	40472	2135,8
17,4	17,4	35,634	0	13,115	2,33E+08	21	4258900	6014000
4,8	1,2	99,978	0	22,836	6,46E+09	38	29561000	133310
41	41	38,069	0	64,295	2,72E+09	54	13368000	2663600

iBAQ 1-5	iBAQ 2-1	iBAQ 2-2	iBAQ 2-3	iBAQ 2-4	iBAQ 3-1	iBAQ 3-2	iBAQ 3-3	iBAQ 3-4
29242000	39609000	32490000	17230000	26972000	24950000	20929000	14283000	8368400
2033800	3624300	2735800	2599600	2297300	400020	8990700	4583100	6714600
39423000	14621000	14565000	7251600	14615000	0	9435800	0	12914000
10513000	204400	19545000	13218000	7775800	34127000	30843000	47751000	39640000
1,55E+08	1,92E+08	2,04E+08	99544000	77229000	4,11E+08	2,89E+08	2,88E+08	2,03E+08
1,59E+08	3,83E+08	5,26E+08	1,52E+08	1,34E+08	1,12E+08	1,32E+08	40595000	65369000
30019000	65485000	89792000	52195000	55961000	1,92E+08	1,2E+08	1,97E+08	1E+08
2335900	1838500	1734200	4253600	4157300	3882100	3745000	2287600	2609700
509920	1673100	350350	253220	301970	27477	10296	0	0
232680	886330	483090	178550	1638200	11482000	1409300	9706600	1869100
1063800	9016000	7426100	3955500	7096900	2074800	517490	0	362010
29674000	80375000	1,16E+08	42079000	81811000	1,9E+08	2,94E+08	3,28E+08	1,43E+08
4528100	11167000	17090000	19611000	10555000	45536000	30795000	35279000	28766000
0	2944800	2909900	600990	2468800	984840	2354600	0	1706800
865890	939080	67069	33608	92605	237110	204290	169660	437960
7034500	12217000	2674500	8963800	9819600	5250600	2834400	8120600	8996500
1423900	19339000	18337000	16780000	23172000	911920	1128200	1135700	1642200
419210	1406200	748630	5639800	955750	214840	430000	552740	456350
1948900	1929500	1741400	879280	1289500	8670700	8390800	7596200	6608100
3462600	4445400	3489800	3876100	5970400	2786800	1432100	116750	4681100
105440	34258000	9122300	0	0	31512000	25213000	40969000	14786000
10790000	2615500	12031000	409580	9383900	19149000	1070800	9065700	7795300
11216000	10729000	6854500	7890300	8859200	22486000	31864000	9694800	7987600
343140	951700	842810	444170	405350	250270	232330	198010	463180
0	6970200	1768200	1349200	785500	13768000	12798000	3233600	0
4480700	35552000	46108000	43277000	22996000	22419000	18940000	21083000	6627200
1,39E+08	5,7E+08	5,05E+08	2,63E+08	2,72E+08	9,2E+08	8,35E+08	7,71E+08	5,14E+08
11247	2089000	4025700	2553100	389920	8701000	8097200	8049000	10453000
8268700	40633000	37509000	16472000	19337000	50362000	39359000	30740000	21783000
21210	126400	78488	13311	4421,6	397330	213590	275180	296080
2969700	211360	210550	320570	293730	95702	57033	50182	59157
171330	17590000	16631000	12359000	19762000	13544000	7727300	6778300	7545500
3127200	10215000	6946400	1788900	3031000	21819000	14093000	21828000	9978000

Student's T	Student's T	Student's T	Student's T	Majority pr	Fasta headers
0,000428	0,0246	0,560557	3,11013	sp|O00391	sp|O00391|QSOX1_HUMAN Sulfhydryl oxida
6,37E-06	0	-4,80394	-11,2219	tr|B2R717	tr|B2R717|B2R717_HUMAN cDNA, FLJ9323
0,02611	0,0492	4,698645	2,762629	tr|B4DY05	tr|B4DY05|B4DY05_HUMAN cDNA FLJ5830
0,00669	0,022824	-1,45239	-3,16983	tr|B3KNF2	tr|B3KNF2|B3KNF2_HUMAN cDNA FLJ1448
5,15E-06	0,011	-0,76263	-5,07655	tr|Q5H9A7	tr|Q5H9A7|Q5H9A7_HUMAN Metalloprotei
0,005153	0,013143	1,389258	3,277427	tr|Q5TZP0	tr|Q5TZP0|Q5TZP0_HUMAN Matrix metallo
0,001075	0,014154	-0,77643	-3,34502	tr|Q53H39	tr|Q53H39|Q53H39_HUMAN Activin beta-A
0,003054	0,02887	-0,80415	-2,99913	tr|A0A024	tr|A0A024RDW8|A0A024RDW8_HUMAN Co
0,009072	0,025895	1,824889	3,138353	tr|V9HWJ7	tr|V9HWJ7|V9HWJ7_HUMAN Epididymis se
0,00324	0,003667	-1,49603	-3,59101	tr|A0A024	tr|A0A024R8V7|A0A024R8V7_HUMAN Met
0,000966	0,0088	2,989621	4,99568	sp|P19957	sp|P19957|ELAF_HUMAN Elafin OS=Homo s
0,000671	0,0044	-1,13602	-4,10434	tr|E9PPJ5|	tr|E9PPJ5|E9PPJ5_HUMAN Midkine (Fragme
0,000248	0,0055	-1,15084	-4,62101	tr|B4DUV1	tr|B4DUV1|B4DUV1_HUMAN Fibulin-1 OS=H
0,020636	0,043143	3,240671	2,844386	tr|Q59F03	tr|Q59F03|Q59F03_HUMAN Integrin alpha 3
0,002483	0,004	1,714172	3,863522	tr|Q5U0A0	tr|Q5U0A0|Q5U0A0_HUMAN Proteasome s
0,003263	0,037333	0,785824	2,948189	tr|V9HWD	tr|V9HWD9|V9HWD9_HUMAN Epididymis l
6,31E-05	0	3,183593	7,523012	sp|P35442	sp|P35442|TSP2_HUMAN Thrombospondin-
0,004635	0,025867	1,220905	3,227093	tr|A0A024	tr|A0A024R2B6|A0A024R2B6_HUMAN Serp
4,62E-05	0	-1,55537	-6,23382	tr|D3DSM4	tr|D3DSM4|D3DSM4_HUMAN Collagen, typ
0,00831	0,050897	0,969624	2,763653	tr|V9HWF2	tr|V9HWF2|V9HWF2_HUMAN Malate dehy
0,026208	0,049125	-3,87301	-2,72592	tr|Q8NE89	tr|Q8NE89|Q8NE89_HUMAN Similar to tissu
0,001696	0,021556	-0,76532	-3,15908	sp|P52799	sp|P52799|EFNB2_HUMAN Ephrin-B2 OS=H
0,000317	0,004889	-1,07323	-4,3736	sp|P78324	sp|P78324-2|SHPS1_HUMAN Isoform 2 of T
0,000585	0,006286	1,625356	4,690488	sp|P80188	sp|P80188|NGAL_HUMAN Neutrophil gelati
0,02168	0,044741	-3,86098	-2,85194	tr|E9PKG6	tr|E9PKG6|E9PKG6_HUMAN Nucleobindin-2
0,006421	0,03792	1,028663	2,926995	tr|Q59G97	tr|Q59G97|Q59G97_HUMAN EGF-containin
0,003797	0,027818	-0,88861	-3,01943	sp|Q13753	sp|Q13753|LAMC2_HUMAN Laminin subun
0,006606	0,026286	-1,17995	-3,02393	sp|Q6UVK	sp|Q6UVK1|CSPG4_HUMAN Chondroitin su
0,003061	0,040769	-0,72736	-2,8846	sp|Q92743	sp|Q92743|HTRA1_HUMAN Serine protease
0,009528	0,02425	-2,21346	-3,20553	sp|Q92823	sp|Q92823-5|NRCAM_HUMAN Isoform 5 of
0,018537	0,049548	1,876957	2,735875	tr|B7Z5J4|	tr|B7Z5J4|B7Z5J4_HUMAN Carboxypeptidas
0,005931	0,049212	0,785157	2,72015	tr|A8K6T3	tr|A8K6T3|A8K6T3_HUMAN cDNA FLJ78674
0,000321	0,007333	-1,43398	-4,86586	tr|Q5M8T4	tr|Q5M8T4|Q5M8T4_HUMAN Connective ti

ase 1 OS=Homo sapiens OX=9606 GN=QSOX1 PE=1 SV=3;tr|A0A140VKE5|A0A140VKE5_HUMAN Sulf
5, highly similar to Homo sapiens cathepsin L2 (CTSL2), mRNA OS=Homo sapiens OX=9606 PE=2 SV=1
3, highly similar to WD repeat protein 1 OS=Homo sapiens OX=9606 PE=2 SV=1;sp|O75083-3|WDR1_
4 fis, clone MAMMA1002485, highly similar to Stanniocalcin-2 OS=Homo sapiens OX=9606 PE=2 SV=1
inase inhibitor 1 OS=Homo sapiens OX=9606 GN=TIMP1 PE=1 SV=1;tr|Q6FGX5|Q6FGX5_HUMAN Me
proteinase 1 (Interstitial collagenase) OS=Homo sapiens OX=9606 PE=2 SV=1;tr|Q53G96|Q53G96_H
chain (Fragment) OS=Homo sapiens OX=9606 PE=2 SV=1;tr|A4D1W7|A4D1W7_HUMAN Activin bet
ollagen, type IV, alpha 2, isoform CRA_a OS=Homo sapiens OX=9606 GN=COL4A2 PE=4 SV=1;sp|P085
cretory protein Li 37 OS=Homo sapiens OX=9606 GN=HEL-S-37 PE=2 SV=1;tr|Q53FI1|Q53FI1_HUMA
alloproteinase inhibitor 2 OS=Homo sapiens OX=9606 GN=TIMP2 PE=3 SV=1;tr|A0A140VK57|A0A14

ent) OS=Homo sapiens OX=9606 GN=MDK PE=1 SV=1;sp|P21741|MK_HUMAN Midkine OS=Homo sa
Homo sapiens OX=9606 PE=2 SV=1;sp|P23142-4|FBLN1_HUMAN Isoform C of Fibulin-1 OS=Homo sa
3 isoform b, variant (Fragment) OS=Homo sapiens OX=9606 PE=2 SV=1;tr|B4E0H8|B4E0H8_HUMAN
subunit alpha type OS=Homo sapiens OX=9606 PE=2 SV=1;tr|A0A109NGN6|A0A109NGN6_HUMAN P
uminal protein 107 OS=Homo sapiens OX=9606 GN=HEL107 PE=2 SV=1;tr|Q53EM5|Q53EM5_HUMA
-2 OS=Homo sapiens OX=9606 GN=THBS2 PE=1 SV=2;tr|A0A3B3ITK0|A0A3B3ITK0_HUMAN Thrombo
oin peptidase inhibitor, clade B (Ovalbumin), member 5, isoform CRA_b OS=Homo sapiens OX=9606 G
e XVIII, alpha 1, isoform CRA_d OS=Homo sapiens OX=9606 GN=COL18A1 PE=4 SV=1;sp|P39060-2|C
drogenase OS=Homo sapiens OX=9606 GN=HEL-S-32 PE=2 SV=1;sp|P40925|MDHC_HUMAN Malate
ue factor pathway inhibitor 2 (Fragment) OS=Homo sapiens OX=9606 PE=2 SV=1;sp|P48307-2|TFPI2_

yrosine-protein phosphatase non-receptor type substrate 1 OS=Homo sapiens OX=9606 GN=SIRPA;s
inase-associated lipocalin OS=Homo sapiens OX=9606 GN=LCN2 PE=1 SV=2;tr|B2ZDQ1|B2ZDQ1_HU
2 OS=Homo sapiens OX=9606 GN=NUCB2 PE=1 SV=2;sp|P80303-2|NUCB2_HUMAN Isoform 2 of Nuc
g fibulin-like extracellular matrix protein 1 isoform b variant (Fragment) OS=Homo sapiens OX=9606
it gamma-2 OS=Homo sapiens OX=9606 GN=LAMC2 PE=1 SV=2;sp|Q13753-2|LAMC2_HUMAN Isofor

e HTRA1 OS=Homo sapiens OX=9606 GN=HTRA1 PE=1 SV=1;tr|Q05DJ8|Q05DJ8_HUMAN HTRA1 prot
f Neuronal cell adhesion molecule OS=Homo sapiens OX=9606 GN=NRCAM;sp|Q92823-3|NRCAM_HU
se A4 OS=Homo sapiens OX=9606 GN=CPA4 PE=1 SV=1;sp|Q9UI42-2|CBPA4_HUMAN Isoform 2 of Ca
4, highly similar to Homo sapiens desmocollin type 4 OS=Homo sapiens OX=9606 PE=2 SV=1
issue growth factor OS=Homo sapiens OX=9606 GN=CTGF PE=2 SV=1;tr|B3KRV6|B3KRV6_HUMAN cl

fhydryl oxidase OS=Homo sapiens OX=9606 GN=QSCN6 PE=2 SV=1;sp|O00391-2|QSOX1_HUMAN Isc
L;tr|A0A024R141|A0A024R141_HUMAN Cathepsin L2, isoform CRA_a OS=Homo sapiens OX=9606 G
_HUMAN Isoform 2 of WD repeat-containing protein 1 OS=Homo sapiens OX=9606 GN=WDR1;tr|B3K
L;tr|Q6FHC9|Q6FHC9_HUMAN STC2 protein (Fragment) OS=Homo sapiens OX=9606 GN=STC2 PE=2
etalloproteinase inhibitor 1 OS=Homo sapiens OX=9606 GN=TIMP1 PE=2 SV=1;tr|B3KQF4|B3KQF4_H
UMAN Matrix metalloproteinase 1 preproprotein variant (Fragment) OS=Homo sapiens OX=9606 PE
a-A chain OS=Homo sapiens OX=9606 GN=INHBA PE=2 SV=1;sp|P08476|INHBA_HUMAN Inhibin beta
72|CO4A2_HUMAN Collagen alpha-2(IV) chain OS=Homo sapiens OX=9606 GN=COL4A2 PE=1 SV=4;t
N L-plastin variant (Fragment) OS=Homo sapiens OX=9606 PE=2 SV=1;tr|A0A024RDT4|A0A024RDT4
0VK57_HUMAN Metalloproteinase inhibitor 2 OS=Homo sapiens OX=9606 PE=2 SV=1;sp|P16035|TII

piens OX=9606 GN=MDK PE=1 SV=1;tr|E9PLM6|E9PLM6_HUMAN Midkine OS=Homo sapiens OX=96
piens OX=9606 GN=FBLN1;tr|B1AHL2|B1AHL2_HUMAN Fibulin-1 OS=Homo sapiens OX=9606 GN=FE
cDNA FLJ60385, highly similar to Integrin alpha-3 OS=Homo sapiens OX=9606 PE=2 SV=1;tr|A0A140
Proteasome subunit alpha type OS=Homo sapiens OX=9606 PE=2 SV=1;sp|P28066|PSA5_HUMAN Pr
N Transketolase variant (Fragment) OS=Homo sapiens OX=9606 PE=2 SV=1;sp|P29401|TKT_HUMAN

GN=SERPINB5 PE=3 SV=1;sp|P36952|SPB5_HUMAN Serpin B5 OS=Homo sapiens OX=9606 GN=SERPI
OIA1_HUMAN Isoform 3 of Collagen alpha-1(XVIII) chain OS=Homo sapiens OX=9606 GN=COL18A1;t
dehydrogenase, cytoplasmic OS=Homo sapiens OX=9606 GN=MDH1 PE=1 SV=4;sp|P40925-3|MDHC
_HUMAN Isoform 2 of Tissue factor pathway inhibitor 2 OS=Homo sapiens OX=9606 GN=TFPI2;sp|P4

p|P78324|SHPS1_HUMAN Tyrosine-protein phosphatase non-receptor type substrate 1 OS=Homo s
MAN Neutrophil gelatinase-associated lipocalin OS=Homo sapiens OX=9606 GN=NGAL PE=2 SV=1;tr|
leobindin-2 OS=Homo sapiens OX=9606 GN=NUCB2;sp|P80303|NUCB2_HUMAN Nucleobindin-2 OS
PE=4 SV=1;sp|Q12805-2|FBLN3_HUMAN Isoform 2 of EGF-containing fibulin-like extracellular matri
rm Short of Laminin subunit gamma-2 OS=Homo sapiens OX=9606 GN=LAMC2;tr|B4DLD1|B4DLD1_

tein (Fragment) OS=Homo sapiens OX=9606 GN=HTRA1 PE=2 SV=1;tr|B3KRN4|B3KRN4_HUMAN cDN
UMAN Isoform 3 of Neuronal cell adhesion molecule OS=Homo sapiens OX=9606 GN=NRCAM;sp|Q9
arboxypeptidase A4 OS=Homo sapiens OX=9606 GN=CPA4;tr|A4D1M3|A4D1M3_HUMAN Carboxype

DNA FLJ34961 fis, clone NTONG2003839, highly similar to CONNECTIVE TISSUE GROWTH FACTOR OS

506 GN=MDK PE=1 SV=1;sp|P21741-2|MK_HUMAN Isoform 2 of
3LN1 PE=1 SV=1;sp|P23142-2|FBLN1_HUMAN Isoform A

oteasome subunit alpha type-5 OS=Homo sapiens OX=96
N Transketolase OS=Homo sapiens OX=9606 GN=TKT PE=

_HUMAN Isoform 3 of Malate dehydrogenase, cytoplasmic

=Homo sapiens OX=9606 GN=NUCB2 PE=1 SV=3;tr|Q2L696|

HUMAN cDNA FLJ57342, highly similar to Laminin gamma

eptidase A4 OS=Homo sapiens OX=9606 GN=CPA4 PE=2 SV
