# Supplementary material for: hTERT-Driven Immortalization of RDEB Fibroblast and Keratinocyte Cell Lines Followed by Cre-Mediated Transgene Elimination
Source: Int J Mol Sci. 2021 Apr 7;22(8):3809. doi: 10.3390/ijms22083809 (PMC8067634; doi:10.3390/ijms22083809)
Supplement: Supplementary file 1 [file ijms-22-03809-s001.zip › sup/Table S3.pdf]

node1	node2	node1 acc	node2 acc	node1 ann	node2 ann	score
WDR1	TXNDC17	ENSP0000	ENSP0000	WD repeat	Thioredoxi	0,816
WDR1	FLNA	ENSP0000	ENSP0000	WD repeat	Filamin-A;	0,439
TXNDC17	WDR1	ENSP0000	ENSP0000	Thioredoxi	WD repeat	0,816
TIMP2	THBS2	ENSP0000	ENSP0000	Metallopr	Thrombos	0,435
TIMP2	QPCT	ENSP0000	ENSP0000	Metallopr	Glutaminyl	0,904
TIMP2	PLAT	ENSP0000	ENSP0000	Metallopr	Tissue-typ	0,488
TIMP2	NID1	ENSP0000	ENSP0000	Metallopr	Nidogen-1	0,443
TIMP2	MMP1	ENSP0000	ENSP0000	Metallopr	Interstitial	0,868
TIMP2	LCN2	ENSP0000	ENSP0000	Metallopr	Neutrophil	0,954
TIMP2	ITGA3	ENSP0000	ENSP0000	Metallopr	Integrin al	0,438
TIMP2	CXCL1	ENSP0000	ENSP0000	Metallopr	Growth-re	0,938
TIMP2	CTGF	ENSP0000	ENSP0000	Metallopr	Connectiv	0,588
TIMP2	COL6A1	ENSP0000	ENSP0000	Metallopr	Collagen al	0,404
TIMP2	COL18A1	ENSP0000	ENSP0000	Metallopr	Collagen al	0,504
THBS2	TIMP2	ENSP0000	ENSP0000	Thrombos	Metallopr	0,435
THBS2	ITGA3	ENSP0000	ENSP0000	Thrombos	Integrin al	0,738
THBS2	FBLN1	ENSP0000	ENSP0000	Thrombos	Fibulin-1;	0,421
THBS2	CTGF	ENSP0000	ENSP0000	Thrombos	Connectiv	0,504
THBS2	COL6A1	ENSP0000	ENSP0000	Thrombos	Collagen al	0,571
THBS2	COL18A1	ENSP0000	ENSP0000	Thrombos	Collagen al	0,556

THBS2	AEBP1	ENSP0000	ENSP0000	Thrombos	Adipocyte	0,585
TFPI2	PLAT	ENSP0000	ENSP0000	Tissue fact	Tissue-typ	0,528
TFPI2	MMP1	ENSP0000	ENSP0000	Tissue fact	Interstitial	0,509
STC2	NUCB1	ENSP0000	ENSP0000	Stanniocal	Nucleobin	0,902
SERPINB5	PI3	ENSP0000	ENSP0000	Serpin B5;	Elafin; Neu	0,61
SERPINB5	CLIC1	ENSP0000	ENSP0000	Serpin B5;	Chloride in	0,451
SERPINB2	PLAT	ENSP0000	ENSP0000	Plasminog	Tissue-typ	0,963
SERPINB2	MMP1	ENSP0000	ENSP0000	Plasminog	Interstitial	0,531
SERPINB2	CXCL1	ENSP0000	ENSP0000	Plasminog	Growth-re	0,427
SDF4	NUCB1	ENSP0000	ENSP0000	45 kDa cal	Nucleobin	0,476
RAET1G	CD109	ENSP0000	ENSP0000	UL-16 bind	CD109 ant	0,901
QPCT	TIMP2	ENSP0000	ENSP0000	Glutaminyl	Metallopr	0,904
QPCT	LCN2	ENSP0000	ENSP0000	Glutaminyl	Neutrophil	0,901
QPCT	CXCL1	ENSP0000	ENSP0000	Glutaminyl	Growth-re	0,906
PLAT	TIMP2	ENSP0000	ENSP0000	Tissue-typ	Metallopr	0,488
PLAT	TFPI2	ENSP0000	ENSP0000	Tissue-typ	Tissue fact	0,528
PLAT	SERPINB2	ENSP0000	ENSP0000	Tissue-typ	Plasminog	0,963
PLAT	MMP1	ENSP0000	ENSP0000	Tissue-typ	Interstitial	0,501
PKM	LDHB	ENSP0000	ENSP0000	Pyruvate k	Lactate del	0,958
PKM	CLIC1	ENSP0000	ENSP0000	Pyruvate k	Chloride in	0,565

PI3	SERPINB5	ENSP0000	ENSP0000	Elafin; Neu	Serpin B5;	0,61
PI3	LCN2	ENSP0000	ENSP0000	Elafin; Neu	Neutrophil	0,501
PCDH7	NID1	ENSP0000	ENSP0000	Protocadh	Nidogen-1	0,54
NUCB1	STC2	ENSP0000	ENSP0000	Nucleobin	Stanniocal	0,902
NUCB1	SDF4	ENSP0000	ENSP0000	Nucleobin	45 kDa cal	0,476
NRCAM	CNTN1	ENSP0000	ENSP0000	Neuronal c	Contactin-	0,903
NID1	TIMP2	ENSP0000	ENSP0000	Nidogen-1	Metallopr	0,443

NID1	PCDH7	ENSP0000(	ENSP0000(	Nidogen-1,	Protocadh	0,54
NID1	MMP1	ENSP0000(	ENSP0000(	Nidogen-1,	Interstitial	0,935
NID1	LGALS3BP	ENSP0000(	ENSP0000(	Nidogen-1,	Galectin-3-	0,506
NID1	LAMC2	ENSP0000(	ENSP0000(	Nidogen-1,	Laminin su	0,591
NID1	LAMA1	ENSP0000(	ENSP0000(	Nidogen-1,	Laminin su	0,972
NID1	ITGA3	ENSP0000(	ENSP0000(	Nidogen-1,	Integrin al	0,931
NID1	FBLN1	ENSP0000(	ENSP0000(	Nidogen-1,	Fibulin-1; I	0,595
NID1	COL6A1	ENSP0000(	ENSP0000(	Nidogen-1,	Collagen al	0,434
NID1	COL18A1	ENSP0000(	ENSP0000(	Nidogen-1,	Collagen al	0,577
MMP1	TIMP2	ENSP0000(	ENSP0000(	Interstitial	Metallopro	0,868
MMP1	TFPI2	ENSP0000(	ENSP0000(	Interstitial	Tissue fact	0,509
MMP1	SERPINB2	ENSP0000(	ENSP0000(	Interstitial	Plasminoge	0,531
MMP1	PLAT	ENSP0000(	ENSP0000(	Interstitial	Tissue-type	0,501

MMP1	NID1	ENSP0000(	ENSP0000(	Interstitial	Nidogen-1,	0,935
MMP1	CXCL1	ENSP0000(	ENSP0000(	Interstitial	Growth-re	0,587
MMP1	CTGF	ENSP0000(	ENSP0000(	Interstitial	Connective	0,584
MMP1	COL18A1	ENSP0000(	ENSP0000(	Interstitial	Collagen al	0,404
MANF	HTRA1	ENSP0000(	ENSP0000(	Mesencepl	Serine prot	0,453
LGALS3BP	NID1	ENSP0000(	ENSP0000(	Galectin-3-	Nidogen-1,	0,506
LDHB	PKM	ENSP0000(	ENSP0000(	Lactate del	Pyruvate k	0,958
LDHB	CLIC1	ENSP0000(	ENSP0000(	Lactate del	Chloride in	0,432
LCN2	TIMP2	ENSP0000(	ENSP0000(	Neutrophil	Metallopro	0,954
LCN2	QPCT	ENSP0000(	ENSP0000(	Neutrophil	Glutaminyl	0,901
LCN2	PI3	ENSP0000(	ENSP0000(	Neutrophil	Elafin; Neu	0,501
LCN2	CXCL1	ENSP0000(	ENSP0000(	Neutrophil	Growth-re	0,959
LCN2	CSF3	ENSP0000(	ENSP0000(	Neutrophil	Granulocyt	0,441
LAMC2	NID1	ENSP0000(	ENSP0000(	Laminin su	Nidogen-1,	0,591
LAMC2	LAMA1	ENSP0000(	ENSP0000(	Laminin su	Laminin su	0,915
LAMC2	ITGA3	ENSP0000(	ENSP0000(	Laminin su	Integrin al	0,955
LAMC2	FBLN1	ENSP0000(	ENSP0000(	Laminin su	Fibulin-1; I	0,456
LAMA1	NID1	ENSP0000(	ENSP0000(	Laminin su	Nidogen-1,	0,972
LAMA1	LAMC2	ENSP0000(	ENSP0000(	Laminin su	Laminin su	0,915
LAMA1	L1CAM	ENSP0000(	ENSP0000(	Laminin su	Neural cell	0,902

LAMA1	ITGA3	ENSP0000(	ENSP0000(	Laminin su	Integrin al	0,956
LAMA1	COL6A1	ENSP0000(	ENSP0000(	Laminin su	Collagen al	0,47
LAMA1	COL18A1	ENSP0000(	ENSP0000(	Laminin su	Collagen al	0,918
L1CAM	LAMA1	ENSP0000(	ENSP0000(	Neural cell	Laminin su	0,902
L1CAM	CNTN1	ENSP0000(	ENSP0000(	Neural cell	Contactin-	0,961
ITGA3	TIMP2	ENSP0000(	ENSP0000(	Integrin al	Metallopro	0,438
ITGA3	THBS2	ENSP0000(	ENSP0000(	Integrin al	Thrombos	0,738
ITGA3	NID1	ENSP0000(	ENSP0000(	Integrin al	Nidogen-1,	0,931
ITGA3	LAMC2	ENSP0000(	ENSP0000(	Integrin al	Laminin su	0,955
ITGA3	LAMA1	ENSP0000(	ENSP0000(	Integrin al	Laminin su	0,956
ITGA3	FLNA	ENSP0000(	ENSP0000(	Integrin al	Filamin-A;	0,71
ITGA3	CSPG4	ENSP0000(	ENSP0000(	Integrin al	Chondroiti	0,918
ITGA3	COL6A1	ENSP0000(	ENSP0000(	Integrin al	Collagen al	0,726
ITGA3	COL18A1	ENSP0000(	ENSP0000(	Integrin al	Collagen al	0,92
IL1R2	CXCL1	ENSP0000(	ENSP0000(	Interleukin	Growth-re	0,489

HTRA1	MANF	ENSP0000(	ENSP0000(	Serine prot	Mesencepl	0,453
FLNA	WDR1	ENSP0000(	ENSP0000(	Filamin-A;	WD repeat	0,439
FLNA	ITGA3	ENSP0000(	ENSP0000(	Filamin-A;	Integrin alp	0,71
FBLN1	THBS2	ENSP0000(	ENSP0000(	Fibulin-1; I	Thrombosp	0,421
FBLN1	NID1	ENSP0000(	ENSP0000(	Fibulin-1; I	Nidogen-1,	0,595

FBLN1	LAMC2	ENSP0000(	ENSP0000(	Fibulin-1; I	Laminin su	0,456
FBLN1	EFEMP1	ENSP0000(	ENSP0000(	Fibulin-1; I	EGF-contai	0,935
FBLN1	CTGF	ENSP0000(	ENSP0000(	Fibulin-1; I	Connective	0,592
FBLN1	COL6A1	ENSP0000(	ENSP0000(	Fibulin-1; I	Collagen al	0,552
FBLN1	COL18A1	ENSP0000(	ENSP0000(	Fibulin-1; I	Collagen al	0,489
EFEMP1	FBLN1	ENSP0000(	ENSP0000(	EGF-contai	Fibulin-1; I	0,935
EFEMP1	CTGF	ENSP0000(	ENSP0000(	EGF-contai	Connective	0,533
EFEMP1	AEBP1	ENSP0000(	ENSP0000(	EGF-contai	Adipocyte	0,514
CXCL1	TIMP2	ENSP0000(	ENSP0000(	Growth-reg	Metallopro	0,938
CXCL1	SERPINB2	ENSP0000(	ENSP0000(	Growth-reg	Plasminoge	0,427
CXCL1	QPCT	ENSP0000(	ENSP0000(	Growth-reg	Glutaminyl	0,906
CXCL1	MMP1	ENSP0000(	ENSP0000(	Growth-reg	Interstitial	0,587
CXCL1	LCN2	ENSP0000(	ENSP0000(	Growth-reg	Neutrophil	0,959
CXCL1	IL1R2	ENSP0000(	ENSP0000(	Growth-reg	Interleukin	0,489
CXCL1	CTGF	ENSP0000(	ENSP0000(	Growth-reg	Connective	0,44
CXCL1	CSF3	ENSP0000(	ENSP0000(	Growth-reg	Granulocyt	0,802
CTSV	COL18A1	ENSP0000(	ENSP0000(	Cathepsin	Collagen al	0,918
CTGF	TIMP2	ENSP0000(	ENSP0000(	Connective	Metallopro	0,588
CTGF	THBS2	ENSP0000(	ENSP0000(	Connective	Thrombosp	0,504
CTGF	MMP1	ENSP0000(	ENSP0000(	Connective	Interstitial	0,584

CTGF	FBLN1	ENSP0000(	ENSP0000(	Connective	Fibulin-1; I	0,592
CTGF	EFEMP1	ENSP0000(	ENSP0000(	Connective	EGF-contai	0,533
CTGF	CXCL1	ENSP0000(	ENSP0000(	Connective	Growth-reg	0,44
CTGF	COL6A1	ENSP0000(	ENSP0000(	Connective	Collagen al	0,413
CTGF	COL18A1	ENSP0000(	ENSP0000(	Connective	Collagen al	0,414
CSPG4	ITGA3	ENSP0000(	ENSP0000(	Chondroiti	Integrin alp	0,918
CSPG4	COL6A1	ENSP0000(	ENSP0000(	Chondroiti	Collagen al	0,472
CSF3	LCN2	ENSP0000(	ENSP0000(	Granulocyt	Neutrophil	0,441
CSF3	CXCL1	ENSP0000(	ENSP0000(	Granulocyt	Growth-reg	0,802
CSF3	CLIC1	ENSP0000(	ENSP0000(	Granulocyt	Chloride in	0,416
COL6A1	TIMP2	ENSP0000(	ENSP0000(	Collagen al	Metallopro	0,404
COL6A1	THBS2	ENSP0000(	ENSP0000(	Collagen al	Thrombosp	0,571
COL6A1	NID1	ENSP0000(	ENSP0000(	Collagen al	Nidogen-1,	0,434
COL6A1	LAMA1	ENSP0000(	ENSP0000(	Collagen al	Laminin su	0,47
COL6A1	ITGA3	ENSP0000(	ENSP0000(	Collagen al	Integrin alp	0,726
COL6A1	FBLN1	ENSP0000(	ENSP0000(	Collagen al	Fibulin-1; I	0,552
COL6A1	CTGF	ENSP0000(	ENSP0000(	Collagen al	Connective	0,413
COL6A1	CSPG4	ENSP0000(	ENSP0000(	Collagen al	Chondroiti	0,472
COL6A1	COL18A1	ENSP0000(	ENSP0000(	Collagen al	Collagen al	0,933
COL18A1	TIMP2	ENSP0000(	ENSP0000(	Collagen al	Metallopro	0,504

COL18A1	THBS2	ENSP0000(	ENSP0000(	Collagen al	Thrombosp	0,556
COL18A1	NID1	ENSP0000(	ENSP0000(	Collagen al	Nidogen-1,	0,577

COL18A1	MMP1	ENSP0000(	ENSP0000(	Collagen al	Interstitial	0,404
COL18A1	LAMA1	ENSP0000(	ENSP0000(	Collagen al	Laminin su	0,918
COL18A1	ITGA3	ENSP0000(	ENSP0000(	Collagen al	Integrin alp	0,92
COL18A1	FBLN1	ENSP0000(	ENSP0000(	Collagen al	Fibulin-1; I	0,489
COL18A1	CTSV	ENSP0000(	ENSP0000(	Collagen al	Cathepsin	0,918
COL18A1	CTGF	ENSP0000(	ENSP0000(	Collagen al	Connective	0,414
COL18A1	COL6A1	ENSP0000(	ENSP0000(	Collagen al	Collagen al	0,933
CNTN1	NRCAM	ENSP0000(	ENSP0000(	Contactin-:	Neuronal c	0,903
CNTN1	L1CAM	ENSP0000(	ENSP0000(	Contactin-:	Neural cell	0,961
CLIC1	SERPINB5	ENSP0000(	ENSP0000(	Chloride in	Serpin B5;	0,451
CLIC1	PKM	ENSP0000(	ENSP0000(	Chloride in	Pyruvate k	0,565
CLIC1	LDHB	ENSP0000(	ENSP0000(	Chloride in	Lactate del	0,432
CLIC1	CSF3	ENSP0000(	ENSP0000(	Chloride in	Granulocyt	0,416
CD109	RAET1G	ENSP0000(	ENSP0000(	CD109 ant	UL-16 bind	0,901
AEBP1	THBS2	ENSP0000(	ENSP0000(	Adipocyte	Thrombos	0,585
AEBP1	EFEMP1	ENSP0000(	ENSP0000(	Adipocyte	EGF-contai	0,514

node1	node2	node1 acc	node2 acc	node1 ann	node2 ann	score
COL18A1	COL6A1	ENSP0000(	ENSP0000(	Collagen al	Collagen al	0,933
COL18A1	CTGF	ENSP0000(	ENSP0000(	Collagen al	Connective	0,414
COL18A1	CTSV	ENSP0000(	ENSP0000(	Collagen al	Cathepsin	0,918
COL18A1	LAMA1	ENSP0000(	ENSP0000(	Collagen al	Laminin su	0,918
COL18A1	MMP1	ENSP0000(	ENSP0000(	Collagen al	Interstitial	0,404
COL18A1	TIMP2	ENSP0000(	ENSP0000(	Collagen al	Metallopr	0,504
COL6A1	COL18A1	ENSP0000(	ENSP0000(	Collagen al	Collagen al	0,933
COL6A1	CSPG4	ENSP0000(	ENSP0000(	Collagen al	Chondroiti	0,472
COL6A1	CTGF	ENSP0000(	ENSP0000(	Collagen al	Connective	0,413
COL6A1	LAMA1	ENSP0000(	ENSP0000(	Collagen al	Laminin su	0,47
COL6A1	TIMP2	ENSP0000(	ENSP0000(	Collagen al	Metallopr	0,404
CSPG4	COL6A1	ENSP0000(	ENSP0000(	Chondroiti	Collagen al	0,472
CTGF	COL18A1	ENSP0000(	ENSP0000(	Connective	Collagen al	0,414
CTGF	COL6A1	ENSP0000(	ENSP0000(	Connective	Collagen al	0,413
CTGF	MMP1	ENSP0000(	ENSP0000(	Connective	Interstitial	0,584
CTGF	TIMP2	ENSP0000(	ENSP0000(	Connective	Metallopr	0,588
CTSV	COL18A1	ENSP0000(	ENSP0000(	Cathepsin	Collagen al	0,918
L1CAM	LAMA1	ENSP0000(	ENSP0000(	Neural cell	Laminin su	0,902
LAMA1	COL18A1	ENSP0000(	ENSP0000(	Laminin su	Collagen al	0,918
LAMA1	COL6A1	ENSP0000(	ENSP0000(	Laminin su	Collagen al	0,47
LAMA1	L1CAM	ENSP0000(	ENSP0000(	Laminin su	Neural cell	0,902
LAMA1	LAMC2	ENSP0000(	ENSP0000(	Laminin su	Laminin su	0,915
LAMC2	LAMA1	ENSP0000(	ENSP0000(	Laminin su	Laminin su	0,915
MMP1	COL18A1	ENSP0000(	ENSP0000(	Interstitial	Collagen al	0,404
MMP1	CTGF	ENSP0000(	ENSP0000(	Interstitial	Connective	0,584
MMP1	SERPINB2	ENSP0000(	ENSP0000(	Interstitial	Plasminog	0,531
MMP1	TFPI2	ENSP0000(	ENSP0000(	Interstitial	Tissue fact	0,509
MMP1	TIMP2	ENSP0000(	ENSP0000(	Interstitial	Metallopr	0,868
SERPINB2	MMP1	ENSP0000(	ENSP0000(	Plasminog	Interstitial	0,531
TFPI2	MMP1	ENSP0000(	ENSP0000(	Tissue fact	Interstitial	0,509
TIMP2	COL18A1	ENSP0000(	ENSP0000(	Metallopr	Collagen al	0,504
TIMP2	COL6A1	ENSP0000(	ENSP0000(	Metallopr	Collagen al	0,404
TIMP2	CTGF	ENSP0000(	ENSP0000(	Metallopr	Connective	0,588
TIMP2	MMP1	ENSP0000(	ENSP0000(	Metallopr	Interstitial	0,868
